# Borylated Five-Membered Ring Iminosugars: Synthesis, Spectroscopic Analysis, and Biological Evaluation for Glycosidase Inhibition and Anticancer Properties for Application in Boron Neutron Capture Therapy (BNCT)—Part 1

**DOI:** 10.3390/ph18091302

**Published:** 2025-08-29

**Authors:** Kate Prichard, Suzuka Yamamoto, Yuna Shimadate, Kosuke Yoshimura, Barbara Bartholomew, Jayne Gilbert, Jennette Sakoff, Robert Nash, Atsushi Kato, Michela Simone

**Affiliations:** 1 Discipline of Chemistry, University of Newcastle, Callaghan, NSW 2308, Australia; 2 Department of Hospital Pharmacy, University of Toyama, 2630 Sugitani, Toyama 930-0194, Japankato@med.u-toyama.ac.jp (A.K.); 3 PhytoQuest Ltd., Plas Gogerddan, Aberystwyth, Ceredigion SY23 3EB, UKrobert.nash@phytoquest.co.uk (R.N.); 4 Department of Medical Oncology, Calvary Mater Newcastle Hospital, Edith St, Waratah, NSW 2298, Australiajennette.sakoff@newcastle.edu.au (J.S.); 5 Newcastle CSIRO Energy Centre, 10 Murray Dwyer Circuit, Mayfield West, NSW 2304, Australia

**Keywords:** monosaccharide, iminosugar, boronic acid, boronate, boron, BNCT, glycosidase, cancer, Fsp^3^ index, NMR, mutarotation, borarotation, induced fit

## Abstract

**Background/Objectives**: This article reports pyrrolidine iminosugars of L-gulose absolute stereochemical configuration that are functionalised via *N*-alkylation to bear boronate ester and boronic acid pharmacophores. Inclusion of boron pharmacophores has been shown to reduce toxicity profiles of drugs and can expand the range of interactions between drugs and target enzymes. **Methods:** The synthetic development, detailed spectroscopic analysis, and biological investigation against glycosidase enzymes and cancer cell lines of these novel five-membered ring iminosugars are reported. **Results:** This family of iminosugars displays selective, moderate-to-weak inhibition (IC_50_s = 133–501 μM) of β-d-galactosidase (bovine liver) and emerging inhibition of β-d-glucosidases (almond) and (bovine liver). The boronic acid pharmacophore may be suitable for the management of lysosomal storage disorders to support the restoration of biological activity of mutant enzymes via the chaperone-mediated therapy approach. From a structure–activity perspective, the cancer screening revealed slight growth inhibition in a panel of cancer cell lines, with A2780 ovarian carcinoma cells showing the strongest response across all compounds. Beyond the growth inhibition capabilities, the real therapeutic potential of these borylated drugs lies in their switch-on/switch-off activation under BNCT radiotherapeutic conditions. **Conclusions:** This is an important novel family of drug leads capable of interacting with drug targets via intermolecular and intramolecular interactions, changing shape and electronics. Introduction of organic boron atoms to organic molecules presents significant synthetic and purification challenges, as well as analysis of the equilibria that arise in aqueous systems. We provide a methodology to achieve all this and introduce boron pharmacophores onto carbohydrate scaffolds in a systematic manner to facilitate a more widespread adoption of boron pharmacophores.

## 1. Introduction

Introduction of organic boron pharmacophores on biologically active molecules provides the opportunity to expand the profile of interactions between ligand and enzyme, and lower the toxicity of drug leads of high potency. Iminosugars are an important family of high Fsp^3^ index [1,2,3] carbohydrate-active enzyme modulators isolated from plants and bacteria. Several synthetic analogues have reached the clinic for the management of diabetes [4,5,6,7,8,9], with advances in the areas of lysosomal storage disorders [10,11,12,13,14,15], cystic fibrosis [16,17], cancer [18,19,20], and broad-spectrum antivirals [21,22,23,24,25,26,27,28]. In this last area, the viral glycan shield [29] or the *N*-glycan pathway of host cells [30,31] is targeted. This drug target is validated in vitro [21,22,23,24,25,26,27,28,30] and in vivo [24,32], and provides a crucial new avenue for new antiviral drug regimens that are: more effective in achieving clinical endpoints, not hampered by resistance, acting at a different site of action, and providing lower treatment costs.

The chemistry of iminosugars is rich, and the chemical space traditionally occupied by medicinal chemistry is greatly expanded through carbohydrate analogues [16,33,34,35,36,37,38,39,40,41,42,43]. This is primarily because carbohydrates ubiquitously mediate biological processes, and the three-dimensional structures of carbohydrates allow for exquisite fine-tuning of substrate–enzyme interactions. In the vast landscape of medicinal chemistry, a high Fsp^3^ index [1] has been identified as one of the all-encompassing factors propelling drug leads through to clinical success, with pyrrolidine iminosugars of l-*gulo* absolute stereochemical configuration displaying significant antiviral properties [3,44,45].

Inclusion of organic boron pharmacophores in our carbohydrate chemistry [46,47] and iminosugar research [22] expands the palette of interactions between drugs and enzymes through the exploitation of reversible covalent bond formation with nucleophilic atoms available in active sites or their vicinity [47,48,49,50,51].

An organic B atom, in the form of a trigonal planar boronic acid or boronate ester, in a molecule can reversibly form dative bonds with nucleophilic atoms located intramolecularly and/or intermolecularly. These interactions give rise to borarotation, the equilibrium of trigonal planar and tetrahedral boron species. The final equilibrium mixture depends on several factors, including pH, solvent, temperature, concentration, arrangement of atoms in space, and intramolecular proximity of nucleophilic atoms [47,51,52]. To comprehensively elucidate the solution (and eventual physiological) behaviour of borylated drugs, an investigation of borarotation should be included. This—in conjunction with machine learning and molecular modelling—would facilitate a greater understanding of ADME processes.

Here, we communicate the synthesis of two families of five-membered ring iminosugars (of which one is borylated) of l-*gulo* absolute stereochemical configuration via the development of synthetic and purification protocols. The target compounds display selective and moderate glycosidase inhibitions and are imbued with switch-on/switch-off BNCT capabilities. We have specialised in installing organic boron pharmacophores on high Fsp^3^ index target compounds that are packed with biological capabilities and perspective favourable ADME profiles [33,47,48,49,51,53,54,55]. Mutarotation and borarotation equilibria in aqueous/protic solutions are also investigated in detail, utilising NMR.

## 2. Results and Discussion

### 2.1. Synthesis

**Scheme 1:**
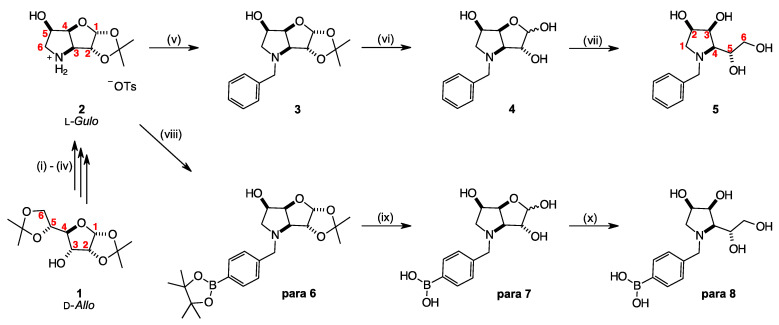
Reagents and reaction conditions: (i) (a) Pyridine, DCM; Tf_2_O, N_2_, −10 °C, 20 min; (b) NaN_3_, DMF, 50 °C, 18 h, 80.5% over two steps; (ii) AcOH/MeOH/H_2_O (4:5:6), 50 °C, 21 h, 95%; (iii) *p*-Toluenesulfonyl chloride, pyridine, N_2_, −10 °C to r.t. over 24 h, 68%; (iv) H_2_, Pd/C 10%, EtOH, r.t., 24 h, 99%. (v) **2** (1.1 eq.), benzyl bromide (1.0 eq.), K_2_CO_3_ (1.0 eq.), DMF, 100 °C, 19 h, 61%; (vi) HCl (aq. 1.0 M), r.t., 19.5 h, 99%; (vii) NaBH_4_ (2.4 eq.), EtOH (aq. 50%), r.t., 30 min; then NaBH_4_ (2.4 eq.), r.t., 17 h, 88%. (viii) **2** (1.1 eq.), 4-bromomethylphenylboronic acid pinacol ester (1.0 eq.), K_2_CO_3_ (1.0 eq.), DMF, 100 °C, 19 h, 97%; (ix) HCl (aq. 1.0 M), 40 °C, 17 h, 99%; (x) NaBH_4_ (1.4 eq., then 1.2 eq.), EtOH (aq. 50%), r.t., 1 h, 48%. The numbering of these molecular systems is shown on structures **1**, **2** and **5**.

The first four synthetic steps employed a protocol available from the literature to divergent intermediate **2** [56], where from 1,2:5,6-di-*O*-isopropylidene-α-d-allofuranose starting material **1**, iminosugar precursor **2** was achieved with an overall yield of 51.5% over four steps on a multigram scale (Scheme 1). The intermediates were 3-azido-3-deoxy-1,2;5,6-di-*O*-isopropylidene-α-d-glucofuranose [80.5% yield over two steps, [α]_289_^19^ −0.29 (c. 0.095 in MeOH)], 3-azido-3-deoxy-1,2-*O*-isopropylidene-α-d-glucofuranose [95% yield, [α]_289_^24^ −34.3 (c 0.021 in CHCl_3_), lit [56] [α]_D_^20^ −32.3 (c 0.95 in CHCl_3_), and m.p. 80–82 °C, lit [56] 86–87 °C], 3-azido-3-deoxy-1,2-*O*-isopropylidene-6-*O*-*p*-toluenesulfonyl-α-d-glucofuranose (68% yield) and 3,6-dideoxy-3,6-imino-1,2-*O*-isopropylidene-α-d-gulofuranose *p*-toluenesulfonate **2** (99% yield, [α]_289_^24^ +23.5 (c 0.0017 in H_2_O), lit [56] [α]_D_^20^ +28.1 (c 0.83 in H_2_O). M.p. 161–162 °C, lit [56] 187–188 °C]. All NMR, mass spectrometry, optical rotation, and melting point data for intermediates and precursor **2** matched the literature data.

The *N*-alkylation reaction methodology required optimisation as initial attempts employing a successful method for six-membered ring systems were found to be unsuitable for a complete reaction of the five-membered ring. Initial strategies involved a 31-h reaction time at a temperature of 50 °C, after which product formation was confirmed despite difficulties with purification, resulting in low yields of the borylated product. Attempted purifications included flash column chromatography and resin columns. Known purification challenges relating to borylated compounds are summarised in [57]. We encountered further challenges relating to wide solubility ranges for borylated intermediates in this research project and cognate ones. Various solvents, temperatures, and the number of equivalents of potassium carbonate and alkylating agent were utilised. Additional synthetic strategies involved the global deprotection of the crude *N*-alkylated product and also the global deprotection of iminosugar **2** prior to the *N*-alkylation.

The final optimised method uses a slight excess of the iminosugar starting material (1.1 eq.) compared to the base K_2_CO_3_ and benzylation reagent (benzyl bromide and 4-bromomethylphenyl boronic acid pinacol esters, 1.0 eq.). DMF was found to be the best solvent, using a concentration of 10 mg iminosugar/mL DMF. The resultant crude was dissolved in chloroform and washed with NaOH (aq. 0.1 M), requiring no column purification. This method resulted in high purity products in yields of 61% and 97%, respectively, for **3** and **para 6**.

The deprotection conditions to give the corresponding lactol were optimised using HCl (aq. 1.0 M) at temperatures which varied depending on the benzyl substitution. The deprotection of the benzylated compound **3** occurred at room temperature overnight (19.5 h), while the borylated compound **para 6** required 40 °C and a 17 h reaction time. The higher temperatures affected the global deprotection (i.e., acetonide and pinacol groups were cleaved). The free pinacol proved challenging to remove.

The final reduction step to give the target compounds **5** and **para 8** was achieved by sodium borohydride reduction of lactols **4** and **para 7**. The optimised reaction conditions for **5** were a half an hour reaction time at r.t. with NaBH_4_ (2.4 eq.), followed by an additional portion of NaBH_4_ (2.4 eq.) for 17 h. The optimised reaction conditions to **para 8** were half an hour reaction time at r.t. with NaBH_4_ (1.4 eq.), followed by an additional portion of NaBH_4_ (1.2 eq.) for a further 30 min. Several purification methods were attempted, with the most success observed from methanol/ethanol triturations.

### 2.2. ^11^B-NMR Data Analysis

**Figure 1:**
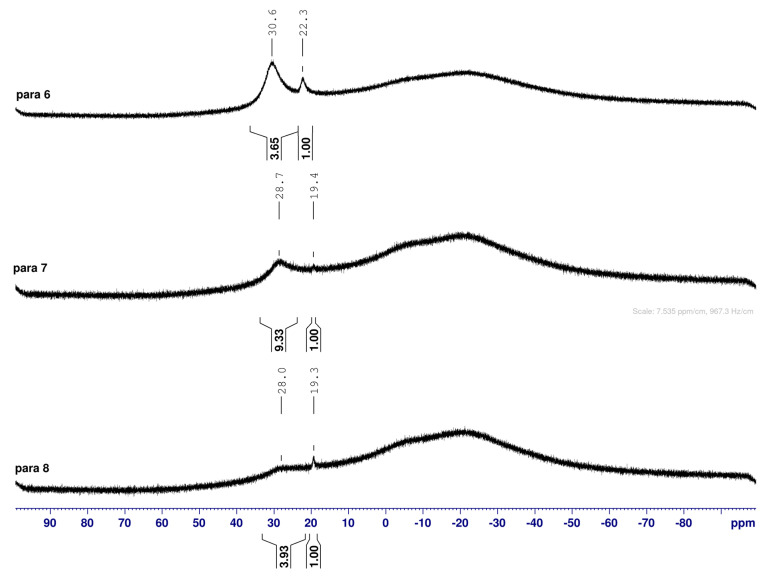
^11^B-NMR (128 MHz) spectral comparisons for library **para 6**, **para 7**, and **para 8**.

The borarotation process occurs for borylated species **para 6**, **para 7,** and **para 8**. Figure 1 shows stacked plots of ^11^B-NMR spectra of **para 6**, **para 7,** and **para 8**. The boron hump is located in the range ~10 ppm and ~−40 ppm. This arises from the borosilicate compounds contained in NMR tubes and the NMR probe [38,39,40]. The borarotation and mutarotation phenomena occur in intermediate **para 7** (with postulated equilibria shown in Table 1).

The ^11^B-NMR spectra show signals at 30.6 and 22.3 ppm (**para 6**, integration ratio 3.7:1.0), 28.7 and 19.4 ppm (**para 7**, integration ratio 9.3:1.0), and 28.0 and 19.3 ppm (**para 8**, integration ratio 3.9:1.0). In **para 7** the relative concentration of the partially tetrahedral species to the boronic acid species is reduced, compared to their relative concentrations in **para 6** and **para 8**. This is postulated to arise from a higher degree of conformational flexibility or molecular tumbling in solution upon removal of the protecting groups, which could decrease the efficacy of the interaction between nucleophilic atoms and the B empty *p*-orbital. **para 8** has a similar relative concentration of the partially tetrahedral species as in **para 6**. This is postulated to arise from more efficient hydrogen bonding interactions with nearby water molecules, after the anomeric equilibrium is abrogated.

In **para 6**, the signals at 30.6 and 22.3 ppm correspond to the boronate esters of the trigonal planar and partially tetrahedral geometries, respectively. The latter likely occurs via a CDCl_3_ molecule dative bonding to the pinacol B atom (a slight pink colour emerges, as also observed in a structurally related system under investigation in our research laboratory). In **para 7**, the spectra are complex due to the concomitant mutarotation equilibrium, where both the α-furanose (*α-fur*) and β-furanose (*β-fur*) anomers arise via the open-chain carbonyl form at C-1. At equilibrium, **para 7** exists in an *α-fur* anomer/*β-fur* anomer mutarotation ratio of ~1.0:0.4. The B atom in both anomers is found primarily in the trigonal planar boronic acid form, as indicated by the chemical shift at 28.7 ppm. The partially tetrahedral forms are found at 19.4 ppm. The partially tetrahedral species likely arise intermolecularly from a partial dative bond between the less hindered boronic acid B atom and the O atom lone pair of a D_2_O molecule. In **para 8**, it is postulated that the boronic acid signal is very broad because it encompasses several boronic acids, which are very weakly complexed to a water molecule. Weak complexation to a water molecule would shift the signal progressively upfield due to the B atom receiving more and more electron density from the O atom of the water. However, once the water molecule and B atom come close enough and a stabler interaction can be formed, this occurs and gives rise to the signal at 19.3 ppm.

**Table 1:** ^11^B-NMR data (128 MHz) highlighting the borarotation process for all **para**, **meta**, and **ortho** species and the interplay between borarotation and mutarotation for intermediates **para 7**, **meta 3**, and **ortho 3** [51]. The signal integration ratios are normalised to 1.0 for the partially tetrahedral/tetrahedral species. The most likely equilibria are shown for each chemical species. Chemical shifts in ppm. Legend: “*tet*” = tetrahedral, “*trig*” = trigonal planar.

Compound (Deuterated Solvent)	Chemical Shifts		Compound (Deuterated Solvent)	Chemical Shifts		Compound (Deuterated Solvent)	Chemical Shifts		
	Signal Integration Ratio			Signal Integration Ratio			Signal Integration Ratio		
	Signal Shape			Signal Shape			Signal Shape		
	Geometry			Geometry			Geometry		
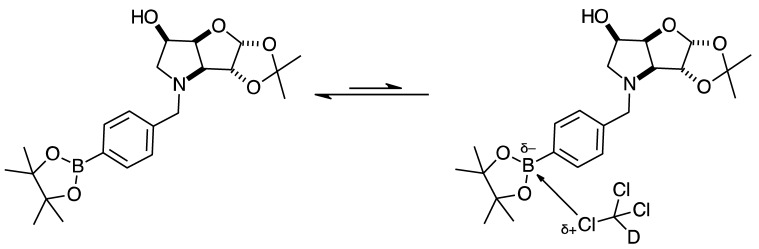 **para 6** (CDCl_3_)	30.6	22.3	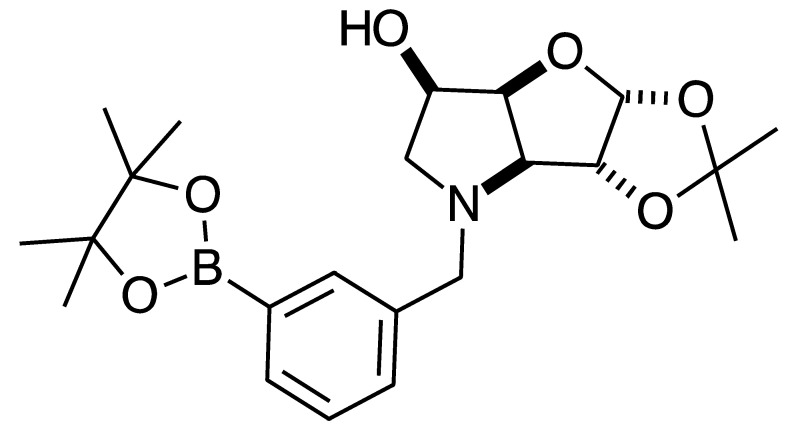 **meta 2** (CDCl_3_)	30.8		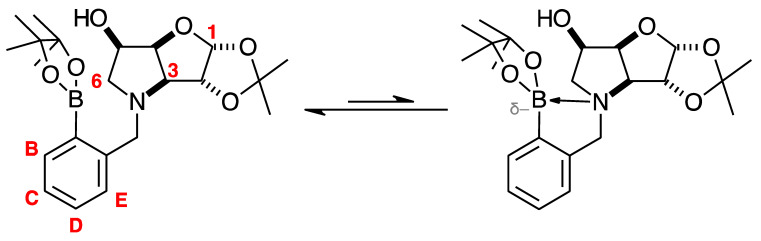 **ortho 2** (CDCl_3_)	31.0	22.3	
	3.7	1.0		NA			6.4	1.0	
	broad	sharp		Broad			sharp	sharp	
	Boronate ester (*trig*)	Boronate ester (partially *tet*)		Boronate ester (*trig*)			Boronate ester (*trig*)	Boronate ammonium (partially *tet*)	
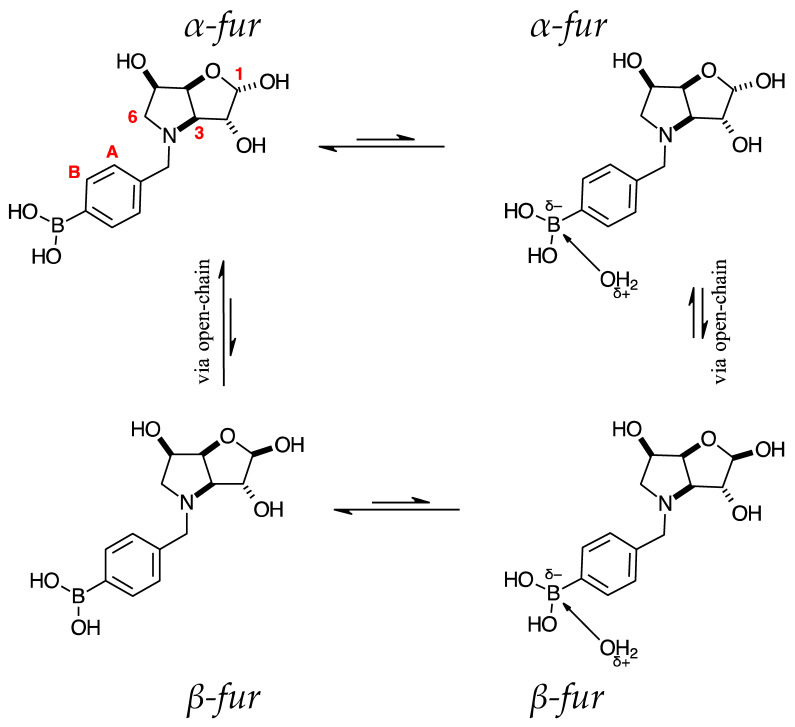 **para 7** (D_2_O) *α-fur/β-fur*, 1.0:0.4	28.7	19.4	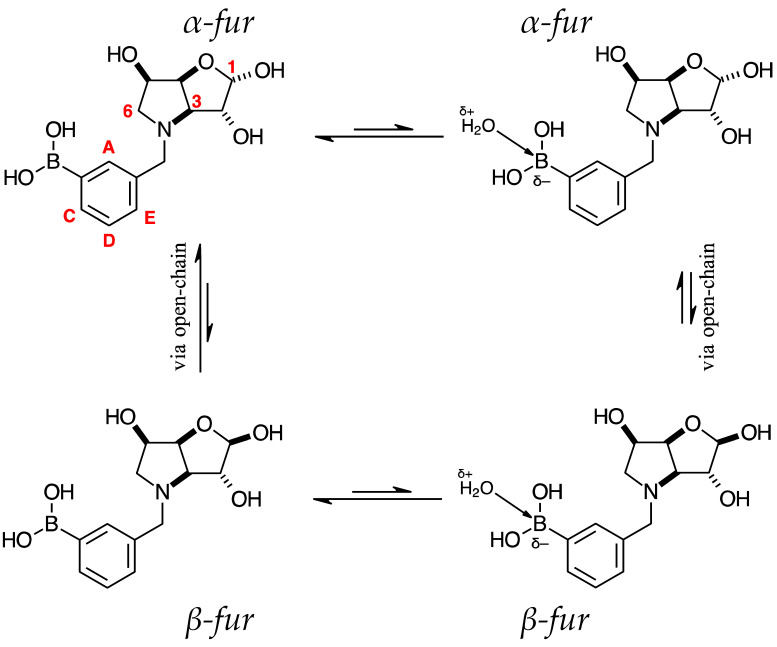 **meta 3** (D_2_O) *α-fur/β-fur*/open-chain, 1.0:0.5:0.002	28.6	19.2	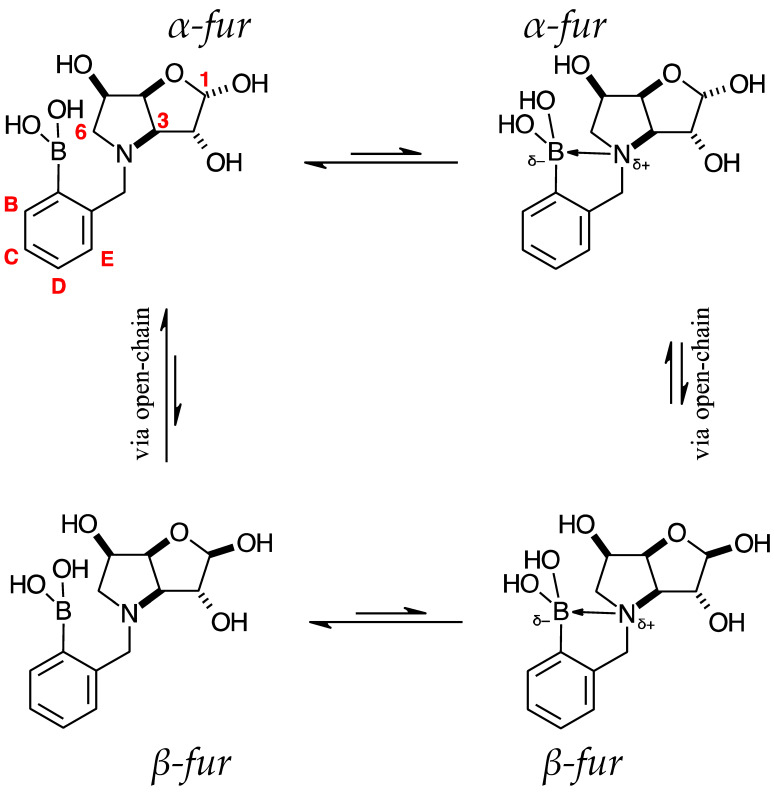 **ortho 3** (D_2_O) *α-fur:β-fur*, 1.0:0.7	28.0	19.3	
	9.3	1.0		6.5	1.0		4.0	1.0	
	broad	sharp		broad	sharp		broad	sharp	
	Boronic acid (*trig*)	Boronate (partially *tet*)		Boronic acid (*trig*)	Boronate (partially *tet*)		Boronic acid (*trig*)	Boronate (partially *tet*)	
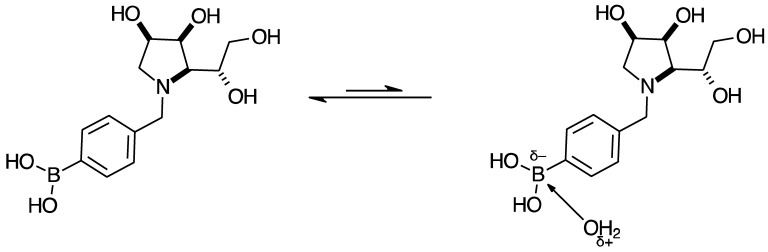 **para 8** (D_2_O)	28.0	19.3	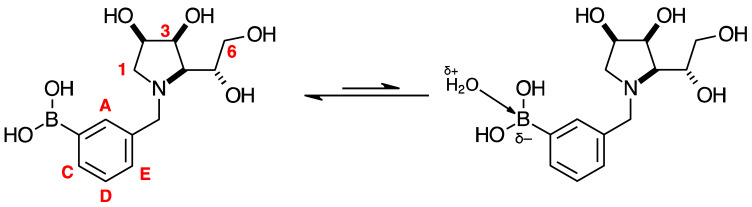 **meta 4** (D_2_O)	27.8	19.3	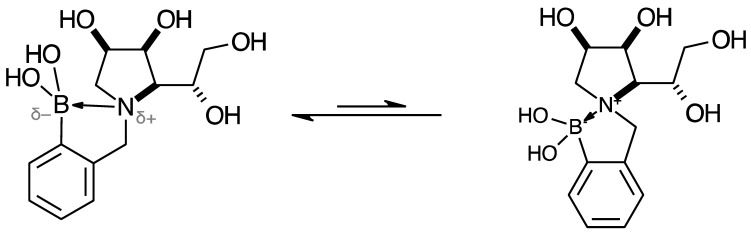 **ortho 4** (D_2_O)	*28.3*	19.4	12.3, 11.0
	3.9	1.0		1.7	1.0		*1.1*	6.1	1.2, 1.0
	broad	sharp		broad	sharp		*broad*	sharp	Sharp, merging
	Boronic acid (*trig*)	Boronate (partially *tet*)		Boronic acid (*trig*)	Boronate (partially *tet*)		*Boronic acid* *(trig)*	Boronate ammonium	
								(partially *tet*)	(*tet*)
			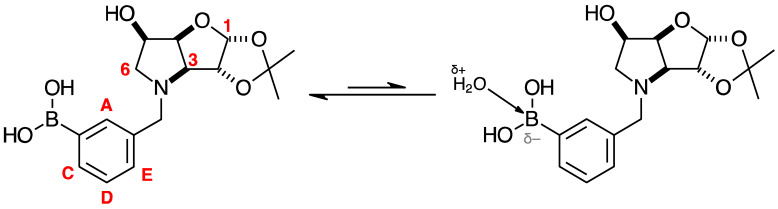 **meta 5** (MeOD)	28.6	18.6	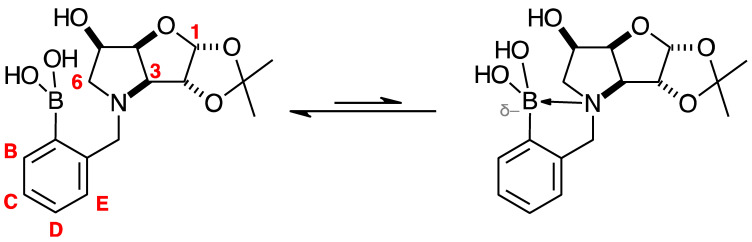 **ortho 5** (D_2_O)	29.5	19.3	
				8.6	1.0		4.5	1.0	
				sharp	sharp		broad	sharp	
				Boronic acid (*trig*)	Boronate (partially *tet*)		Boronic acid (*trig*)	Boronate (partially *tet*)	

The signals for trigonal planar B atoms tend to be broad, and those for partially tetrahedral/tetrahedral B atoms are significantly sharper. The broadness of the trigonal planar B atoms introduces a certain degree of error in the estimation of the borarotated composition. It is nonetheless important to estimate these ratios for application purposes.

### 2.3. Observations on NMR Features for Borylated and Non-Borylated Compounds

#### 2.3.1. Signal Broadening in the ^13^C-NMR Spectra

Table 2 and Table 3, data on light blue background with yellow cells indicating broadened signals.

There are signal broadening effects in ^13^C-NMR spectra arising from different factors.

1. The ArC_quat_-B signals in **para 6**, **para 7**, and **para 8** were not discernible. These signals are broadened due to the presence of the quadrupolar B nuclei one bond away.
2. Lactols **4** and **para 7** display several broadened C signals (see highlighted yellow cells in Table 3). Lactols (or cyclic hemi-acetals/ketals) can exhibit signal broadening [58,59,60,61,62] due to dynamic processes, such as:

- ring-opening/-closing tautomerism
- Hydrogen bonding. Intramolecular or intermolecular hydrogen bonding in lactols can also influence relaxation times and contribute to line broadening.
- Conformational exchanges. The cyclic structure of lactols can adopt multiple conformations, causing additional dynamic averaging of carbon signals. These processes cause the carbon nuclei to experience different chemical environments on the NMR timescale (10^2^–10^4^ s^−1^), leading to broadened or averaged signals.

Most of the studies in this area present in the scientific literature relate to ^1^H-NMR signal broadening, with relatively fewer investigations around ^13^C-NMR signal broadening [63]. Here we present a very clear example of lactol ^13^C-NMR signal broadening in aqueous solutions.

**Table 2:** Comparison of selected ^1^H-NMR data for *N*-benzylated 1,4-dideoxy-1,4-imino-hexitols available from the chemical literature and -on light blue background—for benzylated iminosugars **3**–**5**, and borylated iminosugars **para 6**–**para 8**. The NMR spectra were acquired in D_2_O, unless stated otherwise. # Estimated assignment. NA = not available, δ = chemical shifts (ppm), *J* = coupling constant (Hz). Enantiomeric pairs are of the same colour.

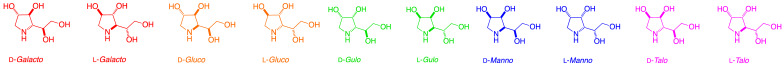										
**Compound:** **1,4-Dideoxy-1,4-Imino-**	**^1^H-NMR Chemical Shifts (δ, ppm), Multiplicity, and Coupling Constants (*J*, Hz) for Nucleus:**									
	**H-1**	**H-1′**	**H-2**	**H-3**	**H-4**	**H-5**	**H-6**	**H-6′**	**ArCH_2_**	**ArCHs**
*N*-benzyl-1,4-dideoxy-1,4-imino-d-allitol # [64]	3.51, dd *J* 12.8 and 4.3	δ 3.28, m	4.42, app-s	δ 4.24, m	δ 3.58, dd *J* 6.3 and 2.5	δ 4.24, m	δ 3.40, m	δ 3.28, m	δ 3.40, m NA	δ 7.37, m (5Hs)
*N*-benzyl-1,4-dideoxy-1,4-imino-d-galactitol (CD_3_OD) [65]	δ 2.86, m	δ 2.71, dd *J*_H-1′,H-1_ 10.7 *J*_H-1′,H-2_ 4.4	δ 3.95–3.84, m	δ 4.09, m	δ 2.91, dd *J*_H-4,H-5_ 4.6 *J*_H-4,H-3_ 2.7	δ 3.95–3.84, m	δ 3.72, dd *J*_H-6,H-6′_ 11.1 *J*_H-6,H-5_ 5.6	δ 3.68, dd *J*_H-6′,H-6_ 11.1 *J*_H-6′,H-5_ 6.2	δ 4.20, d *J* 13.6 **δ** 3.52, d *J* 13.6	δ 7.39–7.20, m
*N*-benzyl-1,4-dideoxy-1,4-imino-d-glucitol.HCl [64]	δ 3.27, d *J*_H-1,H-1′_ 13.2	δ 3.71, dd *J*_H-1′,H-1_ 13.2 *J*_H-1′,H-2_ 4.2	δ 4.31, m	δ 4.16, d	δ 3.60, br s		δ 3.79, m	δ 3.60, br s	δ 4.46, d *J* 13.0 δ 4.31, m	δ 7.37, m
*N*-benzyl-3,6-dideoxy-3,6-imino-1,2-*O*-isopropylidene-α-d-gulofuranose [CDCl_3_] **3**	δ 5.99, d *J*_H-1,H-2_ 3.5	NA	δ 4.51, d *J*_H-2,H-1_ 3.5	δ 3.26, d *J*_H-3,H-4_ 5.6	δ 4.83, t *J*_H-4,H-3/H-5_ 5.8	δ 4.15, app-dddd *J*_H-5,H-6/H-4/OH_ 5.8 *J*_H-5,H-6′_ 2.0	δ 2.93, dd *J*_H-6,H-6′_ 10.8 *J*_H-6,H-5_ 2.1	δ 2.43, dd *J*_H-6′,H-6_ 10.8 *J*_H-6′,H-5_ 5.5	δ 3.95, d *J*_Ha,Hb_ 13.4 δ 3.49, d *J*_Hb,Ha_ 13.4	**δ** 7.36–7.24, m
*N*-benzyl-3,6-dideoxy-3,6-imino-d-gulofuranose **4** *α-fur*	δ 5.51, d *J*_H-1,H-2_ 4.4	NA	δ 4.48–4.43, m, obscured	δ 4.31, dd *J*_H-3,H-4_ 8.2 *J*_H-3,H-2_ 6.1	δ 4.98, d *J* 8.3	δ 4.48–4.43, m obscured	δ 3.66–3.60, m	δ 3.57, dd *J*_H-6′,H-6_ 12.4 *J*_H-6′,H-5_ 3.6	δ 4.57, d *J*_Ha,Hb_ 13.0 δ 4.49, d *J*_Hb,Ha_ 13.0	**δ** 7.60–7.47, m
*N*-benzyl-3,6-dideoxy-3,6-imino-d-gulofuranose **4** *β-fur*	δ 5.36, d *J*_H-1,H-2_ 2.3	NA	δ 4.57–4.53, m obscured	δ 4.23, dd *J*_H-3,H-4_ 7.0 *J*_H-3,H-2_ 2.6	δ 5.00, app-d *J*_H-4,H-3_ 8.2	δ 4.57–4.53, m obscured	δ 3.81, dd *J*_H-6,H-6′_ 12.2 *J*_H-6,H-5_ 4.9	δ 3.62–3.55, m obscured	δ 4.63, d *J*_Ha,Hb_ 12.9 δ 4.40, d *J*_Hb,Ha_ 12.9	**δ** 7.60–7.47, m
*N*-benzyl-1,4-dideoxy-1,4-imino-l-gulitol **5**	δ 3.59, dd *J*_H-1,H-1′_ 12.0 *J*_H-1,H-2_ 7.0	δ 3.28, dd *J*_H-1′,H-1_ 12.1 *J*_H-1′,H-2_ 9.1	δ 4.55, dddd *J*_H-2,H-1′_ 9.3 *J*_H-2,H-1/H-3_ 6.9 *J* 3.6	δ 4.42, ddd *J*_H-3,H-2_ 7.1 *J*_H-3,H-4_ 4.2 *J* 3.0	δ 3.84, dd *J*_H-4,H-5_ 9.3 *J*_H-4,H-3_ 4.0	δ 4.38, ddd *J*_H-5,H-4_ 8.5 *J*_H-5,H-6′_ 4.7 *J*_H-5,H-6_ 3.2	δ 3.85, dd *J*_H-6,H-6′_ 12.6 *J*_H-6,H-5_ 3.2	δ 3.70, dd *J*_H-6′,H-6_ 12.8 *J*_H-6′,H-5_ 4.8	δ 4.79, obscured. δ 4.29, d *J*_Hb,Ha_ 12.8 Hz	δ 7.61–7.52, m
**para 6** [CDCl_3_] boronic acid	δ 5.99, d *J*_H-1,H-2_ 3.5	NA	δ 4.52, d *J*_H-2,H-1_ 3.5	δ 3.25, d *J*_H-3,H-4_ 5.5	δ 4.80, t *J*_H-4,H-3/H-5_ 5.8	δ 4.13, app-dddd *J*_H-5,H-6/H-4/OH_ 6.0 *J*_H-5,H-6′_ 2.3	δ 2.90, dd *J*_H-6′,H-6_ 10.8 *J*_H-6′,H-5_ 2.3	δ 2.42, dd *J*_H-6,H-6′_ 10.8 *J*_H-6,H-5_ 5.6	δ 3.95, d *J*_Ha,Hb_ 13.6 δ 3.51, d *J*_Hb,Ha_ 13.6	**δ** 7.77, d *J* 7.9 **δ** 7.28, d *J* 7.9
**para 7** *α-fur* boronic acid	δ 5.51, d *J*_H-1,H-2_ 4.3	NA	δ 4.49–4.44, m	δ 4.32, dd *J*_H-3,H-4_ 8.1 *J*_H-3,H-2_ 6.3	δ 4.99, app-d partially obscured *J*_H-4,H-3_ 8.2	δ 4.49–4.44, m	δ 3.65–3.60, m	δ 3.57, dd *J*_H-6′,H-6_ 12.2 *J*_H-6′,H-5_ 3.4	δ 4.59, d *J*_Ha,Hb_ 13.0 δ 4.51, d *J*_Hb,Ha_ 13.0	**δ** 7.87, d *J* 7.9 **δ** 7.59, d *J* 7.9
**para 7** *β-fur* boronic acid	δ 5.36, d *J*_H-1,H-2_ 2.2	NA	δ 4.36–4.29, obscured	δ 4.22, dd *J* 6.9 *J* 2.4	δ 5.00, app-d partially obscured *J* 8.5	δ 4.56–4.53 obscured	δ 3.82, dd *J*_H-6,H-6′_ 12.6 *J*_H-6,H-5_ 5.0	δ 3.62–3.50 partially obscured *J*_H-6′,H-5_ 5.2	δ 4.65, d *J*_Ha,Hb_ 13.0 δ 4.42, d *J*_Hb,Ha_ 13.0	**δ** 7.88, d *J* 7.9 **δ** 7.60, d *J* 7.9
**para 8** boronic acid	δ 3.55, dd *J*_H-1,H-1′_ 12.0 *J*_H-1,H-2_ 6.9	δ 3.25, dd *J*_H-1′,H-1_ 12.2 *J*_H-1′,H-2_ 9.2	δ 4.54–4.50, m	δ 3.79, app-d *J* 3.2	δ 4.40–4.34, m	δ 3.82, app-dd *J*_H-5,H-6_ 8.9 *J*_H-5,H-6′_ 5.1	δ 4.40–4.34, m	δ 3.66, dd *J*_H-6′,H-6_ 12.0 *J*_H-6′,H-5_ 5.2	δ 4.83, obscured. δ 4.27, d *J*_Hb,Ha_ 13.0	δ 7.85, dd ^3^*J*_ArH,ArH_ 8.0 ^5^*J*_ArH,ArH_ 1.6 δ 7.57, dd ^3^*J*_ArH,ArH_ 8.0 ^5^*J*_ArH,ArH_ 1.7
*N*-benzyl-1,4-dideoxy-1,4-imino-d-mannitol.HCl # [66]	δ 3.64, dd *J* 12.0, 7.2	δ 3.38, dd *J* 12.0, 7.2	δ 4.54–4.47, m	δ 3.89–3.78, m	δ 4.54–4.47, m	δ 3.96, q *J* 5.0	δ 3.89–3.78, m, 2H		δ 4.61, d *J*_Ha,Hb_ 13.0 δ 4.36, d *J*_Hb,Ha_ 13.0	δ 7.58–7.51, m
*N*-benzyl-1,4-dideoxy-1,4-imino-l-mannitol [67]	δ 2.83, dd *J*_H-1,H-1′_ 11.4 *J*_H-1,H-2_ 6.6	δ 2.76, dd *J*_H-1′,H-1_ 11.4 *J*_H-1′’,H-2_ 6.6	δ 4.13, dt *J*_H-2,H-1/H-1′_ 6.6 *J*_H-2,H-3_ 4.6	δ 4.34–4.29, m	δ 3.01–2.97, m	δ 3.93, dt *J*_H-5,H-6_ 6.3 *J*_H-5,H-6′_ 3.7	δ 3.79, dd *J*_H-6,H-6′_ 11.8 *J*_H-6,H-5_ 3.7	δ 3.72, dd *J*_H-6′,H-6_ 11.8 *J*_H-6′,H-5_ 6.3	δ 3.89, d *J*_Ha,Hb_ 13.3 δ 3.56, d *J*_Hb,Ha_ 13.3	δ 7.43–7.34, m
*N*-benzyl-1,4-dideoxy-1,4-imino-d-talitol.HCl [68]	δ 3.28, dd *J*_H-1,H-1′_ 12.9 *J*_H-1,H-2_ 3.9	δ 3.20, dd *J*_H-1′,H-1_ 12.9 *J*_H-1′,H-2_ 4.2	δ 4.21, q	δ 4.11, dd *J*_H-3,H-4_ 6.3 *J*_H-3,H-2_ 4.2	δ 3.53, m	δ 3.80, m	δ 3.53, m	δ 3.44, dd *J*_H-6,H-6′_ 12.3 *J*_H-6,H-5_ 4.9	δ 4.26, d *J* 13.3 Other signal obscured by HOD signal	δ 7.30, m

**Table 3:** Comparison of selected ^13^C-NMR data, melting point, and optical rotation data for *N*-benzylated 1,4-dideoxy-1,4-imino-hexitols available from the chemical literature and on a light blue background for benzylated iminosugars **3**–**5** and borylated iminosugars **para 6**–**para 8**. The NMR spectra were acquired in D_2_O, unless stated otherwise. # Estimated assignment. Yellow cells denote broadened signals. NA = not available, δ = chemical shifts (ppm), *J* = coupling constant (Hz).

Compound: 1,4-Dideoxy-1,4-Imino-	^13^C-NMR Chemical Shifts (δ, ppm) (in D_2_O Unless Stated Otherwise) for Nucleus:										Melting Points (°C)	Optical Rotation	
	C-1	C-2	C-3	C-4	C-5	C-6	ArCH_2_	ArC_quat_1	ArC_quat_-B)	ArCHs		Temp (°C)	[α]_D_ (°)
*N*-benzyl-1,4-dideoxy-1,4-imino-d-allitol.HCl # [64]	δ 58.4, t	δ 70.7, d	δ 71.4, d	δ 70.3, d	δ 69.1, d	δ 63.0, t	δ 62.4, t	δ 130.3	NA	δ 130.6 δ 131.5 δ 131.9	NA	20	+23.1 (c 0.72, H_2_O)
*N*-benzyl-1,4-dideoxy-1,4-imino-l-allitol [69]	NA	NA	NA	NA	NA	NA	NA	NA	NA	NA	110–111	20	−25.5 (c 1.07, H_2_O)
*N*-benzyl-1,4-dideoxy-1,4-imino-d-galactitol (CD_3_OD) [65]	δ 58.9	75.7	79.2	73.3	71.2	δ 63.7	δ 60.7	δ 138.6	NA	δ 128.4 δ 127.9 δ 126.7	133–135	NA	−25.5 (c 1.0, CHCl_3_)
*N*-benzyl-1,4-dideoxy-1,4-imino-d-glucitol.HCl # [64]	δ 59.9, t	74.8, d	76.9, d	70.0, d	68.8, d	63.6, t	61.4, t	Not reported	NA	δ 130.5 δ 131.4 δ 131.9	NA	20	−31.9 (c 0.68, H_2_O)
*N*-benzyl-3,6-dideoxy-3,6-imino-1,2-*O*-isopropylidene-α-d-gulofuranose [CDCl_3_] **3**	δ 107.6	84.1	72.6	83.1	69.6	61.3	58.1	137.7	NA	δ 128.9 δ 128.5 δ 127.4	NA	22	–14.0 (c 1.0 in CHCl_3_)
*N*-benzyl-3,6-dideoxy-3,6-imino-d-gulofuranose **4** *α-fur*	δ 99.0 (31 Hz)	72.6	72.2	78.8 (32 Hz)	67.4	60.5–58.8 (171 Hz)	60.0–58.2 (177 Hz)	142.4	NA	δ 133.1 δ 131.1 δ 129.3	NA	25	0.06 (c 0.005, MeOH)
*N*-benzyl-3,6-dideoxy-3,6-imino-d-gulofuranose **4** *β-fur*	103.2	68.6 (21 Hz)	72.8	80.7	67.5	57.5	59.8–58.8 (102 Hz)	139.4	NA	130.8 130.4 129.5			
*N*-benzyl-1,4-dideoxy-1,4-imino-l-gulitol **5**	53.1	68.9	70.2	70.1	68.6	63.1	61.6	Not discernible	NA	131.0 130.2 129.3	NA	25	−0.04 (c 0.08, MeOH) [herein]
**para 6** [CDCl_3_] boronic acid	107.6	84.0	72.5	83.1	69.6	61.2	58.1	140.8	not discernible	135.0 128.2	NA	25	0.13 (c 0.16 in MeOH)
**para 7** *α-fur* boronic acid	99.0 (46 Hz)	72.6 (38 Hz)	72.3 (18 Hz)	78.8 (56 Hz)	67.5	60.5–58.8 (173 Hz)	58.8–58.1 (76 Hz).	143.0	not discernible	134.3 130.5	NA	19	+0.15 (c 0.047, MeOH)
**para 7** *β-fur* boronic acid	103.2 (28 Hz)	72.3 (18 Hz)	72.9 (19 Hz)	80.7	67.4	60.5–58.8 (173 Hz)		Not discernible	not discernible	134.5 130.2			
**para 8** boronic acid	53.2	68.9	70.3	70.3	63.1		61.5	134.5	not discernible	134.3 130.4	NA	19	−0.03 (c 0.04, H_2_O)
*N*-benzyl-1,4-dideoxy-1,4-imino-d-mannitol.HCl [66]	55.2	68.4	70.9	67.7	68.5	62.6	58.3	131.0	NA	130.4 129.4 129.0	NA	26	−25.2 (c 0.27, CH_3_OH)
*N*-benzyl-1,4-dideoxy-1,4-imino-l-mannitol [67]	55.8	70.0	72.8	66.3	71.2	63.7	59.7	137.1	NA	130.3 129.0 128.3	108–109	21	+37.7 (c 1.20, H_2_O)
*N*-benzyl-1,4-dideoxy-1,4-imino-d-talitol.HCl # [68]	56.0, t	70.7	73.9	70.6	73.4	64.5, t	63.6, t	130.9	NA	130.4 131.3 132.1	NA	20	−10.1 (c 0.94, H_2_O)

#### 2.3.2. Electronic Effects of B on the Aromatic Ring 

Table 2 and Table 3, data on light blue background.

Boron ester and boronic acid groups (trigonal planar geometry) on aromatic ring are slightly electron-withdrawing, due to B’s Lewis acidity, not its electronegativity, as it is less electronegative than the C atom. This can be seen when comparing aromatic chemical shifts in the ^1^H-NMR and ^13^C-NMR spectra.

##### Comparison of Compounds **3** and **para 6**

In the ^13^C-NMR spectrum: the aromatic carbons are located at 128.9, 128.5, 127.4 (ArCHs) and 137.7 ppm (ArC_quat_) in **3**, and at 135.0, 128.2 (ArCHs, respectively in *ortho* and *meta* positions to the boron ester group) and 140.8 ppm in **para 6**.

It is possible to see a deshielding effect in the *ortho* and *para* positions to the boron ester group. The signals in the sugar region are unaffected.

In the ^1^H-NMR spectrum, the aromatic hydrogens are at 7.36–7.24 ppm in **3** and at 7.77 and 7.28 ppm in **para 6**. The deshielding is pronounced (~0.45 ppm) for the two hydrogens in *ortho* positions with respect to the boron ester group. The signals in the sugar region are unaffected.

##### Comparison of Compounds **4** and **para 7**

In the ***α-fur* anomer,** the deshielding effect seems abrogated in the ^13^C-NMR spectrum, where the aromatic carbons are found at 133.1, 131.1, 129.3 (ArCHs), and 142.4 ppm (ArC_quat_) in **4** and at 134.3, 130.5 (ArCHs, respectively, in *ortho* and *meta* positions to the boronic acid group), and 143.0 ppm in **para 7**. The signals in the sugar region are unaffected.

In the ^1^H-NMR spectrum, the aromatic hydrogens are found at 7.60–7.47 ppm in **4**, and at 7.87 and 7.59 ppm in **para 7**. The deshielding is less pronounced (~0.28 ppm) for the two hydrogens in *ortho* positions with respect to the boronic acid group. The signals in the sugar region are unaffected.

In the ***β-fur* anomer,** a slight deshielding effect is visible in the ^13^C-NMR spectrum: the aromatic carbons are located at 130.8, 130.4, 129.5 (ArCHs), and 139.4 ppm (ArC_quat_) in **4** and at 134.5, 130.2 (ArCHs, respectively in *ortho* and *meta* positions to the boronic acid group), and not discernible in **para 7**. The signals in the sugar region are unaffected, apart from a ~4 ppm difference for C-2s.

In the ^1^H-NMR spectrum, the aromatic hydrogens are at 7.60–7.47 ppm in **4** and at 7.88 and 7.60 ppm in **para 7**. The deshielding is ~0.30 ppm for the two hydrogens in *ortho* positions with respect to the boronic acid group. The signals in the sugar region are unaffected, apart from a ~0.2 ppm difference for H-2s.

##### Comparison of Compounds **5** and **para 8**

In the ^13^C-NMR spectrum, a slight deshielding effect is detected in **para 8** compared to **5**, where the aromatic carbons are located at 131.0, 130.2, and 129.3 (ArCHs) in **5** and at 134.3 and 130.4 (ArCHs), respectively, in *ortho* and *meta* positions to the boronic acid group, and 134.5 ppm in **para 8**. The signals in the sugar region are unaffected.

In the ^1^H-NMR spectrum, the aromatic hydrogens are at 7.61–7.52 ppm in **5**, and at 7.85 and 7.57 ppm in **para 8**. The deshielding is ~0.30 ppm for the two hydrogens in *ortho* positions with respect to the boronic acid group. The signals in the sugar region are unaffected.

### 2.4. ^1^H-, ^13^C- and 2D NMR Data Analysis

A suite of 1D- and 2D-NMR spectra was collected for detailed characterisation of intermediates **3**, **4**, **para 6**, **para 7**, and target compounds **5** and **para 8**. The ones we deem highlight particularly important structural aspects are discussed in the sections below, with spectra available in the Supplementary Information.

NMR analysis of borylated compounds **para 6**, **para 7,** and **para 8** revealed the appearance of more than one signal in the ^11^B-NMR spectra (Figure S8 ^11^B-NMR, Figure S9 ^11^B-NMR, Figure S13 ^11^B-NMR, Table 1). In the literature, ^11^B-NMR investigations of equilibria [70,71,72,73] are very limited. This research and our previous work [47,48,49,50,51,52] provide significant insights into intramolecular (with nucleophilic atoms) and intermolecular (e.g., with solvent molecules) interactions of B atoms in high Fsp^3^ and low Fsp^3^ index drug leads.

**^11^B-NMR of trigonal planar boronate/boronic acid species.** These (whether aliphatic or aromatic) have been found in the range 90–15 ppm, depending on the substitution pattern [74]. For boronate esters and boronic acids, this range is 31–18 ppm [74,75]. The aromatic trigonal planar boronate esters (e.g., protected by the pinacol) are generally found as a sharp peak at ≈32–30 ppm, as observed in **11**–**15** (Table S1) [48,49]. In reference compounds **9** (*ortho*, *meta*, *para*), this boronate ester is also observed at 31 ppm, in line with literature data [76]. Compound **10** decomposed rapidly in D_2_O, whereas boronic acids **11** were found slightly upfield at 30–29 ppm.

**^11^B-NMR of tetrahedral boronate species.** These include diethanolamine derivatives and are found in the range 10 and −10 ppm [77,78,79,80].

High Fsp^3^ index compounds such as **16** and **17** (Table S1) analysed at borarotation and mutarotation equilibria or at the beginning of such processes show complex equilibria established between tetrahedral (major) and trigonal planar species for **16** and mostly trigonal planar for **17** [47]. The novel species **para 6**–**8** provide further insight to enrich the body of equilibrium data relating to the high Fsp^3^ borylated system and what to expect during these investigations.

Comparisons for all compounds in Table 1 are discussed in [51].

#### 2.4.1. NMR Analysis of *N*-Benzyl-3,6-dideoxy-3,6-imino-d-gulofuranose **4** (Figures S2–S5)

The NMR spectra show two major species, the *α-fur* and the *β-fur* anomers, in a 1.0:0.4 ratio. It is also possible to see the open chain form (in a 0.0016 ratio to the *α-fur*) via the aldehyde hydrogen visible at 9.63 ppm. Other signals belonging to minor species likely arising from anomeric equilibria are not analysed here; however, they are visible in the spectra in the Supporting Information.

**Aromatic region**: This region is characterised by the presence of one multiplet, 7.70–7.57 ppm, corresponding to 10 hydrogen atoms, five belonging to the *α-fur* anomer and five to the *β-fur* anomer. COSY correlations between aromatic protons are not discernible, as they are too close on the chemical shift scale. HSQC correlations occur from the aromatic multiplet to carbon signals at 133.1, 131.1, 129.3 ppm (*α-fur*) and to 130.8, 130.5, 129.5 ppm (*β-fur*). The quaternary aromatic carbons are at 142.4 ppm (*α-fur*) and 139.4 ppm (*β-fur*).

**Sugar region**: This region has overlapping signals, with assignments made using the correlations in 2D spectra, and comparison to analogous molecules if the NMR data were not sufficient.

The ***α-fur* anomer** was assigned from the *J* coupling between H-1 and H-2 (4.4 Hz), indicating a Karplus angle in the range of 50° between these atoms and a relative *cis* arrangement. H-1 is a highly deshielded atom at 5.61 ppm, a doublet. This signal is COSY-correlated to an obscured signal in the 4.58–4.53 ppm range, which indicates the presence of H-2. H-2 has a COSY correlation to a signal at 4.41 ppm, assigned as H-3. H-3 is a dd with COSY correlations to a partially obscured signal at 5.08 ppm. The *J* coupling between H-3 and H-4 is 8.3 Hz. H-4 is obscured by *β-fur* H-4, an apparent doublet at 5.10 ppm. The integrations for these are not reliable to determine the assignment of the signal to one of the anomers, as the degree of overlap significantly impacts the assignment process. H-4 correlates to an obscured signal located at 4.58–4.53 ppm, which is assigned as H-5. The H-5 signal is correlated to a multiplet at 3.76–3.70 ppm and a doublet of doublets (dd) at 3.67 ppm, respectively assigned to H-6′ and H-6. The doublet at 4.67 ppm is COSY correlated to the doublet at 4.59 ppm, with a *J* coupling value of 13.0 Hz, which corresponds to ArC*H^a^*H^b^ and ArCH^a^*H^b^*.

**2D-NMR.** HSQC Correlations are as follows: H-1 at 5.61 ppm correlates to a slightly broadened signal at 99.0 ppm (width 31 Hz), H-2 at 4.58–4.53 ppm to 72.6 ppm, H-3 at 4.41 ppm to 72.2 ppm, H-4 at 5.08 ppm to a slightly broadened signal at 78.8 ppm (width 32 Hz), H-5 at 4.58–4.53 ppm to 67.4 ppm, H-6 and H-6′ at 3.76–3.70 and 3.67 ppm to a broad signal at 60.5–58.8 ppm (width 171 Hz), ArC*H^a^*H^b^ at 4.67 ppm and ArCH^a^*H^b^* at 4.59 ppm to a broad signal at 60.0–58.2 ppm (width 177 Hz).

The ***β-fur* anomer** was assigned starting from the H-1, which appears as a doublet at 5.46 ppm. H-1 does not COSY correlate to any other signal. Locating H-2 was somewhat arduous and was achieved by exclusion, once all other signals had been assigned. At 5.10 ppm, the H-4 appears as a partially obscured apparent doublet. This signal is COSY correlated to two signals: one at 4.67–4.63 ppm and one centred at 4.33 ppm. The former is obscured due to overlap with ArC*H^a^*H^b^ (*α-fur*) and is assigned to H-5 as a multiplet. The signal at 4.33 ppm is assigned to H-3. The H-5 signal is COSY correlated to two signals that are correlated to each other, namely the H-6 and H-6′. H-6 appears as a dd at 3.91 ppm, whereas H-6′ is an obscured signal (presumably a dd as well) in the range 3.72–3.65 ppm. H-2 is obscured by ArC*H^a^*H^b^ (*α-fur*) and appears in the range 4.67–4.63 ppm based on HSQC correlations. ArC*H^a^*H^b^ and ArCH^a^*H^b^* are at 4.73 and 4.50 ppm, respectively, as two COSY correlated doublets.

**2D-NMR.** HSQC Correlations are as follows: H-1 at 5.46 ppm correlates to a carbon at 103.2 ppm, H-2 and H-5 at 4.67–4.63 ppm to a slightly broadened carbon signal at 68.6 ppm (C-2, width 21 Hz) and to 67.5 ppm (C-5). H-3 at 4.33 ppm to 72.8 ppm (C-3), H-4 at 5.10 ppm to a carbon at 80.7 ppm. H-6 and H-6′ at 3.91 and 3.72–3.65 ppm, respectively, correlate to a carbon signal at 57.5 ppm (C-6). ArC*H^a^*H^b^ and ArCH^a^*H^b^* at 4.73 and 4.50 ppm correlate to a broad carbon signal at 59.8–58.8 ppm (width 102 Hz), where both carbon atoms are found.

#### 2.4.2. NMR Analysis of *N*-Benzyl-1,4-dideoxy-1,4-imino-l-gulitol **5** (Figures S6 and S7)

Some highlights from the ^1^H-NMR spectrum of intermediate **5** show H-2 as a doublet of doublets of doublets of doublets (dddd) with COSY correlations to both H-1 and H-1′, H-3, and presumably OH-2. H-3 and H-5 are both doublets of doublets of doublets (ddd), partly overlapping with each other. H-6 and H-4 are another set of two overlapping dd_s_.

#### 2.4.3. NMR Analysis of *N*-(4-Methylphenylboronic acid pinacol ester)-3,6-dideoxy-3,6-imino-1,2-*O*-isopropylidene-α-d-gulofuranose **para 6** (Figure S8, Scheme 2, Table 4)

The elucidation of this structure was carried out as usual via 1D and 2D NMR experiments. It is important to highlight one important aspect described below.

The ^1^H-NMR spectrum of **para 6** shows one predominant structure in ~10:1.5 integration intensity to the most populated minor species. Then, a few more minor species are visible clearly in the aromatic region. Minor signals are also visible in the remainder of the spectrum. However, the aromatic region is particularly helpful in this case as two doublets are expected for **para 6** (7.77 and 7.26 ppm), which are seen, along with a series of other doublets. This implies that species containing a *para*-substituted aromatic group are present. In a first instance, one may instinctively think these minor signals derive from impurities. However, analysis of the ^11^B-NMR reveals the presence of two signals: the expected one for the boronate ester (trigonal planar) for the B-pinacol group at 30.6 ppm, and a second signal at 22.3 ppm. Two hypotheses are considered.

**Scheme 2:**
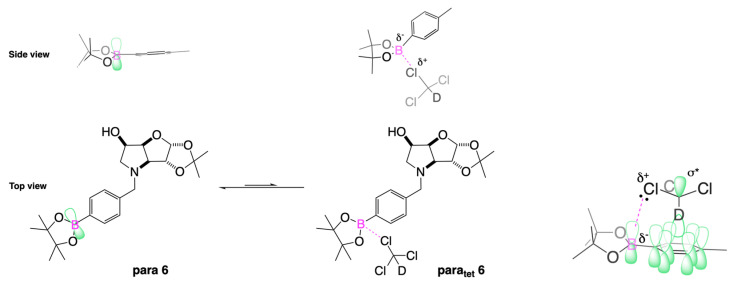
(*top*) Interactions between the empty B *p*-orbital of **para 6** with a molecule of CDCl_3_ as described in hypothesis 1, with side and top views. (*bottom*) Interactions between the aromatic region of para 6 with a molecule of CDCl_3_, as described in hypothesis 2.

1. The presence of two signals points to an equilibrium between the trigonal planar B atom and several slightly quaternised B species visible on the NMR timescale. The integration between these two signals is ~3.7:1.0. This is reflected in the ^1^H-NMR, where the sum of integrations of the minor species mirrors this ratio. The hypothesis is that the pinacol B atom may be forming a partial dative bond with the Cl atom of the NMR solvent in such a way as to flick back and forth between the trigonal planar **para 6** and a series of slightly quaternised species **para_tet_ 6** (Scheme 2). This phenomenon was also observed in another project in our research laboratory, where a slight pink tinge appeared upon dissolving a structurally related molecule in CDCl_3_.
2. The signal at 22 ppm could arise from an interaction between the aromatic π-system of **para 6** and the antibonding C-D σ-bond of a solvent molecule (Scheme 2). To our knowledge, this phenomenon has not been reported in the literature for borylated systems, and any resultant effects on the ^11^B-NMR are also yet to be reported. What has been reported in the literature is that aromatic rings can act as donors to result in electron density transfer to small molecules such as chloroform [81,82]. This interaction would shift the location of the boron signal in the ^11^B-NMR. However, it would be expected that this interaction would have a deshielding effect on the B and to find another signal downfield from 30.2 ppm. There is potentially another phenomenon coupled to this that shifts electron density back onto the B atom. The proximity of one of the Cl atoms to the empty *p*-orbital of boron may be in the correct orientation and distance for a lone pair to complex into that empty *p*-orbital to make the B atom more d^-^ and the Cl atom involved more δ^+^.

This way there would be a circular shift of electron density from the π-system of the aromatic ring to the σ* orbital of the C-D bond, from the D to the Cl atoms via the s-system, from the Cl to the B via the lone pair/empty *p*-orbital Lewis base/acid interaction, from the B to the aromatic ring via the σ-system.

When MeOD is the NMR solvent, it is possible to see two signals at 32.0 and 18.0 ppm in integration ratios of 1.00:0.06. The signal at 18.0 ppm is ascribed to the **para_tet_ 6** species, where the CDCl_3_ solvent molecule interacting with the B has been replaced by a MeOD molecule complexing to the B empty *p*-orbital via a lone pair on the O atom. Upon acidification with HCl to pH 1, the signals appeared at 29.8 and 19.3 ppm in an integration ratio of 1.00:0.12.

#### 2.4.4. NMR Analysis of *N*-(4-Methylphenyl boronic acid)-3,6-dideoxy-3,6-imino-d-gulofuranose **para 7** (Figures S9–S12)

This intermediate is an equilibrium mixture of two predominant conformations, the *α-fur* and the *β-fur* anomers, with the boron in trigonal planar geometry in its boronic acid form (^11^B-NMR signal at 28.7 ppm). Minor signals can be seen in the ^1^H-NMR, likely arising from the same anomers where the boron is in the boronate form (^11^B-NMR signal at 19.4 ppm) (Figure S9). The anomeric ratio for the α-*fur*:*β-fur* is 1.0:0.4. Only the boronic acid data for the *α-fur* and *β-fur* anomers are reported here. The minor species are visible in the spectra provided in the Supporting Information.

**Aromatic region**: This region is characterised by the presence of the two doublets, each corresponding to two hydrogen atoms. The doublets belonging to the *α-fur* anomer partially obscure the two doublets belonging to the *β-fur* anomer. Further to these, there are also other doublets whose integrations are significantly smaller, potentially belonging to the same aromatic atoms for the boronate species (for which there is evidence in the ^11^B-NMR spectrum, Figure S9).

The *α-fur* anomer displays COSY correlations between the two doublets. HSQC correlations occur from the doublets at 7.87 and 7.59 ppm to carbon signals at 134.3 and 130.5 ppm, respectively. The aromatic C directly bonded to the B atom is not discernible. The other quaternary aromatic carbon is at 143.0 ppm.

The COSY correlations between the two doublets of the *β-fur* anomer are obscured by the correlations for the *α-fur* anomer. HSQC correlations occur from partially obscured doublets at 7.88 and 7.60 ppm to carbon signals at 134.5 and 130.2 ppm, respectively. The aromatic C directly bonded to the B atom is not discernible. The other quaternary aromatic carbon is at 150.2 ppm.

**Sugar region**: This region has many overlapping signals, with assignments made using the correlations in 2D spectra (HSQC, HMBC, and COSY), and comparison to analogous molecules if the NMR data were not sufficient.

The *α-fur* anomer was assigned based on the *J* coupling between H-1 and H-2 (4.3 Hz), which indicates a Karplus angle in the range of 50° between these atoms and points to a relative *cis* arrangement. H-1 is a highly deshielded atom at 5.51 ppm as a doublet. This signal has a COSY correlation to a multiplet in the range 4.49–4.44 ppm, which indicates that H-2 is present. H-2 has a COSY correlation to a signal at 4.32 ppm, which is assigned as H-3. H-3 is a dd with COSY correlations to a partially obscured signal at 4.99 ppm. The *J* coupling between H-3 and H-4 is 8.2 Hz. H-4 is obscured by *β-fur* H-4, app-d at 5.00 ppm. The integrations for these are not reliable to determine the assignment of the signal to one of the anomers, as the degree of overlap significantly impacts the assignment process. H-4 correlates to a signal located at 4.49–4.44 ppm, which is assigned as H-5. The H-5 signal is correlated to a multiplet at 3.65–3.60 ppm and a dd at 3.57 ppm (H-6 and H-6′). The doublet at 4.59 ppm is COSY correlated to the doublet at 4.51 ppm, with a *J* coupling value of 13.0 Hz, which corresponds to ArC*H^a^*H^b^ and ArCH^a^*H^b^*.

**2D-NMR.** HSQC Correlations are as follows: H-1 at 5.51 ppm correlates to a broad signal at 99.0 ppm (width 46 Hz), H-2 at 4.49–4.44 ppm to 72.6 ppm as a slightly broadened signal (width 38 Hz), H-3 at 4.32 ppm to 72.3 ppm as a slightly broadened signal (width 18 Hz), H-4 at 4.99 ppm to a broad signal at 78.8 ppm (width 56 Hz), H-5 at 4.49–4.44 ppm to 67.5 ppm, H-6 and H-6′ at 3.65–3.60 and 3.57 ppm to a broad signal at 60.5–58.8 ppm (width 173 Hz), ArC*H^a^*H^b^ at 4.59 ppm and ArCH^a^*H^b^* at 4.51 ppm to a broad signal at 58.8–58.1 ppm (width 76 Hz).

The *β-fur* anomer was assigned starting from H-1, which appears as a doublet at 5.36 ppm. H-1 does not COSY-correlate to any other signal. This made locating H-2 somewhat arduous and was achieved by exclusion, once all other signals had been assigned. At 5.00 ppm, the H-4 appears as a partially obscured app doublet. This signal is COSY-correlated to two signals: one at 4.56–4.53 ppm and one centred at 4.22 ppm. The former is obscured due to overlapping with ArC*H^a^*H^b^ and ArCH^a^*H^b^* and assigned as H-5 (a multiplet). The signal at 4.22 ppm is assigned to H-3. The H-5 signal is COSY-correlated to two signals that are correlated to each other, namely the H-6 and H-6′. H-6 appears as a dd at 3.82 ppm, whereas H-6′ is a partially obscured signal (presumably a dd as well) in the range 3.55–3.47 ppm, where only the minor *J* coupling value could be discerned (5.2 Hz). H-2 is obscured by *α-fur* H-3 and appeared in the range 4.36–4.29 ppm based on HSQC correlations. ArC*H^a^*H^b^ and ArCH^a^*H^b^* are at 4.65 and 4.42 ppm, respectively, as two COSY-correlated doublets.

**2D-NMR.** HSQC Correlations are: H-1 at 5.36 ppm correlates to a slightly broadened carbon at 103.2 ppm (width 28 Hz, C-1), H-2 at 4.36–4.29 ppm to a slightly broadened carbon signal at 72.3 ppm (width 18 Hz, C-2), H-3 at 4.22 ppm to a signal at 72.9 ppm (width 19 Hz, C-3), H-4 at 5.00 ppm to a carbon at 80.7 ppm, H-5 at 4.56–4.53 ppm to 67.4 ppm. H-6, H-6′, ArC*H^a^*H^b^, and ArCH^a^*H^b^* at 3.82, 3.55–3.47, 4.65, and 4.42 ppm correlate to a broad carbon signal at 60.5–58.8 ppm (width 173 Hz), where both carbon atoms are found.

#### 2.4.5. NMR Analysis of N-(4-Methylphenyl boronic acid)-1,4-dideoxy-1,4-imino-l-gulitol **para 8** (Figures S13–S16)

Target molecule **para 8** is present in both forms, the boronic acid and boronate anion, deriving from complexation with a water molecule. This boronate anion seems quite stable once it has formed. It is unlikely that the B atom is forming an intramolecular bond to one of the OH groups of the iminosugar ring, as they are too far away from each other. However, depending on concentration, at higher concentrations, it should be possible for intermolecular bonds between the B atom of one **para 8** with a nucleophilic atom of another **para 8** molecule.

**Aromatic region.** Analysis of the ^1^H-NMR spectrum shows a wider number of signals in this region than in the sugar region. This points to a chemical change occurring on the aromatic ring, namely the equilibria between (potentially several) boronic acid species and one (or more) boronate anion species. The signals in the orange boxes belong to the predominant boronic acid species, and the signals in the green boxes belong to several minor species (boronic acid and boronate anion species). In the aromatic region, the two main signals appear at 7.85 and 7.57 ppm as two dd (in orange boxes), COSY correlated to each other, and represent the four aromatic H nuclei of the predominant boronic acid species **para 8**. Other doublets and dd are visible, hypothesised to correspond to boronate anion species coordinated to a water molecule and other boronic acid species very weakly coordinating to a water molecule.

**Sugar region.** This region is quite crowded, namely showing more signals than expected, especially between 5.00 and 4.20 ppm. The two anomeric H-1s of **para 7** are not visible anymore at 5.51 and 5.36 ppm. There is a signal at 4.83 ppm (obscured by the D_2_O signal) COSY-correlated to a doublet at 4.27 ppm. These have been assigned as ArCH^a^*H^b^* and ArC*H^a^*H^b^, respectively. The dd at 3.55 ppm is COSY correlated to the dd at 3.25 ppm. These were designated as H-1 and H-1′. Both of them are COSY-correlated to a multiplet at 4.54–4.50 ppm, which was assigned as H-2. No other correlation was discernible for H-2.

The doublet at 3.79 ppm was designated as H-3. At 4.40–4.34 ppm, there is a multiplet integrating to two H nuclei with correlations to signals at 3.82 and 3.66 ppm. These signals were assigned as H-6 and H-4. The dd at 3.82 ppm correlates to 4.40–4.34 and 3.66 ppm. It was assigned as H-5. The last signal, located at 3.66 ppm, was assigned as H-6′. The coupling constants are as follows: ArC*H^a^*H^b^ to ArCH^a^*H^b^* is 13.0 Hz, H-1 to H-1′ is 12.0 Hz, H-1 to H-2 is 6.9 Hz, H-1′ to H-2 is 9.2 Hz, H-5 to H-6 is 8.9 Hz, H-5 to H-6′ is 5.1 Hz, and H-6′ to H-6 is 12.0 Hz. H-3 has a *J* value of 3.2 Hz to another H nucleus. It is unclear whether this correlation is to H-2 or H-4, as both of these are multiplets.

**2D-NMR.** HMBC correlates the signal at 134.3 ppm to the ArCH signals, pointing to 134.3 ppm being the ArC_quat_. No further discernible HMBC correlation was possible. HSQC correlations for the sugar region indicate the ^1^H-NMR signals at 3.55 and 3.25 ppm correlated to one C signal at 53.2 ppm, which was designated as C-1. The ArC*H^a^*H^b^-to-ArCH^a^*H^b^* signals are correlated to one carbon at 61.5 ppm, which was assigned as the ArCH_2_.

The ArC_quat_ directly bonded to the B atom was not discernible due to the quadrupolar broadening transmitted to it. The H-2 signal at 4.54–4.50 ppm is correlated to a carbon at 68.9 ppm, which was assigned as C-2. The multiplet at 4.40–4.34 ppm correlated to two carbon signals, one at 68.6 and 63.1 ppm, which were assigned to C-4 and C-6, respectively. The signals at 3.82 ppm correlated to one C atom at 63.1 ppm, which was designated as C-5. The signal at 3.72 ppm was correlated to a carbon at 70.3 ppm, which was assigned as C-3.

The principal other C nuclei signals visible are located at δ_C_: 134.4, 134.0, 132.2, 126.8, 115.9, and 75.7 ppm. These presumably arise from either boronate anion species and/or boronic acid species.

## 3. Materials and Methods

### 3.1. Biological Assays

#### 3.1.1. Glycosidase Inhibition

In the literature, glycosidase inhibition data are available for the following *N*-benzyl-1,4-dideoxy-1,4-imino derivatives (Table 5):

- *N*-Benzyl-1,4-dideoxy-1,4-imino-d-allitol [69]. The parent iminosugar 1,4-dideoxy-1,4-imino-l-allitol has a relatively broad selectivity in glycosidase inhibition. However, a narrower selectivity is observed upon *N*-benzylation from predominantly an inhibitor of α-d-mannosidase to one that selectively inhibits α-l-fucosidase (76%), with weak/no inhibitions for α-d-mannosidase (Golgi II, 11% and lysosomal acidic, 7%), β-d-mannosidase (0%) and β-d-*N*-acetylhexosaminidase (35%).
- *N*-Benzyl-1,4-dideoxy-1,4-imino-d-galactitol [65]. Aldose reductase is recognised as an important checkpoint of the main biochemical abnormalities affecting diabetic tissues. When screened against aldose reductase and α-d-glucosidase, this iminosugar displays an inhibition of 32.6% and 93.2% respectively. A marked increase in efficacy was detected through the addition of the benzyl group compared to the parent iminosugar. The IC_50_ was found to be 40.6 μM towards α-d-glucosidase.
- *N*-Benzyl-1,4-dideoxy-1,4-imino-d-mannitol.HCl. This is by far the most studied in its inhibition against glycosidases [66,83,84]. *N*-Aralkylation with short alkyl chains delivers mostly inactivity [83]. However, inhibition towards β-d-glucosidase, β-d-galactosidase, and β-d-glucuronidase increases as the *N*-alkyl chain is lengthened [66]. In particular, for *N*-Benzyl-1,4-dideoxy-1,4-imino-d-mannitol.HCl, the following inhibitions are observed: α-d-glucosidase (rice) (34.2%), α-d-glucosidase (rat intestinal maltase) (16.6%), α-d-glucosidase (yeast) (6.5%), β-d-glucosidase (almond) (6.1%), β-d-glucosidase (bovine liver) (9.5%), β-d-glucosidase (human lysosome) (4.7%), α-d-galactosidase (coffee bean) (8.8%), β-d-galactosidase (bovine liver) (7.9%), α-d-mannosidase (Jack bean) (27%), β-d-mannosidase (snail) (4.4%), α-l-fucosidase (bovine kidney) (8.4%), trehalase (porcine kidney) (0%), β-d-glucuronidase (*E. coli*) (9.8%), α-l-rhamnosidase (*P. decumbens*) (11.5%), amyloglucosidase (*A. niger*) (11.4%) [66]. Screening against α-d-mannosidases shows significant inhibition towards lysosomal acidic (34%), neutral (44%), and Golgi II (72%) [83]. Screening against α-d-mannosidases: fruit fly lysosomal acidic shows an IC_50_ = 1.5 × 10^−3^ M and fruit fly Golgi II an IC_50_ = 6.9 × 10^−4^ M [84].
- *N*-Benzyl-1,4-dideoxy-1,4-imino-d-talitol.HCl [85]. This iminosugar was screened against β-d-galactosidase (*E. coli*), α-d-galactosidase (coffee bean), and α-d-mannosidase (Jack bean), displaying little inhibition. *N*-Arylation with a borylated benzyl resulted in a significant increase in inhibition of β-d-galactosidase (*E. coli*) (44–55%), and less pronounced increases in inhibition of α-d-galactosidase (coffee bean) (<5%), α-d-mannosidase (Jack bean) (10%) [85].

HIV replication inhibition by *N*-benzyl-1,4-dideoxy-1,4-iminosugar derivatives is also reported in [45].

##### Glycosidase Inhibitions (Table 6)

*Controls*: the controls used are borocaptate sodium (BSH), 4-borono-l-phenylalanine (BPA), and their ^10^B-enriched analogues ^10^B-BSH and ^10^B-BPA. BSH and BPA are clinically used for BNCT. To our knowledge, these drugs have only been reported in a glycosidase assay in these publications [47,48]. Under the conditions screened, none of them significantly inhibits the glycosidases in the panel at 100 or 1000 μM. In the panel, percent inhibitions range from a minimum value of 0 to a maximum value of 19.6.

*Glycosidase Inhibitions*: Intermediate **para 6** and target compound **5** do not provide a strong degree of inhibition of the glycosidase enzymes. However, selective inhibitions (>30%) emerge for β-glycosidases, two glucosidases, and one galactosidase. Specifically, **3** (31.9%) and **para 7** (38.8%) inhibit β-d-glucosidase (bovine liver), and **para 7** (43.6%) additionally inhibits β-d-glucosidase (almond). It is unclear which anomer/s and conformation/s preferentially interact with each enzyme. Rationalisation of inhibition profiles is possible through a systematic postulated framework, such as here [33].

Inhibition of β-d-galactosidase (bovine liver) was stronger and selective for **4**, **para 7,** and **para 8**, with moderate IC_50_ values for **4** (133 μM) and **para 7** (218 μM), and weak for **para 8** (501 μM). β-d-glucosidase (EC 3.2.1.21), and β-d-galactosidase (EC 3.2.1.23) are linked to lysosomal storage disorders, such as Gaucher disease [86,87,88] and GM1 gangliosidosis (β-d-glucosidase), and Morquio syndrome B (β-d-galactosidase) [89].

##### Glycosidase Inhibitions (Table 7)

**Figure 2:**
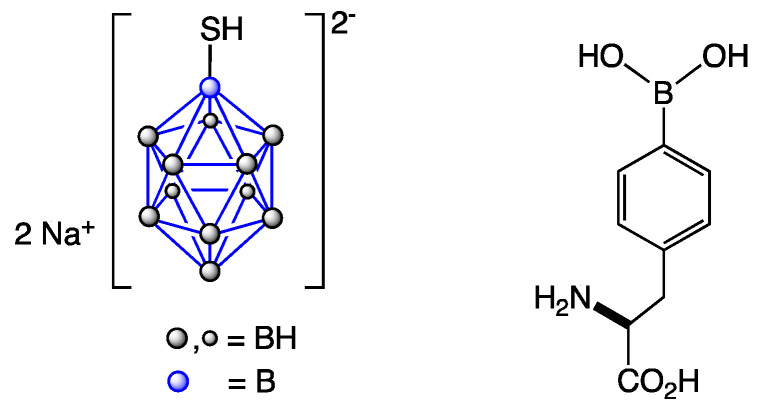
Structures of (left) BSH/^10^BSH, (right) BPA/^10^BPA.

*Controls*: Under the assay conditions, BSH and its ^10^B-BSH (Figure 2) inhibited several glycosidases within the range 31.6–65.9%. BPA and ^10^B-BPA (Figure 2) inhibitions were all below 30%.

*Glycosidase Inhibitions*: The strongest inhibition was seen for **para 7,** which also inhibits β-d-glucosidase (almond) in a significant amount (68.1%, strong inhibition), mirroring the measurement from Laboratory 1. Compound **4** also inhibits the same glycosidase (58.1%), likewise mirroring the corresponding inhibition (27.4%) from Laboratory 1. Both compounds show selective inhibitions. Other appreciable inhibitions were not detected.

#### 3.1.2. Cancer Screening (Table 8)

Two assays were run: the dose screen to ascertain cell growth inhibition in response to 25 µM of drug (on a blue background) and the dose response (GI_50_) (on a green background). For a discussion of BNCT as a radiotherapeutic modality, a rationalisation is provided here [47].

*Controls*: under the conditions screened, none of the controls significantly inhibits cell growth at 25 µM. Cell growth inhibitions range from <0% to 19% in our panel of 10 cancer cell lines (HT29, U87, MCF-7, A2780, H460, A431, Du145, BE2-C, SJ-G2, and MIA-Pa-Ca2) and a normal cell line (MCF10A). In total, 10 entries for the controls were double-digit (≥10%) percent inhibitions, 34 entries displayed inhibition values between 0.01% and 9.99%, and nine entries of ≤0%.

*Cancer Screening*: The strongest cell growth inhibition is observed for **para 7** against the A2780 ovarian carcinoma cell line (27%). Overall, this is a weak/moderate cell growth inhibition. From a structure–activity relationship perspective, it is notable that this particular cancer cell line is inhibited by all compounds, with inhibitions: 11% for **3**, 15% for **4**, 13% for **5**, 14% for **para 6**, 27% for **para 7** and 14% for **para 8**. **para 7** also has the highest inhibitions as we run across the data, with a 15% inhibition against HT29 colon carcinoma and 14% against MIA-Pa-Ca2 pancreatic carcinoma cell lines.

Beyond the inhibition and dose response capabilities, the real therapeutic potential of these borylated drugs lies in their switch-on/switch-off activation under BNCT radiotherapeutic conditions. This is when they become destructive to the cancer molecules, provided they can selectively accumulate in them versus healthy cells. We have demonstrated in earlier work that this is possible [48,90,91].

**Table 8:** Cancer Screening. On blue background DOSE SCREEN: Percentage (%) Cell Growth Inhibition in response to 25 µM of Drug (*The higher the value the greater the growth inhibition*) and inhibition value ranges: 35–59% (green), 10–34% (blue) and 0–9% (black). On a green background, DOSE RESPONSE: GI_50_ = Concentration (µM) that inhibits cell growth by 50% (*The lower the value, the greater the growth inhibition*). In bold are the values of the most potent inhibition. * n = 3–4, otherwise n = 2.

Compound	HT29	U87	MCF-7	A2780	H460	A431	Du145	BE2-C	SJ-G2	MIA-Pa-Ca2	MCF10A
	*Colon* *Carcinoma*	*Glioblastoma*	*Breast* *Carcinoma*	*Ovarian* *Carcinoma*	*Lung* *Carcinoma*	*Skin* *Carcinoma*	*Prostate* *Carcinoma*	*Neuroblastoma*	*Glioblastoma*	*Pancreatic* *Carcinoma*	*Breast* *(Normal)*
**BSH**	* 3 ± 2	* <0	* 15 ± 3	* 2 ± 5	* 8 ± 2	* <0	* 0 ± 8	* 10 ± 6	* 3 ± 8	* 2 ± 6	* 8 ± 3
**^10^B-BSH**	* 5 ± 1	* 0 ± 2	* 5 ± 3	* 5 ± 4	* 4 ± 2	* <0	*7 ± 7	* 8 ± 7	* 1 ± 9	* 2 ± 4	* 13 ± 4
**BPA**	* 14 ± 0	* <0	* <0	* 4 ± 1	* 7 ± 8	* 4 ± 6	* 19 ± 10	* 13 ± 10	* 5 ± 8	* 3 ± 3	* 4 ± 1
**^10^B-BPA**	* 15 ± 4	* <0	* 1 ± 3	* 8 ± 4	* 8 ± 5	* 4 ± 4	* 15 ± 9	* 10 ± 6	* 5 ± 10	* 11 ± 3	* <0
**3**	* 10 ± 0	* 5 ± 4	* 7 ± 2	* 11 ± 3	* 9 ± 3	* 7 ± 4	* <0	* 10 ± 6	* 10 ± 10	* 7 ± 5	* 9 ± 4
**4**	* 11 ± 3	* 8 ± 4	* 9 ± 5	* 15 ± 4	* 8 ± 5	* 11 ± 5	* 0 ± 9	* 12 ± 6	* 12 ± 10	* 8 ± 6	* 14 ± 4
**5**	2 ± 3	NA	NA	13 ± 6	* 0 ± 1	* 2 ± 4	NA	NA	NA	6 ± 1	* 3 ± 2
**5**	>50	NA	NA	>50	>50	>50	NA	NA	NA	>50	>50
**para 6**	* 8 ± 5	* 7 ± 4	* 3 ± 4	* 14 ± 4	* 10 ± 4	* 6 ± 7	* <0	* 5 ± 4	* 9 ± 7	* 9 ± 5	* 14 ± 4
**para 7**	15 ± 3	NA	NA	**27 ± 2**	9 ± 6	9 ± 5	NA	NA	NA	14 ± 0	4 ± 2
**para 7**	>50	NA	NA	>50	>50	>50	NA	NA	NA	>50	>50
**para 8**	5 ± 2	NA	NA	14 ± 7	1 ± 2	0 ± 5	NA	NA	NA	11 ± 1	1 ± 0
**para 8**	>50	NA	NA	>50	>50	>50	NA	NA	NA	>50	>50

### 3.2. Inhibition Experimental

#### 3.2.1. Glycosidase Inhibition Experimental (Table 6)

The enzymes α-d-glucosidase (from yeast), β-d-glucosidases (from almond and bovine liver), α-d-galactosidase (from coffee beans), β-d-galactosidase (from bovine liver), α-d-mannosidase (from Jack bean), β-d-mannosidase (from snail), α-l-rhamnosidase (from *Penicillium decumbens*), α-l-fucosidase (from bovine kidney), trehalase (from porcine kidney), β-d-glucuronidases (from *E. coli* and bovine liver), amyloglucosidase (from *A. niger*), *para*-nitrophenyl glycosides, and various disaccharides were purchased from Sigma-Aldrich Co. (St. Louis, MO, USA).

Brush border membranes were prepared from the rat small intestine according to the method of Kessler et al. [92], and were assayed at pH 6.8 for rat intestinal maltase using maltose. For rat intestinal maltase, porcine kidney trehalase, and *A. niger* amyloglucosidase activities, the reaction mixture contained maltose (25 mM) and the appropriate amount of enzyme, and the incubations were performed for 10–30 min at 37 °C. The reaction was stopped by heating at 100 °C for 3 min. After centrifugation (600× *g*; 10 min), the resulting reaction mixture was added to the Glucose CII-test Wako (Wako Pure Chemical Ind., Osaka, Japan). The absorbance at 505 nm was measured to determine the amount of the released d-glucose. Other glycosidase activities were determined using an appropriate *para*-nitrophenyl glycoside as substrate at the optimum pH of each enzyme. The reaction mixture contained the substrate (2 mM) and the appropriate amount of enzyme. The reaction was stopped by the addition of Na_2_CO_3_ (400 mM). The released *para*-nitrophenol was measured spectrometrically at 400 nm. All reactions run in methanol.

#### 3.2.2. Glycosidase Inhibition Experimental (Table 7)

All enzymes and *para*-nitrophenyl substrates were purchased from Sigma. Enzymes were assayed at 27 °C in citric acid (0.1M)/disodium hydrogen phosphate (0.2 M) buffers at the optimum pH for the enzyme. The incubation mixture consisted of 10 mL enzyme solution, 10 mL of 1 mg/mL aqueous solution of extract, and 50 mL of the appropriate 5 mM *para*-nitrophenyl substrate made up in buffer at the optimum pH for the enzyme. The reactions were stopped by the addition of 70 mL 0.4 M glycine (pH 10.4) during the exponential phase of the reaction, which had been determined at the beginning using uninhibited assays in which water replaced the inhibitor. Final absorbances were read at 405 nm using a Versamax microplate reader (Molecular Devices). Assays were carried out in triplicate, and the values given are the means of the three replicates per assay. All reactions run in water.

#### 3.2.3. Cancer Screening Experimental (Table 8)

All test agents were prepared as stock solutions (20 mM) in dimethyl sulfoxide (DMSO) and stored at −20 °C. Cell lines used in the study included HT29, (colorectal carcinoma); U87, SJ-G2, (glioblastoma); MCF-7, (breast carcinoma); A2780 (ovarian carcinoma); H460 (lung carcinoma); A431 (skin carcinoma); Du145 (prostate carcinoma); BE2-C (neuroblastoma) and MiaPaCa-2 (pancreatic carcinoma); together with the one non-tumour derived normal breast cell line (MCF10A). All cell lines were incubated in a humidified atmosphere 5% CO_2_ at 37 °C. The cancer cell lines were maintained in Dulbecco’s modified Eagle’s medium (DMEM; Sigma, Australia) supplemented with foetal bovine serum (10%), sodium pyruvate (10 mM), penicillin (100 IUmL^−1^), streptomycin (100 µg mL^−1^), and l-glutamine (2 mM).

The non-cancer MCF10A cell line was maintained in DMEM/F12 (1:1) cell culture media, 5% heat inactivated horse serum, supplemented with penicillin (50 IUmL^−1^), streptomycin (50 µg mL^−1^), HEPES (20 mM), l-glutamine (2 mM), epidermal growth factor (20 ng mL^−1^), hydrocortisone (500 ng mL^−1^), cholera toxin (100 ng mL^−1^), and insulin (10 mg mL^−1^).

Growth inhibition was determined by plating cells in duplicate in medium (100 µL) at a density of 2500–4000 cells per well in 96-well plates. On day 0 (24 h after plating), when the cells are in logarithmic growth, medium (100 µL) with or without the test agent was added to each well. After a 72-h drug exposure, growth inhibitory effects were evaluated using the MTT (3-(4,5-dimethyltiazol-2-yl)-2,5-diphenyltetrazolium bromide) assay and absorbance read at 540 nm. The percentage growth inhibition was calculated at a fixed concentration of 25 µM, based on the difference between the optical density values on day 0 and those at the end of drug exposure. Each data point is the mean ± the standard error of the mean (SEM) calculated from three replicates, which were performed on separate occasions and separate cell line passages.

#### 3.2.4. Numbering System

Spectroscopic data for all compounds are assigned based on a numbering system derived from systematic naming of materials according to IUPAC recommendations on carbohydrate nomenclature [93]. The numbering is given in Figure 1 and Table 1 by the red numbers and letters on selected structures.

#### 3.2.5. General Chemical Characterisation Experimental

Spectra were recorded on a Bruker Ascend^TM^ 400 in deuterated solvent as stated. Chemical shifts (δ) are quoted in ppm and coupling constants (*J*) in Hz. Residual signals from the CDCl_3_ (7.26 ppm for ^1^H-NMR and 77.16 ppm for ^13^C-NMR) and deuterium oxide (4.79 ppm for ^1^H-NMR) were used as an internal reference [94]. NMR spectra in the Supplementary Information were produced utilising TopSpin 4.2.0 [95]. The boron hump is visible between ~10 and ~–40 ppm in the ^11^B-NMR spectra. This arises from borosilicate compounds contained in the NMR tubes and the NMR probe [96,97,98]. Pulse sequences can also be advantageous in reducing the magnitude of the boron hump [99].

Infrared (IR) Spectroscopy: IR spectra were obtained on a PerkinElmer Spectrum Two Spectrometer and on a PerkinElmer Spectrum 2 with UATR. Only characteristic peaks are quoted and in units of cm^−1^.

Low Resolution Mass Spectrometry (LRMS) spectra were obtained on an Agilent Technologies 1260 Infinity UPLC system with a 6120 Quadrupole LC/MS in electrospray ionization (ESI) positive and negative modes. All LCMS methods used a mobile phase A of 100% water with 0.1% formic acid and a mobile phase B of 9:1 *v*/*v* ACN/water with 0.1% formic acid.

Optical rotations were carried out on a Jasco P-2000 polarimeter with a length of 1.0 dm. Concentrations are quoted in g/100 mL.

Melting points were taken on a Dynalon SMP100 Digital Melting Point Device and are uncorrected.

#### 3.2.6. Reagents and Solvents

Solvents: Dichloromethane, *N*,*N*-dimethylformamide, acetic acid, and pyridine were purchased from the Aldrich Chemical Company in Sure-Seal^TM^ reagent bottles. Reverse osmosis water was used. All other solvents (analytical or HPLC grade) were used as supplied without further purification. Deuterated solvents were purchased from Cambridge Isotope Laboratories.

Reagents: Reactions performed in dry conditions and under an atmosphere of nitrogen or hydrogen were maintained by an inflated balloon. *p*-Toluenesulfonyl chloride was recrystallised before use. The reagents were used as provided without further purification, with NMR analysis confirming an acceptable degree of purity and correct structural identity.

Purification via silica gel column chromatography was performed on Davisil 40–63-micron silica gel. Thin layer chromatography (t.l.c.) was performed on aluminium sheets coated with 60 F254 silica by Merck and visualised using UVG-11 Compact UV lamp (254 nm) or stained with a solution of 12 g ammonium molybdate and 0.5 g ceric ammonium molybdate in 15 mL concentrated sulfuric acid and 235 mL distilled water.

### 3.3. Chemistry Experimental

#### 3.3.1. *N*-Benzyl-3,6-dideoxy-3,6-imino-1,2-*O*-isopropylidene-α-d-gulofuranose **3**

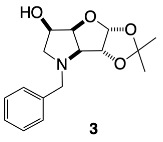



3,6-Dideoxy-3,6-imino-1,2-*O*-isopropylidene-α-d-gulofuranose *p*-toluenesulfonate **2** (297 mg, 0.795 mmol, 1.1 eq.) was dissolved in DMF (30 mL) prior to the addition of K_2_CO_3_ (101 mg, 0.731 mmol, 1.0 eq.) and benzyl bromide (87 µL, 0.731 mmol, 1.0 eq.). The reaction was heated to 100 °C and allowed to stir for 19 h. T.l.c. analysis (EtOAc/hexane, 1:4) revealed the formation of one product (R*_f_* 0.30) and the complete consumption of the starting material (R*_f_* 0.00). The solvent was evaporated, and the crude dissolved in CHCl_3_ (65 mL) and washed with NaOH (aq 0.1 M, 25 mL), concentrated in vacuo, and further triturated in ethyl acetate/hexane (1:1) (20 mL) to give *N*-benzyl-3,6-dideoxy-3,6-imino-1,2-*O*-isopropylidene-α-d-gulofuranose **3** (142 mg, 0.487 mmol, 61%). *m*/*z* (LCMS ES^+^): found mass 292.2 [M+H^+^]^+^, required mass 292.3 [M+H^+^]^+^; [α]_289_^23^ −0.11 (c 0.09 in MeOH); ν_max_ (thin film, cm^−1^): 3360 (m, broad, OH), 3065 and 3030 (w, ArCH), 2989 (m) 2934 (m) 2856 (m) 2801 (m, alkyl CH), 1716 (m, mono-subst, overtone), 1665 (s), 1604 (m) 1496 (m) 1456 (m, Ar C=C), 1382 (s), 1374 (s), 1337 (w), 1310 (w, C-O), 1218 (s) 1164 (s, OH bend), 1080 (s) and 1038 (s, C-N, bend and stretch), 1015 (s), 910 (m), 880 (m), 860 (m), 820 (m), 788 (w), 728 (s) and 697 (s, mono-subst), 664 (m), 646 (m), 620 (m), 554 (m), 515 (m); δ_H_ (CDCl_3_, 400 MHz): 7.36–7.24 (5H, m, 5 x ArCHs), 5.99 (1H, d, *J*_H-1,H-2_ 3.5 Hz, H-1), 4.83 (1H, t, *J*_H-4,H-3/H-5_ 5.8 Hz, H-4), 4.51 (1H, d, *J*_H-2,H-1_ 3.5 Hz, H-2), 4.15 (1H, app-dddd, *J*_H-5,H-6/H-4/OH_ 5.8 Hz, *J*_H-5,H-6′_ 2.0 Hz, H-5), 3.95 (1H, d, *J*_Ha,Hb_ 13.4 Hz, ArC*H^a^*H^b^), 3.49 (1H, d, *J*_Hb,Ha_ 13.4 Hz, ArCH^a^*H^b^*), 3.26 (1H, d, *J*_H-3,H-4_ 5.6 Hz, H-3), 2.93 (1H, dd, *J*_H-6,H-6′_ 10.8 Hz, *J*_H-6,H-5_ 2.1 Hz, H-6), 2.83 (1H, d, *J*_OH,H-5_ 6.3 Hz, OH), 2.43 (1H, dd, *J*_H-6′,H-6_ 10.8 Hz, *J*_H-6′,H-5_ 5.5 Hz, H-6′), 1.51, 1.33 (6Hs, 2 x s, 2 x CH_3_ acetonide); δ_c_ (CDCl_3_, 100 MHz): 137.7 (ArC_quat_), 128.9, 128.5, 127.4 (5 x ArCHs), 112.8 (C_quat_ acetonide), 107.6 (C-1), 84.1 (C-2), 83.1 (C-4), 72.6 (C-3), 69.6 (C-5), 61.3 (C-6), 58.1 (ArCH_2_), 27.7, 26.9 (2 x CH_3_, acetonide).

#### 3.3.2. *N*-Benzyl-3,6-dideoxy-3,6-imino-d-gulofuranose **4**

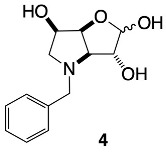



*N*-benzyl-3,6-dideoxy-3,6-imino-1,2-*O*-isopropylidene-α-d-gulofuranose **3** (121 mg, 0.416 mmol) was dissolved in HCl (aq 1M, 12 mL) and allowed to stir at r.t. for 19.5 h. T.l.c. analysis (EtOAc/hexane, 1:4) indicated a complete consumption of the starting material (R*_f_* 0.30) and appearance of a single spot (R*_f_* 0.00). The solvent was evaporated to give *N*-benzyl-3,6-dideoxy-3,6-imino-d-gulofuranose **4** (142 mg, quant.) as a brown oil, as a mixture of two anomers by NMR. *m*/*z* (LRMS ES^+^): found mass 252.1 [M+H^+^]^+^, required mass 252.1 [M+H^+^]^+^; [α]_289_^25^ 0.14 (c 0.08 in MeOH); ν_max_ (thin film, cm^−1^): 3250 (broad, OH), 3369, 3139, 3034 (ArCH), 2291 (alkyl CH), 1642, 1397 (ArC=C), 1083 (C-N). δ_H_ (D_2_O, 400 MHz): the anomeric ratio ***α-fur*/*β-fur*/open-chain 1.0:0.4:0.001**. **Open-chain (only selected signals):** 9.53 (1H, s, CHO); **Major species (*α-fur*)**: δ_H_: 7.60–7.47 (5H, m, 5 x ArCHs), 5.51 (1H, d, *J*_H-1,H-2_ 4.4 Hz, H-1), 4.98 (1H, app-d, *J* 8.3 Hz, H-4), 4.57 (1H, d, *J*_Ha,Hb_ 13.0 Hz, ArC*H^a^*H^b^), 4.49 (1H, d, *J*_Hb,Ha_ 13.0 Hz, ArCH^a^*H^b^*), 4.48–4.43 (2H, m, partially obscured, H-2 and H-5), 4.31 (1H, dd, *J*_H-3,H-4_ 8.2 Hz, *J*_H-3,H-2_ 6.1 Hz, H-3), 3.66–3.60 (1H, m, H-6), 3.57 (1H, dd, *J*_H-6′,H-6_ 12.4 Hz, *J*_H-6′,H-5_ 3.6 Hz, H-6′); δ_c_ (D_2_O, 100 MHz): 142.4 (ArC_quat_), 133.1, 131.1, 129.3 (5 x ArCHs), 99.0 (C-1, slightly broadened, 31 Hz), 78.8 (C-4, slightly broadened, 32 Hz), 72.6 (C-2), 72.2 (C-3), 67.4 (C-5), 60.5–58.8 (C-6, broad, 171 Hz), 60.0–58.2 (ArCH_2_, broad, 177 Hz); **Minor species (*β-fur*)**: δ_H_: 7.60–7.47 (5H, m, 5 x ArCHs), 5.36 (1H, d, *J*_H-1,H-2_ 2.3 Hz, H-1), 5.00 (1H, app-d, *J*_H-4,H-3_ 8.2, H-4), 4.63 (1H, d, *J*_Ha,Hb_ 12.9 Hz, ArC*H^a^*H^b^), 4.57–4.53 (2H, m, obscured, H-2 and H-5), 4.40 (1H, d, *J*_Hb,Ha_ 12.9 Hz, ArCH^a^*H^b^*), 4.23 (1H, dd, *J*_H-3,H-4_ 7.0 Hz, *J*_H-3,H-2_ 2.6 Hz, H-3), 3.81 (1H, dd, *J*_H-6,H-6′_ 12.2 Hz, *J*_H-6,H-5_ 4.9 Hz, H-6) 3.62–3.55 (1H, m, obscured, H-6′); δ_c_ (D_2_O, 100 MHz): 139.4 (ArC_quat_), 130.8, 130.4, 129.5 (5 x ArCHs), 103.2 (C-1), 80.7 (C-4), 72.8 (C-3), 68.6 (C-2, slightly broadened, 21 Hz), 67.5 (C-5), 59.8–58.8 (ArCH_2_, broad, 102 Hz), 57.5 (C-6).

#### 3.3.3. *N*-Benzyl-1,4-dideoxy-1,4-imino-l-gulitol **5**

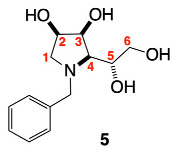



*N*-Benzyl-3,6-dideoxy-3,6-imino-d-gulofuranose **4** (66 mg, 0.262 mmol) was dissolved in EtOH (50% aq, 15 mL) prior to the addition of NaBH_4_ (25 mg, 0.628 mmol, 2.4 eq.), and the reaction was allowed to stir at r.t. for 30 min. A further portion of NaBH_4_ (24 mg, 0.634 mmol, 2.4 eq.) was added and let stir for 17 h at r.t. T.l.c. analysis (EtOAc/MeOH, 95:5) revealed the formation of one product (R*_f_* 0.00) and the complete consumption of the starting material (R*_f_* 0.57). The solvent was removed in vacuo and half of the crude material was dissolved in a small amount of MeOH and H_2_O and purified by prep TLC (MeOH/CHCl_4_, 1:4). Three fractions were collected, two of which were determined to contain the desired product and were combined to give *N*-benzyl-1,4-dideoxy-1,4-imino-l-gulitol **5** (20.8 mg, 0.082 mmol, 88% based on prep-TLC purification mass). m/z (LRMS ES^+^): found mass 254.1 [M+H^+^]^+^, required mass 254.1 [M+H^+^]^+^; [α]_289_^25^ –0.04 (c 0.08 in MeOH); ν_max_ (thin film, cm^−1^): 3600–2500 s, broad, OH), 3137, 3043 (s, ArCH), 2825 (m, alkyl CH), 1647 (s, broad, ArC=C), 1444 (m, ArC=C), 1404 (s, ArC=C), 1213 (w, C-O), 1176 (w), 1122 and 1085 (m, OH bend), 1070, 1036 and 1011 (m, C-N bend and stretch), 962 (m), 935 (m), 886 (m), 859 (m), 817 (m), 755 (m, mono-subst), 702 (m, mono-subst), 685 (m), 602 (m), 569 (m); δ_H_ (D_2_O, 400 MHz): 7.61–7.52 (5H, m, 5 x ArCHs), 4.79 (1H, obscured, ArC*H^a^*H^b^), 4.55 (1H, dddd, *J*_H-2,H-1′_ 9.3 Hz, *J*_H-2,H-1/H-3_ 6.9 Hz, *J* 3.6 Hz, H-2), 4.42 (1H, ddd, *J*_H-3,H-2_ 7.1 Hz, *J*_H-3,H-4_ 4.2 Hz, *J* 3.0 Hz, H-3), 4.38 (1H, ddd, *J*_H-5,H-4_ 8.5 Hz, *J*_H-5,H-6′_ 4.7 Hz, *J*_H-5,H-6_ 3.2 Hz, H-5), 4.29 (1H, d, *J*_Hb,Ha_ 12.8 Hz, ArCH^a^*H^b^*, broad), 3.85 (1H, dd, *J*_H-6,H-6′_ 12.6 Hz, *J*_H-6,H-5_ 3.2 Hz, H-6), 3.84 (1H, dd, *J*_H-4,H-5′_ 9.3 Hz, *J*_H-4,H-3_ 4.0 Hz, H-6), 3.70 (1H, dd, *J*_H-6′,H-6_ 12.8 Hz, *J*_H-6′,H-5_ 4.8 Hz, H-6′), 3.58 (1H, dd, *J*_H-1,H-1′_ 12.0 Hz, *J*_H-1,H-2_ 7.0 Hz, H-1), 3.27 (1H, dd, *J*_H-1′,H-1_ 12.1 Hz, *J*_H-1′,H-2_ 9.1 Hz, H-1′); δ_c_ (CDCl_3_, 100 MHz): 131.0 (2 x ArCH), 130.2 (1 x ArCH), 129.3 (2 x ArCH), 70.2 (C-3), 70.1 (C-4), 68.9 (C-2), 68.6 (C-5), 63.1 (C-6), 61.6 (ArCH_2_), 53.1 (C-1). ArC_quat_ is not discernible.

#### 3.3.4. *N*-(4-Methylphenyl boronic acid pinacol ester)-3,6-dideoxy-3,6-imino-1,2-*O*-isopropylidene-α-d-gulofuranose **para 6**

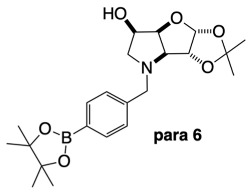



3,6-Dideoxy-3,6-imino-1,2-*O*-isopropylidene-α-d-gulofuranose *p*-toluenesulfonate **5** (306 mg, 0.803 mmol, 1.1 eq.) was dissolved in DMF (30 mL) prior to the addition of K_2_CO_3_ (109 mg, 0.730 mmol, 1.0 eq.) and 4-bromomethylphenyl boronic acid pinacol ester (221 mg, 0.730 mmol, 1.0 eq.). The reaction was heated to 100 °C and allowed to stir for 19 h. T.l.c. analysis (EtOAc/hexane, 1:4) revealed formation of one product (R*_f_* 0.27) and complete consumption of the starting material (R*_f_* 0.00). The solvent was evaporated and the crude dissolved in CHCl_3_ (90 mL) and washed with NaOH (aq. 0.1 M, 30 mL) to give *N*-(4-methylphenylboronic acid pinacol ester)-3,6-dideoxy-3,6-imino-1,2-*O*-isopropylidene-α-d-gulofuranose **para 6** in the organic layer as a brown oil (296 mg, 0.710 mmol, 97%). *m*/*z* (LRMS ES^+^): found mass 418.2 [M+H^+^]^+^, required mass 418.2 [M+H^+^]^+^; [α]_289_^25^ 0.2 (c 0.15 in MeOH); ν_max_ (thin film, cm^−1^): 3454 (w, broad, OH), 2983, 2934 (alkyl CH), 2251 (w), 1724 (w) 1714 (w) 1668 (w, *para*-subst, overtone), 1615 (m) 1515 (m) 1454 (m, ArC=C), 1398 (m, B-O), 1359 (s, B-O), 1321 (sharp m, C-O), 1272 (m), 1216 (m, B-O-H bend), 1163 (m, OH bend), 1143 (s), 1086 (s, B-C), 1069 (m) and 1021 (s, C-N bend and stretch), 962 (m), 909 (s), 858, 827 (m, *para*-subst), 728 (s), 660 (m), 648 (m), 577 (w), 566 (w), 555 (w), 516 (w); δ_H_ (CDCl_3_, 400 MHz): 7.77 (2H, d, *J* 7.9 Hz, 2 x ArCH), 7.28 (2H, d, *J* 7.9 Hz, 2 x ArCH), 5.99 (1H, d, *J*_H-1,H-2_ 3.5 Hz, H-1), 4.80 (1H, app-t, *J*_H-4,H-3/H-5_ 5.8 Hz, H-4), 4.52 (1H, d, *J*_H-2,H-1_ 3.5 Hz, H-2), 4.13 (1H, app-dddd, *J*_H-5,H-6/H-4/OH_ 6.0 Hz, *J*_H-5,H-6′_ 2.3 Hz, H-5), 3.95 (1H, d, *J*_Ha,Hb_ 13.6 Hz, ArC*H^a^*H^b^), 3.51 (1H, d, *J*_Hb,Ha_ 13.6 Hz, ArCH^a^*H^b^*), 3.25 (1H, d, *J*_H-3,H-4_ 5.5 Hz, H-3), 2.90 (1H, dd, *J*_H-6,H-6′_ 10.8 Hz, *J*_H-6,H-5_ 2.3 Hz, H-6), 2.81 (1H, d, *J*_OH,H-5_ 6.5 Hz, OH), 2.42 (1H, dd, *J*_H-6′,H-6_ 10.8, *J*_H-6′,H-5_ 5.6 Hz, H-6′), 1.50 (3H, s, CH_3_ acetonide), 1.33 and 1.32 (15H, 2 x s, 1 x CH_3_ acetonide, 4 x CH_3_ pinacol); δ_c_ (CDCl_3_, 100 MHz): 140.8 (ArC_quat_), 135.0, 128.2 (4 x ArCHs), 112.7 (C_quat_ acetonide), 107.6 (C-1), 84.0 (C-2), 83.9 (2 x C_quat_ pinacol), 83.1 (C-4), 72.5 (C-3), 69.6 (C-5), 61.2 (C-6), 58.1 (ArCH_2_), 27.7, 26.9 (2 x CH_3_, acetonide), 25.0, 24.9 (2 x (CH_3_)_2_ pinacol). ArC_quat_-B was not discernible. δ_B_ (CDCl_3_, 128 MHz): 30.6 (integration: 3.65), 22.3 (integration: 1.00).

#### 3.3.5. *N*-(4-Methylphenyl boronic acid)-3,6-dideoxy-3,6-imino-d-gulofuranose **para 7**

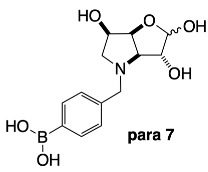



*N*-(4-Methylphenyl boronic acid pinacol ester)-3,6-dideoxy-3,6-imino-1,2-*O*-isopropylidene-α-d-gulofuranose **para 6** (296 mg, 0.709 mmol) was dissolved in HCl (aq 1M, 30 mL) and allowed to stir at 40 °C for 17 h. T.l.c. analysis (EtOAc/hexane, 1:4) indicated a complete consumption of the starting material (R*_f_* 0.27) and appearance of a single spot (R*_f_* 0.0). The solvent was evaporated to give the desired crude product *N*-(4-methylphenyl boronic acid)-3,6-dideoxy-3,6-imino-d-gulofuranose **para 7** and the remaining free pinacol as a brown residue (254 mg). *m*/*z* (LRMS ES^+^): found mass 296.1 [M+H^+^]^+^, required mass 296.1 [M+H^+^]^+^; [α]_289_^19^ +0.15 (c 0.047 in MeOH); ν_max_ (thin film, cm^−1^): 3359 (s, broad, OH), 2982 (m, alkyl CH), 2605, 2367 (w), 1616 (m, with shoulder), 1521 (w, ArC=C), 1413 (m, B-O), 1364 (s, with shoulder, B-O), 1276 (m, C-O), 1212 (m, B-O-H bend), 1143 (m, OH bend), 1128 (m), 1108 (m), 1090 (m, OH bend), 1038, 1022 (m, C-N bend and stretch), 857 (w, *para*-subst), 824 (w, *para*-subst), 794, 657, 567, 485, 455, 437, 406 (w); ***α-fur*:*β-fur* ratio 1.0:0.4. Major species (*α-fur*)**: δ_H_ (D_2_O, 400 MHz): 7.87 (2H, d, *J* 7.9 Hz, 2 x ArCH), 7.59 (2H, d, *J* 7.9 Hz, 2 x ArCH), 5.51 (1H, d, *J*_H-1,H-2_ 4.3 Hz, H-1), 4.99 (1H, app-d, partially obscured, *J*_H-4,H-3_ 8.2 Hz, H-4), 4.59 (1H, d, *J*_Ha,Hb_ 13.0 Hz, ArC*H^a^*H^b^), 4.51 (1H, d, *J*_Hb,Ha_ 13.0 Hz, ArCH^a^*H^b^*), 4.49–4.44 (2H, m, H-2 and H-5), 4.32 (1H, dd, *J*_H-3,H-4_ 8.1 Hz, *J*_H-3,H-2_ 6.3 Hz, H-3), 3.65–3.60 (1H, m, H-6), 3.57 (1H, dd, *J*_H-6′,H-6_ 12.2 Hz, *J*_H-6′,H-5_ 3.4 Hz, H-6′); δ_c_ (D_2_O, 100 MHz): 143.0 (ArC_quat_), 134.3, 130.5 (2 x 2Cs, 4 x ArCHs), 99.0 (C-1, broadened, 46 Hz), 78.8 (C-4, broadened, 56 Hz), 72.6 (C-2, slightly broadened, 38 Hz), 72.3 (C-3, slightly broadened, 18 Hz), 67.5 (C-5), 60.5–58.8 (broadened, 173 Hz, C-6), 58.8–58.1 (broadened, 76 Hz, ArCH_2_). ArC_quat_-B was not discernible.

**Minor species (*β-fur*)**: δ_H_ (D_2_O, 400 MHz): 7.88 (2H, d, *J* 7.9 Hz, 2 x ArCH), 7.60 (2H, d, *J* 7.9 Hz, 2 x ArCH), 5.36 (1H, d, *J*_H-1,H-2_ 2.2 Hz, H-1), 5.00 (1H, app-d, partially obscured, *J* 8.5 Hz, H-4), 4.65 (1H, d, *J*_Ha,Hb_ 13.0 Hz, ArC*H^a^*H^b^), 4.56–4.53 (1H, obscured, H-5) 4.42 (1H, d, *J*_Hb,Ha_ 13.0 Hz, ArCH^a^*H^b^*), 4.36–4.29 (1H, obscured, H-2), 4.22 (1H, dd, *J* 6.9 Hz, *J* 2.4 Hz, H-3), 3.82 (1H, dd, *J*_H-6,H-6′_ 12.6 Hz, *J*_H-6,H-5_ 5.0 Hz, H-6), 3.62–3.50 (1H, partially obscured, *J*_H-6′,H-5_ 5.2 Hz, H-6′); δ_c_ (D_2_O, 100MHz): 134.5, 130.2 (2 x 2Cs, 4 x ArCHs),103.2 (C-1, slightly broadened, 28 Hz), 80.7 (C-4), 72.9 (C-3, slightly broadened, 19 Hz), 72.3 (C-2, slightly broadened, 18 Hz), 67.4 (C-5), 60.5–58.8 (broadened, 173 Hz, C-6 and ArCH_2_). ArC_quat_-B and ArC_quat_ were not discernible.

δ_B_ (D_2_O, 128 MHz): 28.7 (integration: 9.33), 19.4 (integration: 1.00).

#### 3.3.6. *N*-(4-Methylphenyl boronic acid)-1,4-dideoxy-1,4-imino-l-gulitol **para 8**

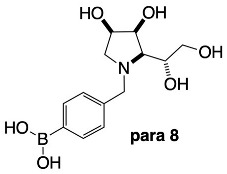



*N*-(4-Methylphenyl boronic acid)-3,6-dideoxy-3,6-imino-d-gulofuranose **para 7** (84 mg, 0.285 mmol) was dissolved in EtOH (50% aq, 20 mL) prior to the addition of NaBH_4_ (15 mg, 0.391 mmol, 1.4 eq.), and the reaction was allowed to stir at r.t. for 30 min. Additional NaBH_4_ (14 mg, 0.342 mmol, 1.2 eq.) was added, and the reaction was allowed to stir for 30 more minutes. T.l.c. analysis (EtOAc/MeOH, 9:1) revealed formation of one product (R*_f_* 0.00–0.10) and complete consumption of the starting material (R*_f_* 0.70–0.95). After quenching with glacial acetic acid, the solvents were evaporated, and the crude product was triturated in MeOH/EtOH (1:1). Solvents were evaporated, and the residue was dissolved in MeOH with the addition of ethyl acetate until a white precipitate was observed, then filtered. The filtrate was evaporated to give *N*-(4-methylphenyl boronic acid)-1,4-dideoxy-1,4-imino-l-gulitol **para 8** (41 mg, 0.138 mmol, 48%). m/z (LRMS ES^+^): found mass 298.1 [M+H^+^]^+^, required mass 298.1 [M+H^+^]^+^; [α]_289_^19^ –0.03 (c 0.04 in H_2_O); ν_max_ (thin film, cm^−1^): 3368 (s, broad, OH), 2945 (w, alkyl CH), 2723, 2682, 2623 (w), 1676 (m, C-N), 1641, 1615, 1519, 1457 (m, ArC=C), 1397 (m, B-O), 1366 (m, B-O), 1270 (m, C-O), 1205 (m, O-H bend), 1177 (m, B-O-H bend), 1083, 1066 (s, C-N, bend and stretch), 1035 (m, B-C), 1012, 954 (s), 837 (m, *para*-subst), 790 (m), 755 (m), 704 (m), 595 (m), 559 (m), 498 (m), 461 (m); δ_H_ (D_2_O, 400 MHz): 7.85 (2H, dd, ^3^*J*_ArH,ArH_ 8.0 Hz, ^5^*J*_ArH,ArH_ 1.6 Hz, 2 x ArCH), 7.57 (2H, dd, ^3^*J*_ArH,ArH_ 8.0 Hz, ^5^*J*_ArH,ArH_ 1.7 Hz, 2 x ArCH), 4.83 (1H, obscured, ArC*H^a^*H^b^), 4.54–4.50 (1H, m, H-2), 4.40–4.34 (2H, m, H-6 and H-4), 4.27 (1H, d, *J*_Hb,Ha_ 13.0 Hz, ArCH^a^*H^b^*), 3.82 (1H, app-dd, *J*_H-5,H-6_ 8.9 Hz, *J*_H-5,H-6′_ 5.1 Hz, H-5), 3.79 (1H, app-d, *J* 3.2 Hz, H-3), 3.66 (1H, dd, *J*_H-6′,H-6_ 12.0 Hz, *J*_H-6′,H-5_ 5.2 Hz, H-6′), 3.55 (1H, dd, *J*_H-1,H-1′_ 12.0 Hz, *J*_H-1,H-2_ 6.9 Hz, H-1), 3.25 (1H, dd, *J*_H-1′,H-1_ 12.2 Hz, *J*_H-1′,H-2_ 9.2 Hz, H-1′).

The main discernible boronate anion and/or boronic acid signals can be found at δ_H:_ 7.85 (2H, integration 0.35, obscured), 7.78 (2H, integration 0.5, d, *J* 8.0 Hz), 7.51 (2H, integration 0.46, d, *J* 8.1 Hz), 7.43 (2H, integration 0.5, d, *J* 8.0 Hz), 7.42 (2H, integration 0.3, d, *J* 8.5 Hz), 6.96 (2H, integration 0.44, dd, *J* 8.6, 1.5 Hz), 4.80 (1H, obscured, ArC*H^a^*H^b^), 4.21 (1H, d, *J* 7.9 Hz, ArCH^a^*H^b^*). δ_c_ (D_2_O, 100 MHz): 134.5 (ArC_quat_), 134.3, 130.4 (4 x ArCH), 70.3 (C-4), 70.3 (C-3), 68.9 (C-2), 63.1 (C-6, C-5), 61.5 (ArCH_2_), 53.2 (C-1); the ArC_quat_-B was not discernible. The main discernible other signals can be found at δ_C_: 134.4, 134.0, 132.2, 126.8, 115.9, 75.7 ppm.

δ_B_ (D_2_O, 128 MHz): 29.0 (integration 3.93), 19.3 (integration 1.00).

## 4. Conclusions

The synthesis, spectroscopic, and biological investigation of five-membered ring iminosugars bearing an organic boron pharmacophore is reported. This is an important novel class of iminosugars capable of establishing not only the traditional intermolecular interactions with enzyme active sites, but are also imbued with the capability of reversible intramolecular interactions via dative bonding from nucleophilic amino acid side chains and the boron atom’s empty *p*-orbital. This communication reports pyrrolidine iminosugars of L-gulose absolute stereochemical configuration that are functionalised via *N*-alkylation to bear a boronate ester and boronic acid groups. This family of iminosugars displays selective, moderate-to-weak inhibition (IC_50_s = 133–501 mM) of β-d-galactosidase (bovine liver) and emerging inhibition of β-d-glucosidases (almond) and (bovine liver). The boronic acid pharmacophore may be suitable for the management of lysosomal storage disorders to support restoration of the biological activity of mutant enzymes via the chaperone-mediated therapy approach. From a structure–activity perspective, the cancer screening revealed slight growth inhibition in a panel of cancer cell lines, with A2780 ovarian carcinoma cells showing the strongest response across all compounds. Beyond the growth inhibition capabilities, the real therapeutic potential of these borylated drugs lies in their switch-on/switch-off activation under BNCT radiotherapeutic conditions.

## Figures and Tables

**Table 4:** ^11^B-NMR Signals for **para 6** in various deuterated solvents.

Solvent	Chemical Shift, ppm	Proposed Assignment
CDCl_3_	30 (1.00)	Boronate ester (trigonal planar)
	22 (0.35)	Solvent CDCl_3_ → aromatic π-system or CDCl_3_ → B *p*-orbital (partially tetrahedral)
MeOD	32 (1.00)	Boronate ester (trigonal planar)
	18 (0.06)	Solvent MeOD → B *p*-orbital (partially tetrahedral)
MeOD acidified with HCl to pH 1	29.8 (1.00)	Boronate ester (trigonal planar)
	19.3 (0.12)	Solvent MeOD → B *p*-orbital (partially tetrahedral)

**Table 5:** Summary of glycosidase inhibitions by *N*-benzyl-1,4-dideoxy-1,4-imino derivatives.

Iminosugar	Glycosidase/s Most Inhibited
*N*-Benzyl-1,4-dideoxy-1,4-imino-	
d-allitol [69]	α-l-fucosidase (76%)
d-galactitol [65]	α-d-glucosidase (93.2%), IC_50_ = 40.6 μM
d-mannitol.HCl [66,83,84]	α-d-mannosidases (lysosomal acidic, 34%), (neutral, 44%), and (Golgi II, 72%)
d-talitol.HCl [85]	NI
*N*-(2-Methylphenyl boronic acid)-1,4-dideoxy-1,4-imino-d-talitol.HCl [85]	β-d-galactosidase (*E. coli*) (44–55%)

**Table 6:** Glycosidase inhibition studies. Percentage inhibition data for controls (BSH, ^10^B-BSH, BPA, ^10^B-BPA), intermediates **3**, **4**, **para 6**, and **para 7**, and target compounds **5** and **para 8** in a panel of 15 glycosidases. IC_50_ values (on green coloured background) for the most potent activities. NA = not available. Blue background denotes biological activity > 30% under conditions screened.

Compound	α-d-Glucosidase			β-d- Glucosidase		α-d- Galactosidase	β-d- Galactosidase	α-d- Mannosidase	β-d- Mannosidase	α-l- Rhamnosidase	α-l- Fucosidase	β-d- Glucuronidase		α,α- Trehalase	Amyloglucosidase
	Rice	Yeast	Rat Intestinal Maltase	Almond	Bovine Liver	Coffee Beans	Bovine Liver	Jack Bean	Snail	*P. decumbens*	Bovine Kidney	*E. coli*	Bovine Liver	Porcine Kidney	*A. niger*
**BSH**	^a^ NI ^b^ (0%)	^a^ NI ^b^ (6.9%)	^a^ NI ^b^ (0%)	^a^ NI ^b^ (0%)	^a^ NI ^b^ (15%)	^a^ NI ^b^ (12.3%)	^a^ NI ^b^ (0%)	^a^ NI ^b^ (7.9%)	^a^ NI ^b^ (19.2%)	^a^ NI ^b^ (0.2%)	^a^ NI ^b^ (0%)	^a^ NI ^b^ (6%)	^a^ NI ^b^ (19.6%)	^a^ NI ^b^ (4.2%)	^a^ NI ^b^ (0%)
**^10^B-BSH**	^a^ NI ^b^ (0%)	^a^ NI ^b^ (5.6%)	^a^ NI ^b^ (0%)	^a^ NI ^b^ (0%)	^a^ NI ^b^ (11.9%)	^a^ NI ^b^ (3.9%)	^a^ NI ^b^ (22.3%)	^a^ NI ^b^ (7.7%)	^a^ NI ^b^ (15%)	^a^ NI ^b^ (0%)	^a^ NI ^b^ (6.5%)	^a^ NI ^b^ (3.2%)	^a^ NI ^b^ (12.5%)	^a^ NI ^b^ (2.3%)	^a^ NI ^b^ (0%)
**BPA**	^c^ NI ^d^ (0%)	^c^ NI ^d^ (0%)	^c^ NI ^d^ (0%)	^c^ NI ^d^ (0%)	^c^ NI ^d^ (0%)	^c^ NI ^d^ (4.3%)	^c^ NI ^d^ (10.9%)	^c^ NI ^d^ (1.1%)	^c^ NI ^d^ (2.1%)	^c^ NI ^d^ (0%)	^c^ NI ^d^ (0%)	^c^ NI ^d^ (0.5%)	^c^ NI ^d^ (7.4%)	^c^ NI ^d^ (0%)	^c^ NI ^d^ (0%)
**^10^B-BPA**	^c^ NI ^d^ (0%)	^c^ NI ^d^ (0%)	^c^ NI ^d^ (0%)	^c^ NI ^d^ (0%)	^c^ NI ^d^ (14.2%)	^c^ NI ^d^ (1.2%)	^c^ NI ^d^ (0%)	^c^ NI ^d^ (0%)	^c^ NI ^d^ (0.3%)	^c^ NI ^d^ (0%)	^c^ NI ^d^ (0%)	^c^ NI ^d^ (2.4%)	^c^ NI ^d^ (0%)	^c^ NI ^d^ (0%)	^c^ NI ^d^ (0%)
**3**	^c^ NI ^d^ (0%)	^c^ NI ^d^ (4.7%)	^c^ NI ^d^ (0%)	^c^ NI ^d^ (0%)	^c^ NI ^d^ (31.9%)	^c^ NI ^d^ (9.5%)	^c^ NI ^d^ (26.1%)	^c^ NI ^d^ (0.9%)	^c^ NI ^d^ (2.7%)	^c^ NI ^d^ (0.5%)	^c^ NI ^d^ (0%)	^c^ NI ^d^ (2.4%)	^c^ NI ^d^ (0%)	^c^ NI ^d^ (0%)	^c^ NI ^d^ (0%)
**4**	^a^ NI ^b^ (1.3%)	^a^ NI ^b^ (0%)	^a^ NI ^b^ (22.4%)	^a^ NI ^b^ (27.4%)	^a^ NI ^b^ (16.4%)	^a^ NI ^b^ (1.8%)	133	^a^ NI ^b^ (7.5%)	^a^ NI ^b^ (1.0%)	^a^ NI ^b^ (0%)	^a^ NI ^b^ (0.4%)	^a^ NI ^b^ (7.0%)	^a^ NI ^b^ (7.0%)	^a^ NI ^b^ (0%)	^a^ NI ^b^ (0.2%)
**5**	^c^ NI ^d^ (0%)	^c^ NI ^d^ (2.9%)	^c^ NI ^d^ (12.9%)	^c^ NI ^d^ (0%)	^c^ NI ^d^ (3.3%)	^c^ NI ^d^ (1.5%)	^c^ NI ^d^ (0%)	^c^ NI ^d^ (4.4%)	^c^ NI ^d^ (6.9%)	^c^ NI ^d^ (1.5%)	NA	^c^ NI ^d^ (0%)	^c^ NI ^d^ (3.7%)	^c^ NI ^d^ (0%)	^c^ NI ^d^ (2.1%)
**para 6**	^c^ NI ^d^ (0%)	^c^ NI ^d^ (3.4%)	^c^ NI ^d^ (0%)	^c^ NI ^d^ (0%)	^c^ NI ^d^ (13%)	^c^ NI ^d^ (1.6%)	^c^ NI ^d^ (22.3%)	^c^ NI ^d^ (0%)	^c^ NI ^d^ (0%)	^c^ NI ^d^ (1.4%)	^c^ NI ^d^ (0%)	^c^ NI ^d^ (3.2%)	^c^ NI ^d^ (0%)	^c^ NI ^d^ (0%)	^c^ NI ^d^ (0%)
**para 7**	^a^ NI ^b^ (0%)	^a^ NI ^b^ (0%)	^a^ NI ^b^ (0%)	^a^ NI ^b^ (43.6%)	^a^ NI ^b^ (38.8%)	^a^ NI ^b^ (0%)	218	^a^ NI ^b^ (0%)	^a^ NI ^b^ (4.1%)	^a^ NI ^b^ (15.8%)	^a^ NI ^b^ (0.8%)	^a^ NI ^b^ (17.8%)	^a^ NI ^b^ (3.5%)	^a^ NI ^b^ (0%)	^a^ NI ^b^ (2.7%)
**para 8**	^a^ NI ^b^ (8.2%)	^a^ NI ^b^ (5.6%)	^a^ NI ^b^ (10.5%)	^a^ NI ^b^ (28%)	^a^ NI ^b^ (27.1%)	^a^ NI ^b^ (1.4%)	501	^a^ NI ^b^ (0%)	^a^ NI ^b^ (6.9%)	^a^ NI ^b^ (26.3%)	^a^ NI ^b^ (1.7%)	^a^ NI ^b^ (22.9%)	^a^ NI ^b^ (6.0%)	^a^ NI ^b^ (0%)	^a^ NI ^b^ (3.6%)

^a^ NI: No inhibition (less than 50% inhibition at 1000 μM). ^b^ ( ): inhibition % at 1000 μM. ^c^ NI: No inhibition (less than 50% inhibition at 100 μM). ^d^ ( ): inhibition % at 100 μM. Compound **5** was also run against α-d-glucosidase (human lysosome) with a ^c^ NI ^d^ (0%) inhibition, and β-d-glucosidase (human lysosome) with a ^c^ NI ^d^ (2.91%) inhibition, and β-d-galactosidase (*E. coli*) with a ^c^ NI ^d^ (0%) inhibition, and β-d-glucanase (*T. longibrachiatum*) with a ^c^ NI ^d^ (6.4%) inhibition.

**Table 7:** Glycosidase inhibition studies. Percentage inhibition data for controls (BSH, ^10^B-BSH, BPA, ^10^B-BPA), intermediates **3**, **4**, **para 6,** and **para 7**, and target compounds **5** and **para 8** in a panel of eight glycosidases. NA = not available. The pale blue background denotes inhibitory activity 30–60%, and the blue background denotes inhibitory activity > 60%, under the conditions screened. “Appearance” is recorded under the following conditions: 1 mL of water was added to 1 mg of solid, and mixed.

Compound	Appearance	α-d-Glucosidase			β-d-Glucosidase	α-d-Mannosidase	*N*-Acetyl-β-d- glucosaminidase	*N*-Acetyl-β-d- hexosaminidase	β-d-Glucuronidase
		Yeast	*Bacillus*	Rat Intestine	Almond	Jack Bean	Bovine Kidney	Rat Intestine	Bovine Liver
**BSH**	In solution	59	48.1	NA	47.3	−18.7	37.1	NA	31.6
**^10^B-BSH**	In solution	65.9	53	NA	49.9	−16.8	40.9	NA	44.1
**BPA**	Some in solution with undissolved sediment	2.9	19.9	NA	3.9	0.6	6.7	NA	−0.7
**^10^B-BPA**	Some in solution with undissolved sediment	3.4	19.5	NA	3	−0.7	6.3	NA	−1
**3**	In solution	−89	−43.2	NA	−8.3	−30.6	−0.1	NA	−0.2
**4**	In solution	−79	−37.9	NA	58.1	−12.1	−9.1	NA	−4.3
**5**	In solution	5.5	NA	−1.3	17.1	NA	NA	−1.5	−2.2
**para 6**	*Completely insoluble*	NA	NA	NA	NA	NA	NA	NA	NA
**para 7**	In solution	4.2	NA	−5.4	68.1	NA	NA	4.4	1.1
**para 8**	In solution	9.7	NA	ND	23.9	NA	NA	2.6	2.4

## Data Availability

The data presented in this study are available on request from the corresponding author.

## Supplementary Materials

The following supporting information can be downloaded at: https://www.mdpi.com/article/10.3390/ph18091302/s1. Table S1: Structures of the reference compounds analysed by ^11^B-NMR and their corresponding signals at 128 MHz, dissolved in the deuterated solvent stated in brackets; Figure S1: ^1^H- (400 MHz), ^13^C-NMR (100 MHz), DEPT, COSY and HSQC spectra of *N*-benzyl-3,6-dideoxy-3,6-imino-1,2-*O*-isopropylidene-α-d-gulofuranose **3** in CDCl_3_; Figure S2: ^1^H- (400 MHz), ^13^C-NMR (100 MHz), DEPT, COSY, HSQC and HMBC spectra of *N*-benzyl-3,6-dideoxy-3,6-imino-d-gulofuranose **4** in D_2_O; Figure S3: ^1^H-NMR spectrum (400 MHz, D_2_O) of *N*-benzyl-3,6-dideoxy-3,6-imino-d-gulofuranose **4** with colour-coded signals, highlighting the furanose anomeric forms they belong to, with interpretation of the isolated signals and tentative interpretation of the overlapping ones. Namely, the orange designates the *α-fur* form and indigo designates the *β-fur* form. Section 8.10 ppm to 7.20 ppm is visible with highlighted principal COSY correlations to hydrogen atoms within the same spin systems. Figure S4: ^1^H-NMR spectrum (400 MHz, D_2_O) of *N*-benzyl-3,6-dideoxy-3,6-imino-d-gulofuranose **4** with colour-coded signals, highlighting the furanose anomeric forms they belong to, with interpretation of the isolated signals and tentative interpretation of the overlapping ones. Namely, the orange designates the *α-fur* form and indigo designates the *β-fur*. (A) section 5.60 ppm to 4.90 ppm; (B) section 4.70 ppm to 3.50 ppm. Highlighted are also the principal COSY correlations to hydrogen atoms within the same spin systems; Figure S5: ^13^C-NMR spectrum (100 MHz, D_2_O) sections of *N*-benzyl-3,6-dideoxy-3,6-imino-d-gulofuranose **4** with colour-coded signals, highlighting the furanose anomeric forms they belong to, with interpretation of the isolated signals and tentative interpretation of the overlapping ones. Namely, the orange designates the *α-fur* form and indigo designates the *β-fur*. (A) section 143.0 ppm to 127.5 ppm; (B) section 105.0 ppm to 76.0 ppm; (C) section 75.0 ppm to 48.5 ppm. Highlighted are also the principal HSQC correlations to hydrogen atoms within the same spin systems; Figure S6: ^1^H- (400 MHz), ^13^C-NMR (100 MHz) and COSY spectra of *N*-benzyl-1,4-dideoxy-1,4-imino-l-gulitol **5** in D_2_O; Figure S7: Sections of the ^1^H-NMR spectrum (400 MHz, D_2_O) of *N*-benzyl-1,4-dideoxy-1,4-imino-l-gulitol **5** with selected signals colour-coded. Namely, the orange and green colour-codes are used for overlapping signals to show the splitting pattern. The green and orange vertical lines designate the locations of the peaks for complex splitting patterns. (A) section 4.59 ppm to 4.25 ppm; (B) section 3.92 ppm to 3.20 ppm. Highlighted are also the principal COSY correlations between hydrogen atoms; Figure S8: ^1^H- (400 MHz), ^13^C-NMR (100 MHz), DEPT, ^11^B-NMR (128 MHz), COSY, HSQC and HMBC spectra of *N*-(4-methylphenylboronic acid pinacol ester)-3,6-dideoxy-3,6-imino-1,2-*O*-isopropylidene-α-d-gulofuranose **para 6** in CDCl_3_; Figure S9: ^1^H- (400 MHz), ^13^C-NMR (100 MHz), ^11^B-NMR (128 MHz), COSY, HSQC and HMBC spectra of *N*-(4-methylphenyl boronic acid)-3,6-dideoxy-3,6-imino-d-gulofuranose **para 7** in D_2_O; Figure S10: ^1^H-NMR spectrum (400 MHz, D_2_O) of *N*-(4-methylphenyl boronic acid)-3,6-dideoxy-3,6-imino-d-gulofuranose **para 7** with colour-coded signals, highlighting the furanose anomeric forms they belong to, with interpretation of the isolated signals and tentative interpretation of the overlapping ones. Namely, the orange designates the *α-fur* form and indigo designates the *β-fur*. Section 7.92 ppm to 7.55 ppm is visible with highlighted principal COSY correlations to hydrogen atoms within the same spin systems; Figure S11: ^1^H-NMR spectrum (400 MHz, D_2_O) of *N*-(4-methylphenyl boronic acid)-3,6-dideoxy-3,6-imino-d-gulofuranose **para 7** with colour-coded signals, highlighting the furanose anomeric forms they belong to, with interpretation of the isolated signals and tentative interpretation of the overlapping ones. Namely, the orange designates the *α-fur* form and indigo designates the *β-fur*. (A) section 5.60 ppm to 4.90 ppm; (B) section 4.75 ppm to 3.50 ppm. Highlighted are also the principal COSY correlations to hydrogen atoms within the same spin systems; Figure S12: ^13^C-NMR spectrum (100 MHz, D_2_O) sections of *N*-(4-methylphenyl boronic acid)-3,6-dideoxy-3,6-imino-d-gulofuranose **para 7** with colour-coded signals, highlighting the furanose anomeric forms they belong to, with interpretation of the isolated signals and tentative interpretation of the overlapping ones. Namely, the orange designates the *α-fur* form and indigo designates the *β-fur*. (A) section 103.6 ppm to 97.5 ppm; (B) section 81.0 ppm to 72.0 ppm; (C) section 68.0 ppm to 56.5 ppm. Highlighted are also the principal HSQC correlations to hydrogen atoms in the same spin systems; Figure S13: ^1^H- (400 MHz), ^13^C-NMR (100 MHz), ^11^B-NMR (128 MHz), COSY and HSQC spectra of *N*-(4-methylphenyl boronic acid)-1,4-dideoxy-1,4-imino-l-gulitol **para 8** in D_2_O; Figure S14: ^1^H-NMR spectrum (400 MHz, D_2_O) of *N*-(4-methylphenyl boronic acid)-1,4-dideoxy-1,4-imino-l-gulitol **para 8** with colour-coded signals, highlighting the predominant boronic acid species (orange) and other boronic acid/boronate anion species (shades of green). Section 8.00 ppm to 6.80 ppm is visible with highlighted the principal COSY correlations to hydrogen atoms within the same spin systems; Figure S15: ^1^H-NMR spectrum (400 MHz, D_2_O) of *N*-(4-methylphenyl boronic acid)-1,4-dideoxy-1,4-imino-l-gulitol **para 8** with colour-coded signals, highlighting the predominant boronic acid species (orange) and other boronic acid/boronate anion species (shades of green). (A) section 5.10 ppm to 4.00 ppm; (B) section 4.10 ppm to 3.10 ppm. Highlighted are also the principal COSY correlations to hydrogen atoms within the same spin systems; Figure S16: ^13^C-NMR spectrum (100 MHz, D_2_O) sections of *N*-(4-methylphenyl boronic acid)-1,4-dideoxy-1,4-imino-l-gulitol **para 8**. Only the main set of signals is presented: (A) section 136.5 ppm to 115.0 ppm; (B) section 75.0 ppm to 50.0 ppm; NMR Experimental details; References [47,48,49,52,100] are cited in the supplementary materials.
