# Supplementary material for: Borylated Five-Membered Ring Iminosugars: Synthesis, Spectroscopic Analysis, and Biological Evaluation for Glycosidase Inhibition and Anticancer Properties for Application in Boron Neutron Capture Therapy (BNCT)—Part 1
Source: Pharmaceuticals (Basel). 2025 Aug 29;18(9):1302. doi: 10.3390/ph18091302 (PMC12472391; doi:10.3390/ph18091302)
Supplement: Supplementary file 1 [file pharmaceuticals-18-01302-s001.zip › pharmaceuticals-3629435 - Supplementary Information - Part 1_vii.pdf]

Supplementary Information

Borylated 5-membered Ring Iminosugars: Spectroscopic Analysis, Glycosidase Inhibition and Anticancer Properties for Application in Boron Neutron Capture Therapy (BNCT) – Part 1

Kate Prichard [1], Suzuka Yamamoto [2], Yuna Shimadate [2], Kosuke Yoshimura [2], Barbara Bartholomew [4], Jayne Gilbert [3], Jennette Sakoff [3], Robert Nash [4], Atsushi Kato [5], Michela Simone [1,2,*]

Index

Table S1. Structures of the reference compounds analysed by ^{11}B-NMR and their corresponding signals at 128 MHz, dissolved in the deuterated solvent stated in brackets. **3**

Figure S1. ^{1}H- (400 MHz), ^{13}C-NMR (100 MHz), DEPT, COSY and HSQC spectra of *N*-benzyl-3,6-dideoxy-3,6-imino-1,2-*O*-isopropylidene-*α*-D-gulofuranose **3** in $CDCl_3$. **4**

Figure S2. ^{1}H- (400 MHz), ^{13}C-NMR (100 MHz), DEPT, COSY, HSQC and HMBC spectra of *N*-benzyl-3,6-dideoxy-3,6-imino-D-gulofuranose **4** in D_2O. **12**

Figure S3. ^{1}H-NMR spectrum (400 MHz, D_2O) of *N*-benzyl-3,6-dideoxy-3,6-imino-D-gulofuranose **4** with colour-coded signals, highlighting the furanose anomeric forms they belong to, with interpretation of the isolated signals and tentative interpretation of the overlapping ones. Namely, the orange designates the *α-fur* form and indigo designates the *β-fur* form. Section 8.10 ppm to 7.20 ppm is visible with highlighted the principal COSY correlations to hydrogen atoms within the same spin systems. **34**

Figure S4. ^{1}H-NMR spectrum (400 MHz, D_2O) of *N*-benzyl-3,6-dideoxy-3,6-imino-D-gulofuranose **4** with colour-coded signals, highlighting the furanose anomeric forms they belong to, with interpretation of the isolated signals and tentative interpretation of the overlapping ones. Namely, the orange designates the *α-fur* form and indigo designates the *β-fur*. A) section 5.60 ppm to 4.90 ppm; B) section 4.70 ppm to 3.50 ppm. Highlighted are also the principal COSY correlations to hydrogen atoms within the same spin systems. **35**

Figure S5. ^{13}C-NMR spectrum (100 MHz, D_2O) sections of *N*-benzyl-3,6-dideoxy-3,6-imino-D-gulofuranose **4** with colour-coded signals, highlighting the furanose anomeric forms they belong to, with interpretation of the isolated signals and tentative interpretation of the overlapping ones. Namely, the orange designates the *α-fur* form and indigo designates the *β-fur*. A) section 143.0 ppm to 127.5 ppm; B) section 105.0 ppm to 76.0 ppm; C) section 75.0 ppm to 48.5 ppm. Highlighted are also the principal HSQC correlations to hydrogen atoms within the same spin systems. **37**

Figure S6. ^{1}H- (400 MHz), ^{13}C-NMR (100 MHz) and COSY spectra of *N*-benzyl-1,4-dideoxy-1,4-imino-L-gulitol **5** in D_2O. **40**

Figure S7. Sections of the ^{1}H-NMR spectrum (400 MHz, D_2O) of *N*-benzyl-1,4-dideoxy-1,4-imino-L-gulitol **5** with selected signals colour-coded. Namely, the orange and green colour-codes are used for overlapping signals to show the splitting pattern. The green and orange vertical lines designate the locations of the peaks for complex splitting patterns. A) section 4.59 ppm to 4.25 ppm; B) section 3.92 ppm to 3.20 ppm. Highlighted are also the principal COSY correlations between hydrogen atoms. **51**

Figure S8. ^{1}H- (400 MHz), ^{13}C-NMR (100 MHz), DEPT, ^{11}B-NMR (128 MHz), COSY, HSQC and HMBC spectra of *N*-(4-methylphenylboronic acid pinacol ester)-3,6-dideoxy-3,6-imino-1,2-*O*-isopropylidene-*α*-D-gulofuranose **para 6** in $CDCl_3$. **53**

Figure S9. ^{1}H- (400 MHz), ^{13}C-NMR (100 MHz), ^{11}B-NMR (128 MHz), COSY, HSQC and HMBC spectra of *N*-(4-methylphenyl boronic acid)-3,6-dideoxy-3,6-imino-D-gulofuranose **para 7** in D_2O. **72**

Figure S10. ^{1}H-NMR spectrum (400 MHz, D_2O) of *N*-(4-methylphenyl boronic acid)-3,6-dideoxy-3,6-imino-D-gulofuranose **para 7** with colour-coded signals, highlighting the furanose anomeric forms they belong to, with interpretation of the isolated signals and tentative interpretation of the overlapping ones. Namely, the orange designates the *α-fur* form and indigo designates the *β-fur*. Section 7.92 ppm to 7.55 ppm is visible with highlighted principal COSY correlations to hydrogen atoms within the same spin systems. **96**

Figure S11. ^{1}H-NMR spectrum (400 MHz, D_2O) of *N*-(4-methylphenyl boronic acid)-3,6-dideoxy-3,6-imino-D-gulofuranose **para 7** with colour-coded signals, highlighting the furanose anomeric forms they belong to, with interpretation of the isolated signals and tentative interpretation of the overlapping ones. Namely, the orange designates the *α-fur* form and indigo designates the *β-fur*. A) section 5.60 ppm to 4.90 ppm; B) section 4.75 ppm to 3.50 ppm. Highlighted are also the principal COSY correlations to hydrogen atoms within the same spin systems. **97**

Figure S12. ^{13}C-NMR spectrum (100 MHz, D_2O) sections of *N*-(4-methylphenyl boronic acid)-3,6-dideoxy-3,6-imino-D-gulofuranose **para 7** with colour-coded signals, highlighting the furanose anomeric forms they belong to, with interpretation of the isolated signals and tentative interpretation of the overlapping ones. Namely, the orange designates the *α-fur* form and indigo designates the *β-fur*. A) section 103.6 ppm to 97.5 ppm; B) section 81.0 ppm to 72.0 ppm; C) section 68.0 ppm to 56.5 ppm. Highlighted are also the principal HSQC correlations to hydrogen atoms in the same spin systems. **99**

Figure S13. ^{1}H- (400 MHz), ^{13}C-NMR (100 MHz), ^{11}B-NMR (128 MHz), COSY and HSQC spectra of *N*-(4-methylphenyl boronic acid)-1,4-dideoxy-1,4-imino-L-gulitol **para 8** in D_2O. **102**

Figure S14. ^{1}H-NMR spectrum (400 MHz, D_2O) of *N*-(4-methylphenyl boronic acid)-1,4-dideoxy-1,4-imino-L-gulitol **para 8** with colour-coded signals, highlighting the predominant boronic acid species (orange) and other boronic acid/boronate anion species (shades of green). Section 8.00 ppm to 6.80 ppm is visible with highlighted the principal COSY correlations to hydrogen atoms within the same spin systems. **118**

Figure S15. ^{1}H-NMR spectrum (400 MHz, D_2O) of *N*-(4-methylphenyl boronic acid)-1,4-dideoxy-1,4-imino-L-gulitol **para 8** with colour-coded signals, highlighting the predominant boronic acid species (orange) and other boronic acid/boronate anion species (shades of green). A) section 5.10 ppm to 4.00 ppm; B) section 4.10 ppm to 3.10 ppm. Highlighted are also the principal COSY correlations to hydrogen atoms within the same spin systems. **119**

Figure S16. ^{13}C-NMR spectrum (100 MHz, D_2O) sections of *N*-(4-methylphenyl boronic acid)-1,4-dideoxy-1,4-imino-L-gulitol **para 8**. Only the main set of signals is presented A) section 136.5 ppm to 115.0 ppm; B) section 75.0 ppm to 50.0 ppm. **121**

NMR Experimental details **123**

References **135**

Table S1. Structures of the reference compounds analysed by ^{11}B-NMR and their corresponding signals at 128 MHz, dissolved in the deuterated solvent stated in brackets.

Structure	Chemical shift, ppm	Reference	Structure	Chemical shift, ppm	Ref
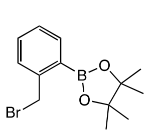 ortho 9	31 (D_2O)	Inventory (Boron Molecular)[1]	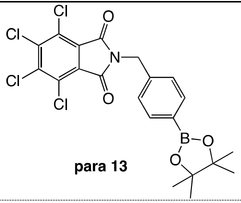 para 13	31.1 ($CDCl_3$)	[2]
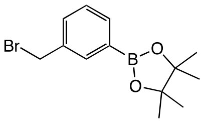 meta 9	31 (D_2O)	Inventory (Boron Molecular)[1]	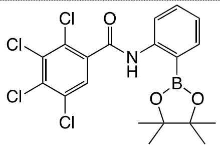 ortho 14	31.1 ($CDCl_3$)	[2]
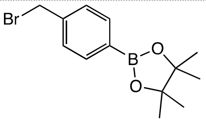 para 9	31 (D_2O)	Inventory (Boron Molecular)[1]	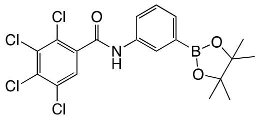 meta 14	31.7 ($CDCl_3$)	[2]
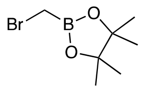 10	Rapidly decomposed (D_2O)	Inventory (Boron Molecular)[1]	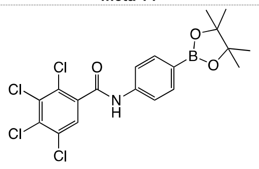 para 14	31.8 ($CDCl_3$)	[2]
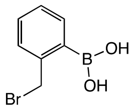 ortho 11	30 (D_2O)	Inventory (Boron Molecular)[1]	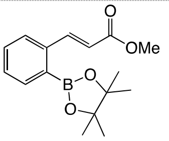 ortho 15	31.5 ($CDCl_3$)	[3]
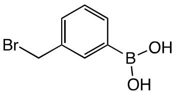 meta 11	29 (D_2O)	Inventory (Boron Molecular)[1]	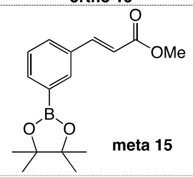 meta 15	30.7 ($CDCl_3$)	[3]
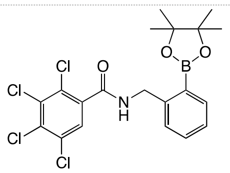 ortho 12	31.8 ($CDCl_3$)	[2]	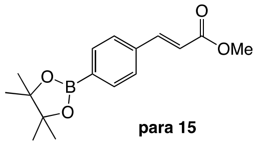 para 15	31.2 ($CDCl_3$)	[3]
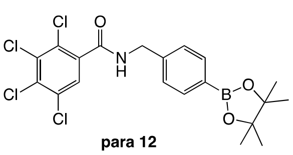 para 12	31.6 ($CDCl_3$)	[2]	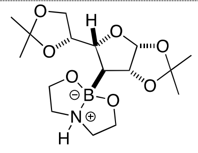 16 D-*Galacto*	30.9, 17.2, 10.3 (integration ratio: 0.02 : 0.03 : 1.0) (CD_3OD)	[4,5]
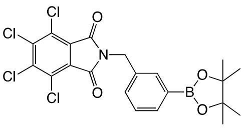 meta 13	31.5 ($CDCl_3$)	[2]	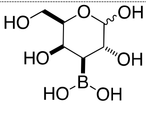 17 D-*Galacto*	31.7, 19.4 (integration ratio: 4.4 : 1.0) (D_2O)	[4,5]

Figure S1. ^{1}H- (400 MHz), ^{13}C-NMR (100 MHz), DEPT, COSY and HSQC spectra of *N*-benzyl-3,6-dideoxy-3,6-imino-1,2-*O*-isopropylidene-α-D-gulofuranose **3** in $CDCl_3$.

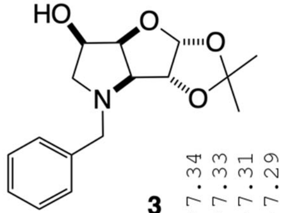

3

^{1}**H-NMR**

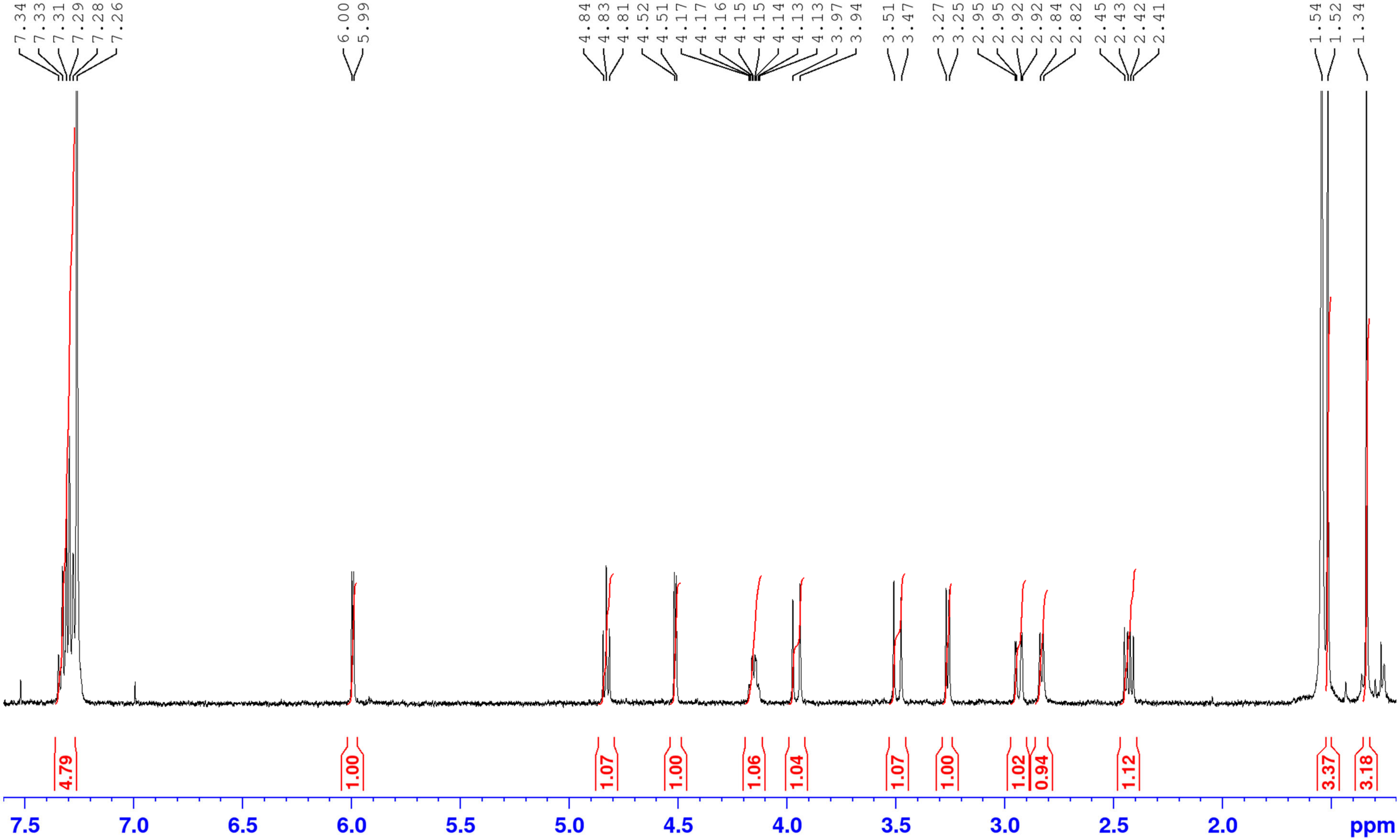

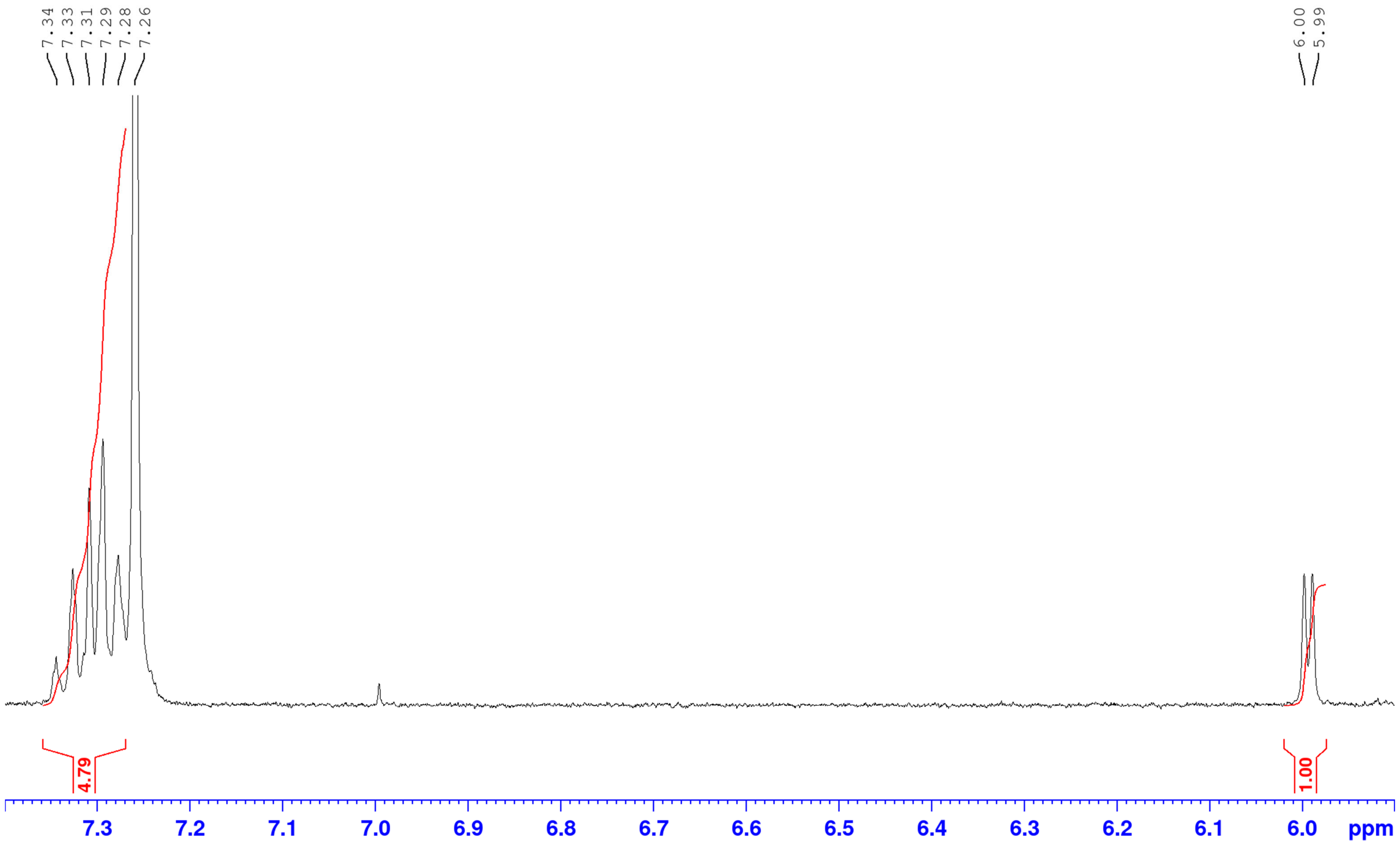
7.34
7.33
7.31
7.29
7.28
7.26
6.00
5.99
4.79
1.00
7.3
7.2
7.1
7.0
6.9
6.8
6.7
6.6
6.5
6.4
6.3
6.2
6.1
6.0
ppm

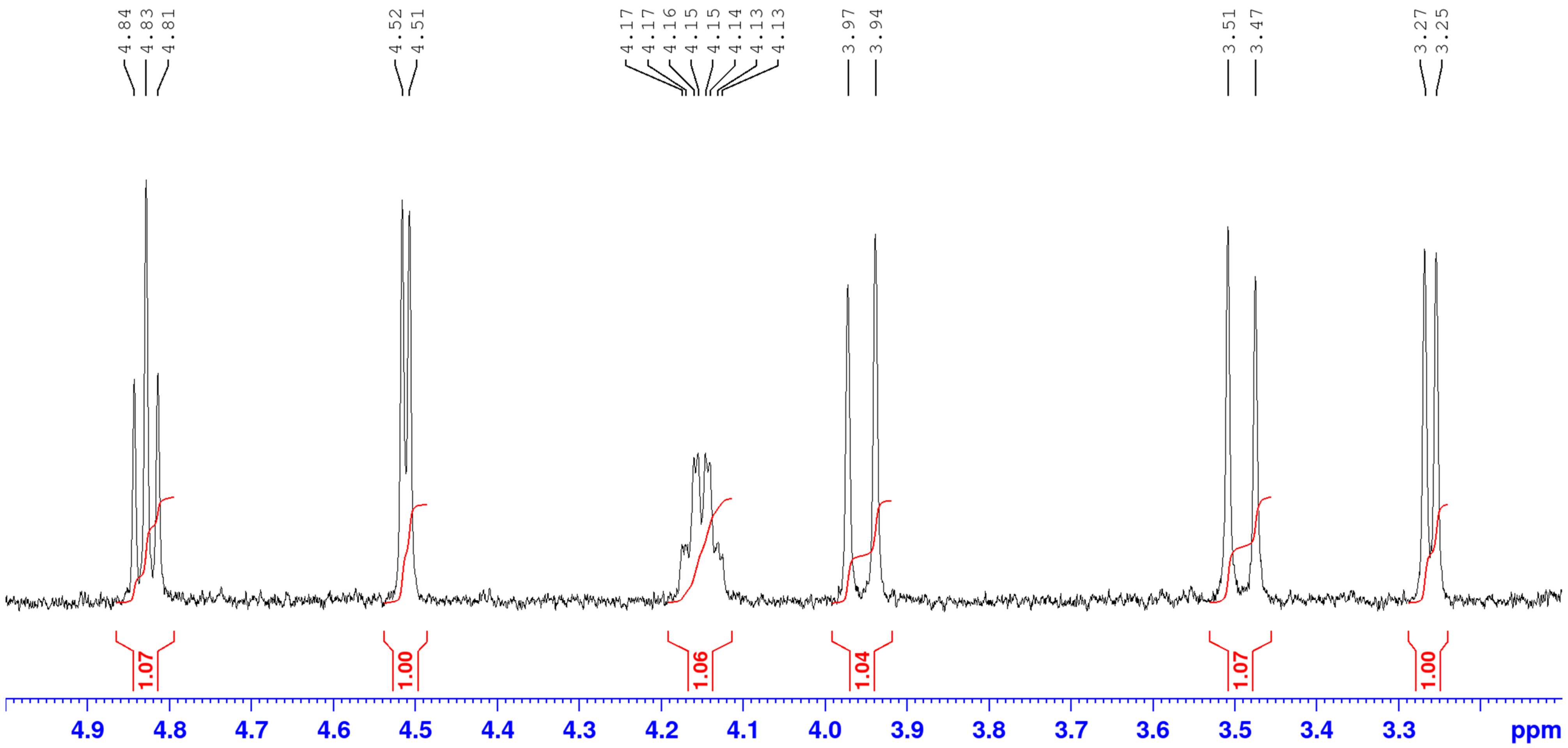
4.84
4.83
4.81
4.52
4.51
4.17
4.17
4.16
4.15
4.15
4.14
4.13
4.13
3.97
3.94
3.51
3.47
3.27
3.25
1.07
1.00
1.06
1.04
1.07
1.00
4.9
4.8
4.7
4.6
4.5
4.4
4.3
4.2
4.1
4.0
3.9
3.8
3.7
3.6
3.5
3.4
3.3
ppm

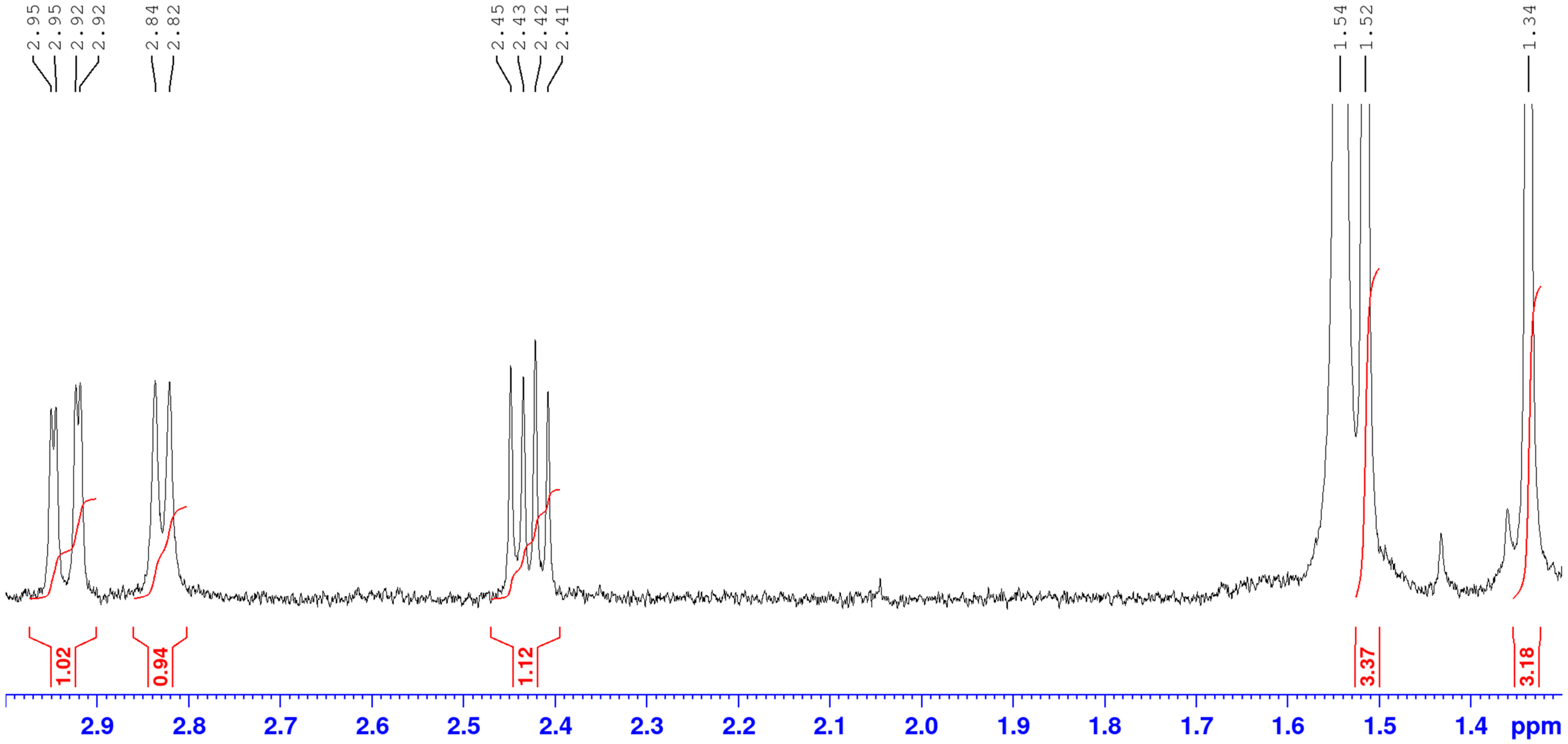

2.95
2.95
2.92
2.92
2.84
2.82
2.45
2.43
2.42
2.41
1.54
1.52
1.34
1.02
0.94
1.12
3.37
3.18
2.9
2.8
2.7
2.6
2.5
2.4
2.3
2.2
2.1
2.0
1.9
1.8
1.7
1.6
1.5
1.4
ppm

^{13}C-NMR

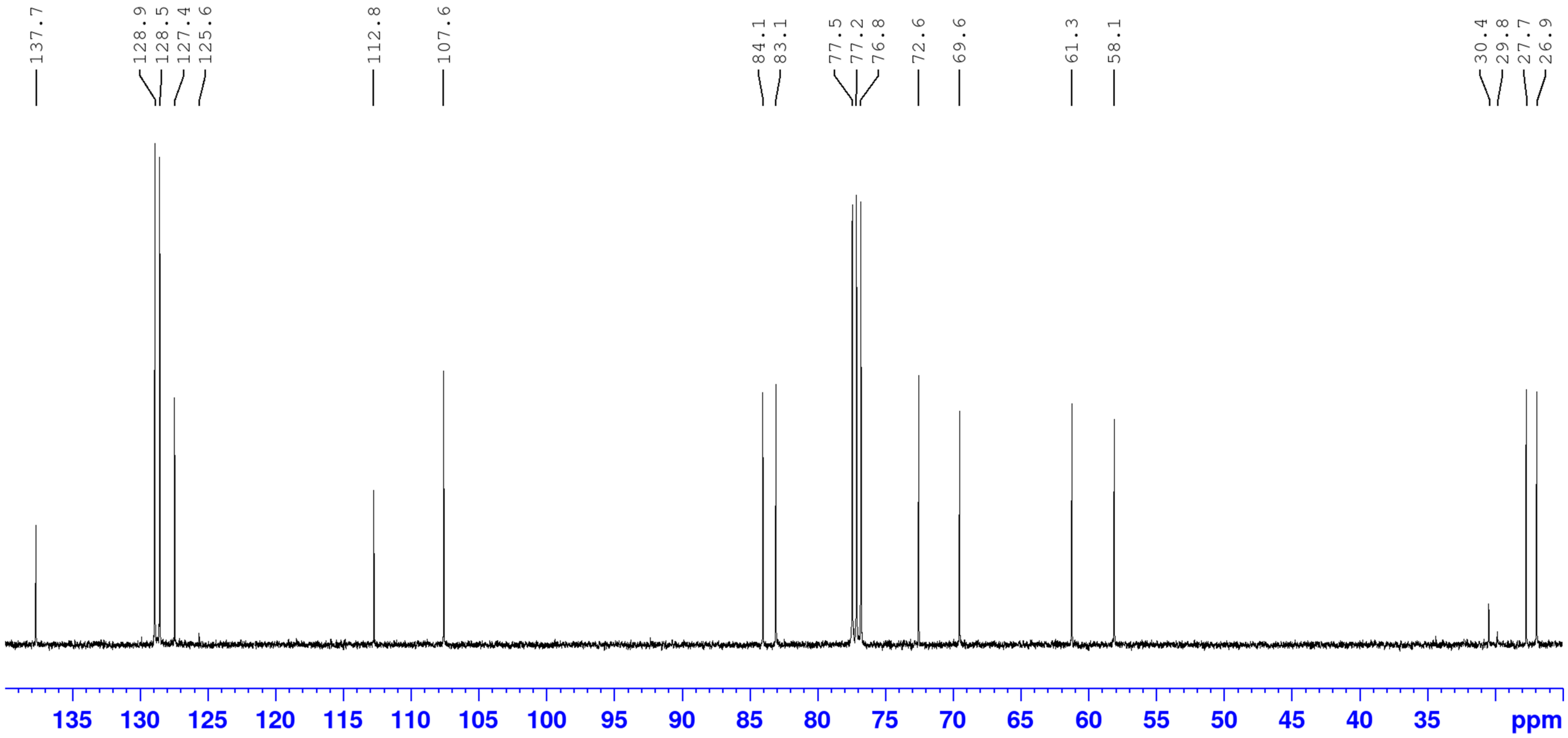
137.7
128.9
128.5
127.4
125.6
112.8
107.6
84.1
83.1
77.5
77.2
76.8
72.6
69.6
61.3
58.1
30.4
29.8
27.7
26.9
135 130 125 120 115 110 105 100 95 90 85 80 75 70 65 60 55 50 45 40 35 ppm

DEPT

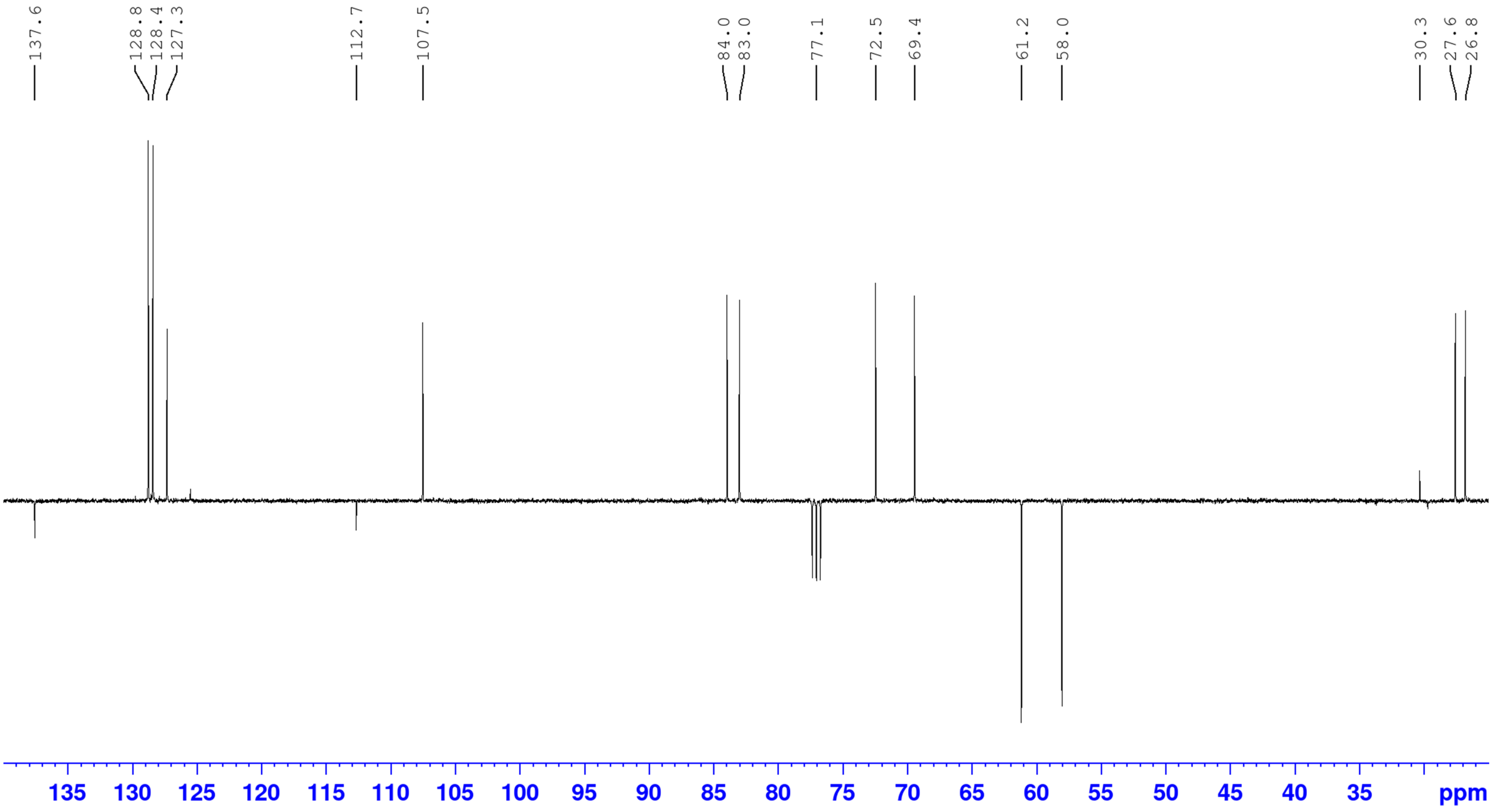
137.6
128.8
128.4
127.3
112.7
107.5
84.0
83.0
77.1
72.5
69.4
61.2
58.0
30.3
27.6
26.8
135
130
125
120
115
110
105
100
95
90
85
80
75
70
65
60
55
50
45
40
35
ppm

COSY

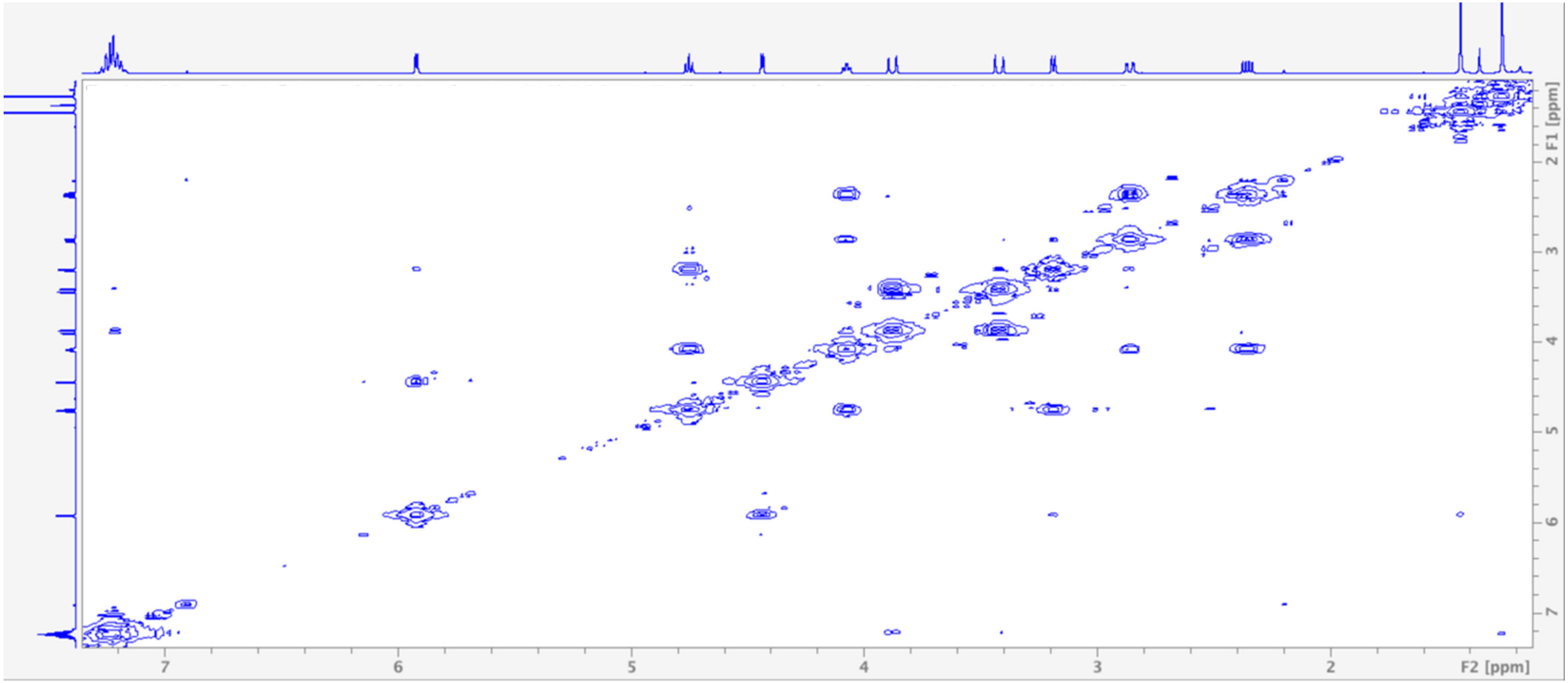

HSQC

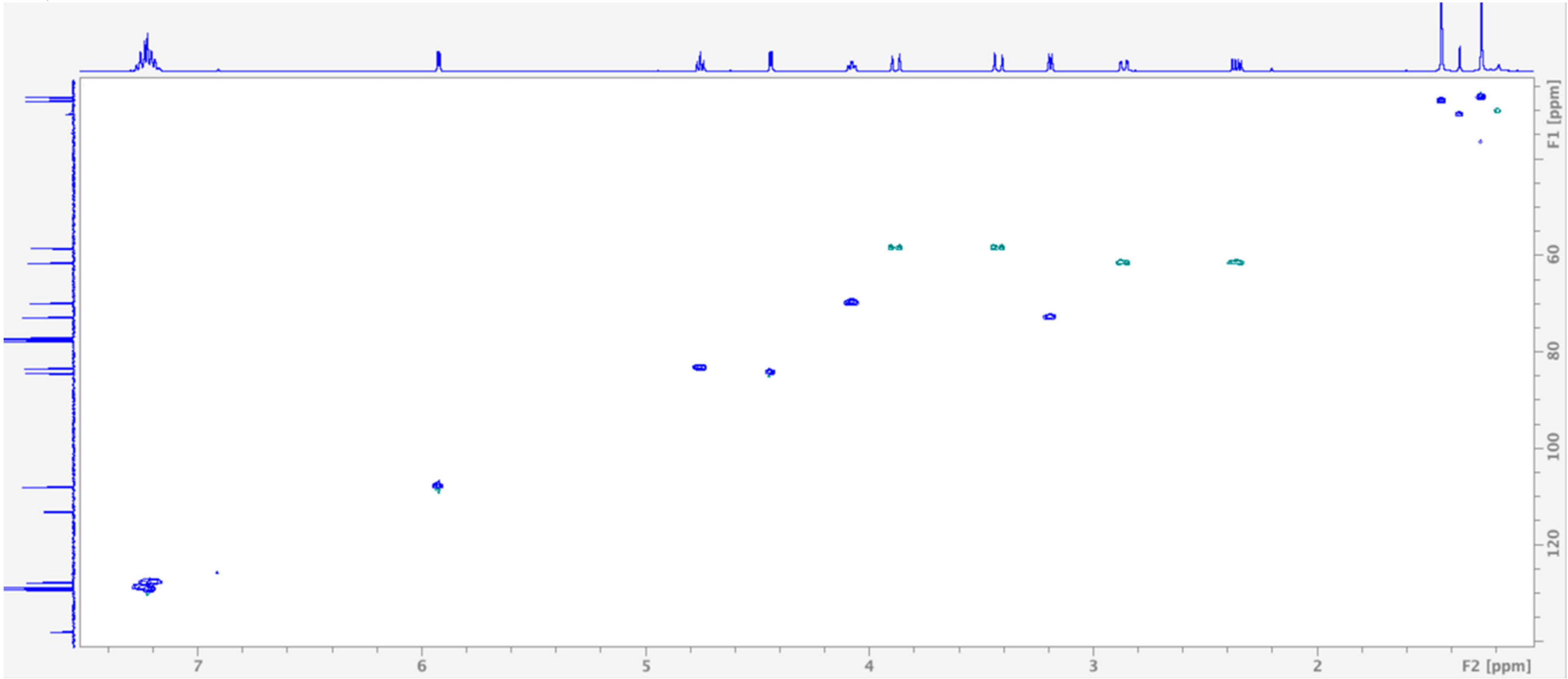

Figure S2. ^1H- (400 MHz), ^{13}C-NMR (100 MHz), DEPT, COSY, HSQC and HMBC spectra of *N*-benzyl-3,6-dideoxy-3,6-imino-D-gulofuranose **4** in D_2O.

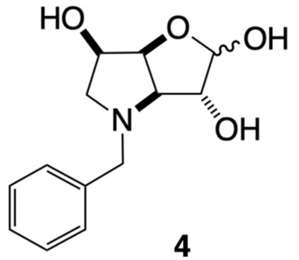

4

^{1}H-NMR

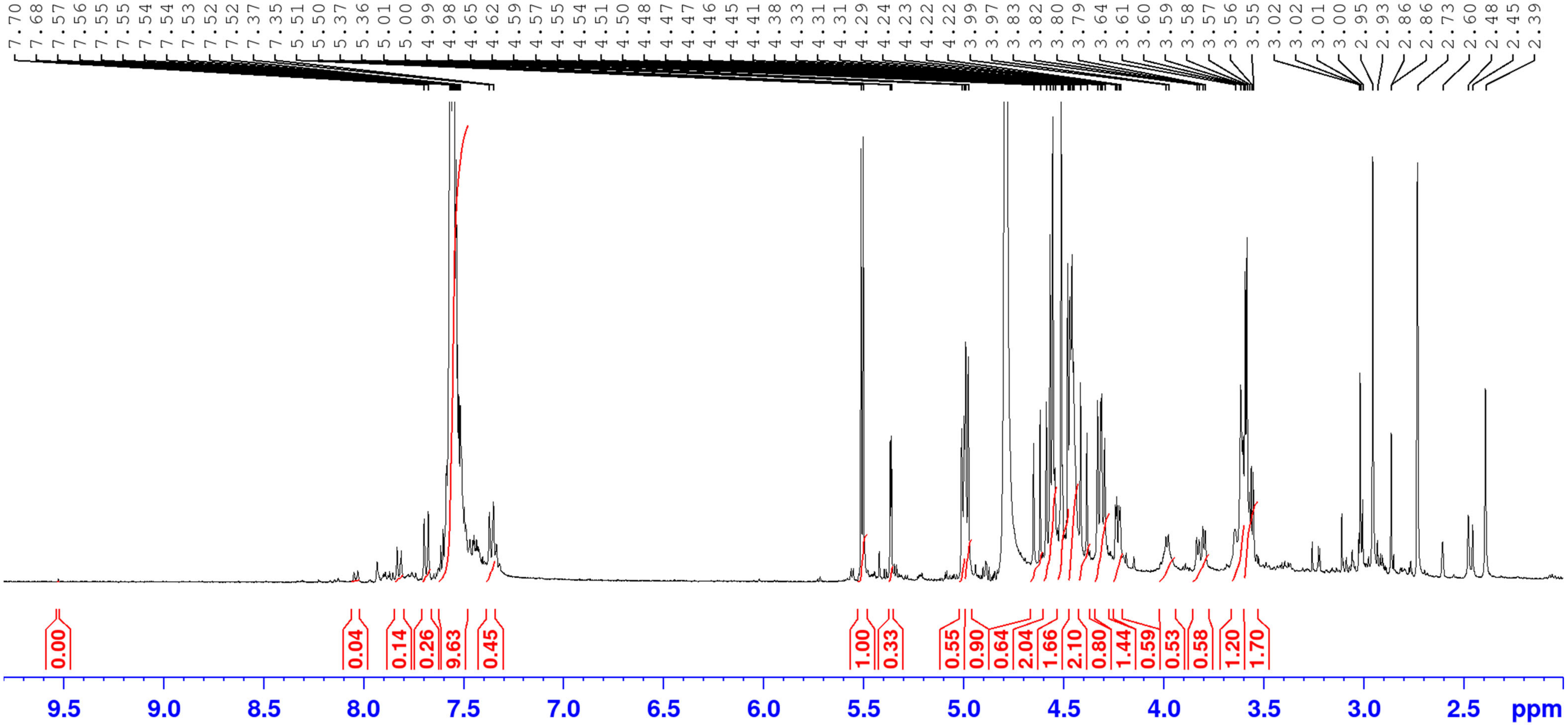

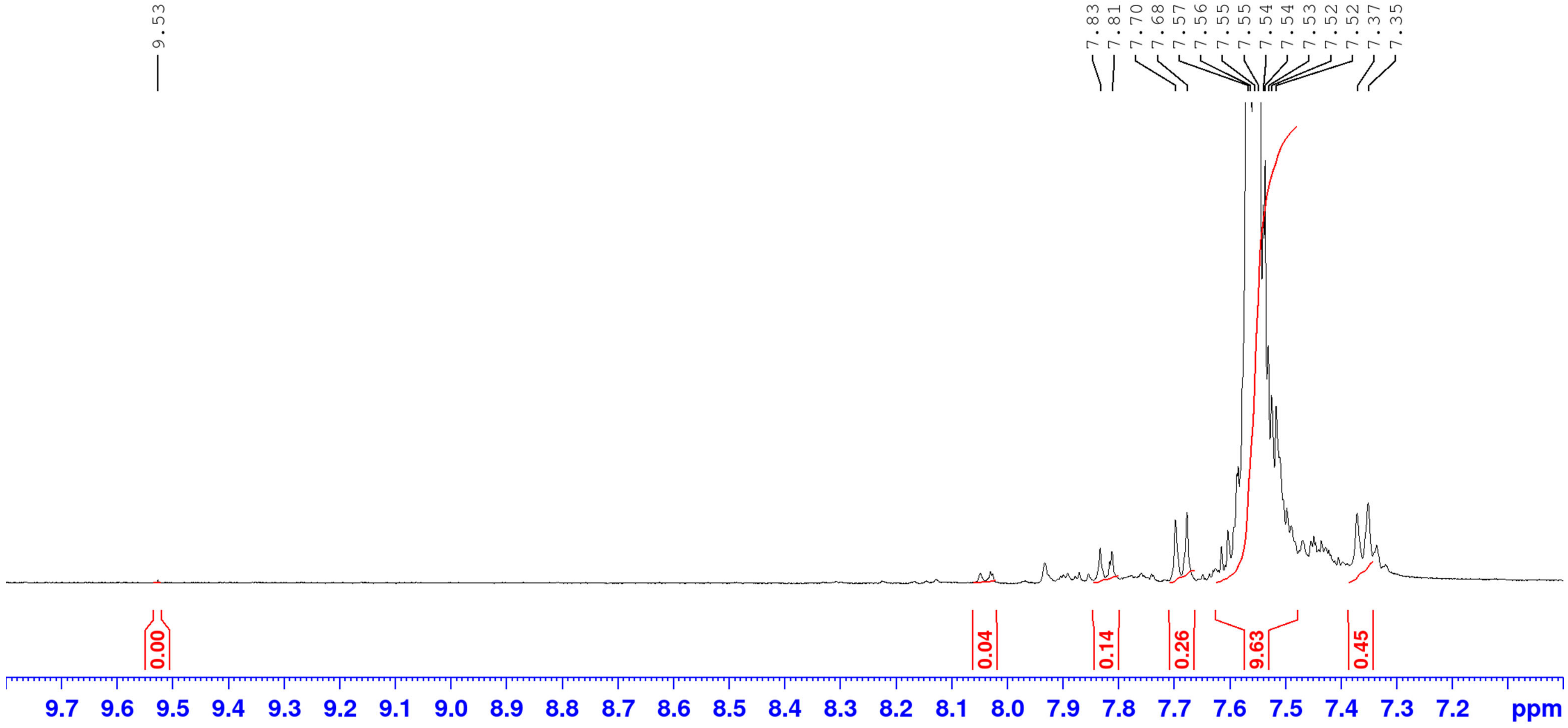
9.53
7.83
7.81
7.70
7.68
7.57
7.56
7.55
7.55
7.54
7.54
7.53
7.52
7.52
7.37
7.35
0.00
0.04
0.14
0.26
9.63
0.45
9.7 9.6 9.5 9.4 9.3 9.2 9.1 9.0 8.9 8.8 8.7 8.6 8.5 8.4 8.3 8.2 8.1 8.0 7.9 7.8 7.7 7.6 7.5 7.4 7.3 7.2
ppm

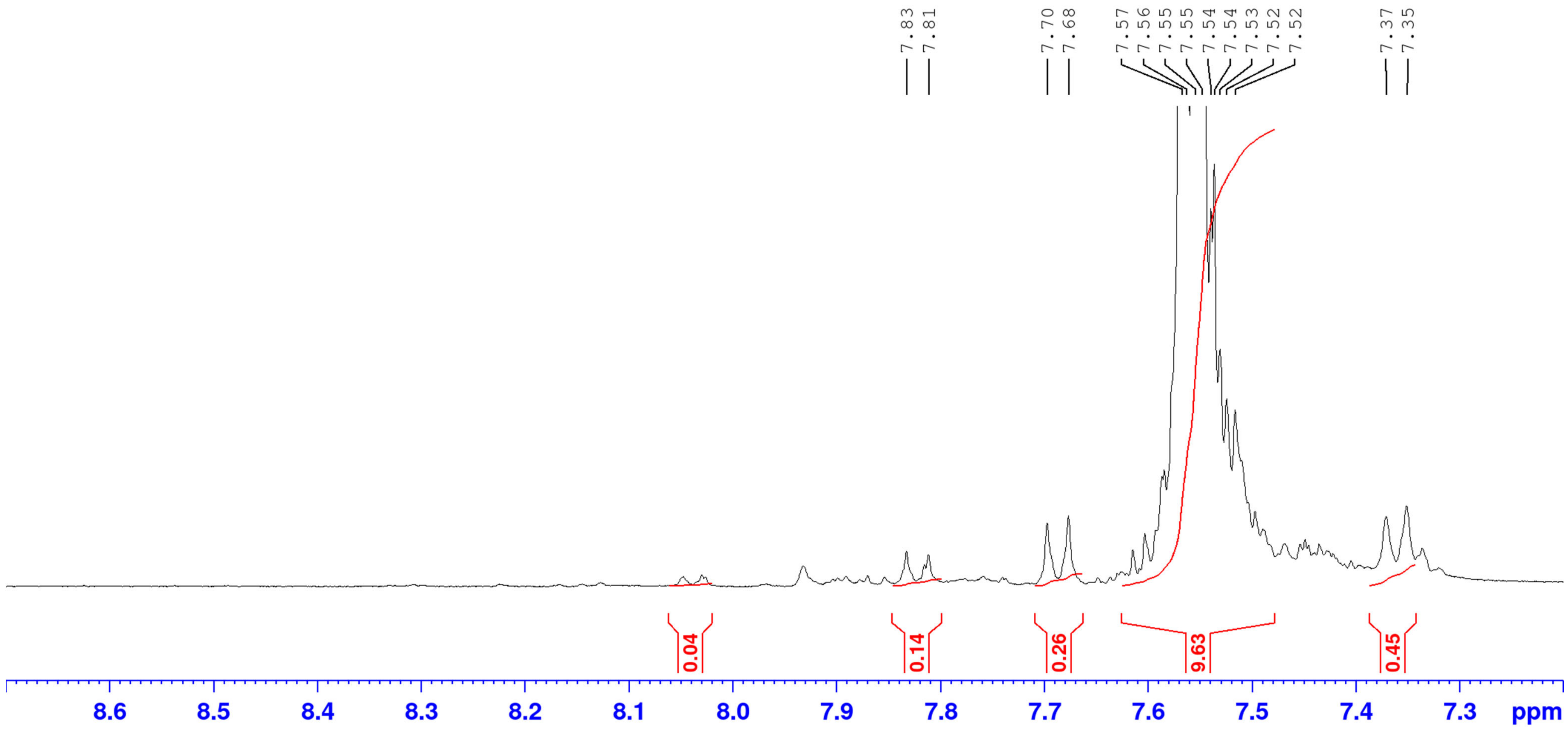
7.83
7.81
7.70
7.68
7.57
7.56
7.55
7.55
7.54
7.54
7.53
7.52
7.52
7.37
7.35
0.04
0.14
0.26
9.63
0.45
8.6
8.5
8.4
8.3
8.2
8.1
8.0
7.9
7.8
7.7
7.6
7.5
7.4
7.3
ppm

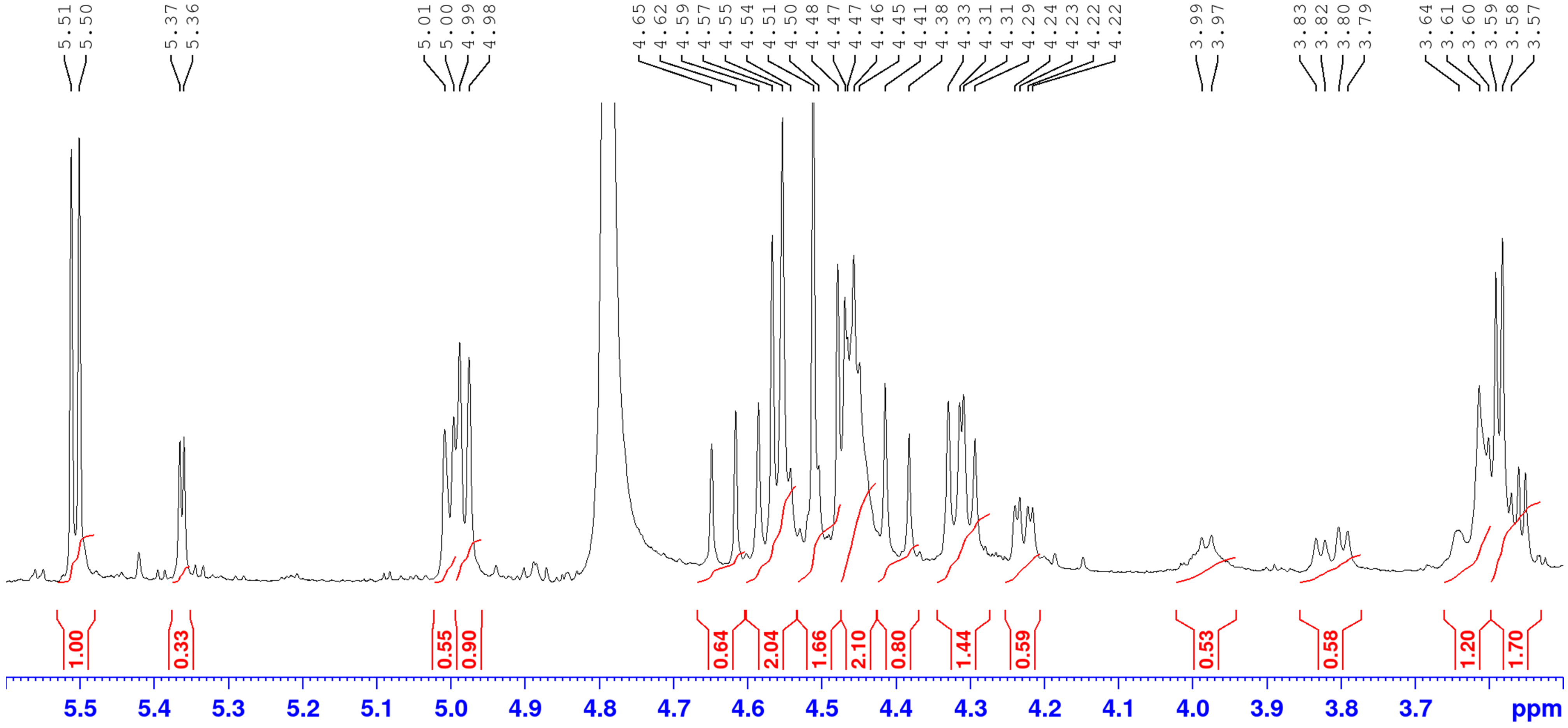
5.51
5.50
5.37
5.36
5.01
5.00
4.99
4.98
4.65
4.62
4.59
4.57
4.55
4.54
4.51
4.50
4.48
4.47
4.47
4.46
4.45
4.41
4.38
4.33
4.31
4.31
4.29
4.24
4.23
4.22
4.22
3.99
3.97
3.83
3.82
3.80
3.79
3.64
3.61
3.60
3.59
3.58
3.57
1.00
0.33
0.55
0.90
0.64
2.04
1.66
2.10
0.80
1.44
0.59
0.53
0.58
1.20
1.70
5.5
5.4
5.3
5.2
5.1
5.0
4.9
4.8
4.7
4.6
4.5
4.4
4.3
4.2
4.1
4.0
3.9
3.8
3.7
ppm

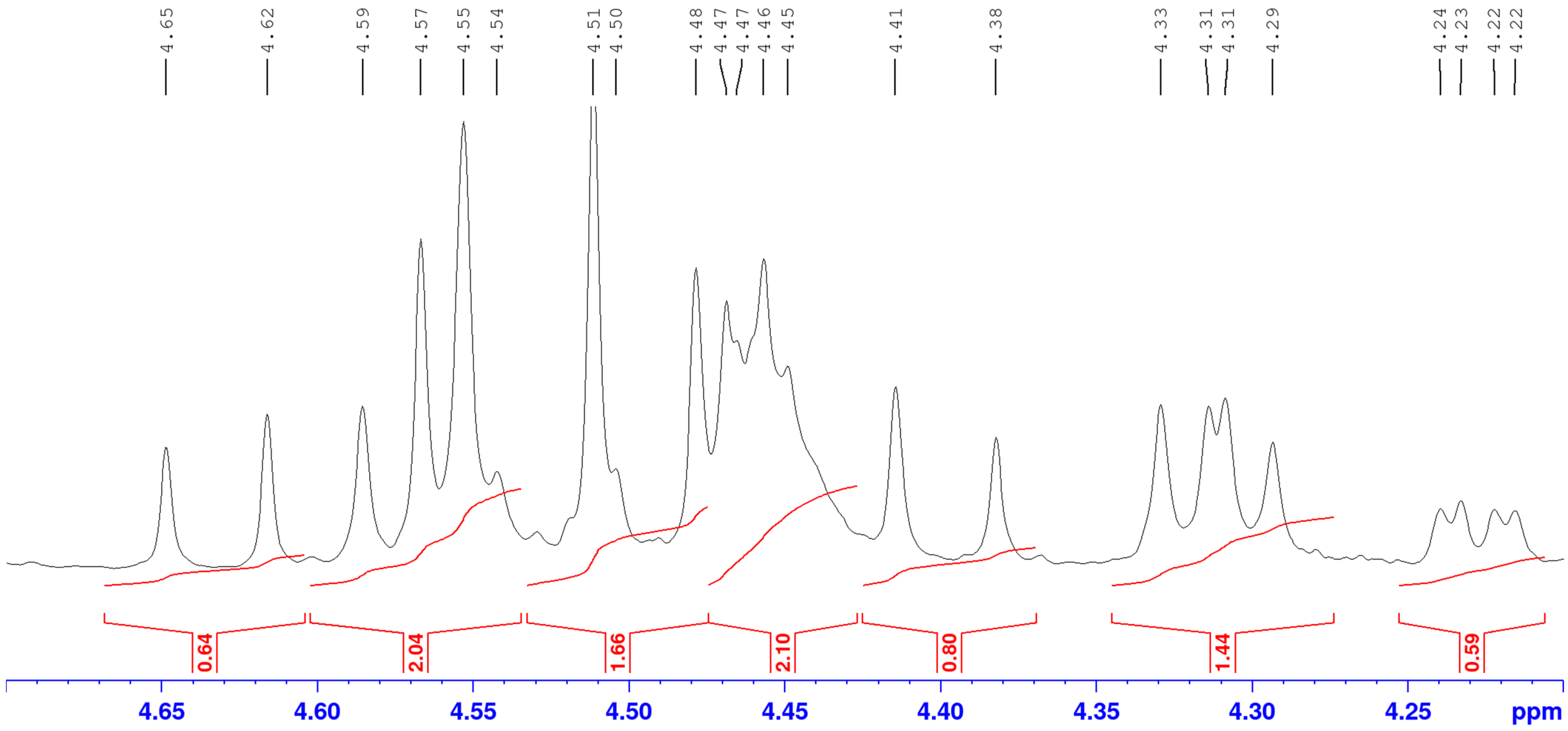
4.65
4.62
4.59
4.57
4.55
4.54
4.51
4.50
4.48
4.47
4.47
4.46
4.45
4.41
4.38
4.33
4.31
4.31
4.29
4.24
4.23
4.22
4.22
0.64
2.04
1.66
2.10
0.80
1.44
0.59
4.65
4.60
4.55
4.50
4.45
4.40
4.35
4.30
4.25
ppm

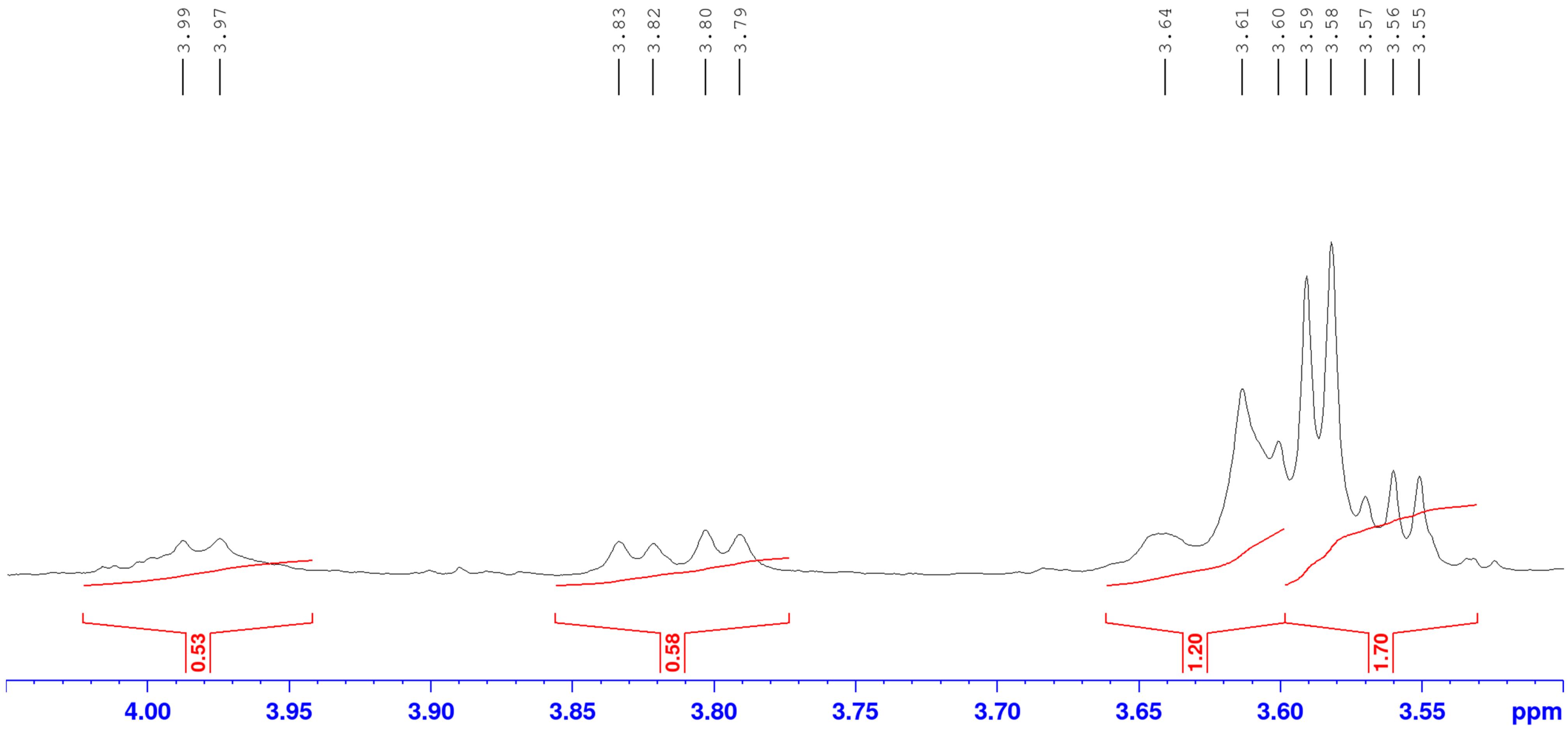
3.99
3.97
3.83
3.82
3.80
3.79
3.64
3.61
3.60
3.59
3.58
3.57
3.56
3.55
0.53
0.58
1.20
1.70
4.00
3.95
3.90
3.85
3.80
3.75
3.70
3.65
3.60
3.55
ppm

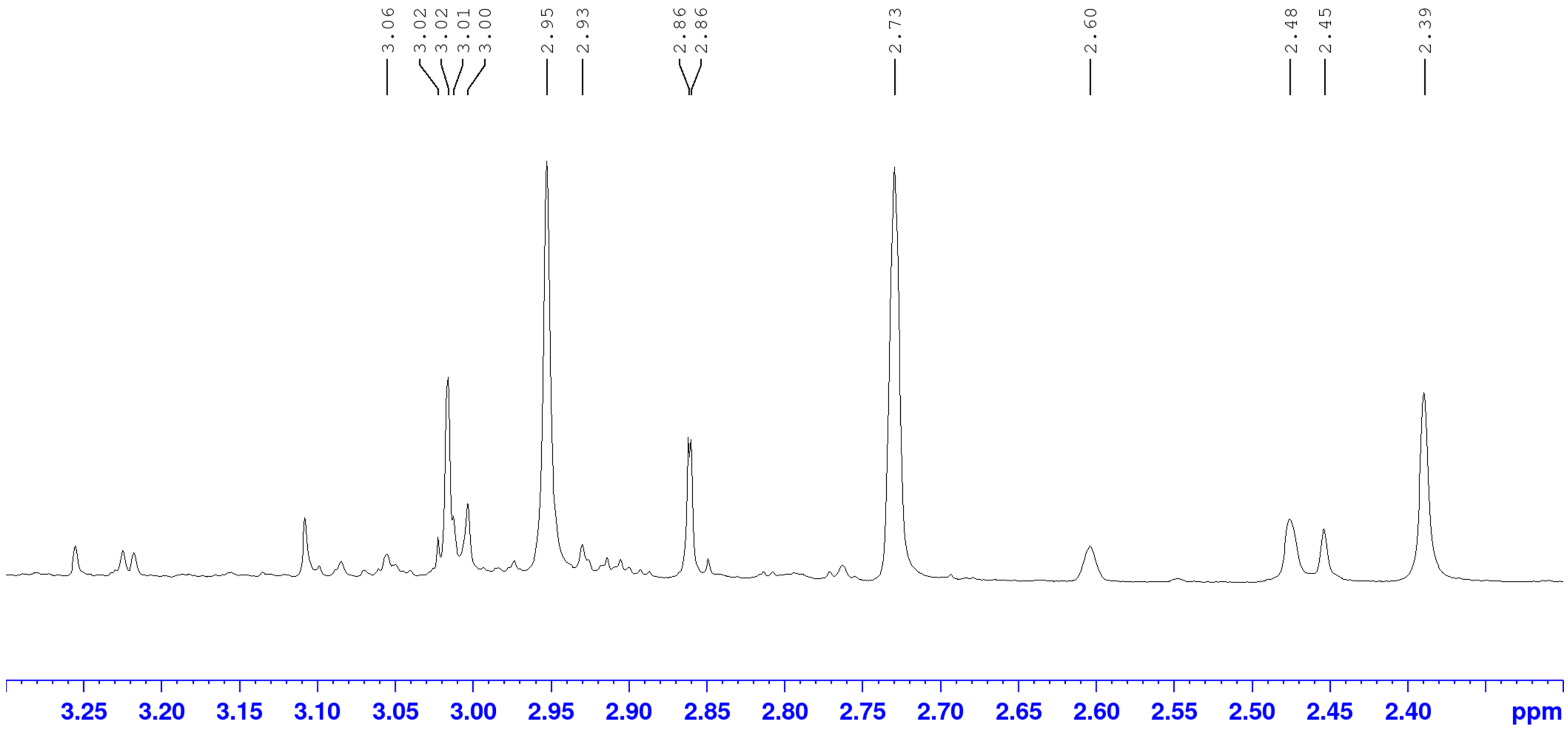
3.06
3.02
3.02
3.01
3.00
2.95
2.93
2.86
2.86
2.73
2.60
2.48
2.45
2.39
3.25
3.20
3.15
3.10
3.05
3.00
2.95
2.90
2.85
2.80
2.75
2.70
2.65
2.60
2.55
2.50
2.45
2.40
ppm

^{13}C-NMR

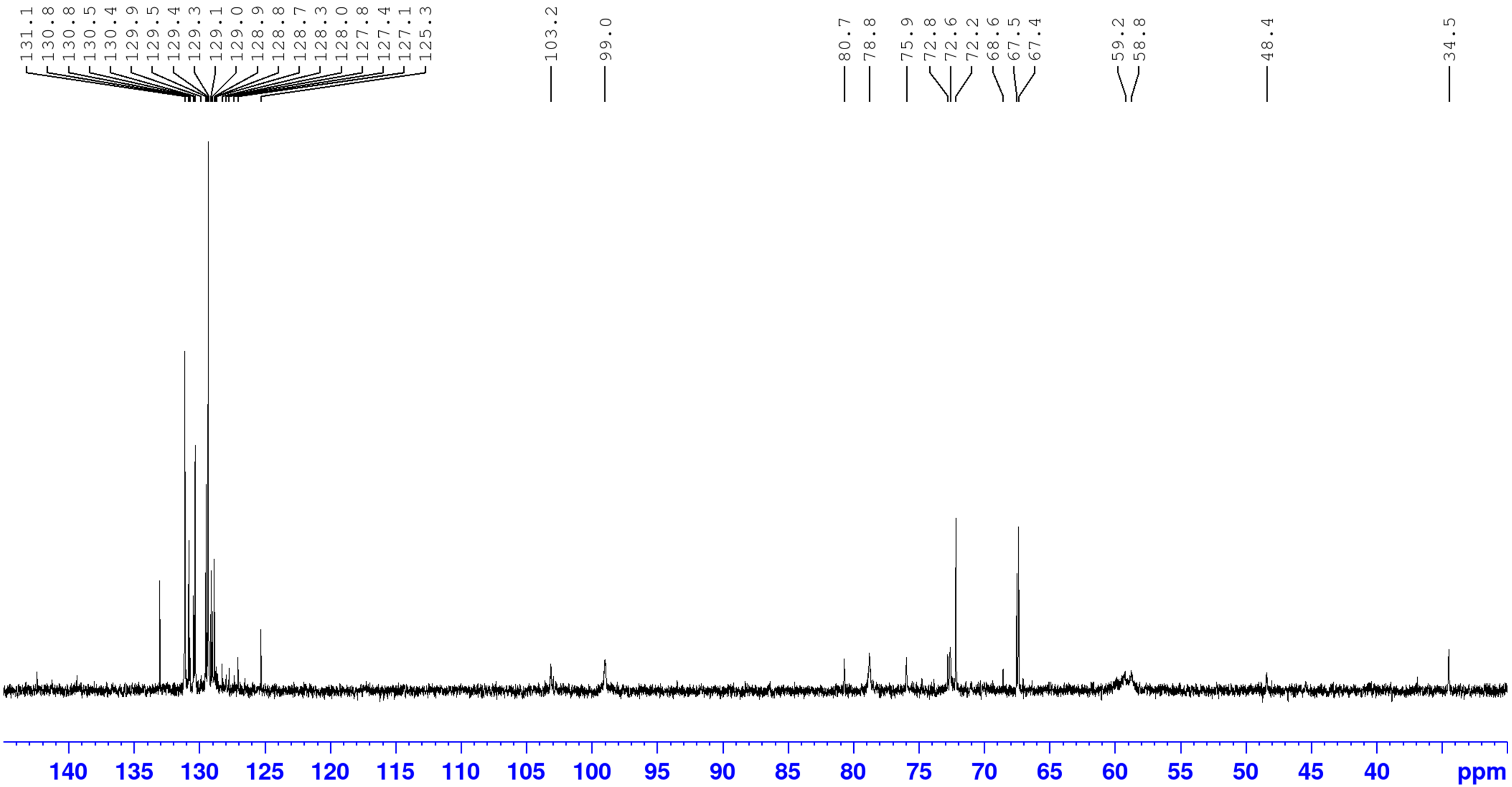
131.1
130.8
130.8
130.5
130.4
129.9
129.5
129.4
129.3
129.1
129.0
128.9
128.8
128.7
128.3
128.0
127.8
127.4
127.1
125.3
103.2
99.0
80.7
78.8
75.9
72.8
72.6
72.2
68.6
67.5
67.4
59.2
58.8
48.4
34.5
140 135 130 125 120 115 110 105 100 95 90 85 80 75 70 65 60 55 50 45 40 ppm

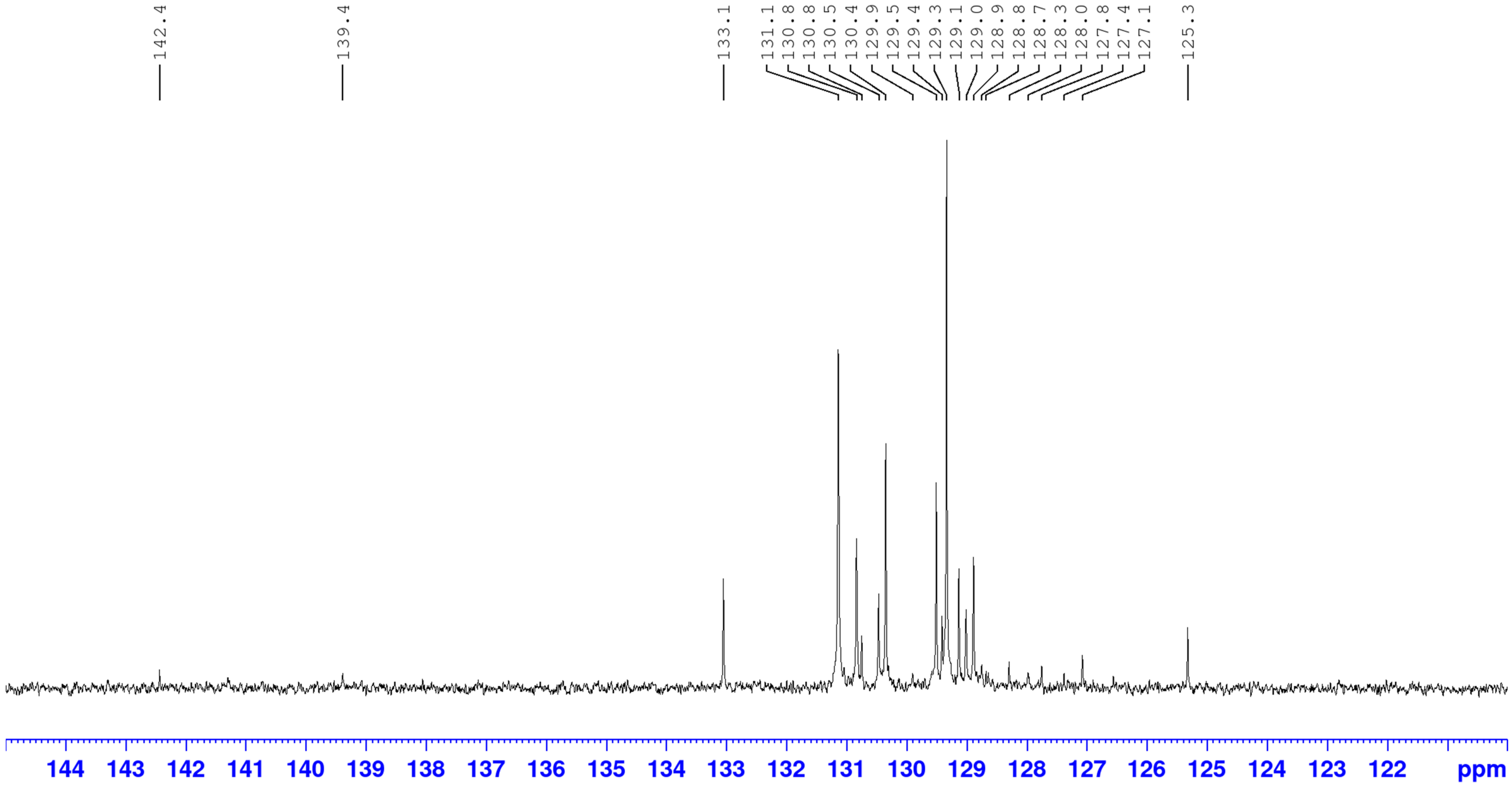
142.4
139.4
133.1
131.1
130.8
130.8
130.5
130.4
129.9
129.5
129.4
129.3
129.1
129.0
128.9
128.8
128.7
128.3
128.0
127.8
127.4
127.1
125.3
144 143 142 141 140 139 138 137 136 135 134 133 132 131 130 129 128 127 126 125 124 123 122 ppm

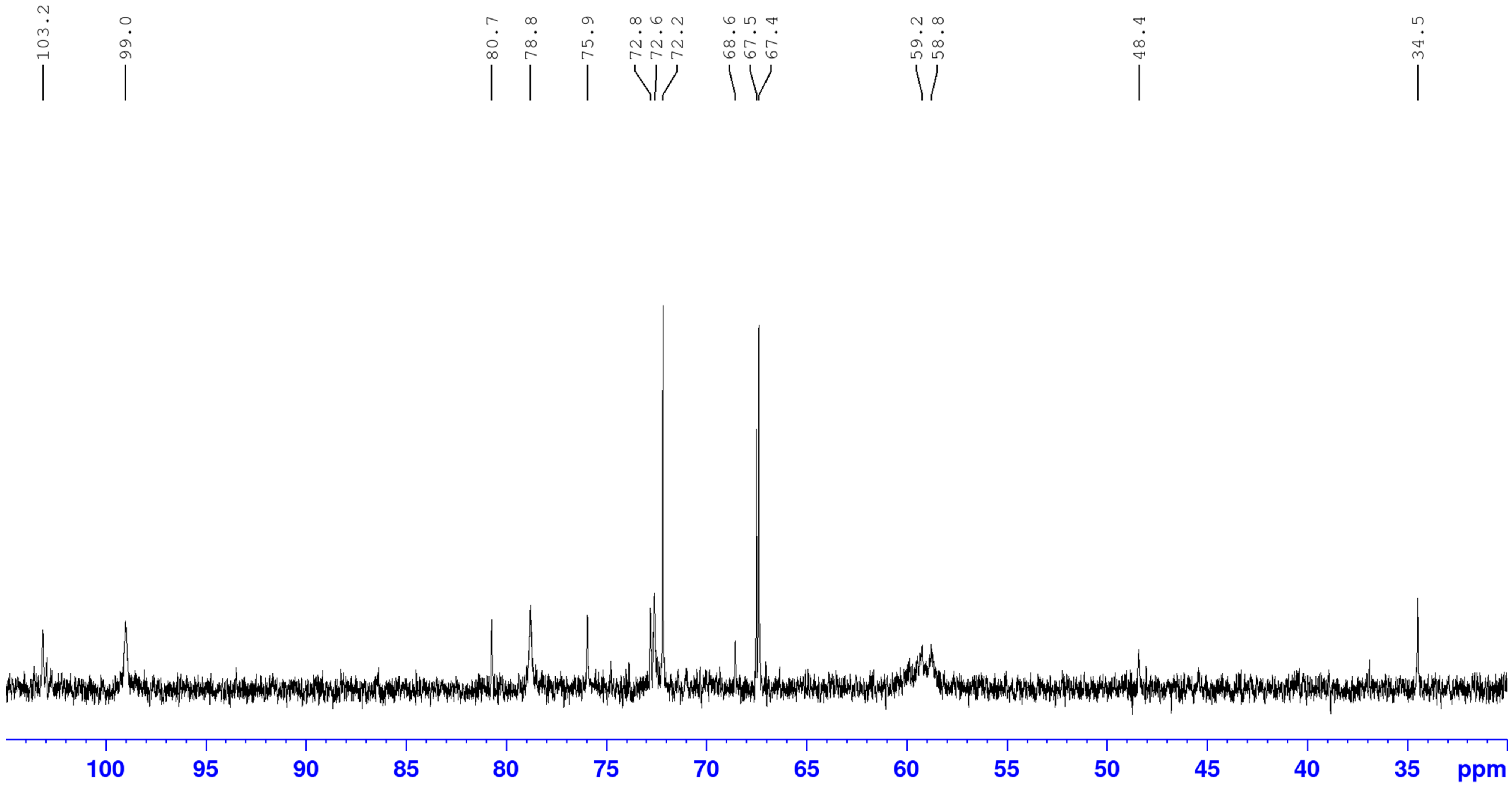
103.2
99.0
80.7
78.8
75.9
72.8
72.6
72.2
68.6
67.5
67.4
59.2
58.8
48.4
34.5
100
95
90
85
80
75
70
65
60
55
50
45
40
35
ppm

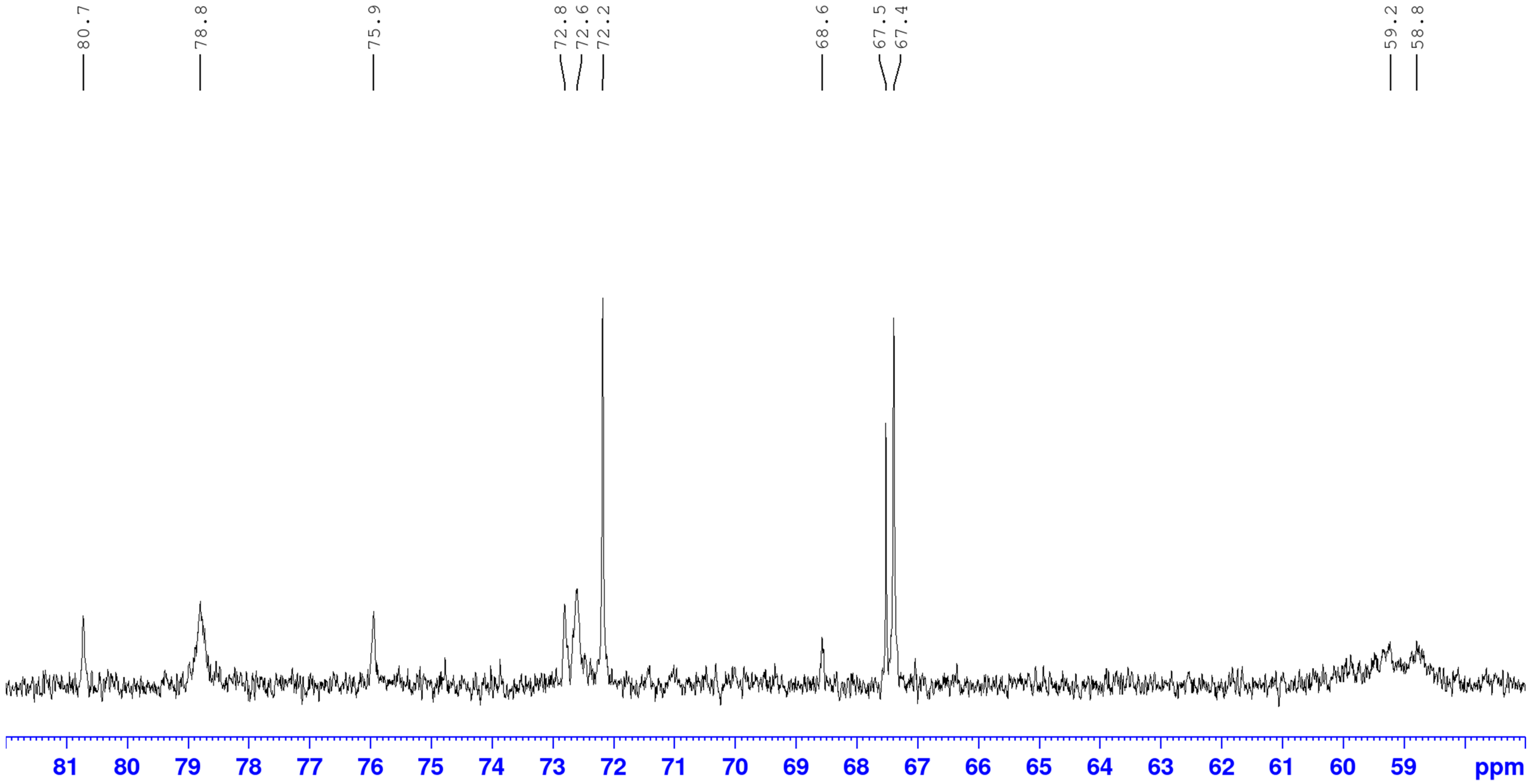
80.7
78.8
75.9
72.8
72.6
72.2
68.6
67.5
67.4
59.2
58.8
81
80
79
78
77
76
75
74
73
72
71
70
69
68
67
66
65
64
63
62
61
60
59
ppm

COSY

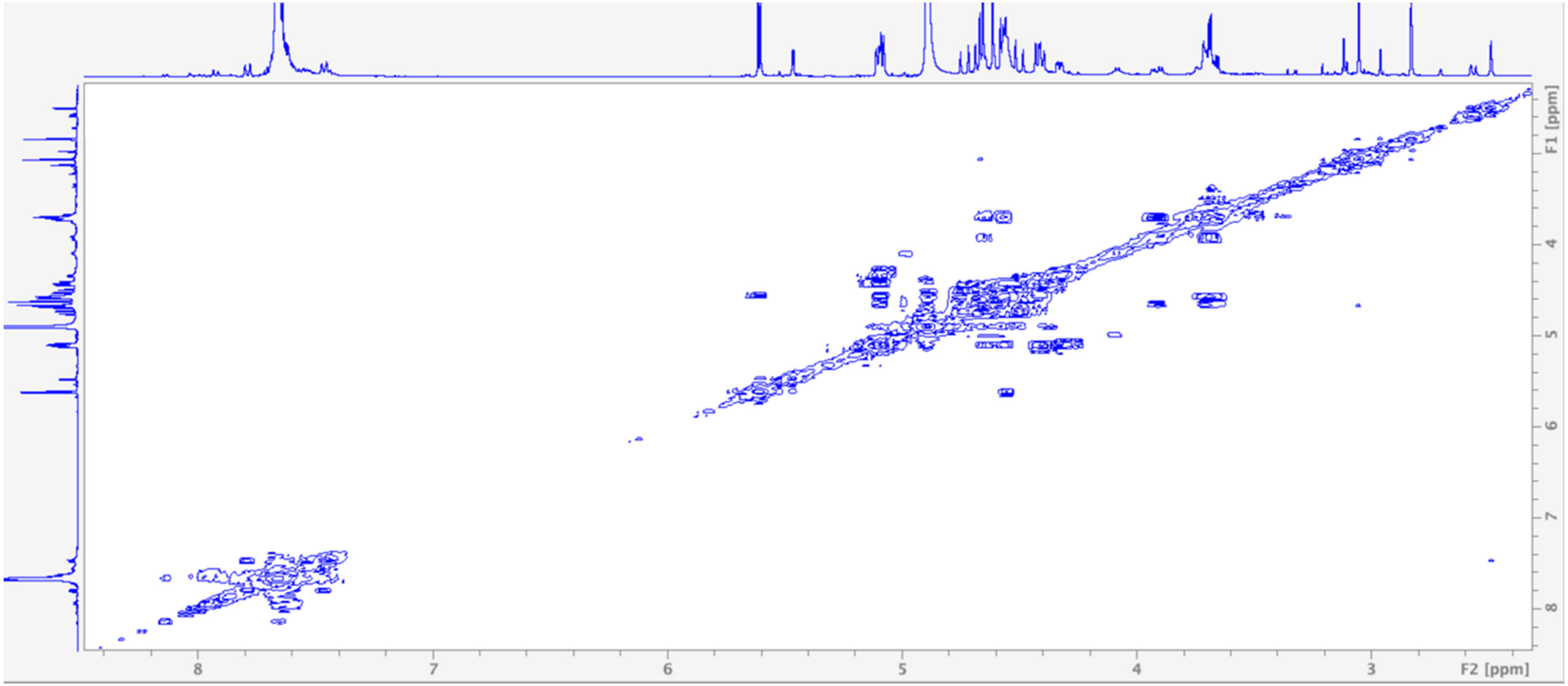

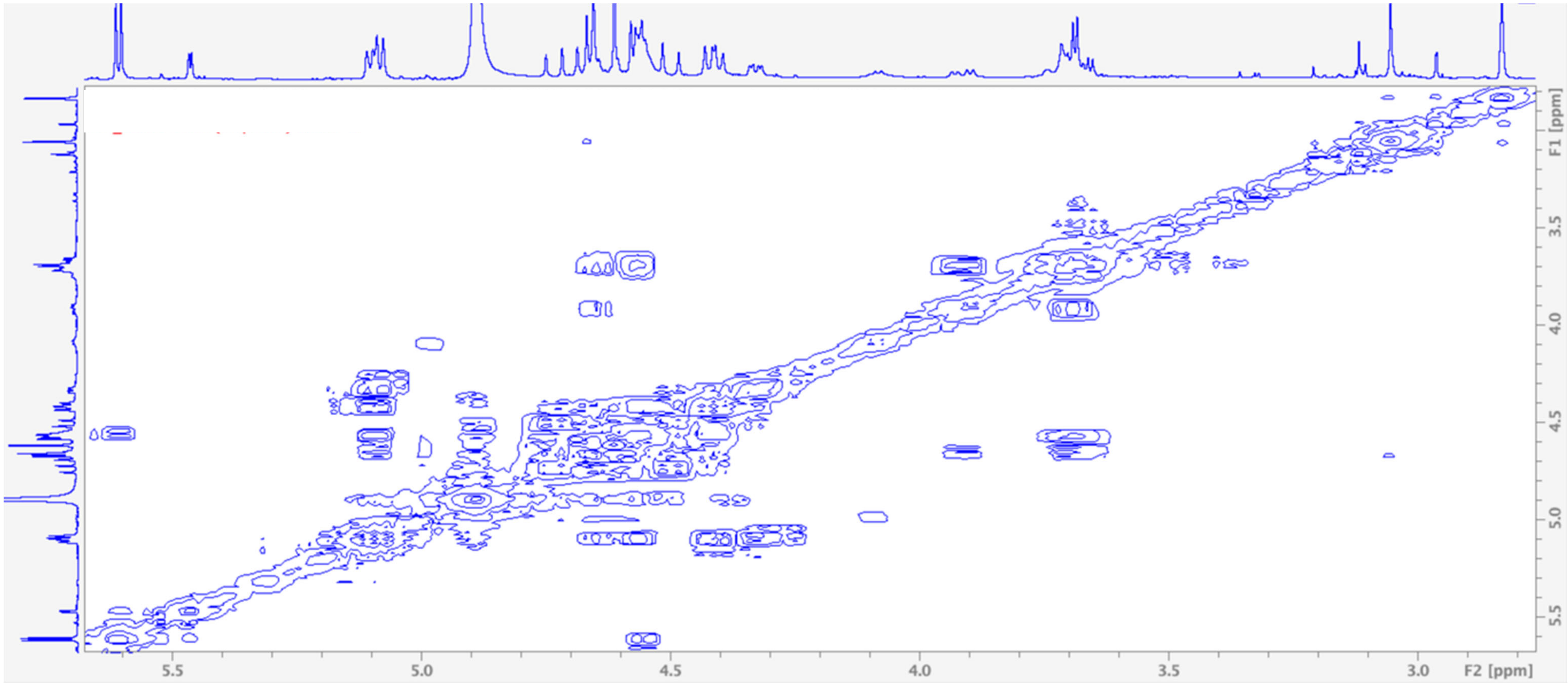
F1 [ppm]
3.5
4.0
4.5
5.0
5.5
F2 [ppm]

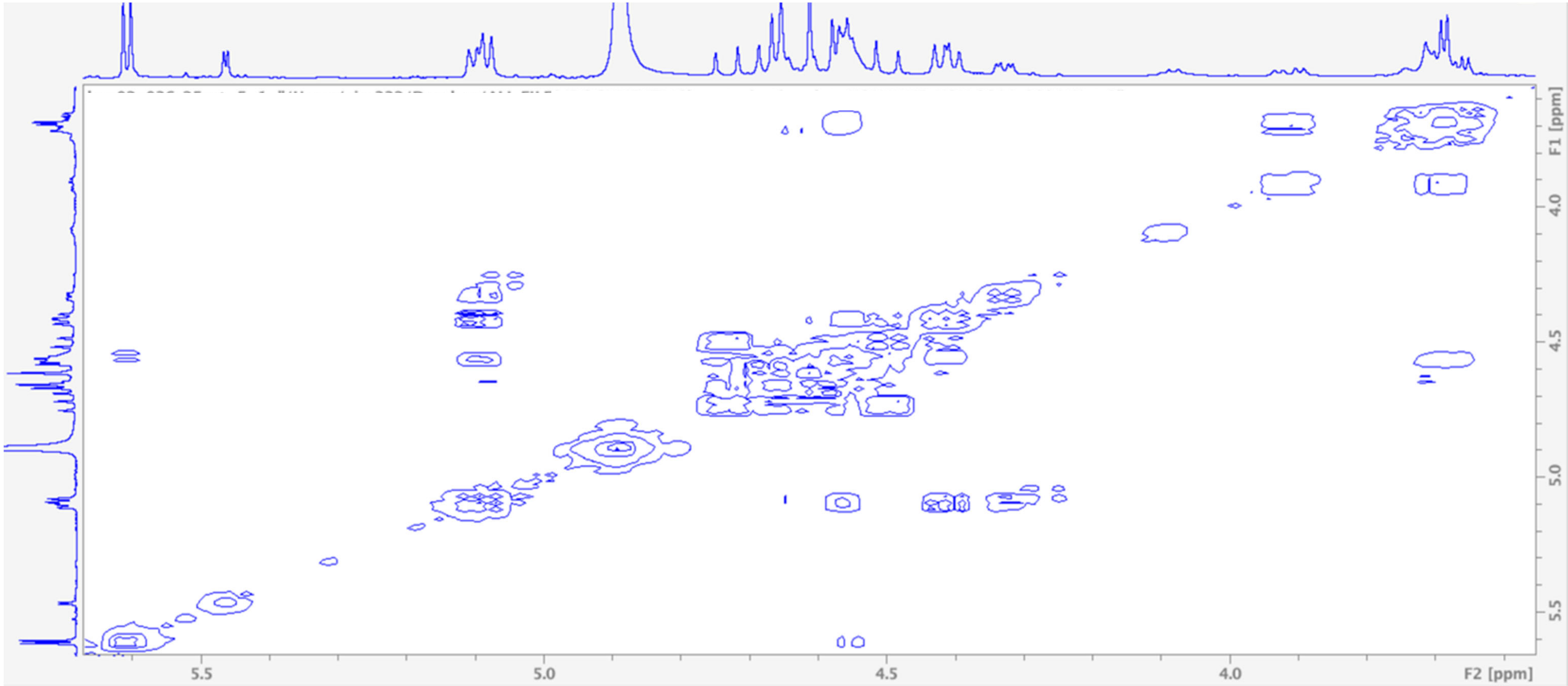
F1 [ppm]
4.0
4.5
5.0
5.5
5.5
5.0
4.5
4.0
F2 [ppm]

HSQC

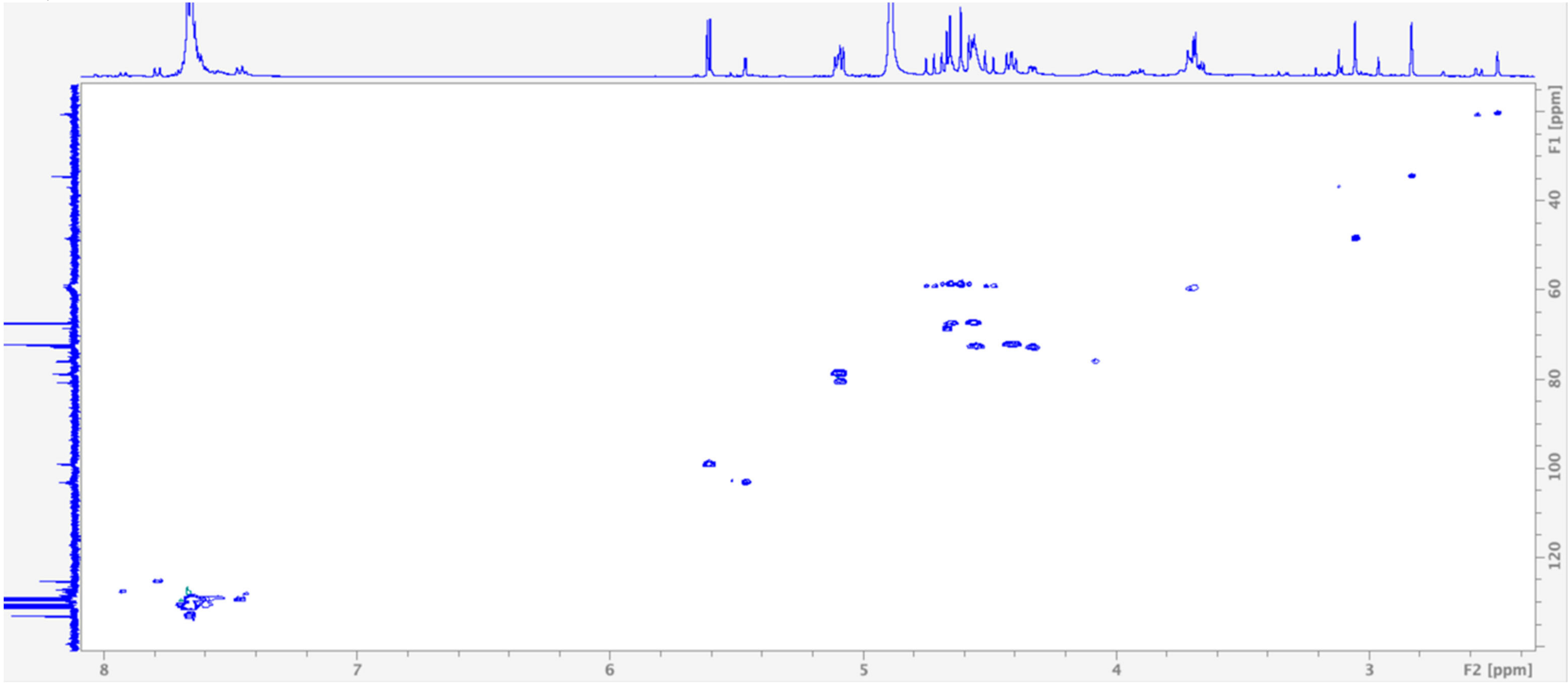

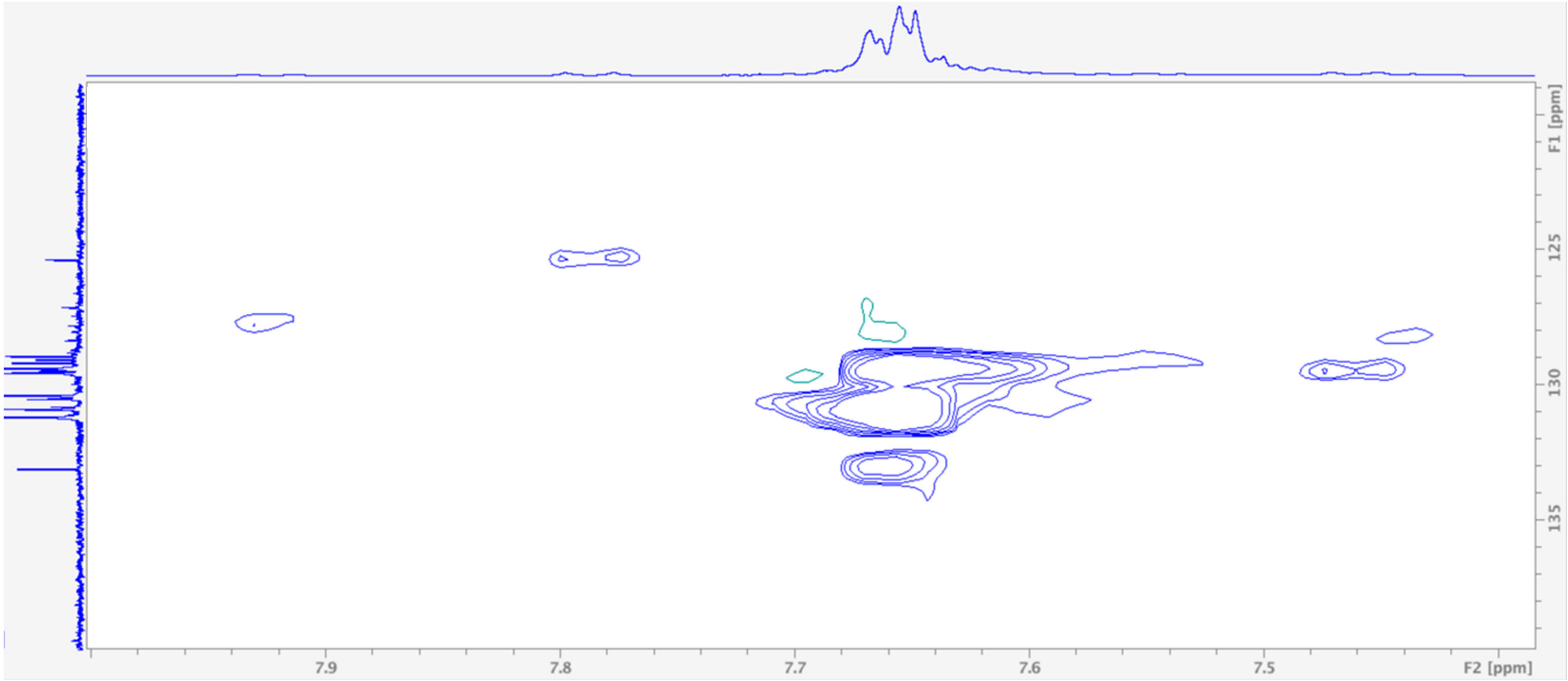
F1 [ppm]
125
130
135
7.9
7.8
7.7
7.6
7.5
F2 [ppm]

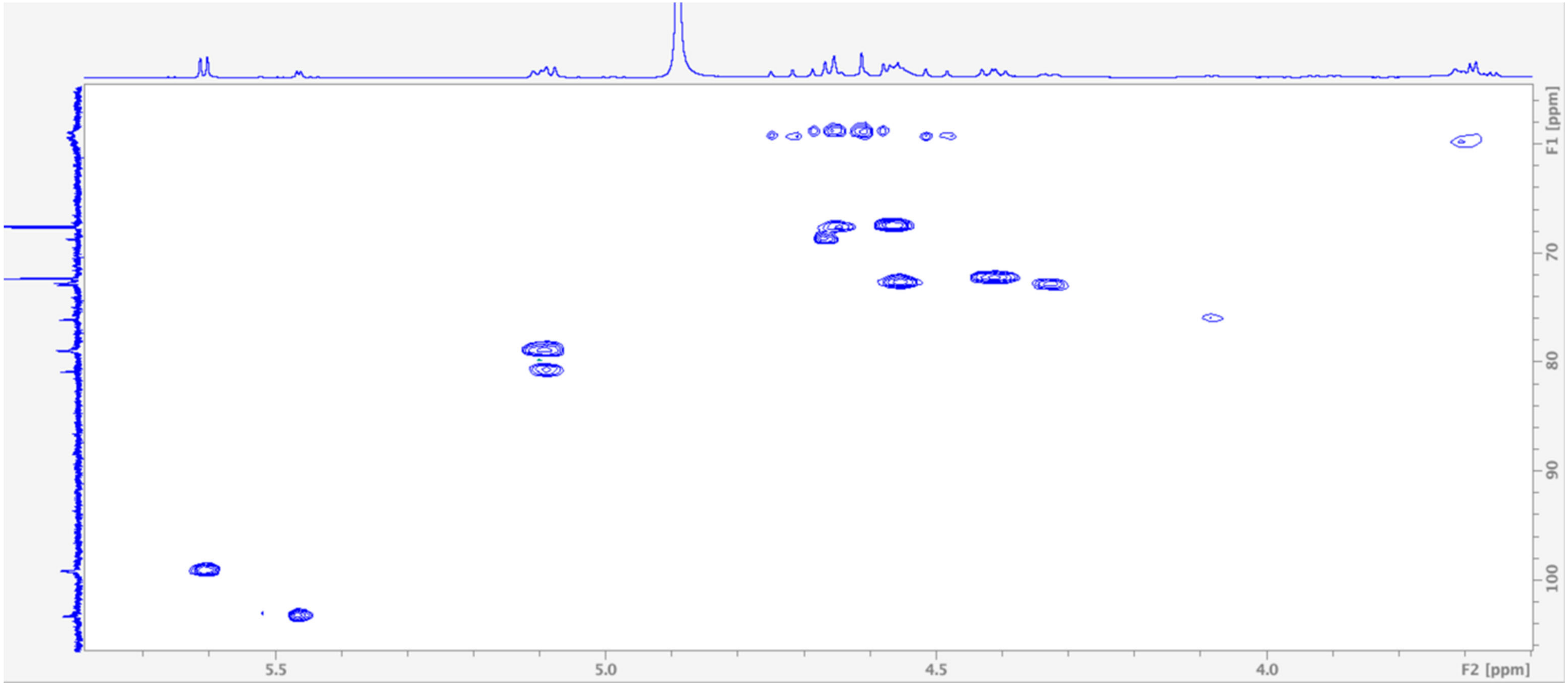
F1 [ppm]
70
80
90
100
5.5
5.0
4.5
4.0
F2 [ppm]

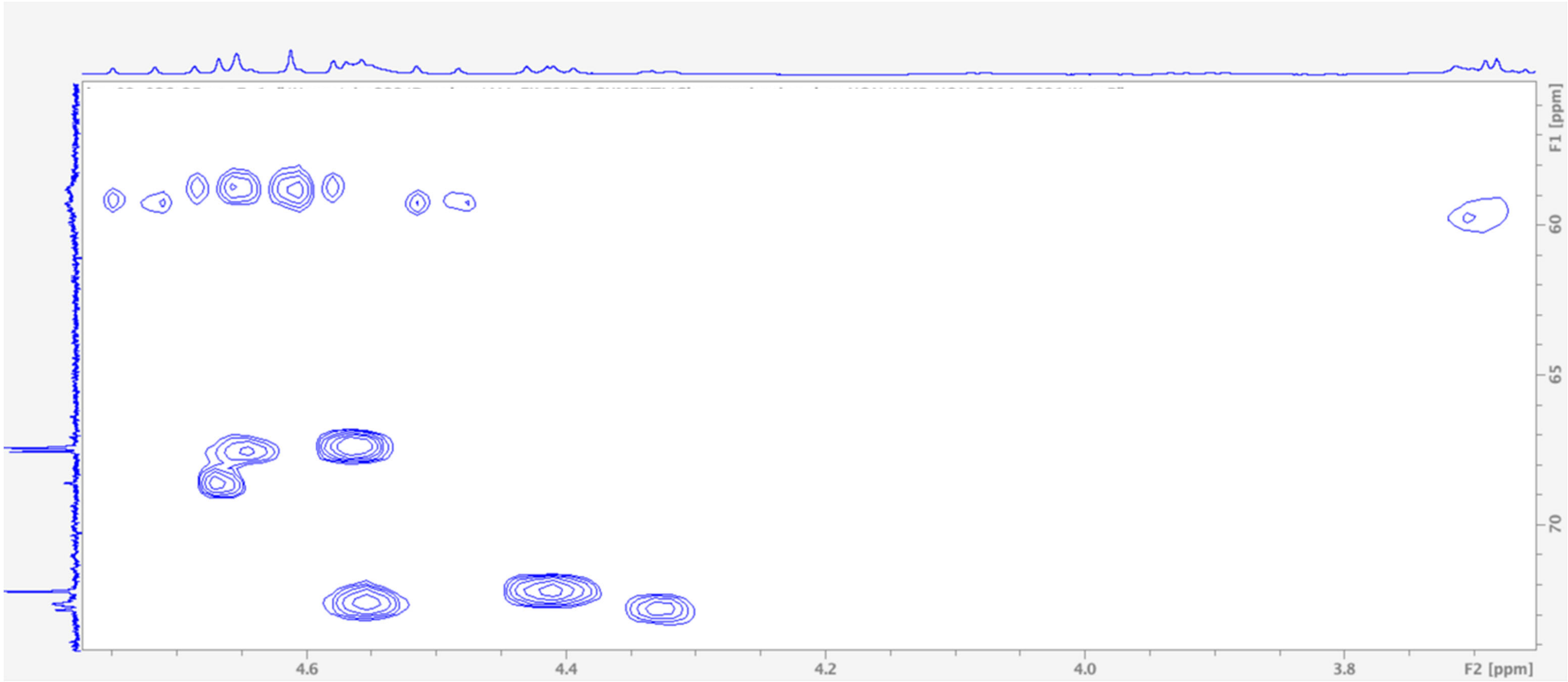
F1 [ppm]
60
65
70
4.6
4.4
4.2
4.0
3.8
F2 [ppm]

HMBC

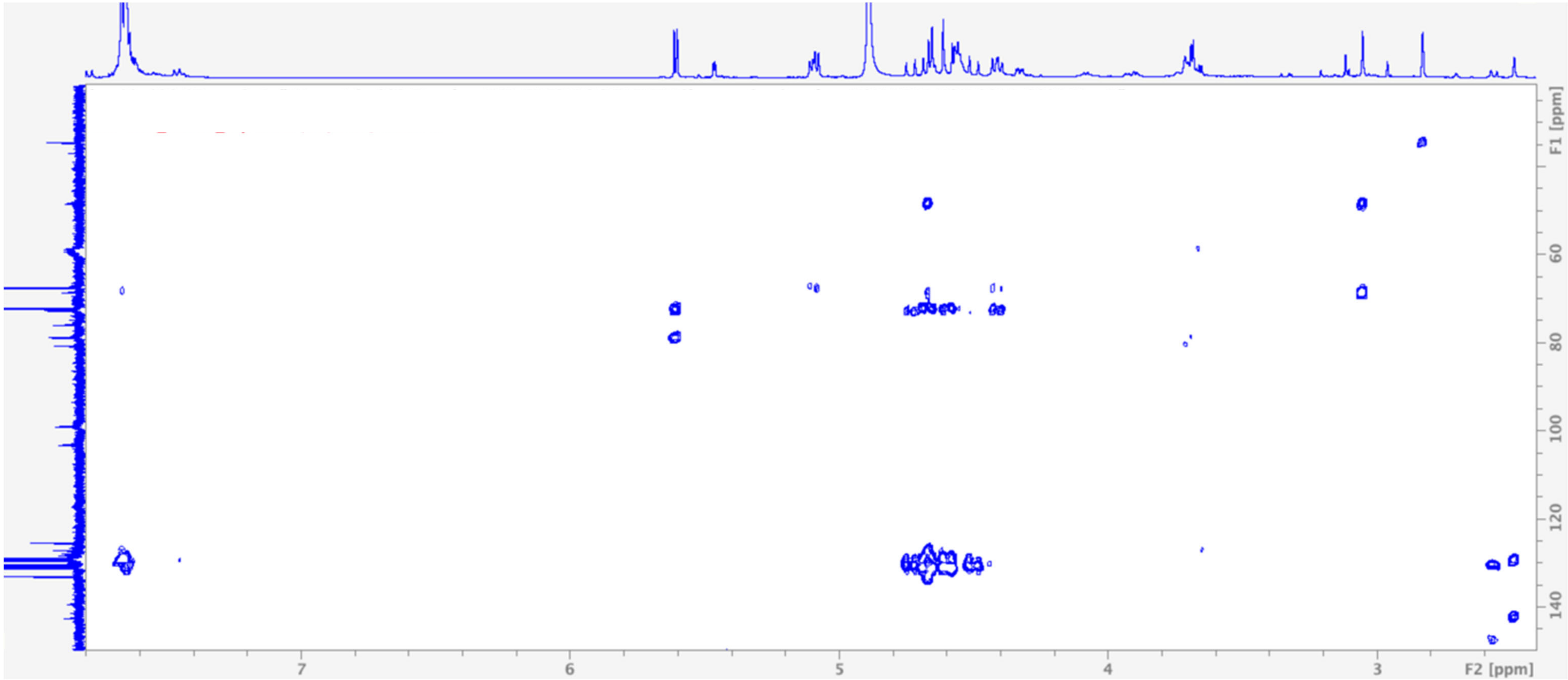

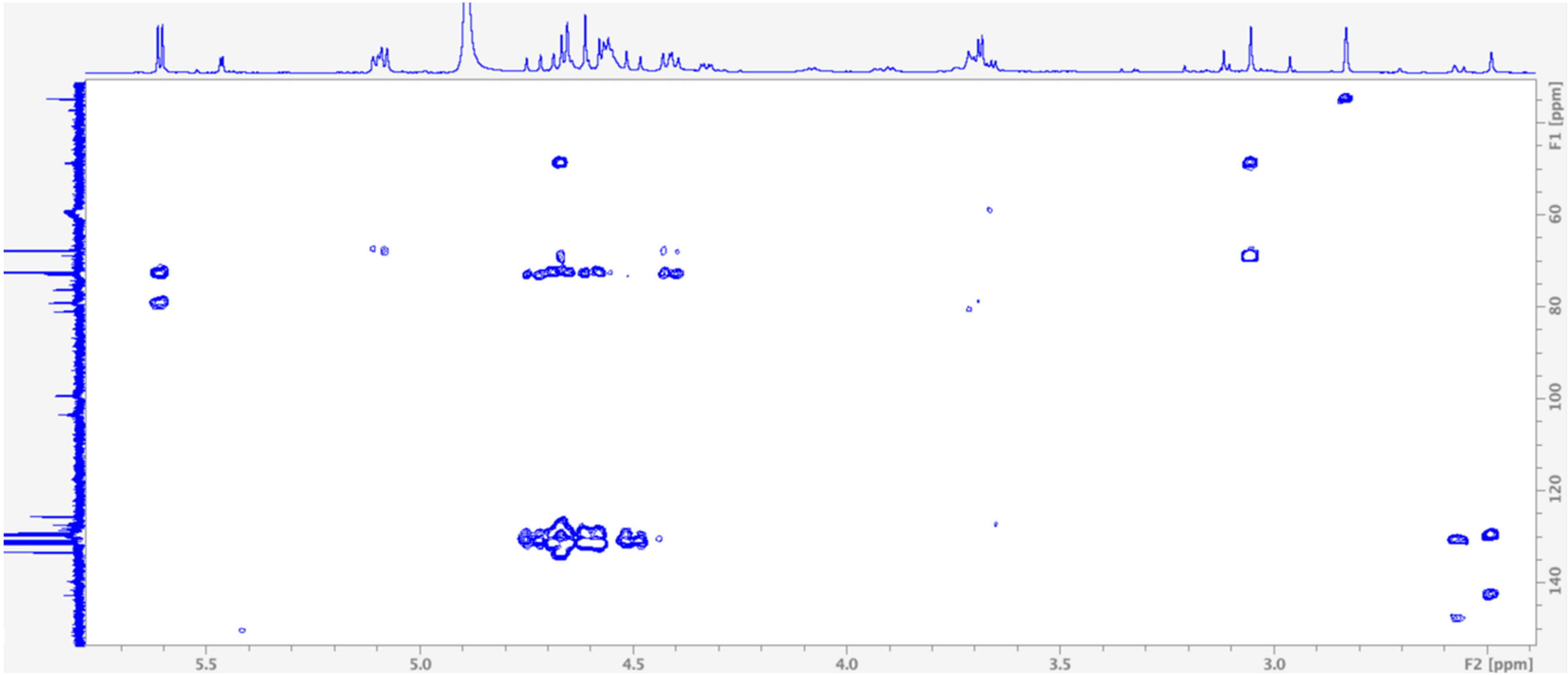
F1 [ppm]
60
80
100
120
140
5.5
5.0
4.5
4.0
3.5
3.0
F2 [ppm]

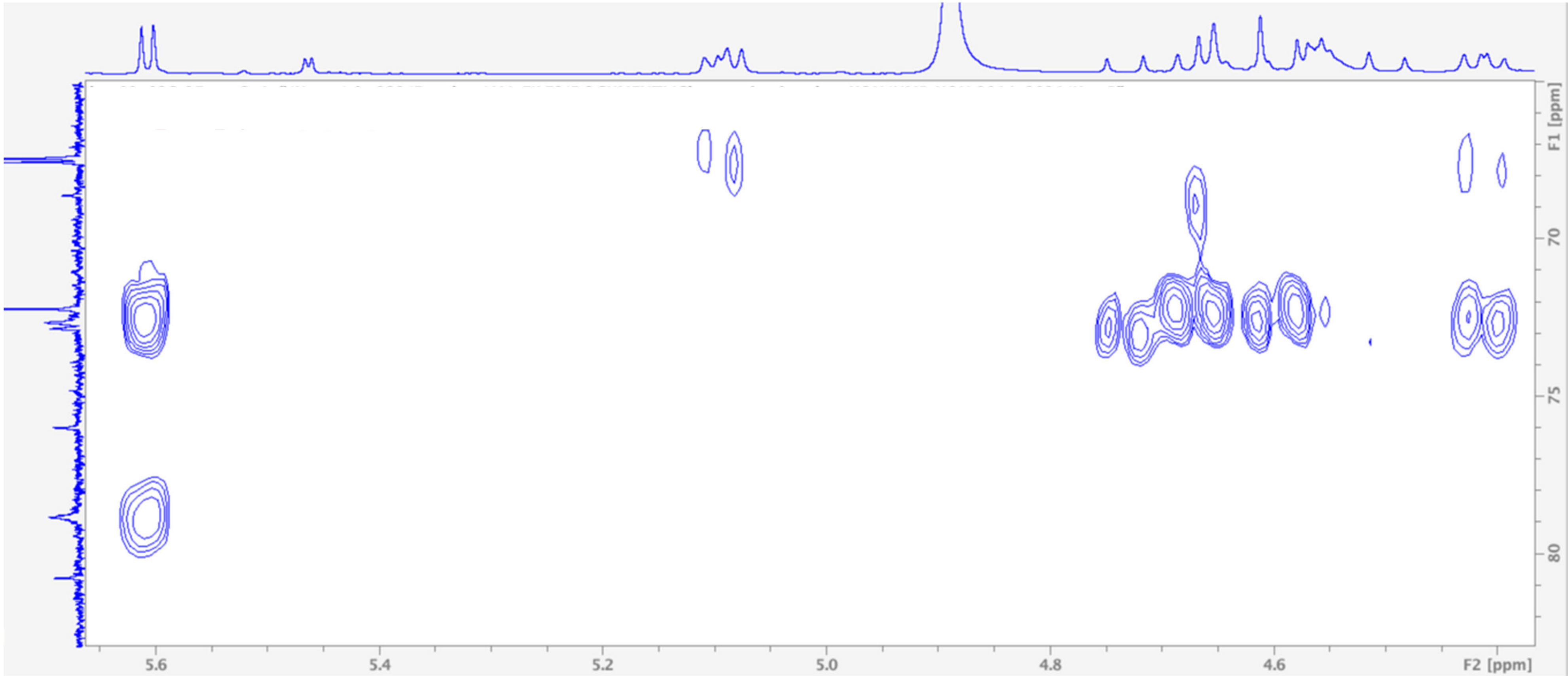

F1 [ppm]
70
75
80
5.6
5.4
5.2
5.0
4.8
4.6
F2 [ppm]

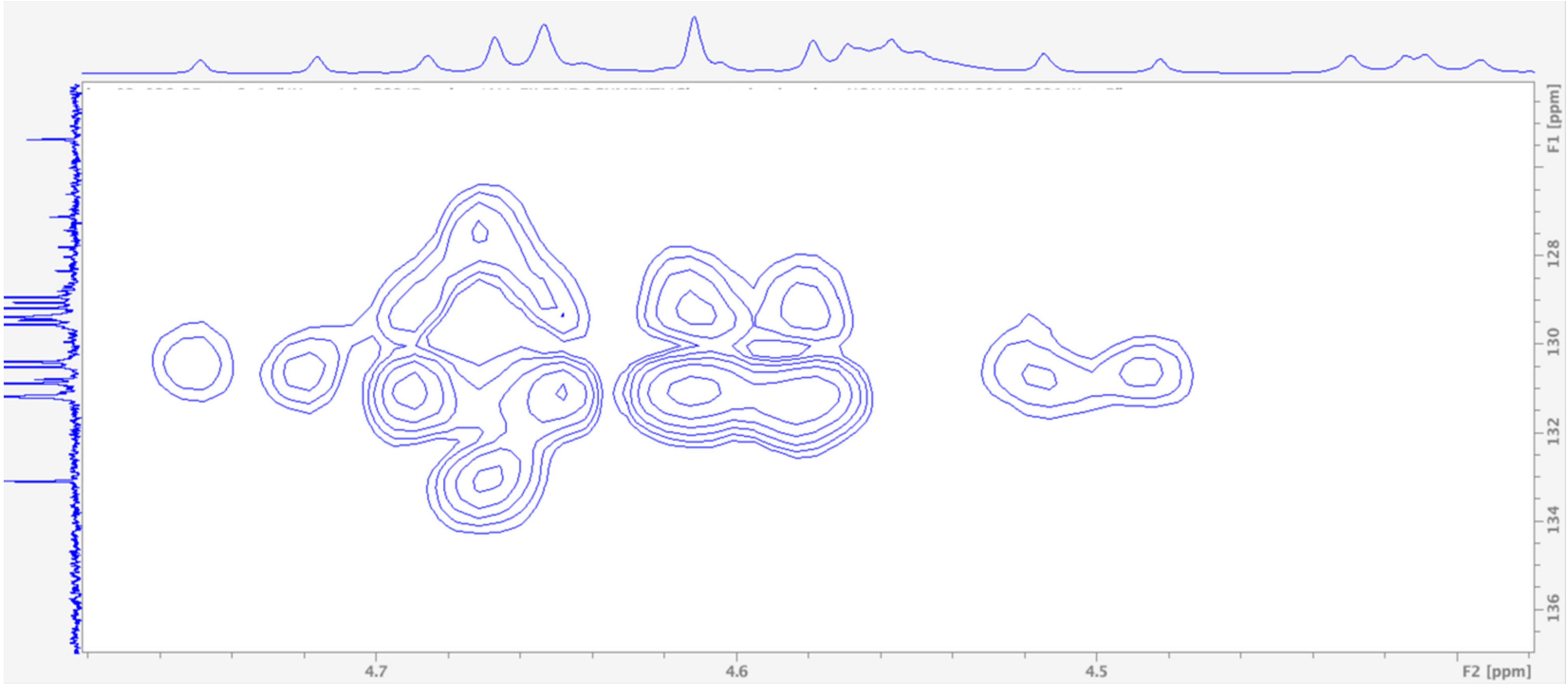
F1 [ppm]
128
130
132
134
136
4.7
4.6
4.5
F2 [ppm]

Figure S3. ^{1}H-NMR spectrum (400 MHz, D_2O) of *N*-benzyl-3,6-dideoxy-3,6-imino-D-gulofuranose **4** with colour-coded signals, highlighting the furanose anomeric forms they belong to, with interpretation of the isolated signals and tentative interpretation of the overlapping ones. Namely, the orange designates the *α-fur* form and indigo designates the *β-fur*. Section 8.10 ppm to 7.20 ppm is visible with highlighted the principal COSY correlations to hydrogen atoms within the same spin systems.

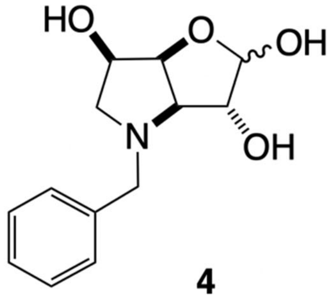

4

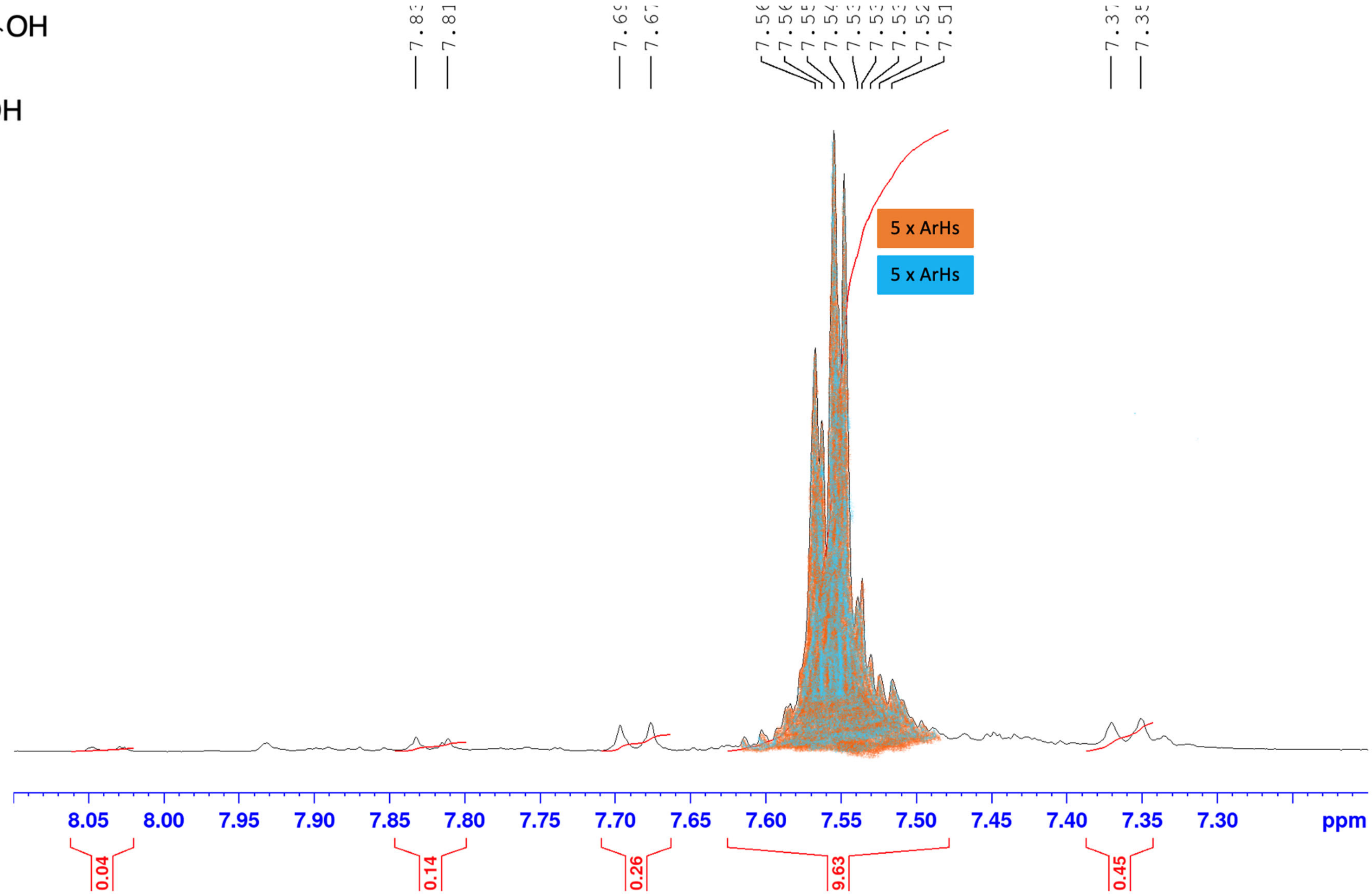

Figure S4. ^{1}H-NMR spectrum (400 MHz, D_2O) of *N*-benzyl-3,6-dideoxy-3,6-imino-D-gulofuranose **4** with colour-coded signals, highlighting the furanose anomeric forms they belong to, with interpretation of the isolated signals and tentative interpretation of the overlapping ones. Namely, the orange designates the *α-fur* form and indigo designates the *β-fur*. A) section 5.60 ppm to 4.90 ppm; B) section 4.70 ppm to 3.50 ppm. Highlighted are also the principal COSY correlations to hydrogen atoms within the same spin systems.

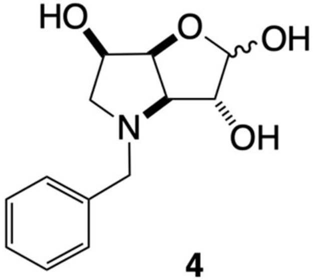

4

A

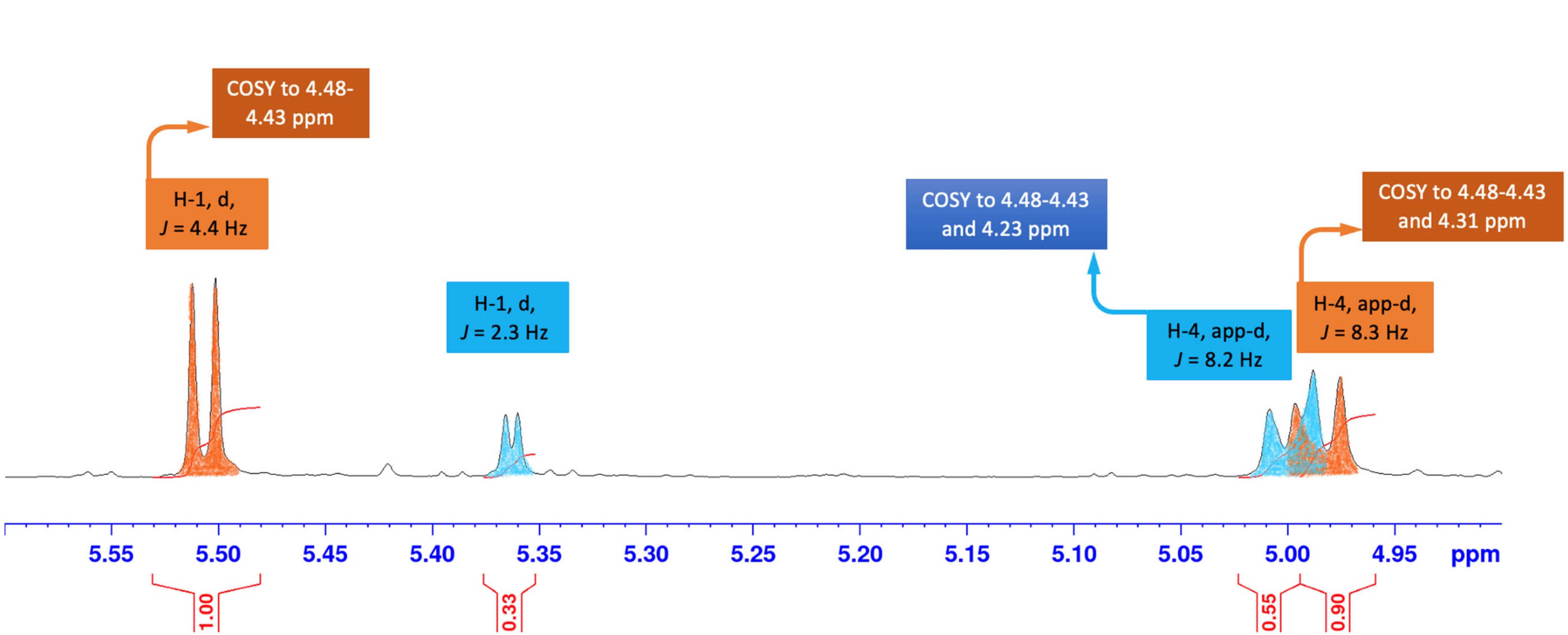

B

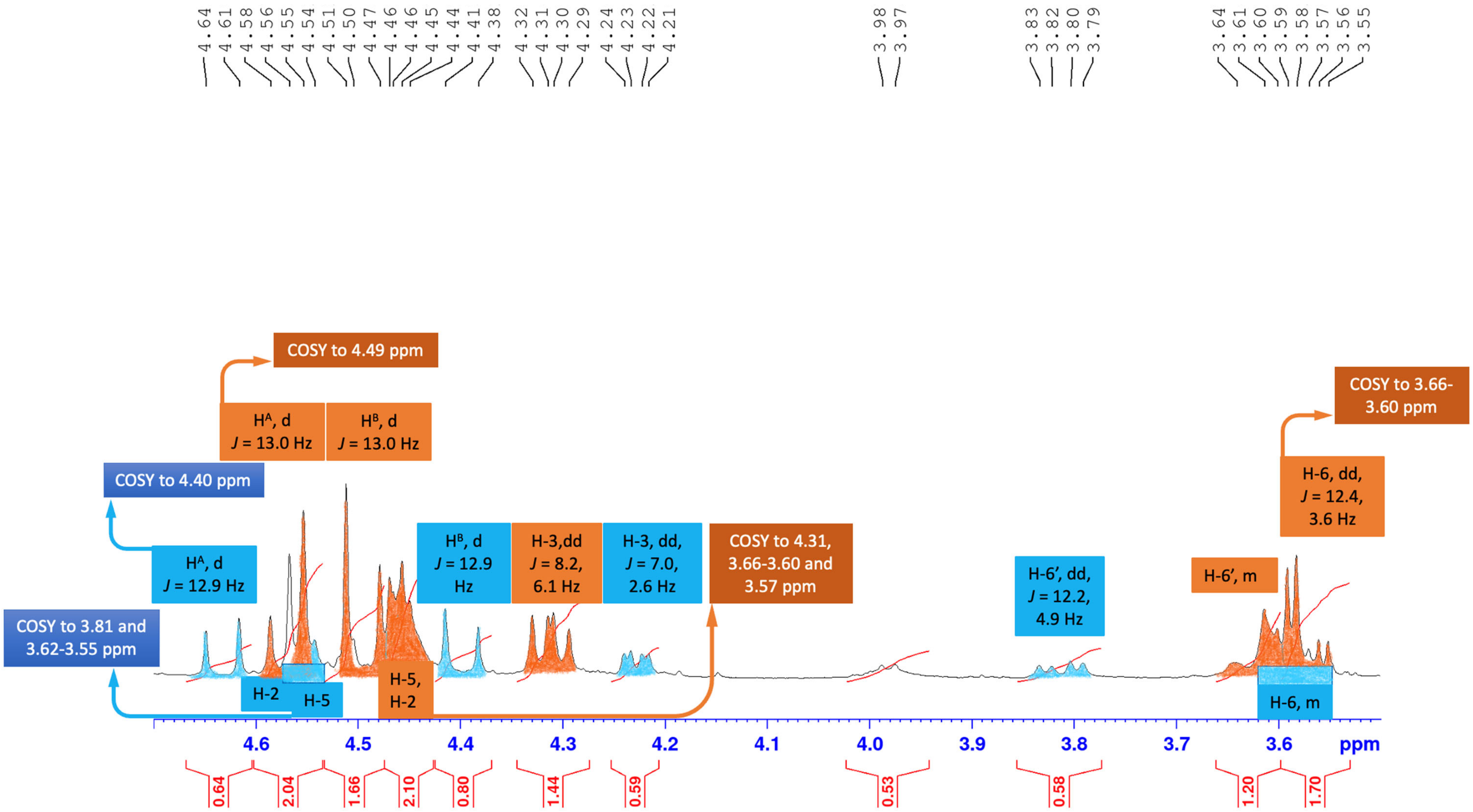
4.64 4.61 4.58 4.56 4.55 4.54 4.51 4.50 4.47 4.46 4.46 4.45 4.44 4.41 4.38 4.32 4.31 4.30 4.29 4.24 4.23 4.22 4.21
3.98 3.97
3.83 3.82 3.80 3.79
3.64 3.61 3.60 3.59 3.58 3.57 3.56 3.55
COSY to 4.49 ppm
H^A, d J = 13.0 Hz
H^B, d J = 13.0 Hz
COSY to 4.40 ppm
COSY to 3.66-3.60 ppm
H-6, dd, J = 12.4, 3.6 Hz
H^A, d J = 12.9 Hz
H^B, d J = 12.9 Hz
H-3,dd J = 8.2, 6.1 Hz
H-3, dd, J = 7.0, 2.6 Hz
COSY to 4.31, 3.66-3.60 and 3.57 ppm
H-6′, dd, J = 12.2, 4.9 Hz
H-6′, m
COSY to 3.81 and 3.62-3.55 ppm
H-2
H-5
H-5, H-2
H-6, m
4.6 4.5 4.4 4.3 4.2 4.1 4.0 3.9 3.8 3.7 3.6 ppm
0.64 2.04 1.66 2.10 0.80 1.44 0.59 0.53 0.58 1.20 1.70

Figure S5. ^{13}C-NMR spectrum (100 MHz, D_2O) sections of *N*-benzyl-3,6-dideoxy-3,6-imino-D-gulofuranose **4** with colour-coded signals, highlighting the furanose anomeric forms they belong to, with interpretation of the isolated signals and tentative interpretation of the overlapping ones. Namely, the orange designates the *α-fur* form and indigo designates the *β-fur*. A) section 143.0 ppm to 127.5 ppm; B) section 105.0 ppm to 76.0 ppm; C) section 75.0 ppm to 48.5 ppm. Highlighted are also the principal HSQC correlations to hydrogen atoms within the same spin systems.

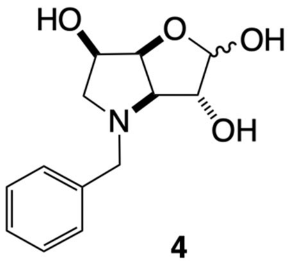

4

A

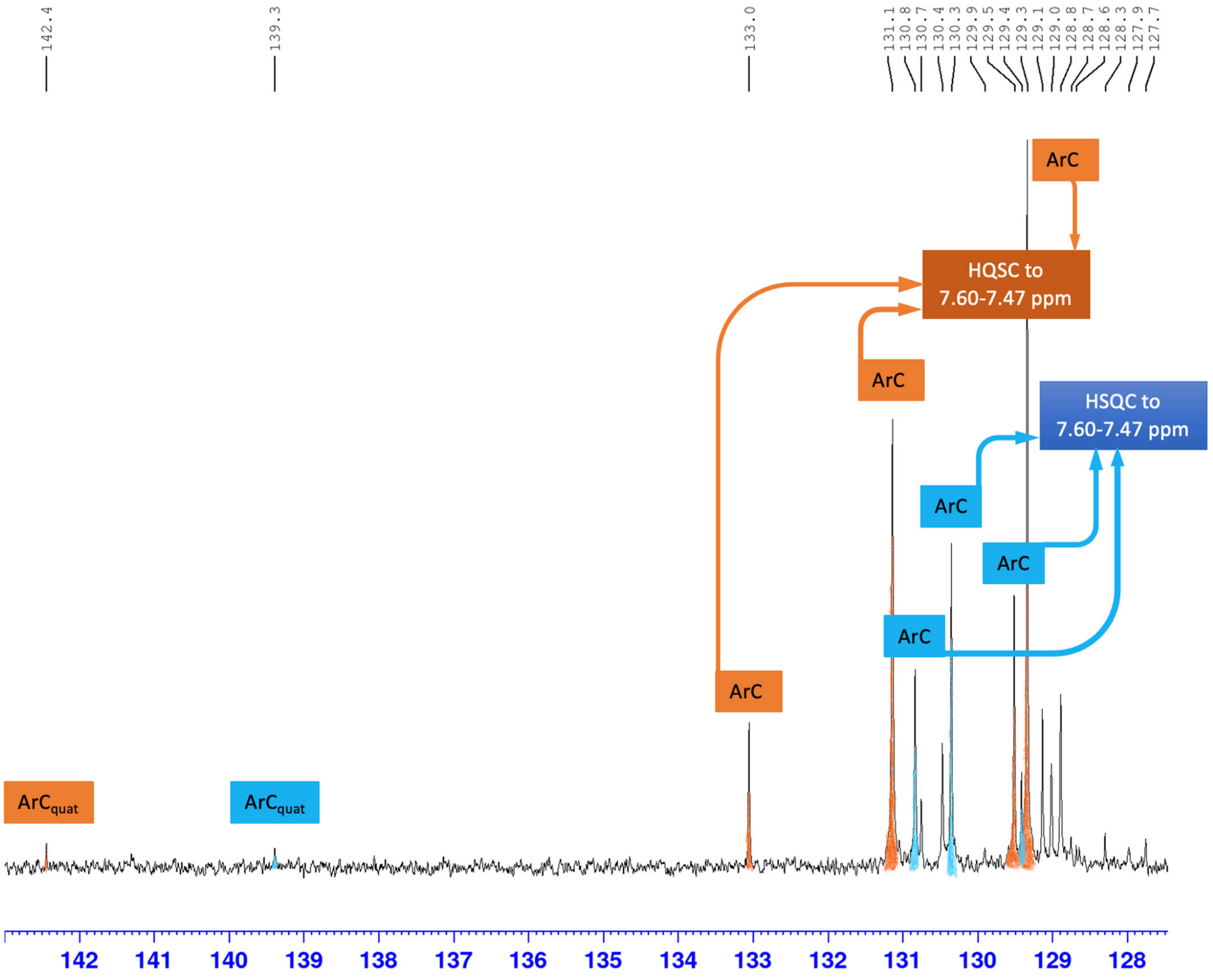

B

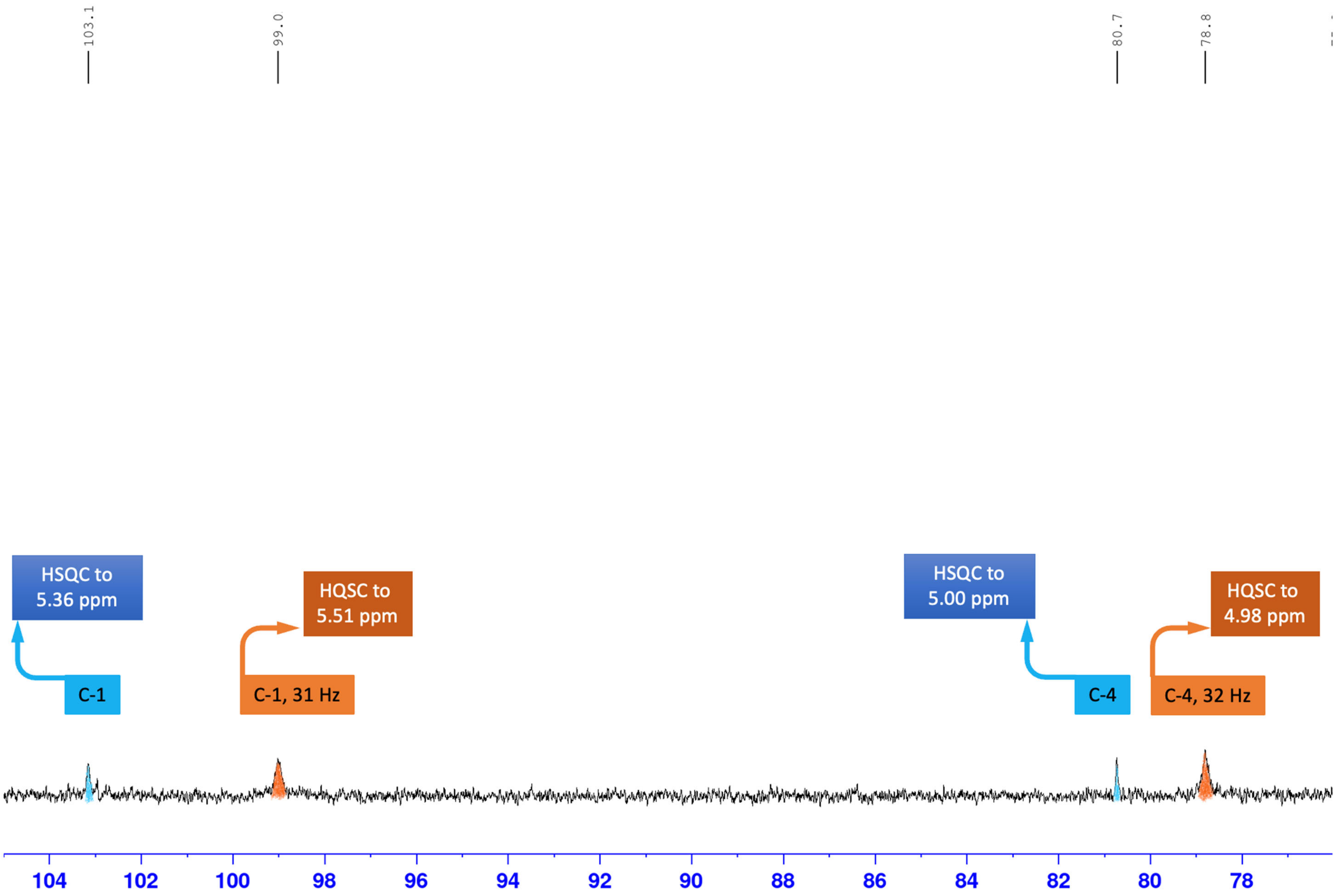

103.1
99.0
80.7
78.8
HSQC to 5.36 ppm
HQSC to 5.51 ppm
HSQC to 5.00 ppm
HQSC to 4.98 ppm
C-1
C-1, 31 Hz
C-4
C-4, 32 Hz
104
102
100
98
96
94
92
90
88
86
84
82
80
78

C

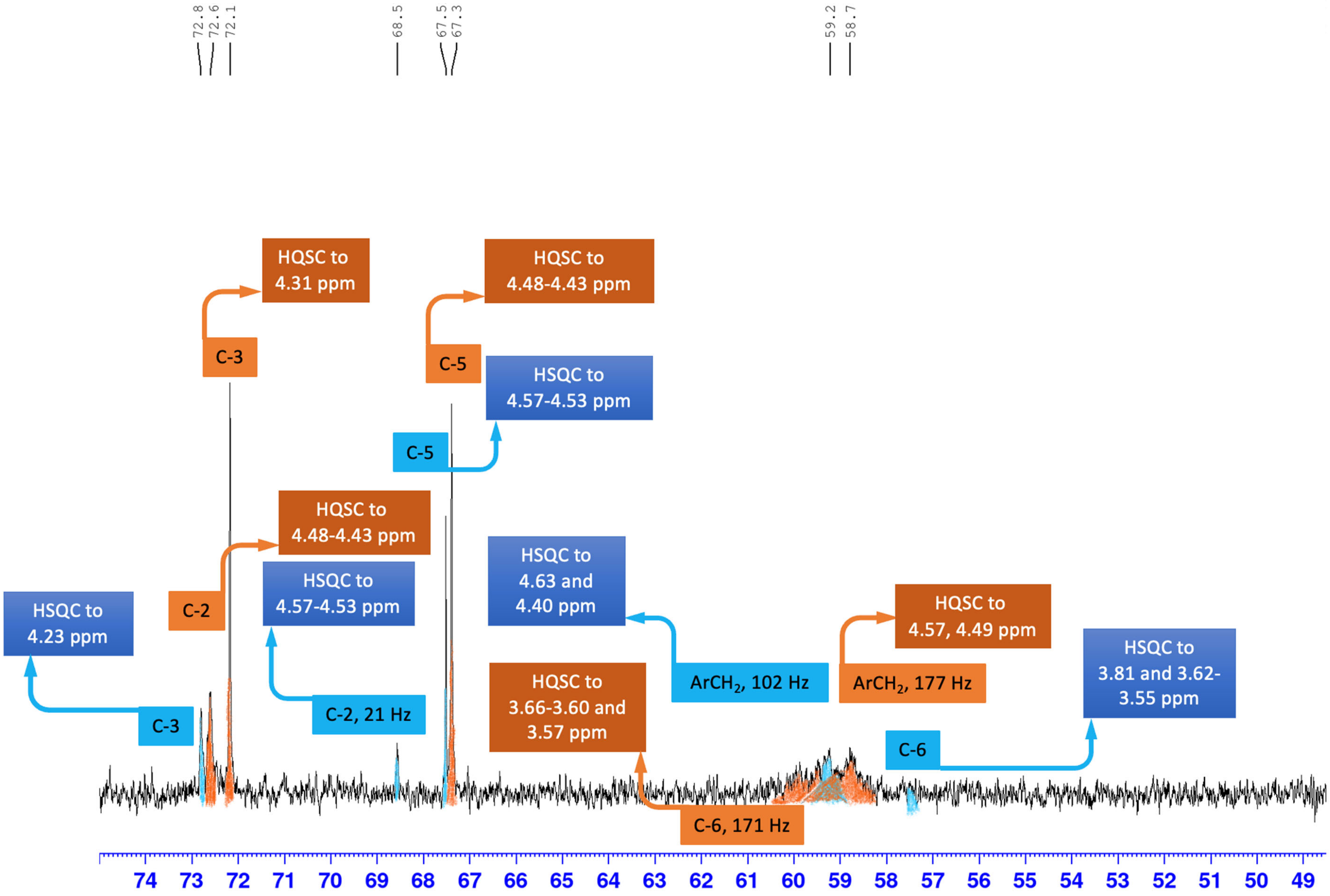
72.8
72.6
72.1
68.5
67.5
67.3
59.2
58.7
HQSC to
4.31 ppm
C-3
HQSC to
4.48-4.43 ppm
C-5
HSQC to
4.57-4.53 ppm
C-5
HQSC to
4.48-4.43 ppm
C-2
HSQC to
4.57-4.53 ppm
HSQC to
4.23 ppm
C-3
C-2, 21 Hz
HSQC to
4.63 and
4.40 ppm
HQSC to
3.66-3.60 and
3.57 ppm
ArCH2, 102 Hz
HQSC to
4.57, 4.49 ppm
ArCH2, 177 Hz
HSQC to
3.81 and 3.62-
3.55 ppm
C-6
C-6, 171 Hz
74 73 72 71 70 69 68 67 66 65 64 63 62 61 60 59 58 57 56 55 54 53 52 51 50 49

Figure S6. ^{1}H- (400 MHz), ^{13}C-NMR (100 MHz) and COSY spectra of *N*-benzyl-1,4-dideoxy-1,4-imino-L-gulitol **5** in D_2O.

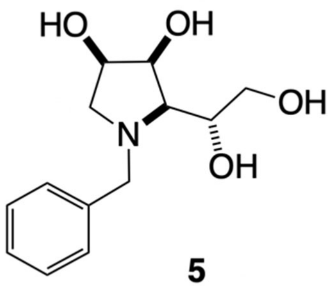

5

^{1}H-NMR

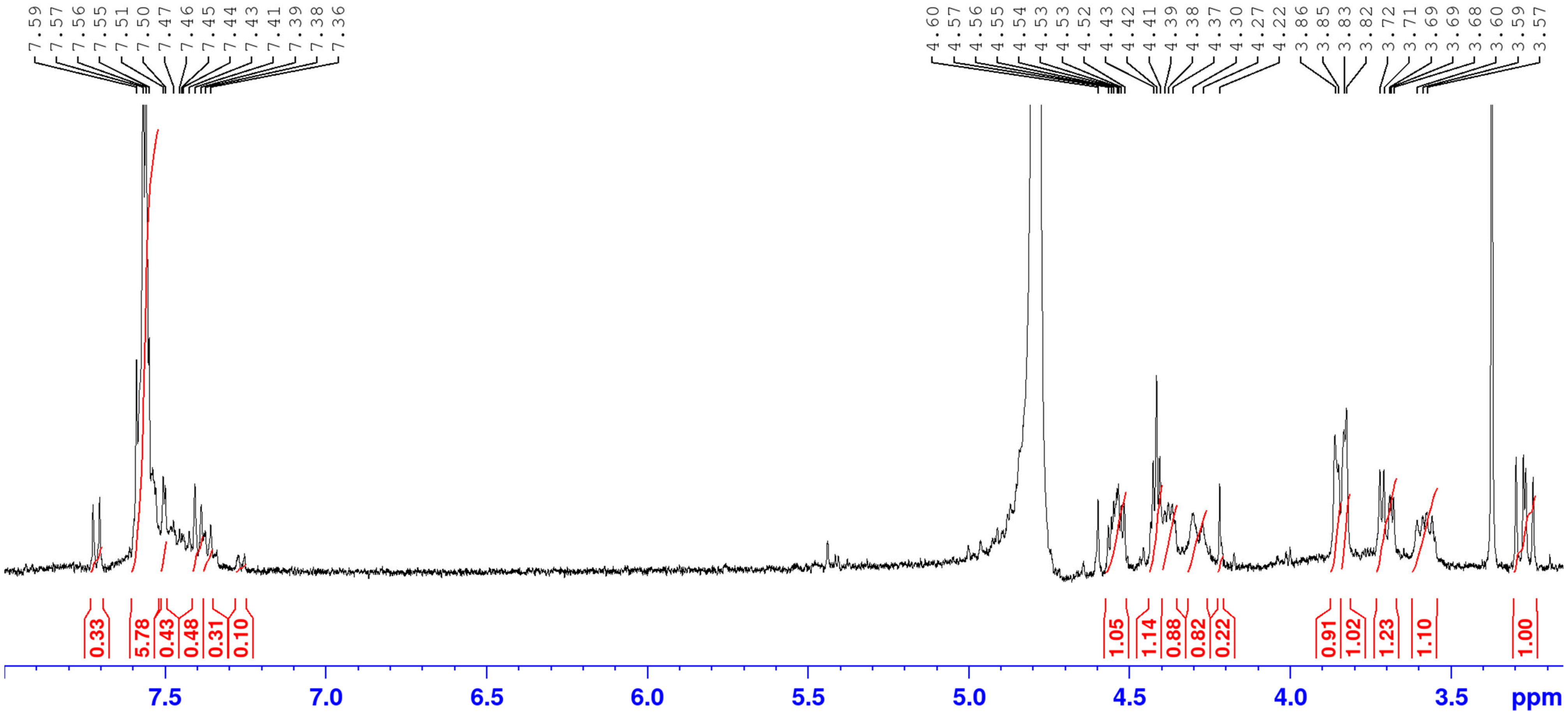

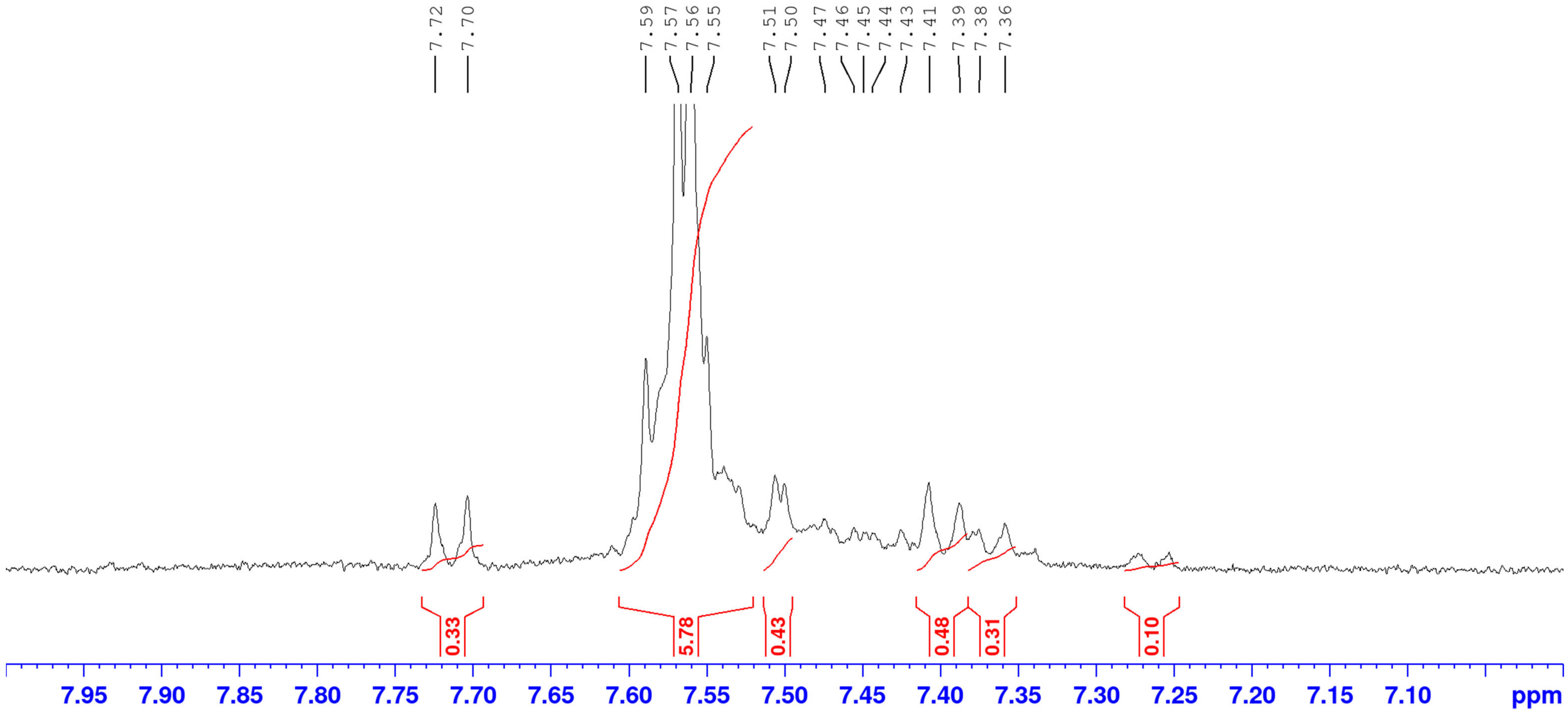
7.72
7.70
7.59
7.57
7.56
7.55
7.51
7.50
7.47
7.46
7.45
7.44
7.43
7.41
7.39
7.38
7.36
0.33
5.78
0.43
0.48
0.31
0.10
7.95 7.90 7.85 7.80 7.75 7.70 7.65 7.60 7.55 7.50 7.45 7.40 7.35 7.30 7.25 7.20 7.15 7.10 ppm

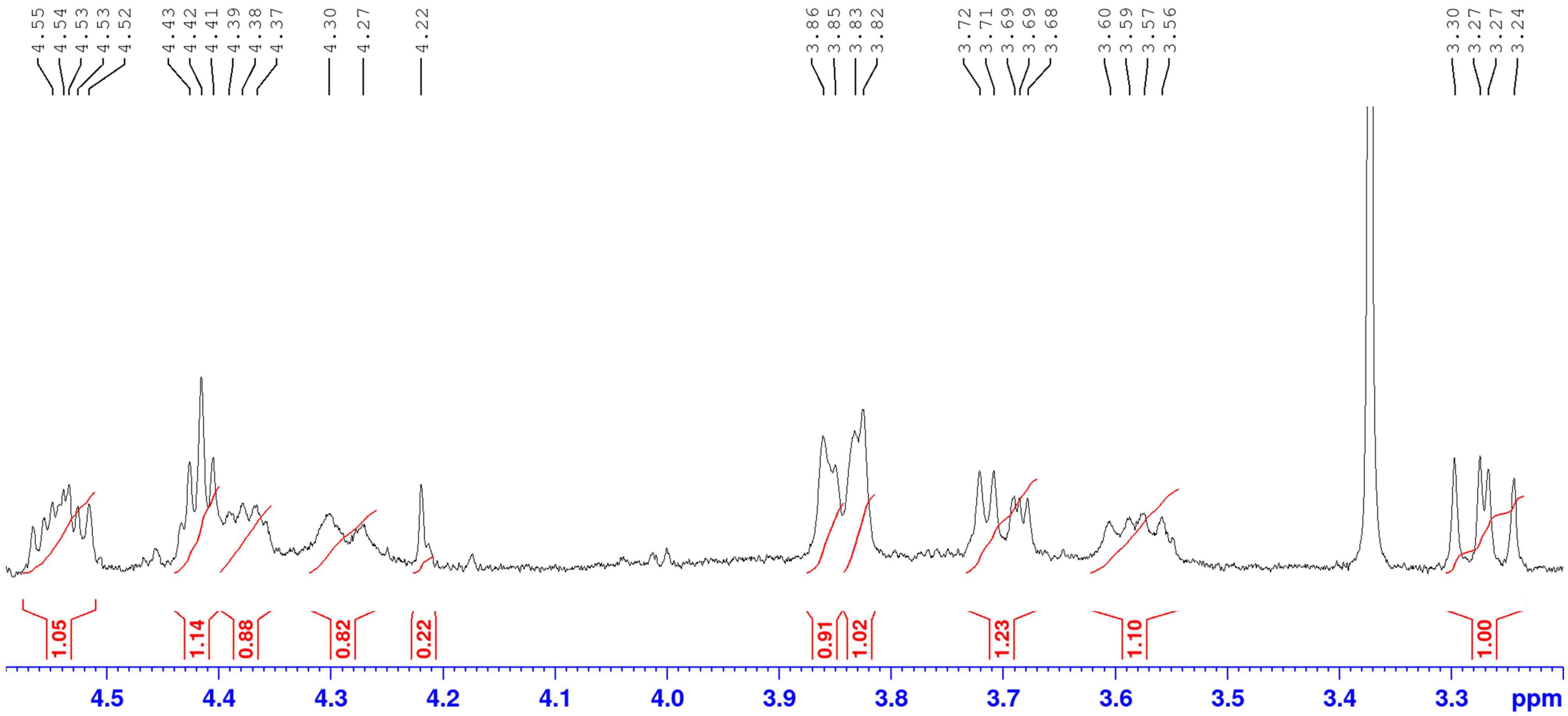
4.55
4.54
4.53
4.53
4.52
4.43
4.42
4.41
4.39
4.38
4.37
4.30
4.27
4.22
3.86
3.85
3.83
3.82
3.72
3.71
3.69
3.69
3.68
3.60
3.59
3.57
3.56
3.30
3.27
3.27
3.24
1.05
1.14
0.88
0.82
0.22
0.91
1.02
1.23
1.10
1.00
4.5
4.4
4.3
4.2
4.1
4.0
3.9
3.8
3.7
3.6
3.5
3.4
3.3
ppm

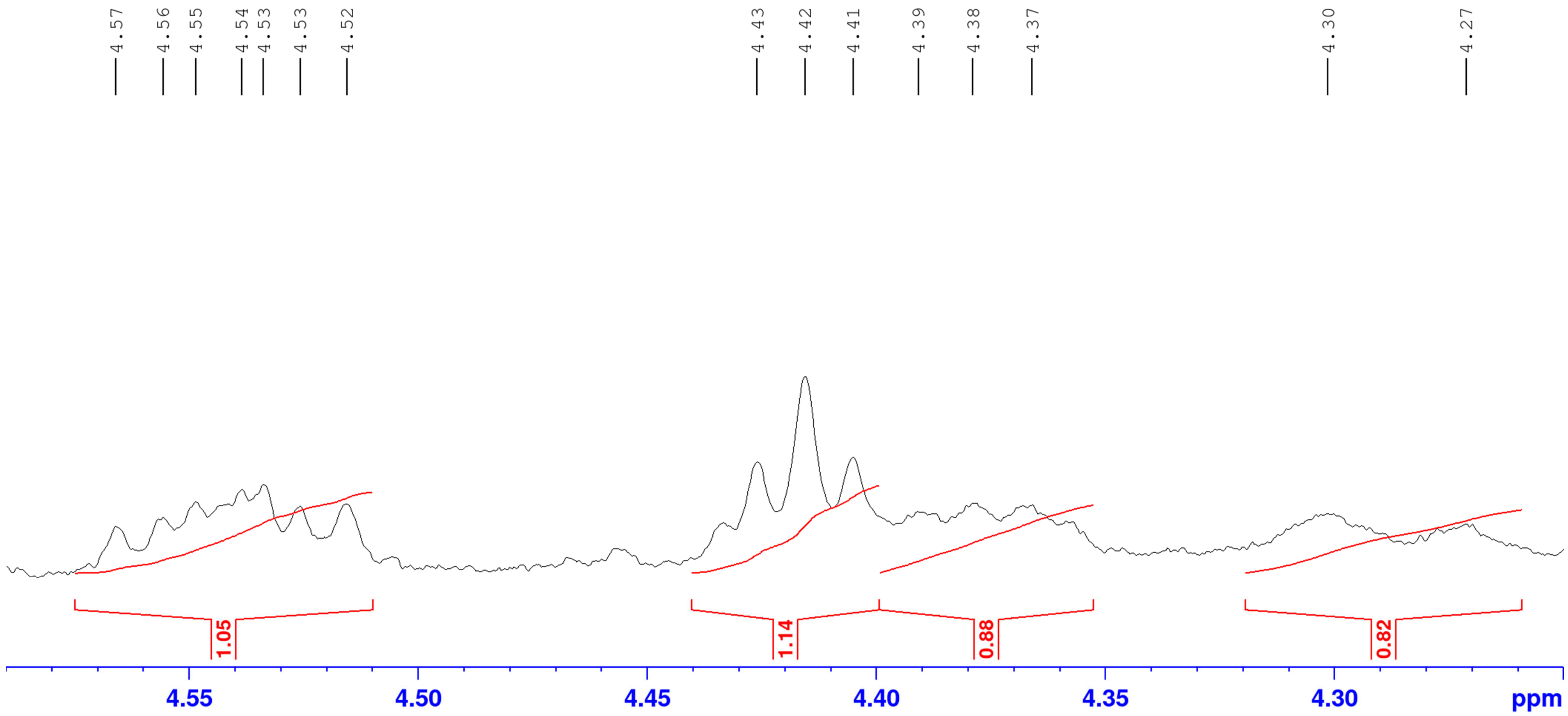
4.57
4.56
4.55
4.54
4.53
4.53
4.52
4.43
4.42
4.41
4.39
4.38
4.37
4.30
4.27
1.05
1.14
0.88
0.82
4.55
4.50
4.45
4.40
4.35
4.30
ppm

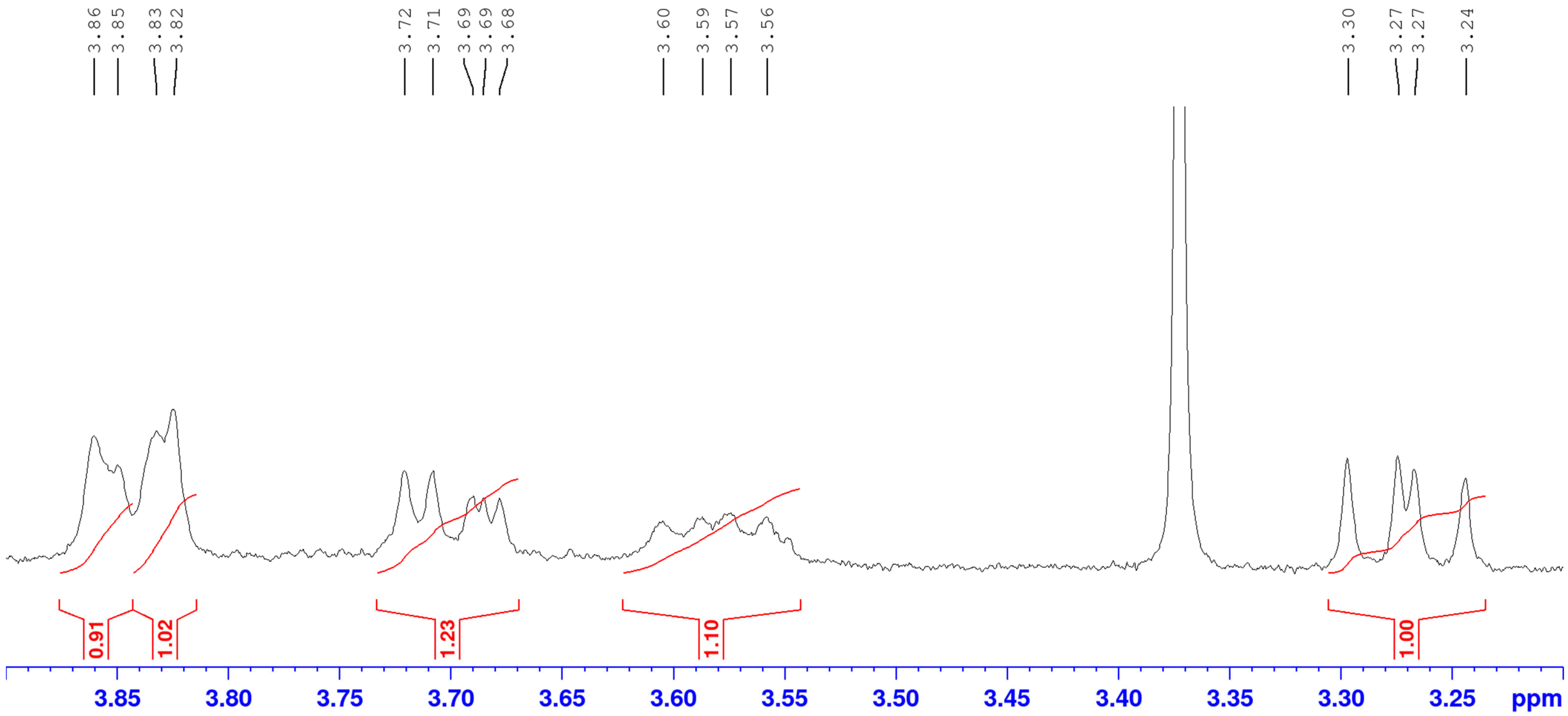
3.86
3.85
3.83
3.82
3.72
3.71
3.69
3.69
3.68
3.60
3.59
3.57
3.56
3.30
3.27
3.27
3.24
0.91
1.02
1.23
1.10
1.00
3.85
3.80
3.75
3.70
3.65
3.60
3.55
3.50
3.45
3.40
3.35
3.30
3.25
ppm

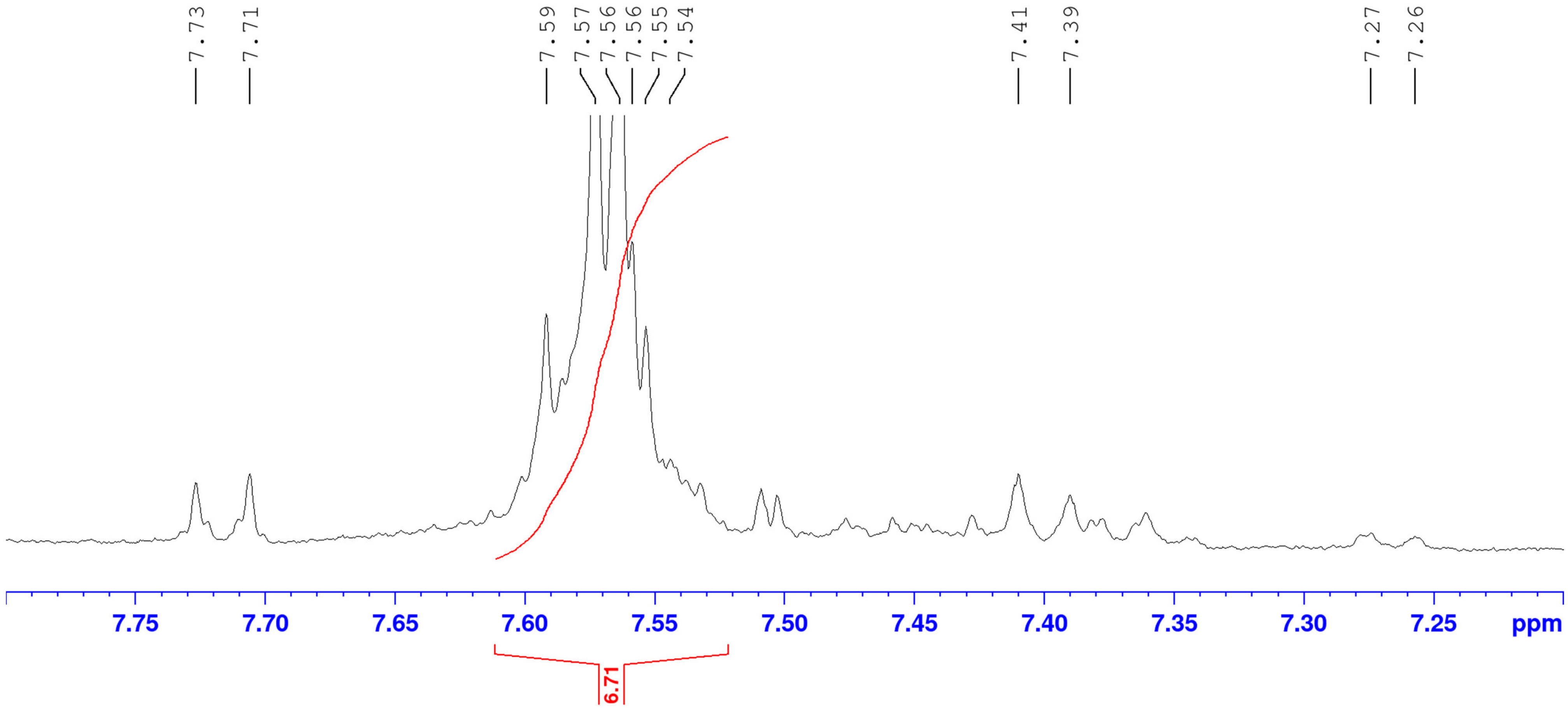
7.73
7.71
7.59
7.57
7.56
7.56
7.55
7.54
7.41
7.39
7.27
7.26
7.75
7.70
7.65
7.60
7.55
7.50
7.45
7.40
7.35
7.30
7.25
ppm
6.71

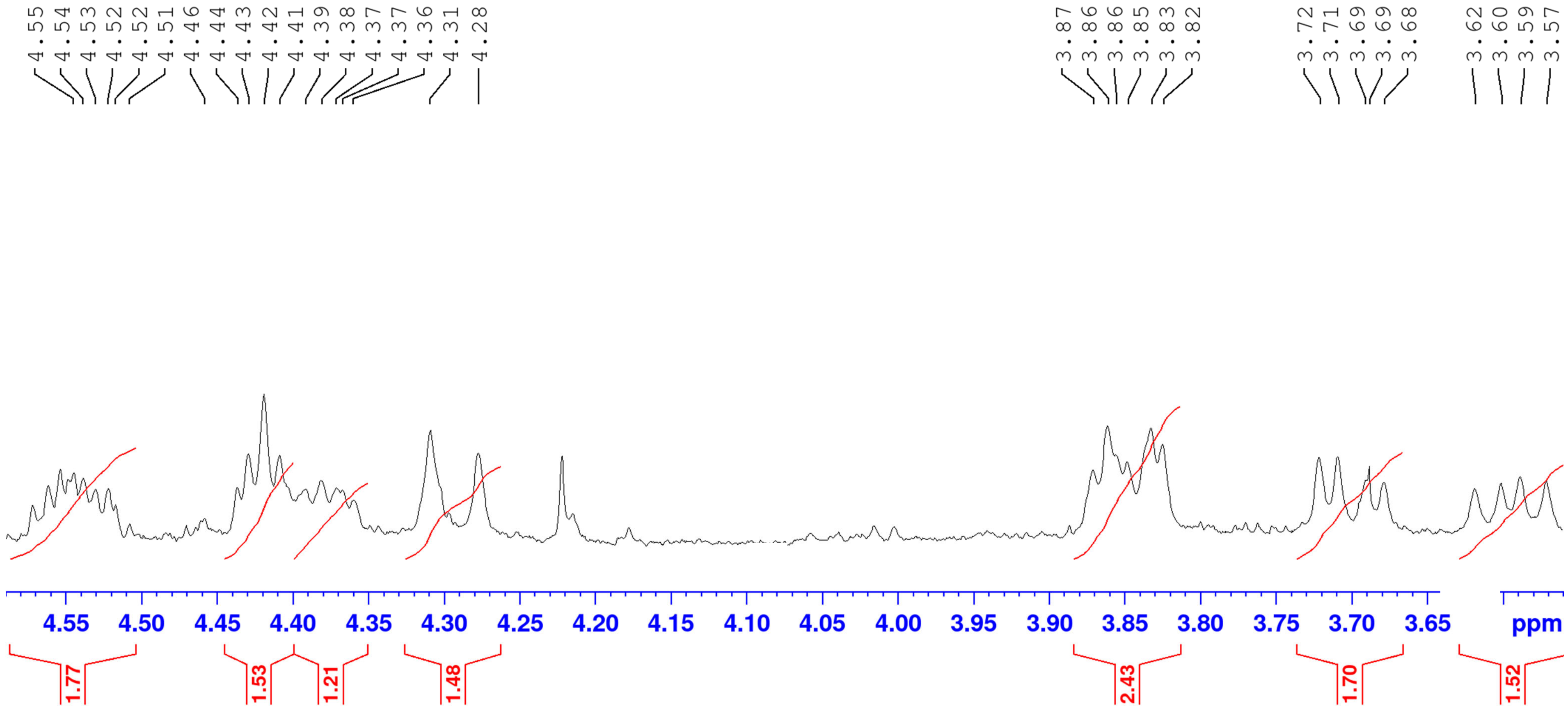
4.55
4.54
4.53
4.52
4.52
4.51
4.46
4.44
4.43
4.42
4.41
4.39
4.38
4.37
4.37
4.36
4.31
4.28
3.87
3.86
3.86
3.85
3.83
3.82
3.72
3.71
3.69
3.69
3.68
3.62
3.60
3.59
3.57
4.55 4.50 4.45 4.40 4.35 4.30 4.25 4.20 4.15 4.10 4.05 4.00 3.95 3.90 3.85 3.80 3.75 3.70 3.65 ppm
1.77
1.53
1.21
1.48
2.43
1.70
1.52

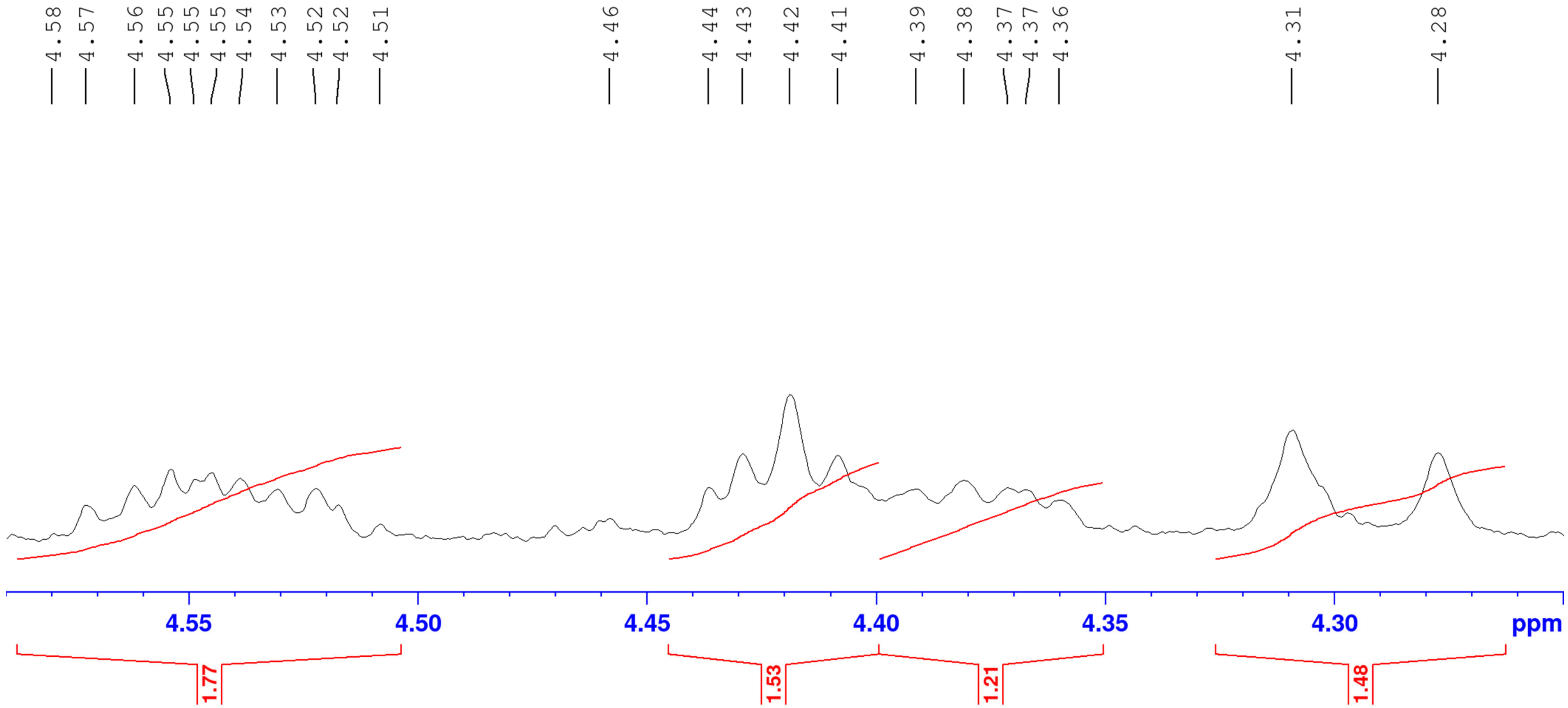
4.58
4.57
4.56
4.55
4.55
4.55
4.54
4.53
4.52
4.52
4.51
4.46
4.44
4.43
4.42
4.41
4.39
4.38
4.37
4.37
4.36
4.31
4.28
4.55
4.50
4.45
4.40
4.35
4.30
ppm
1.77
1.53
1.21
1.48

^{13}C-NMR

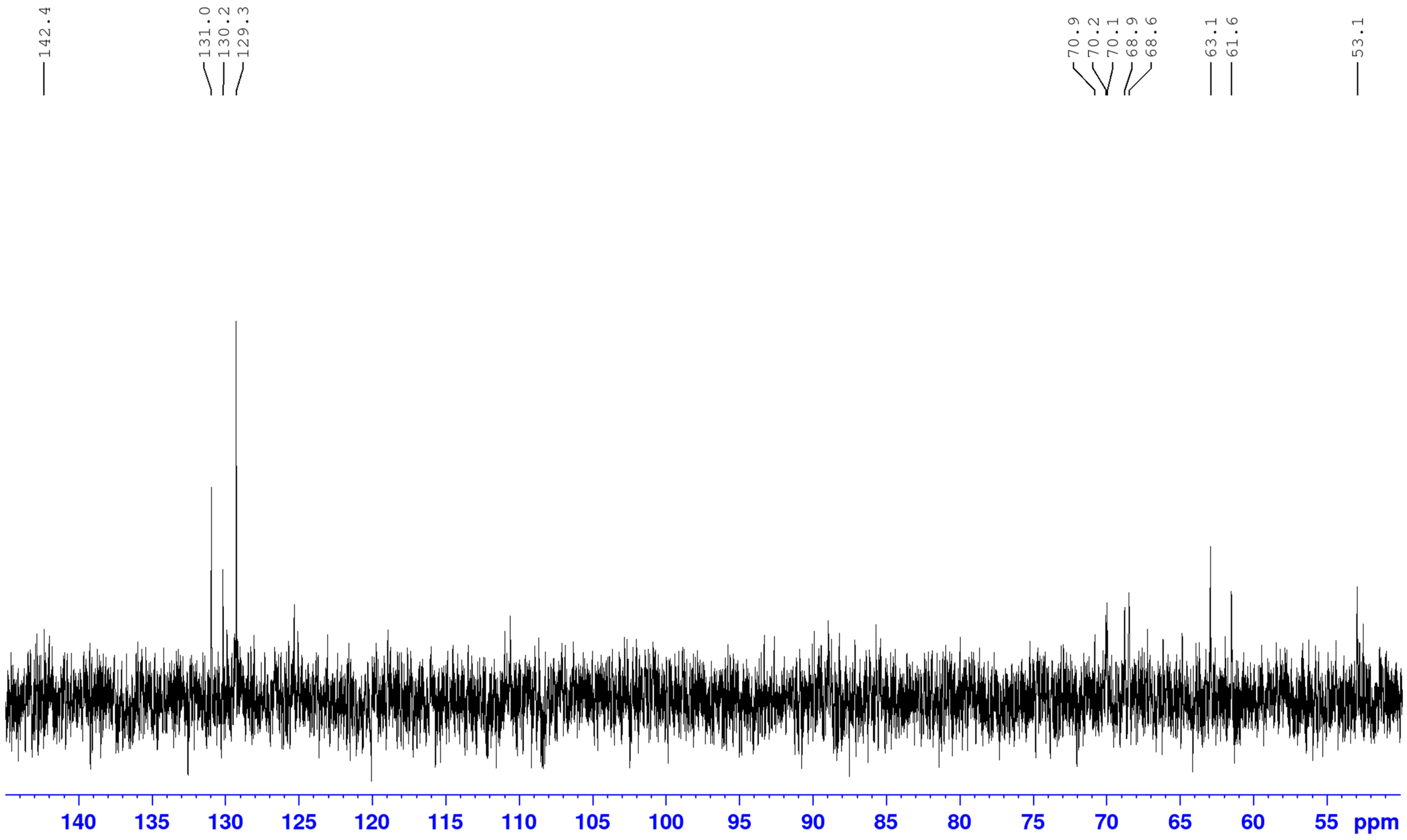

142.4
131.0
130.2
129.3
70.9
70.2
70.1
68.9
68.6
63.1
61.6
53.1
140 135 130 125 120 115 110 105 100 95 90 85 80 75 70 65 60 55 ppm

COSY

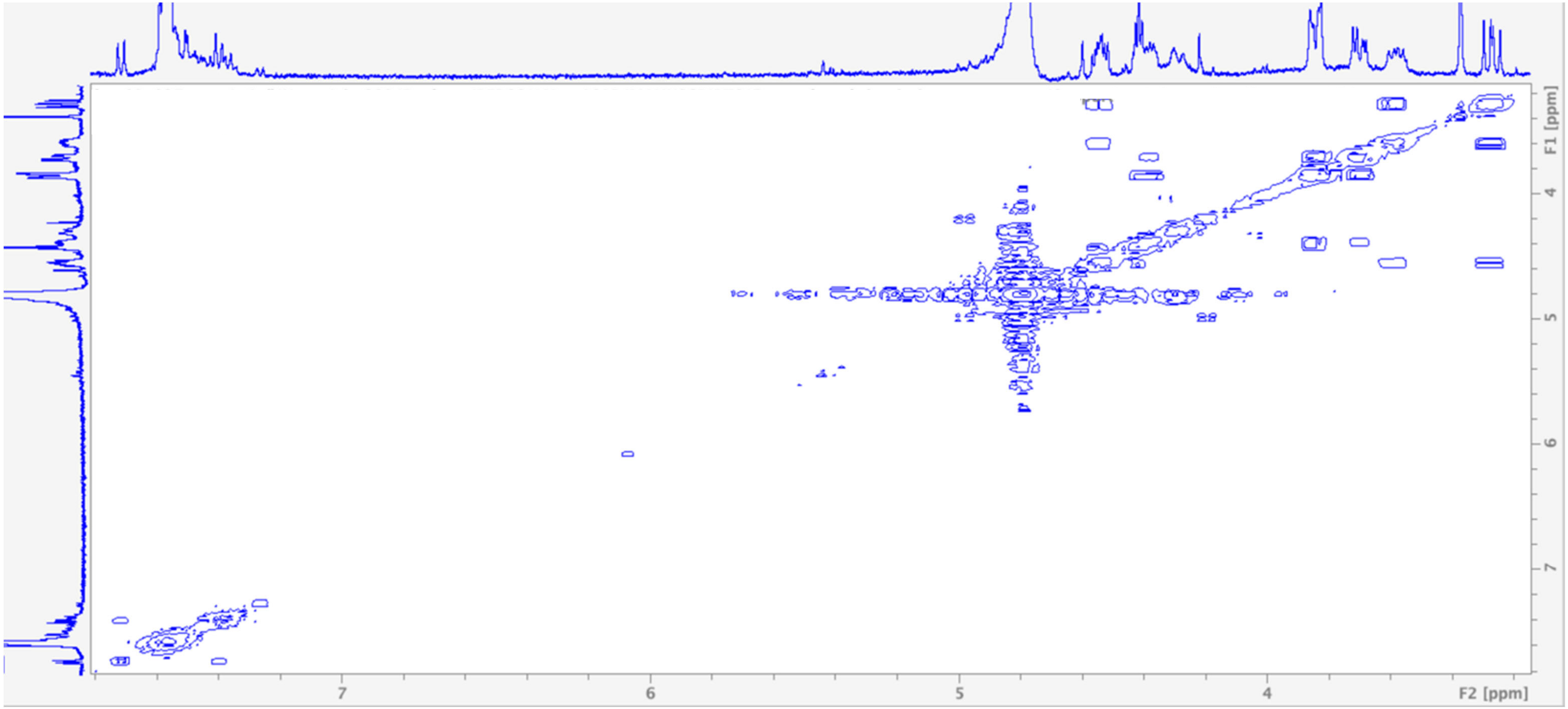

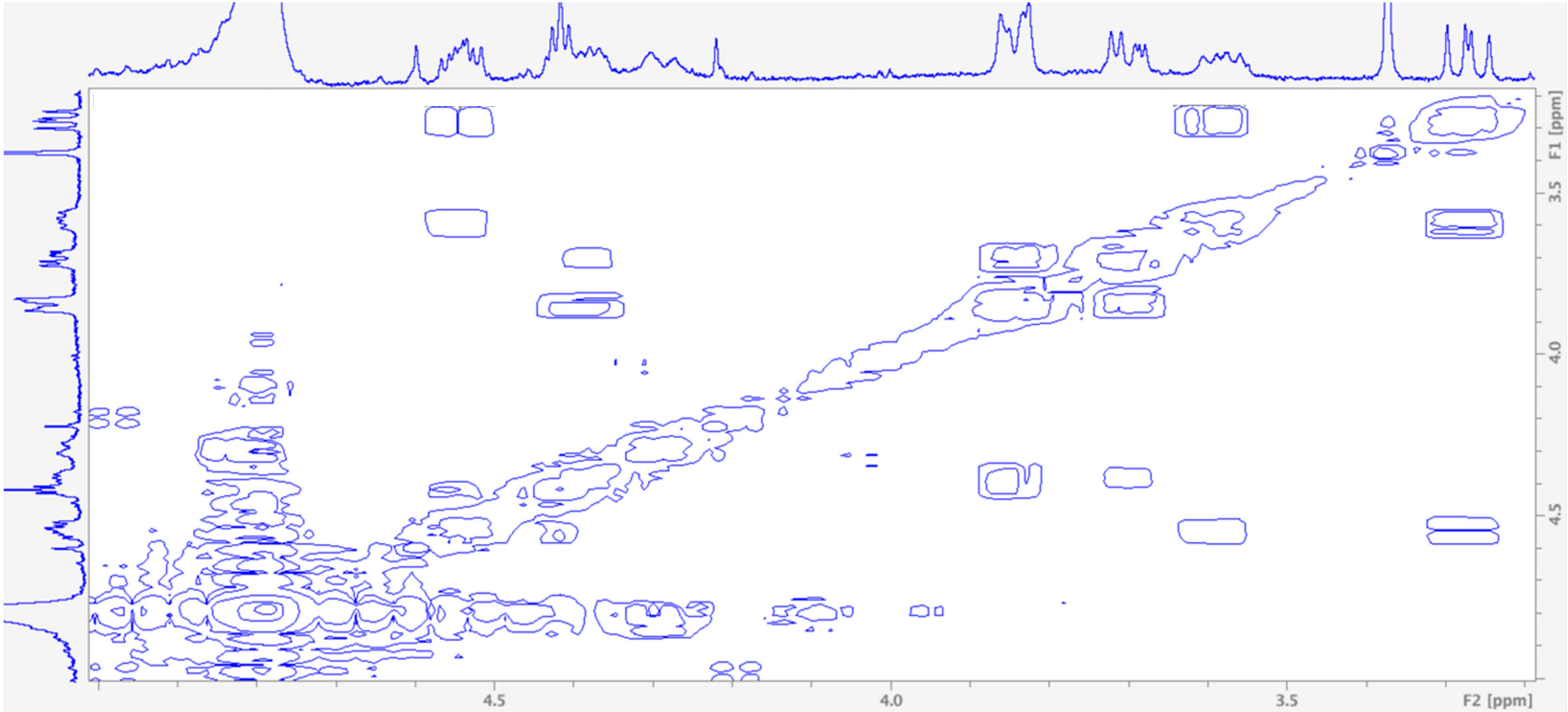

4.5
4.0
3.5
F2 [ppm]
F1 [ppm]

Figure S7. Sections of the ^{1}H-NMR spectrum (400 MHz, D_2O) of *N*-benzyl-1,4-dideoxy-1,4-imino-L-gulitol **5** with selected signals colour-coded. Namely, the orange and green colour-codes are used for overlapping signals to show the splitting pattern. The green and orange vertical lines designate the locations of the peaks for complex splitting patterns. A) section 4.59 ppm to 4.25 ppm; B) section 3.92 ppm to 3.20 ppm. Highlighted are also the principal COSY correlations between hydrogen atoms.

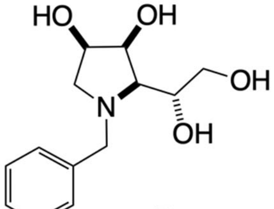

5

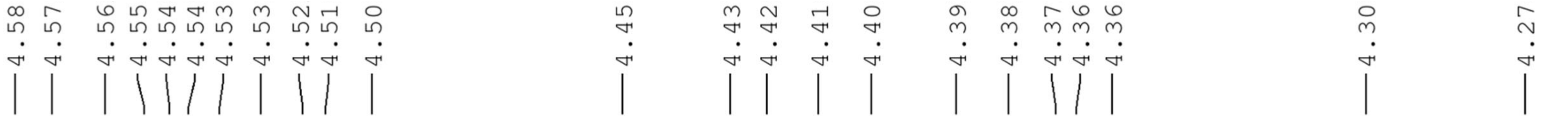

A

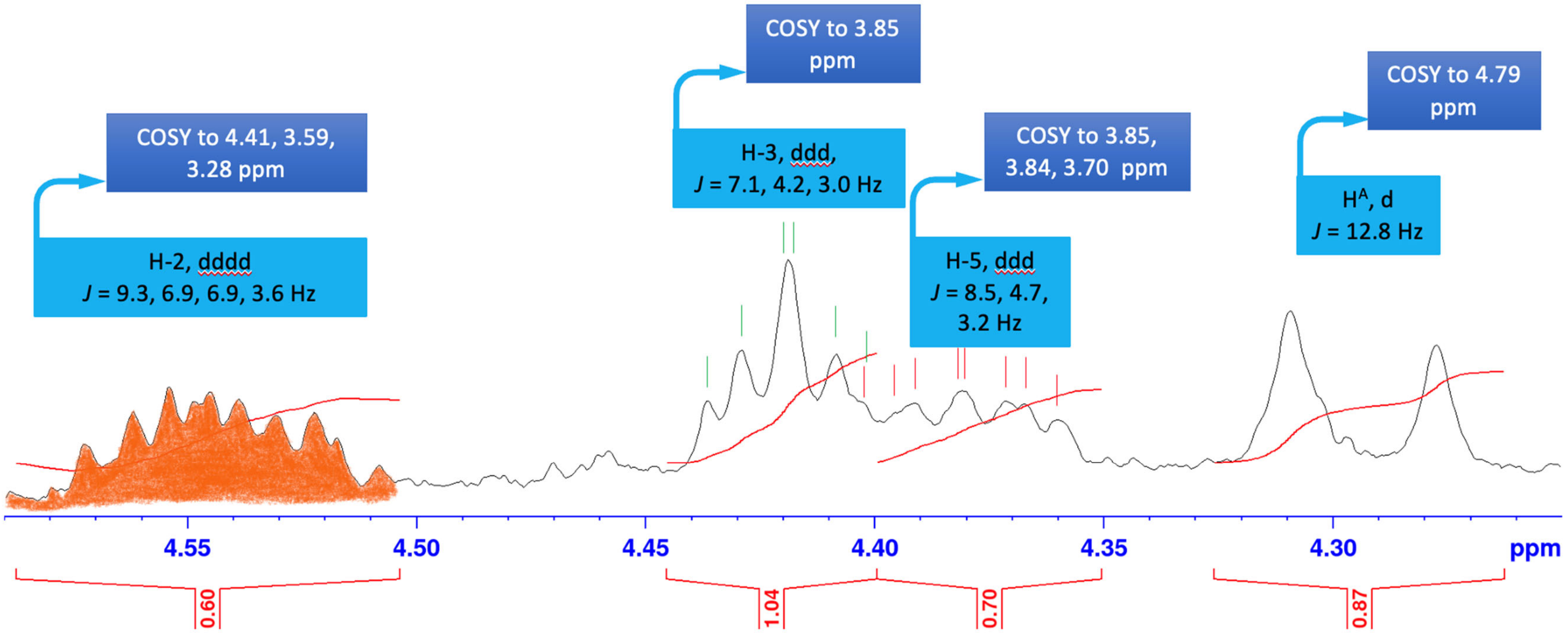

B

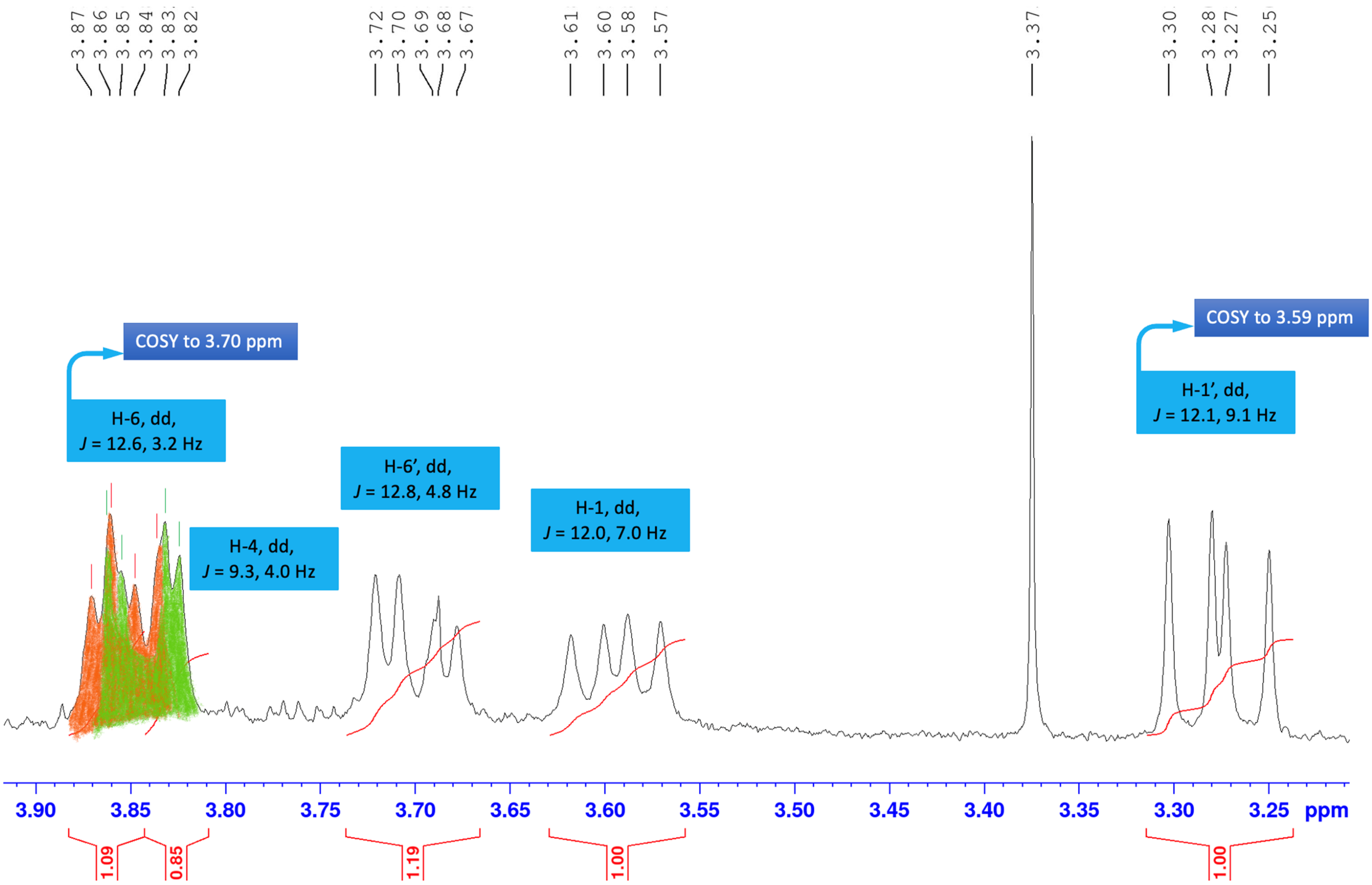
3.87
3.86
3.85
3.84
3.83
3.82
3.72
3.70
3.69
3.68
3.67
3.61
3.60
3.58
3.57
3.37
3.30
3.28
3.27
3.25
COSY to 3.70 ppm
H-6, dd,
J = 12.6, 3.2 Hz
H-6', dd,
J = 12.8, 4.8 Hz
H-1, dd,
J = 12.0, 7.0 Hz
H-4, dd,
J = 9.3, 4.0 Hz
COSY to 3.59 ppm
H-1', dd,
J = 12.1, 9.1 Hz
3.90
3.85
3.80
3.75
3.70
3.65
3.60
3.55
3.50
3.45
3.40
3.35
3.30
3.25
ppm
1.09
0.85
1.19
1.00
1.00

Figure S8. ^{1}H- (400 MHz), ^{13}C-NMR (100 MHz), DEPT, ^{11}B-NMR (128 MHz), COSY, HSQC and HMBC spectra of *N*-(4-methylphenylboronic acid pinacol ester)-3,6-dideoxy-3,6-imino-1,2-*O*-isopropylidene-*α*-D-gulofuranose **para 6** in $CDCl_3$.

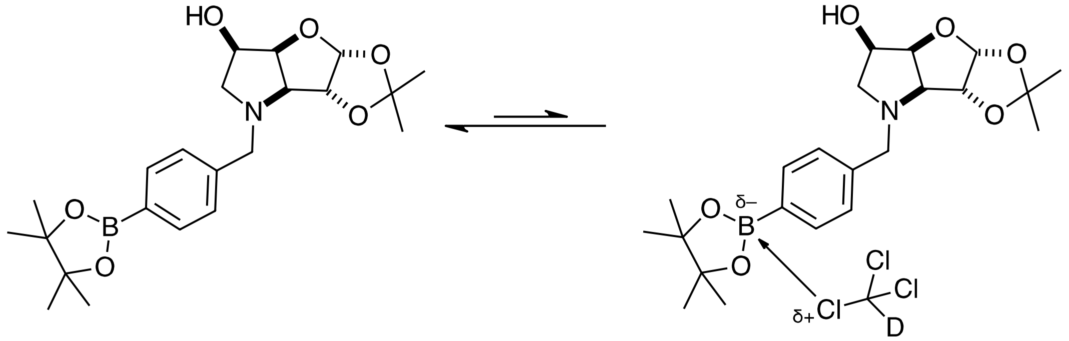

^{1}H-NMR

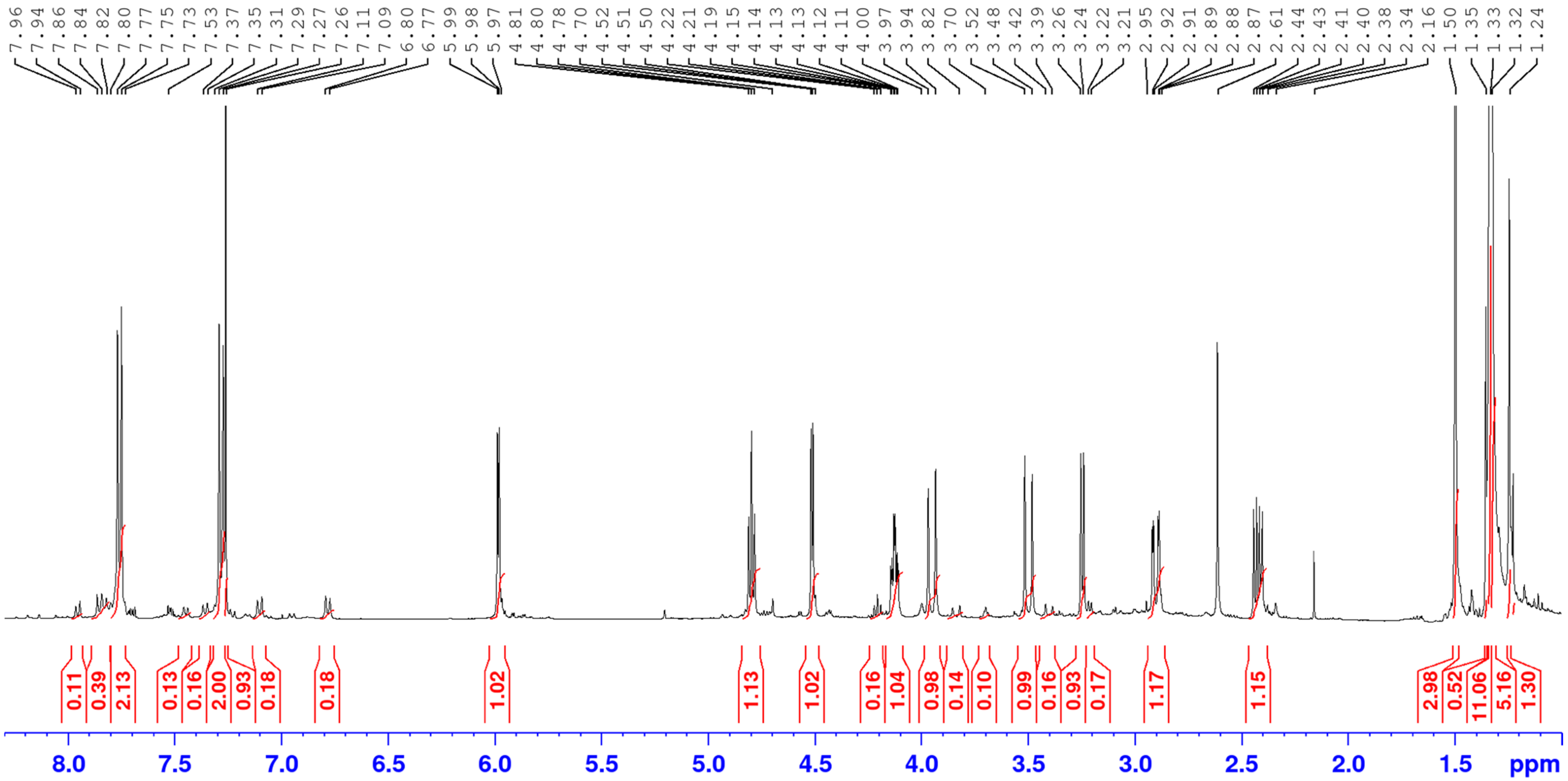

Excerpt of the ^{1}H-NMR spectrum of *N*-(4-methylphenylboronic acid pinacol ester)-3,6-dideoxy-3,6-imino-1,2-*O*-isopropylidene-α-D-gulofuranose **para 6** showing the aromatic region with the two main doublets at 7.76 and 7.28 ppm corresponding to the main Bpin species (trigonal planar) and the doublets belonging to the minor species.

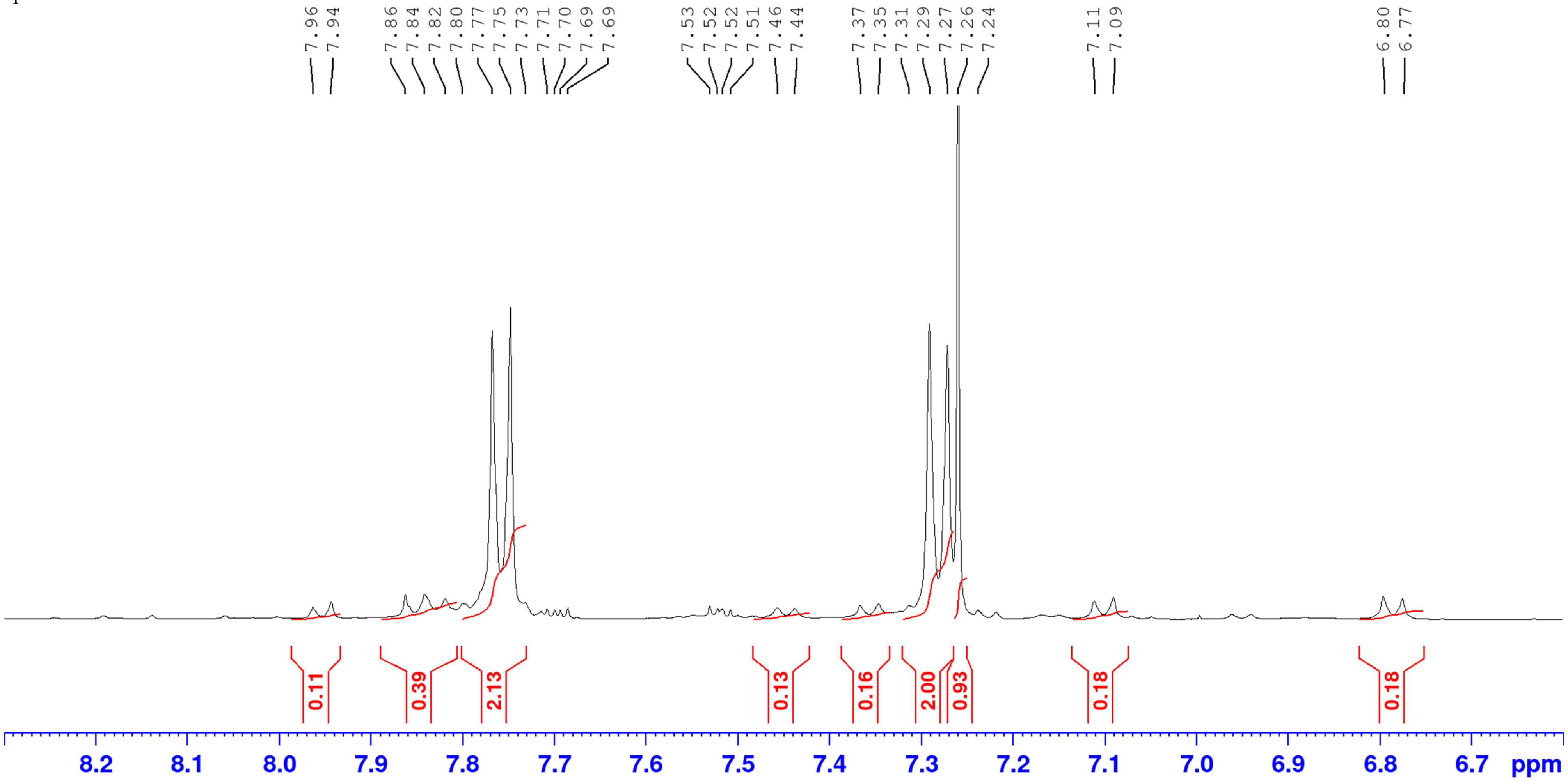

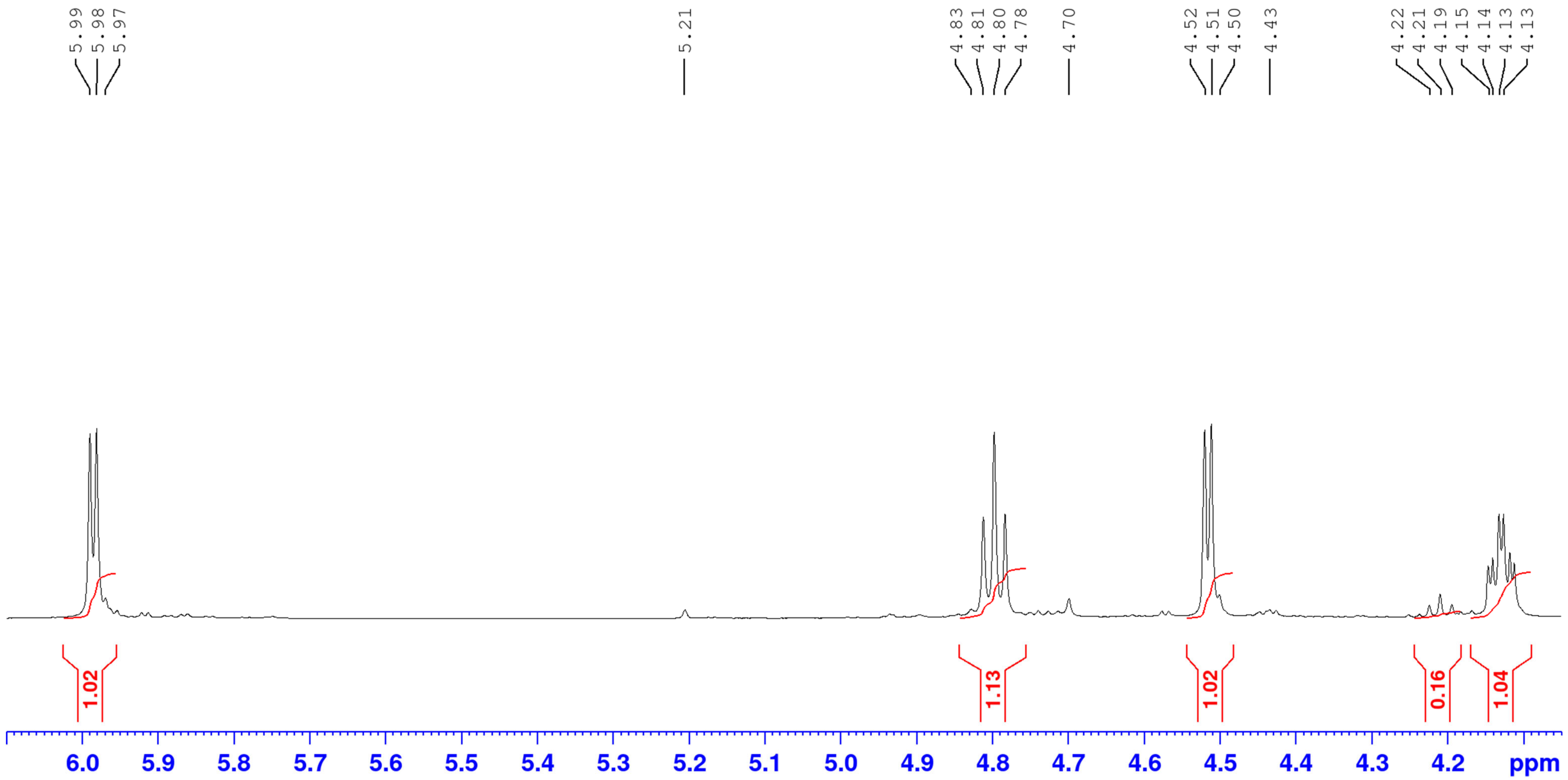

5.99
5.98
5.97
5.21
4.83
4.81
4.80
4.78
4.70
4.52
4.51
4.50
4.43
4.22
4.21
4.19
4.15
4.14
4.13
4.13
1.02
1.13
1.02
0.16
1.04
6.0
5.9
5.8
5.7
5.6
5.5
5.4
5.3
5.2
5.1
5.0
4.9
4.8
4.7
4.6
4.5
4.4
4.3
4.2
ppm

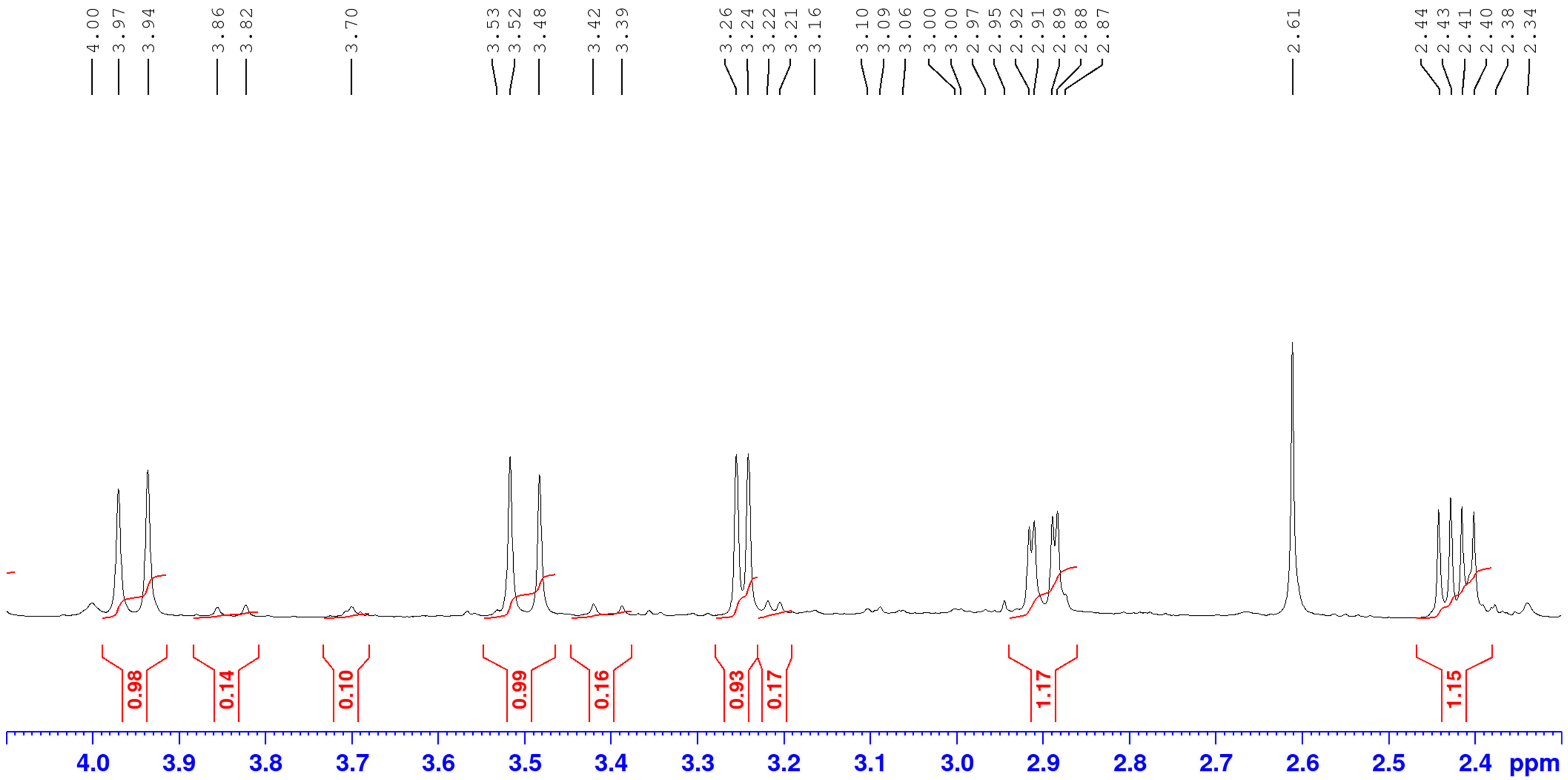
4.00
3.97
3.94
3.86
3.82
3.70
3.53
3.52
3.48
3.42
3.39
3.26
3.24
3.22
3.21
3.16
3.10
3.09
3.06
3.00
3.00
2.97
2.95
2.92
2.91
2.89
2.88
2.87
2.61
2.44
2.43
2.41
2.40
2.38
2.34
0.98
0.14
0.10
0.99
0.16
0.93
0.17
1.17
1.15
4.0
3.9
3.8
3.7
3.6
3.5
3.4
3.3
3.2
3.1
3.0
2.9
2.8
2.7
2.6
2.5
2.4
ppm

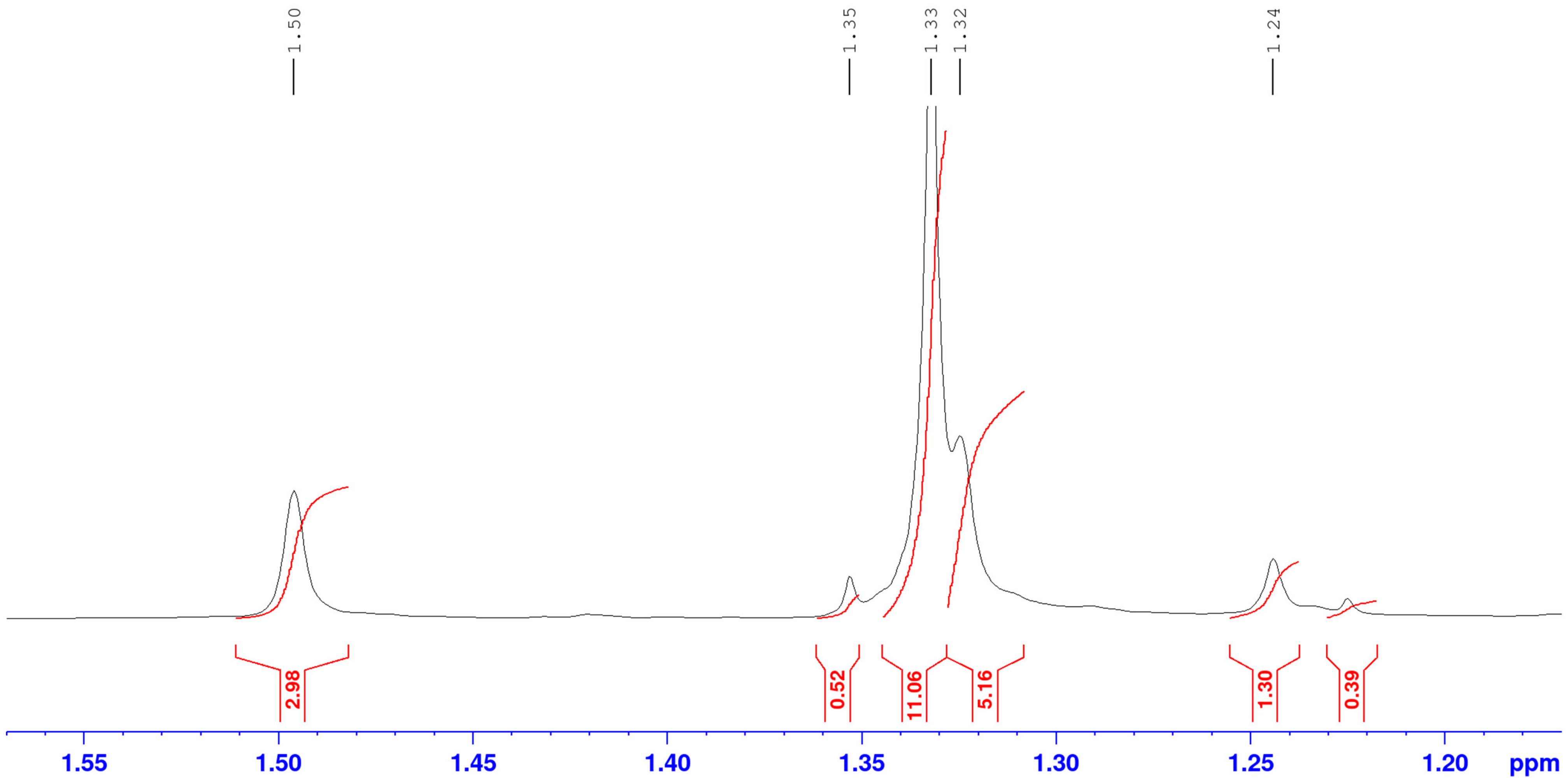
1.50
1.35
1.33
1.32
1.24
2.98
0.52
11.06
5.16
1.30
0.39
1.55
1.50
1.45
1.40
1.35
1.30
1.25
1.20
ppm

^{13}C-NMR

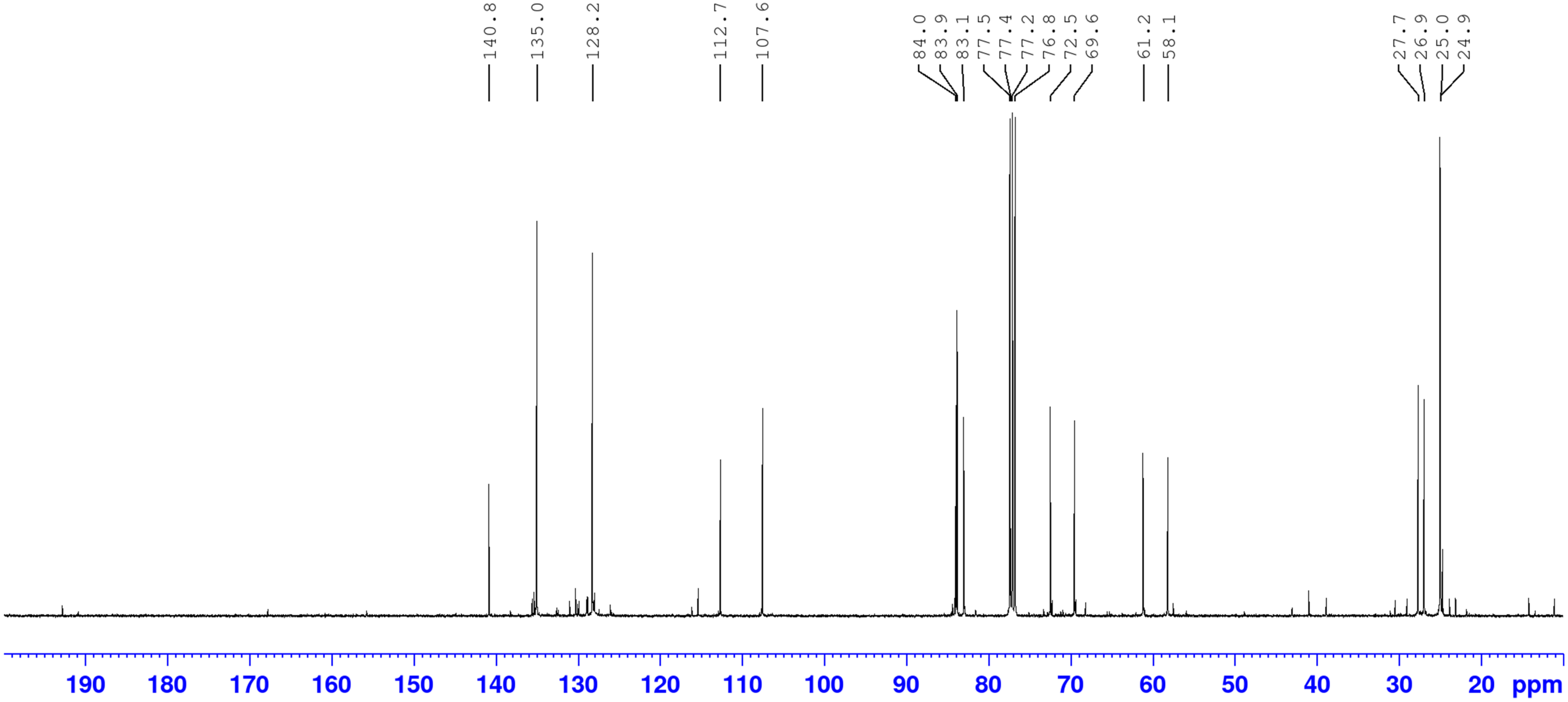
140.8
135.0
128.2
112.7
107.6
84.0
83.9
83.1
77.5
77.4
77.2
76.8
72.5
69.6
61.2
58.1
27.7
26.9
25.0
24.9
190 180 170 160 150 140 130 120 110 100 90 80 70 60 50 40 30 20 ppm

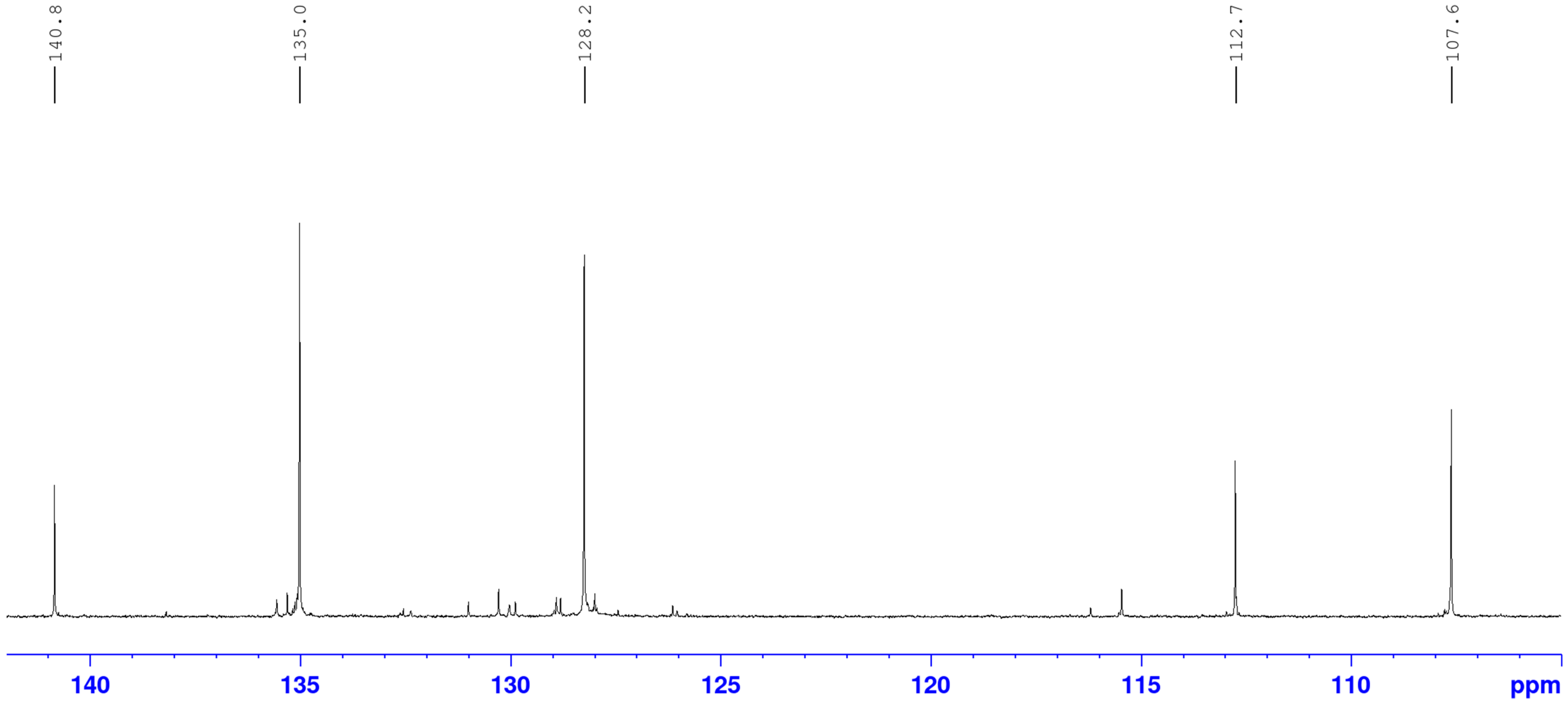

140.8
135.0
128.2
112.7
107.6
140
135
130
125
120
115
110
ppm

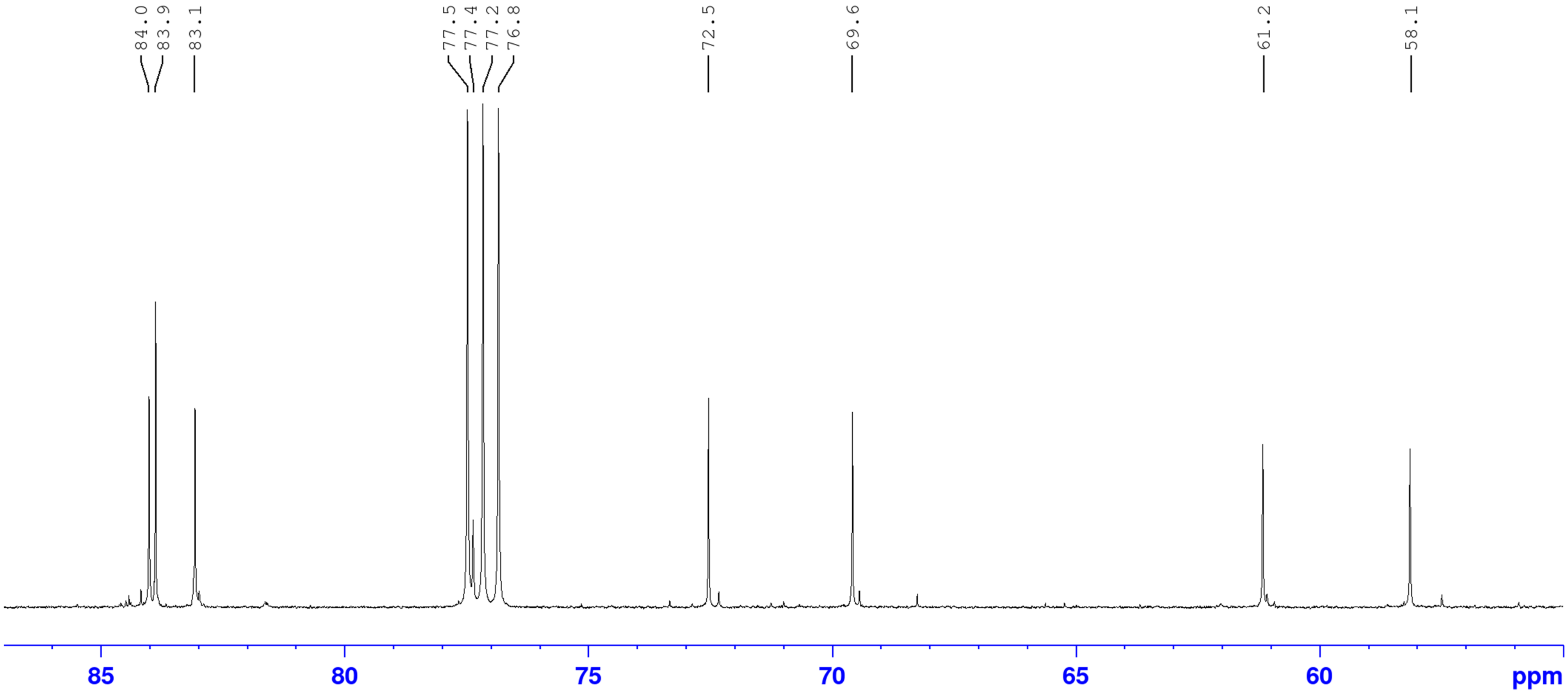
84.0
83.9
83.1
77.5
77.4
77.2
76.8
72.5
69.6
61.2
58.1
85
80
75
70
65
60
ppm

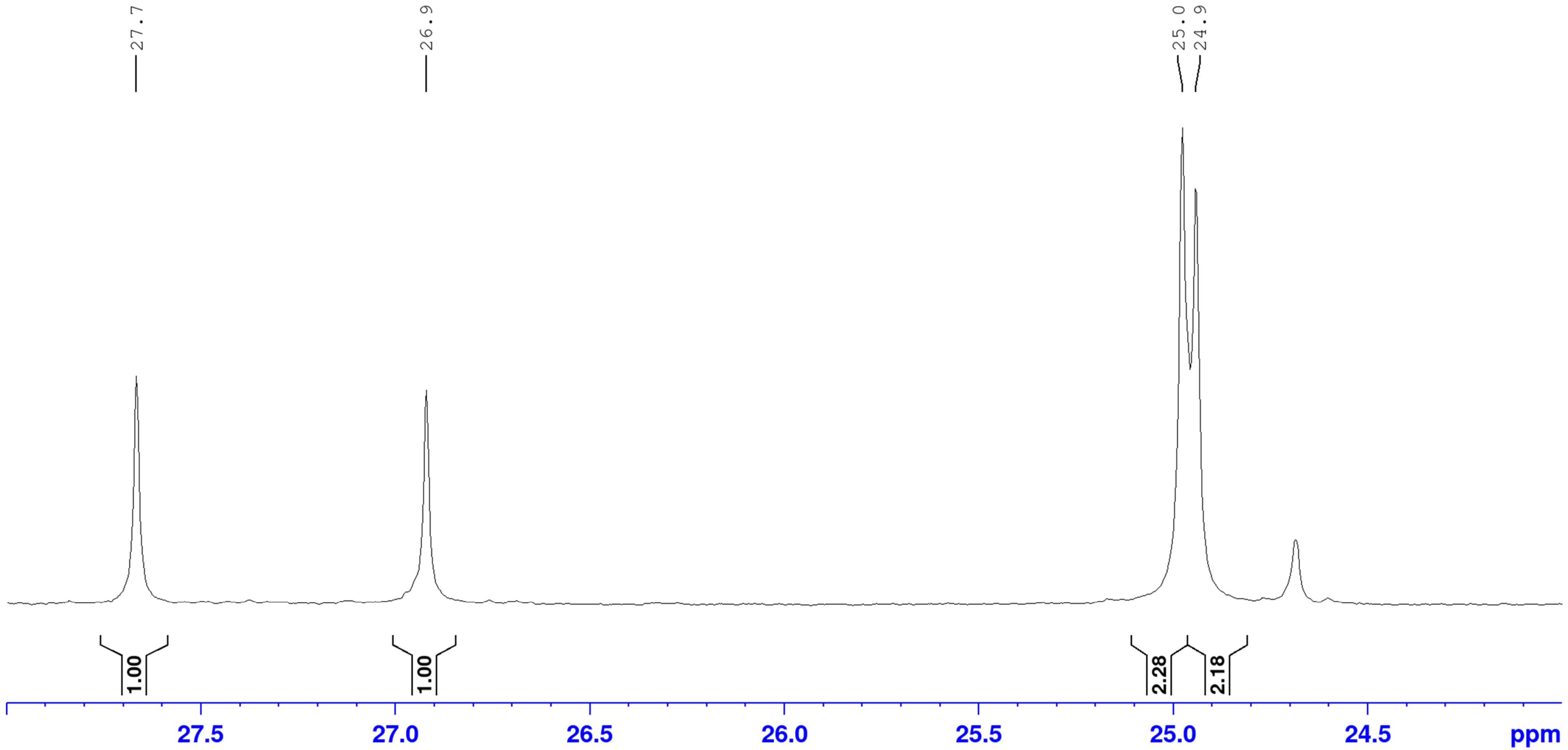
27.7
26.9
25.0
24.9
1.00
1.00
2.28
2.18
27.5
27.0
26.5
26.0
25.5
25.0
24.5
ppm

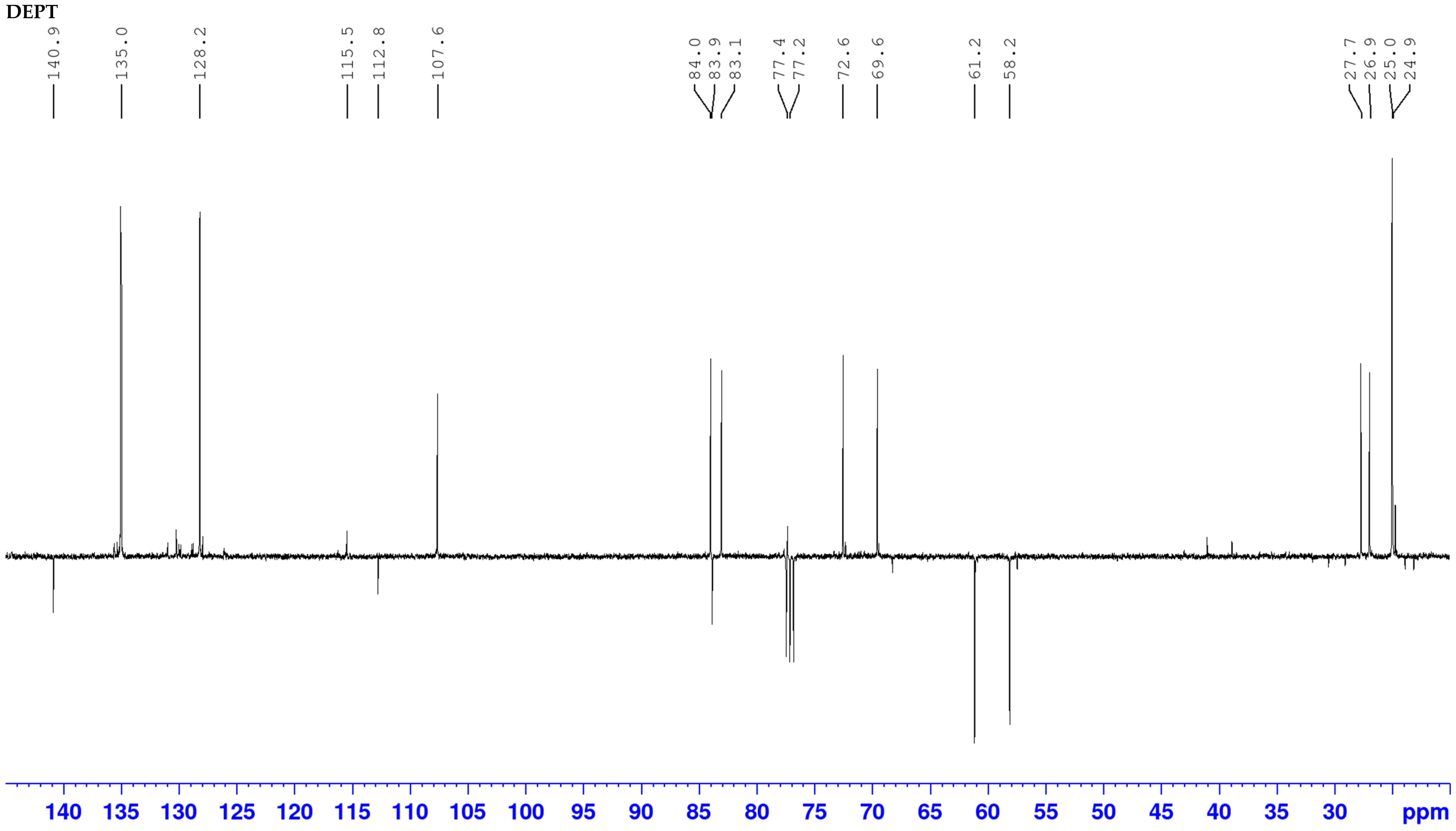
DEPT
140.9
135.0
128.2
115.5
112.8
107.6
84.0
83.9
83.1
77.4
77.2
72.6
69.6
61.2
58.2
27.7
26.9
25.0
24.9
140 135 130 125 120 115 110 105 100 95 90 85 80 75 70 65 60 55 50 45 40 35 30 ppm

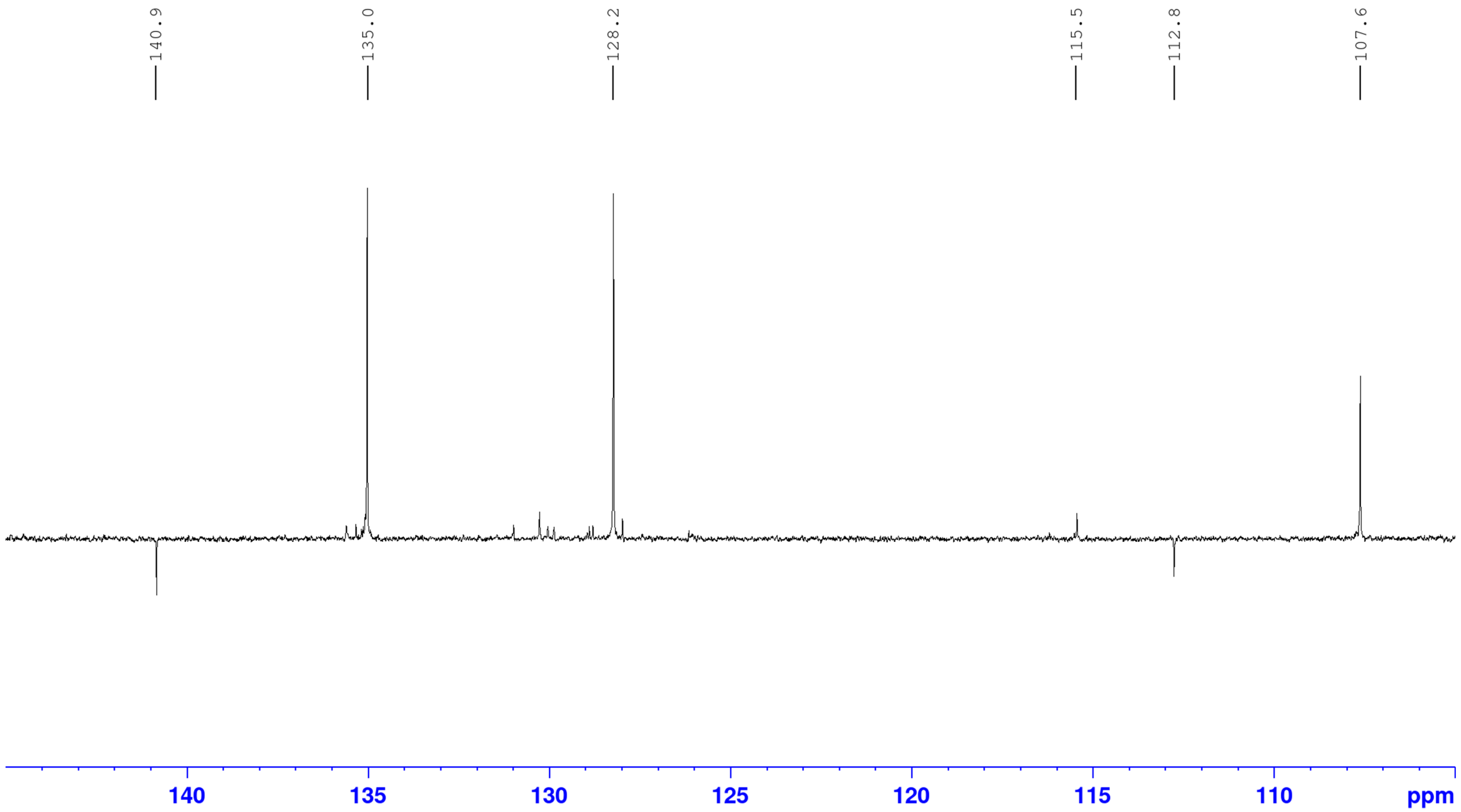

140.9
135.0
128.2
115.5
112.8
107.6
140
135
130
125
120
115
110
ppm

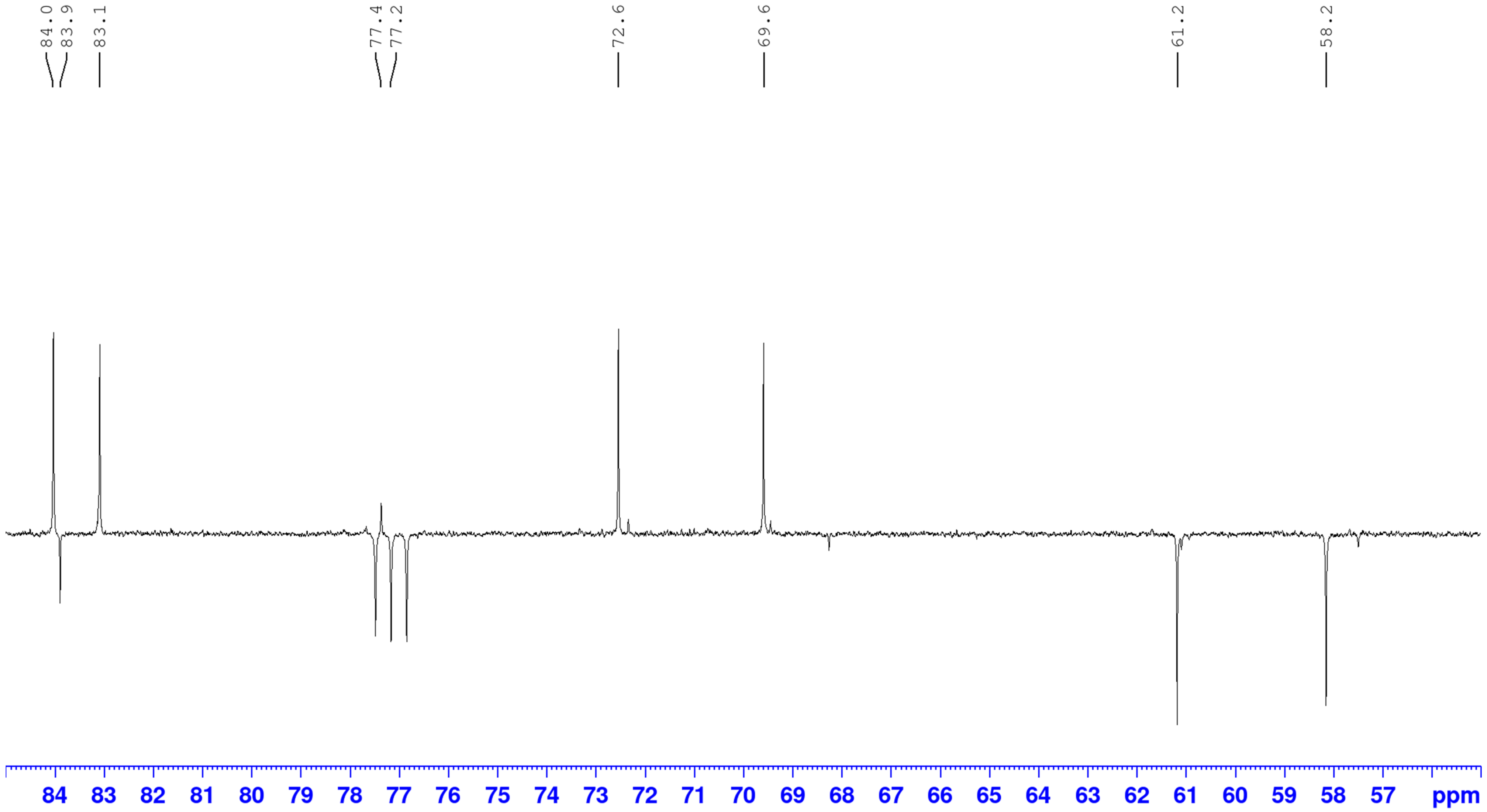
84.0
83.9
83.1
77.4
77.2
72.6
69.6
61.2
58.2
84 83 82 81 80 79 78 77 76 75 74 73 72 71 70 69 68 67 66 65 64 63 62 61 60 59 58 57 ppm

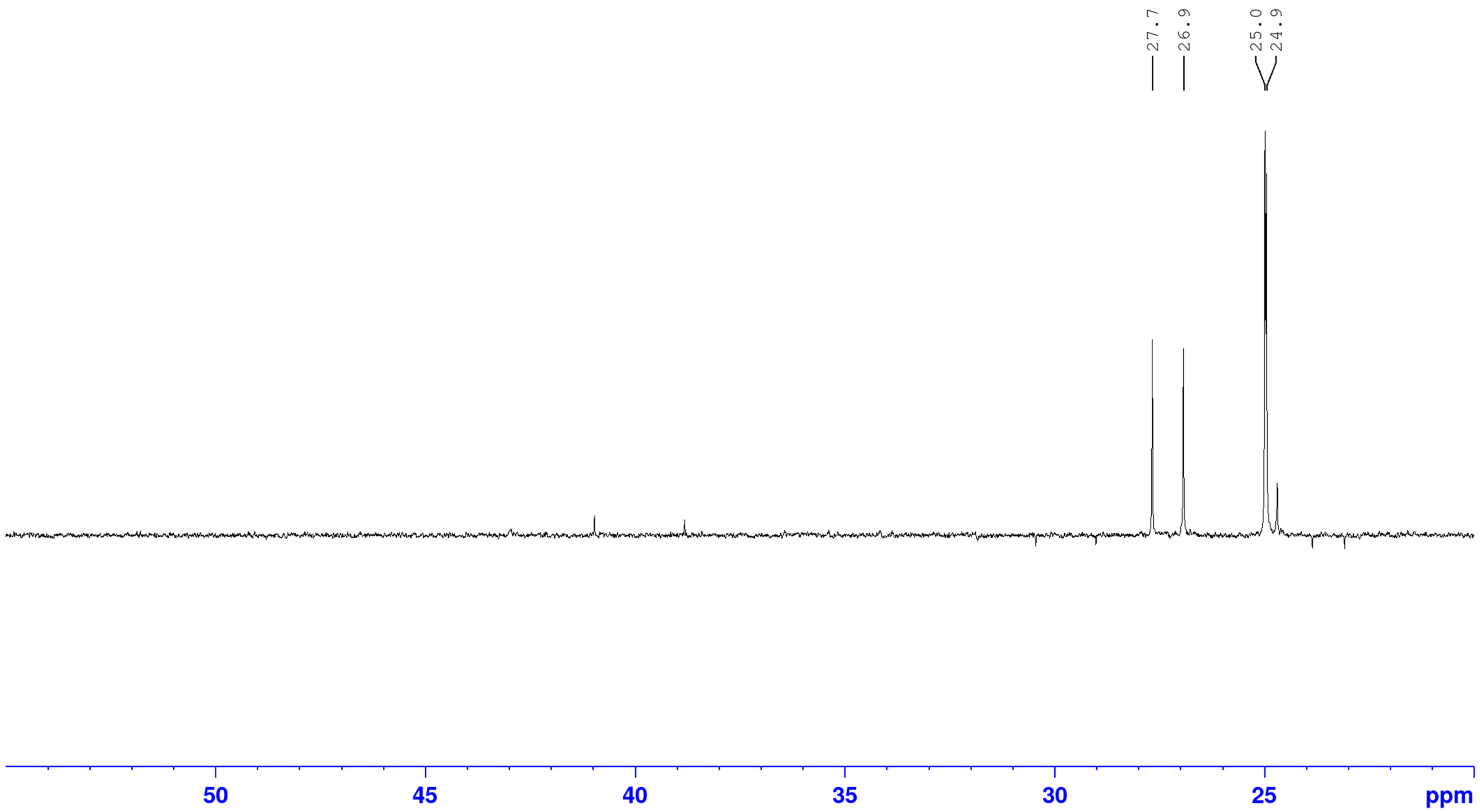
27.7
26.9
25.0
24.9
50
45
40
35
30
25
ppm

^{11}B-NMR

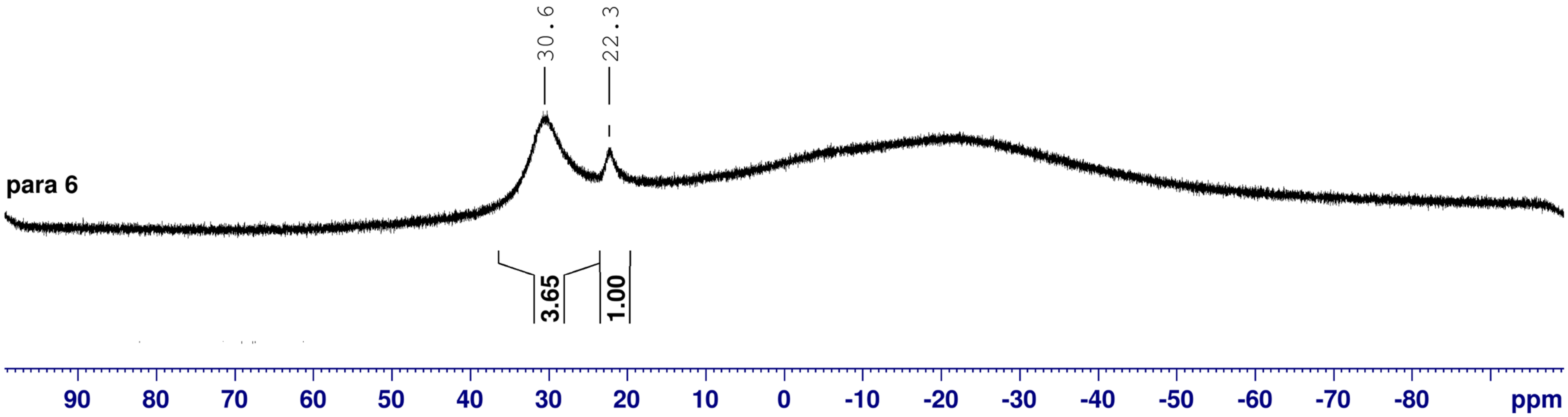
30.6
22.3
para 6
3.65
1.00
90
80
70
60
50
40
30
20
10
0
-10
-20
-30
-40
-50
-60
-70
-80
ppm

COSY

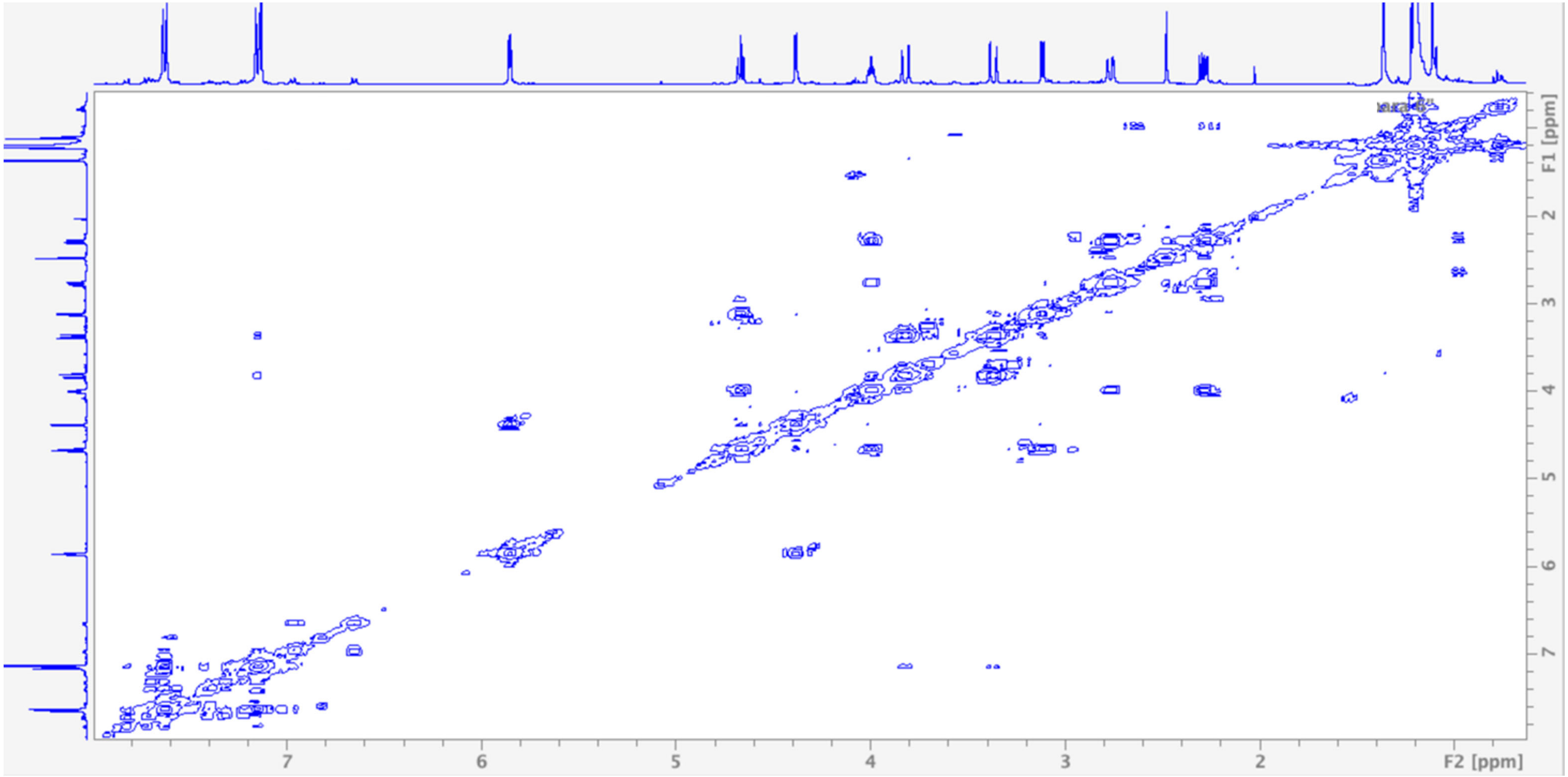

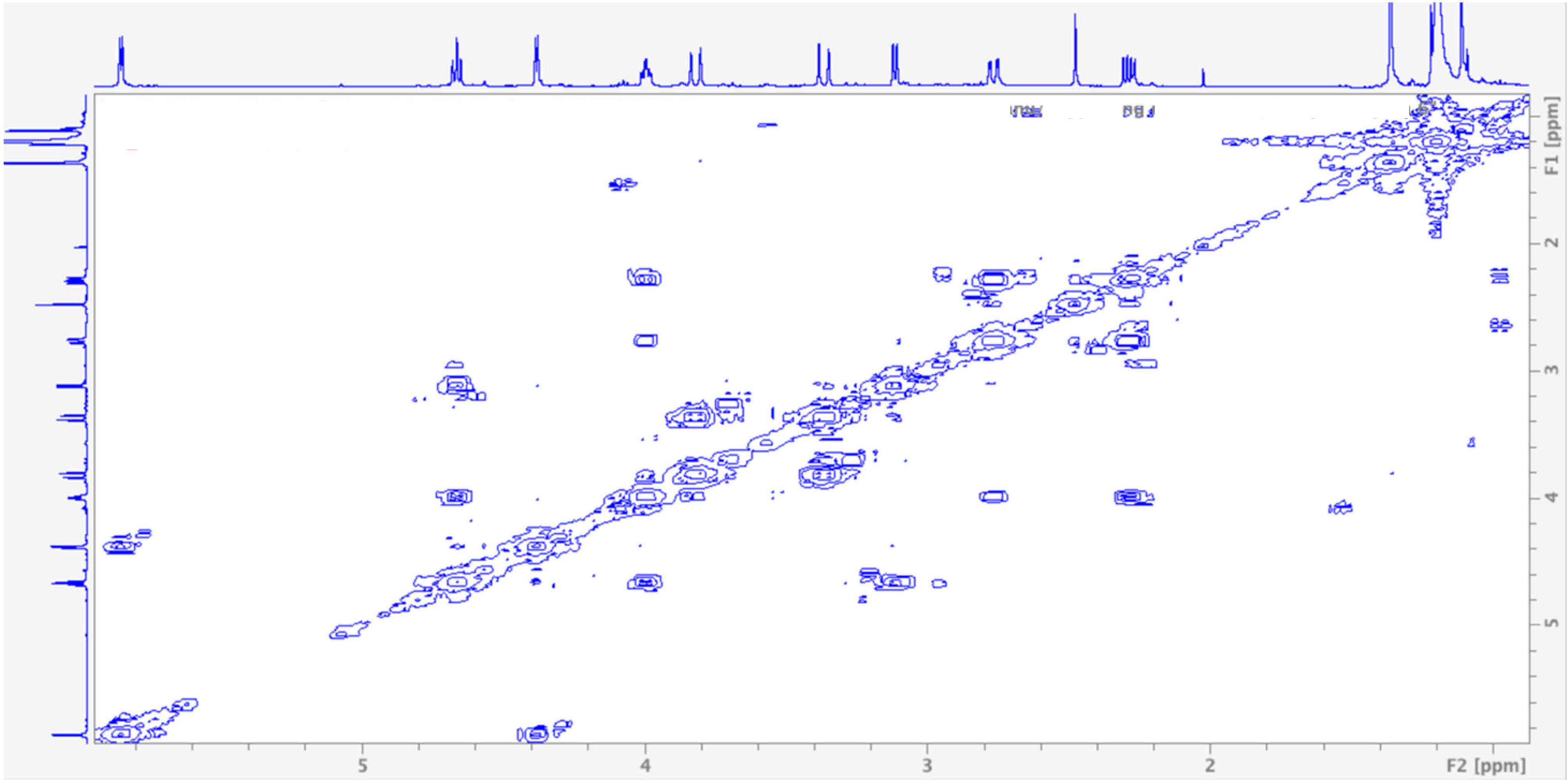
F1 [ppm]
2
3
4
5
5
4
3
2
F2 [ppm]

HSQC

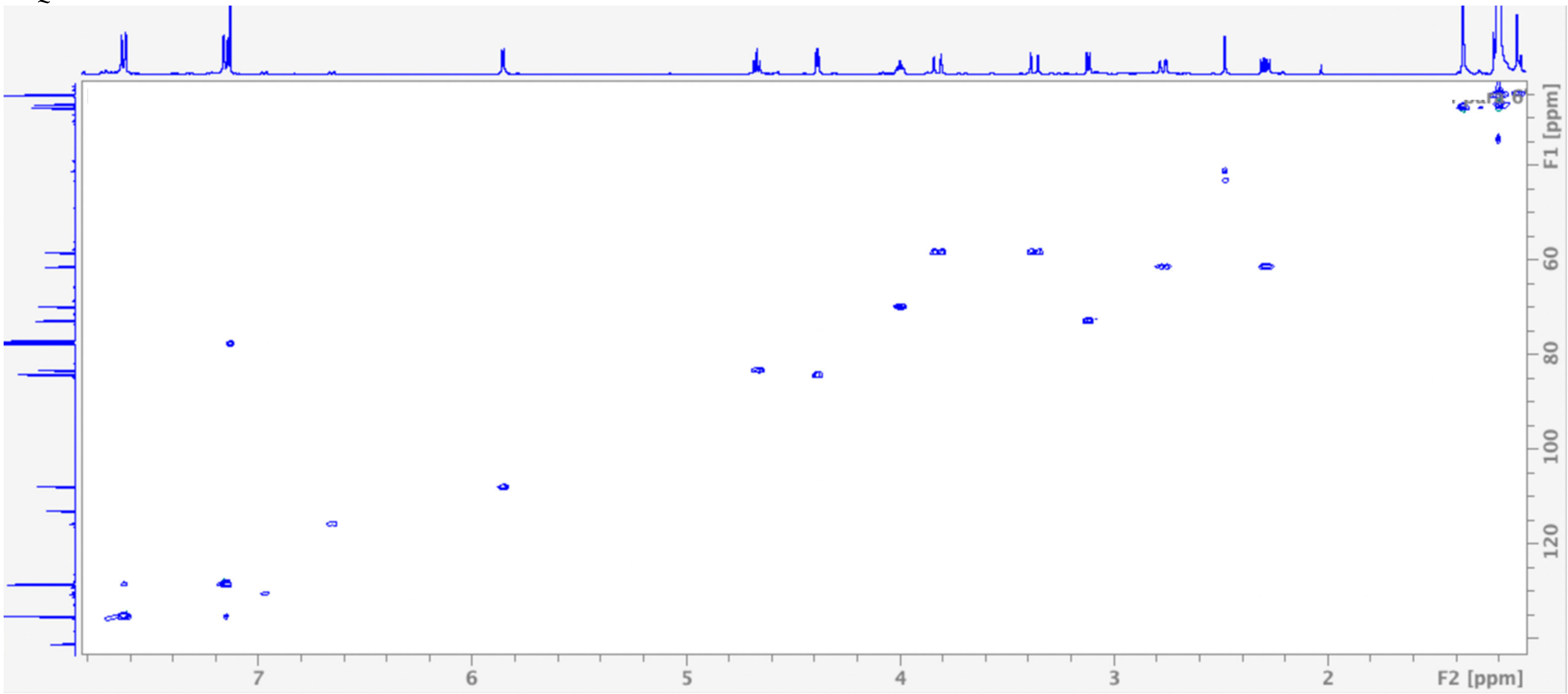

HMBC

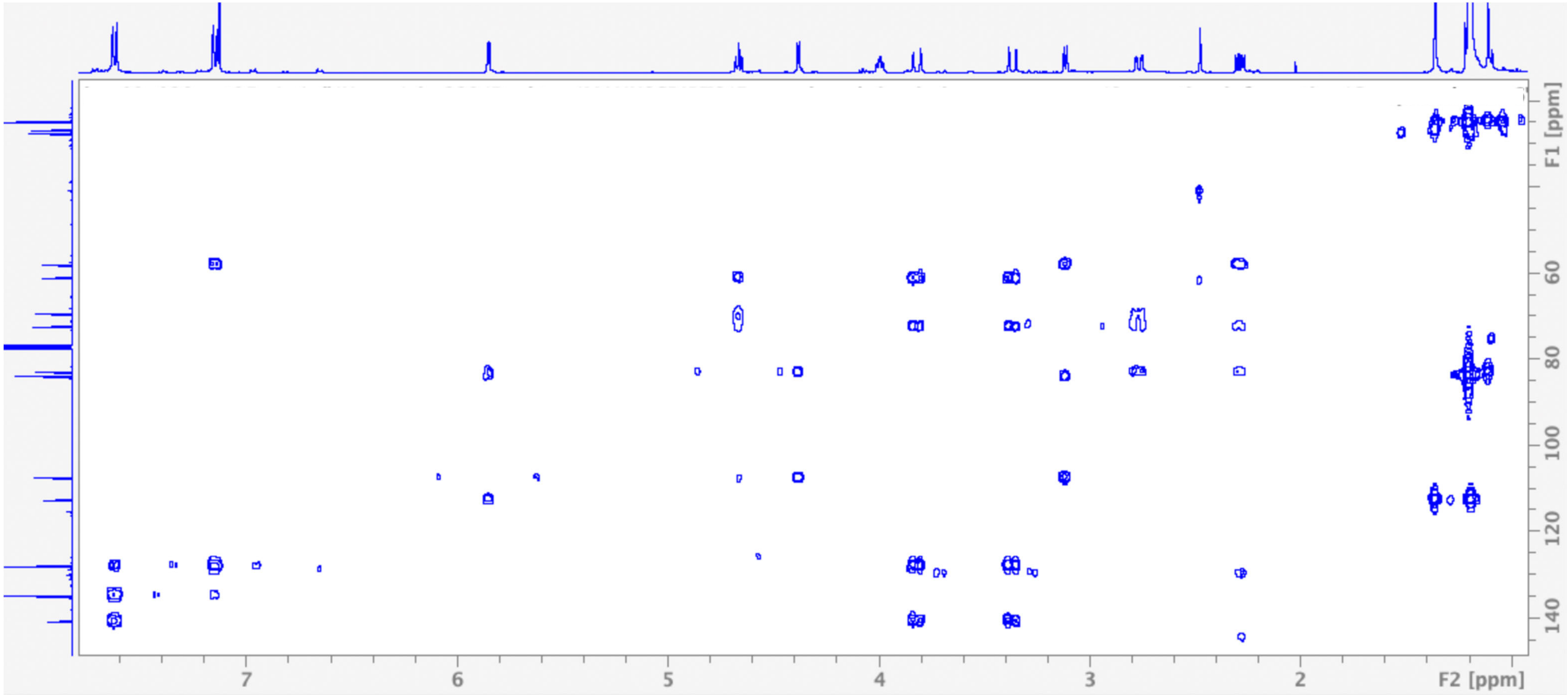

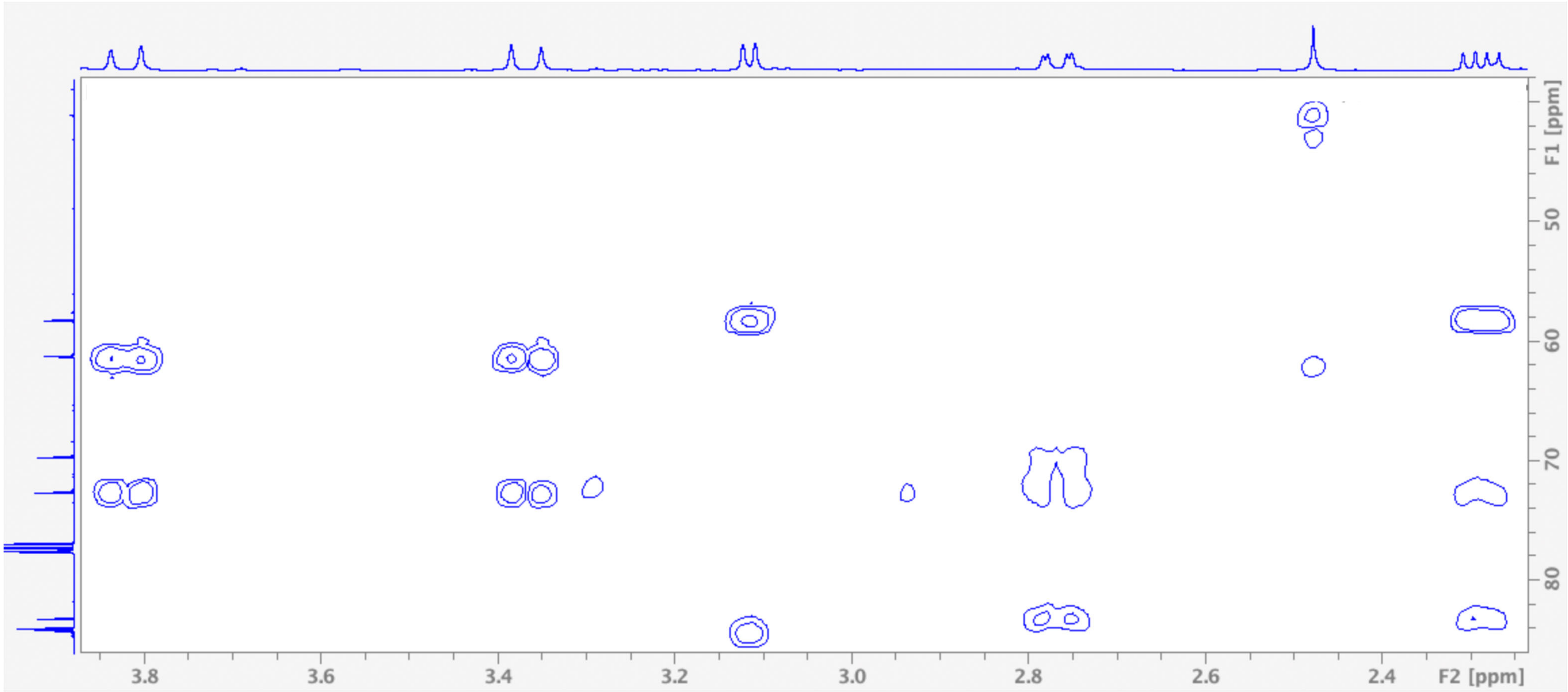
F1 [ppm]
50
60
70
80
3.8
3.6
3.4
3.2
3.0
2.8
2.6
2.4
F2 [ppm]

Figure S9. ^{1}H- (400 MHz), ^{13}C-NMR (100 MHz), ^{11}B-NMR (128 MHz), COSY, HSQC and HMBC spectra of *N*-(4-methylphenyl boronic acid)-3,6-dideoxy-3,6-imino-D-gulofuranose **para 7** in D_2O.

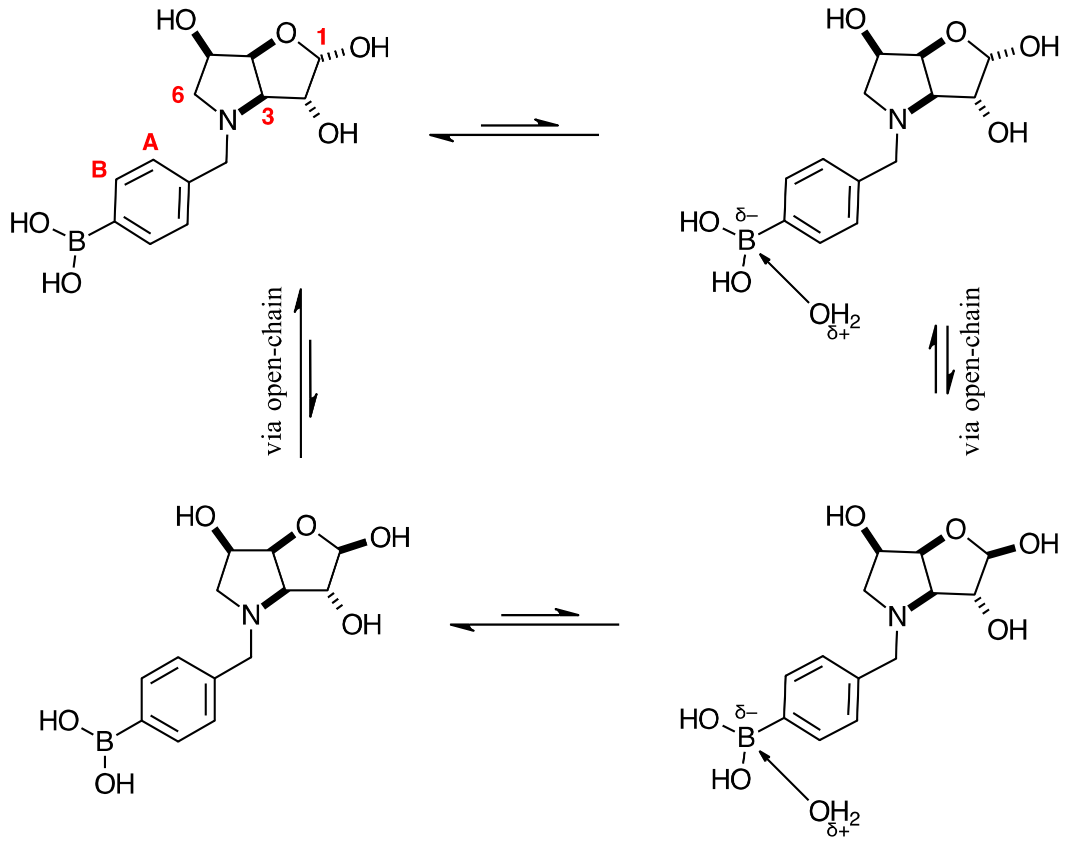

^{1}H-NMR

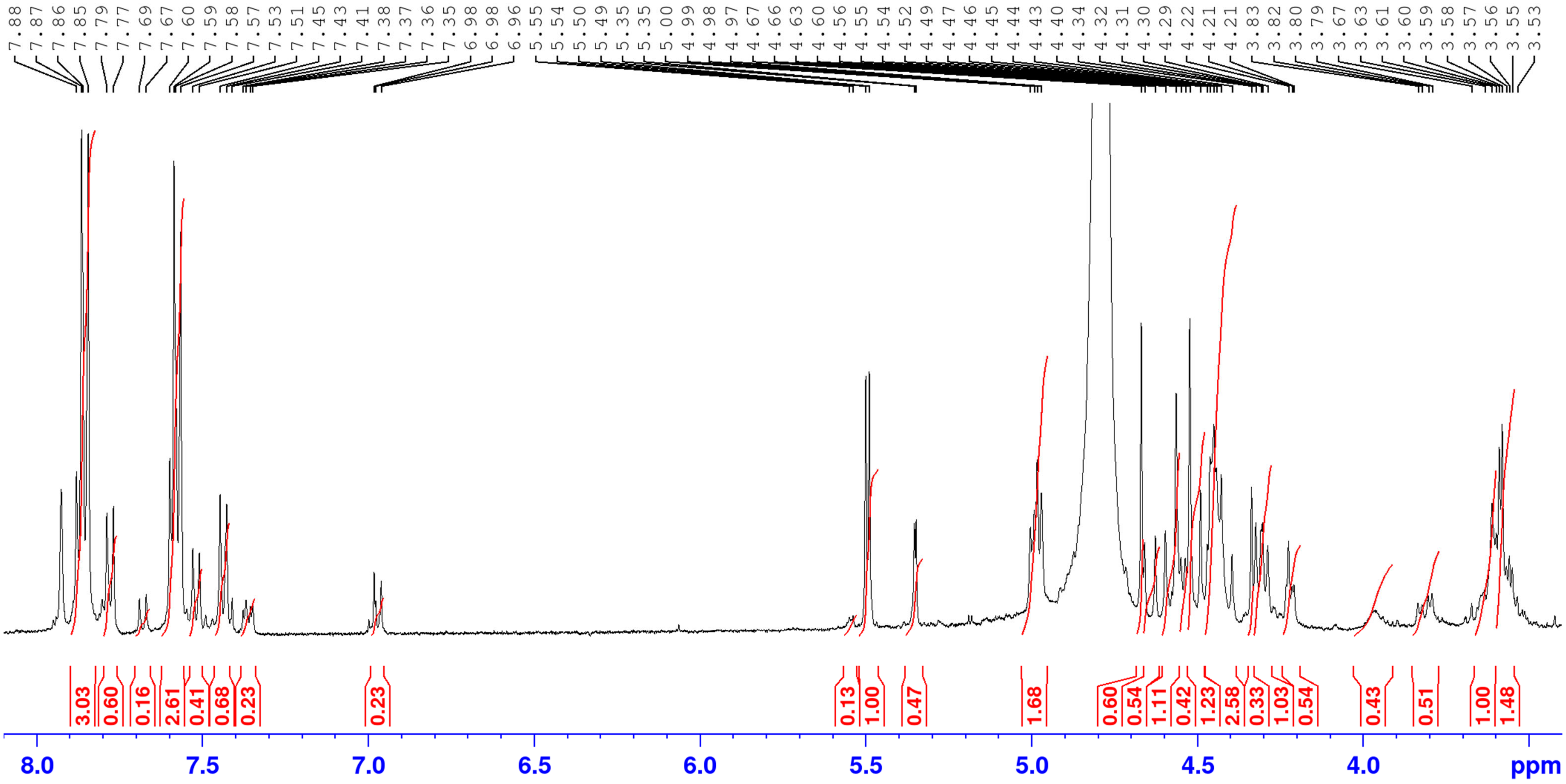

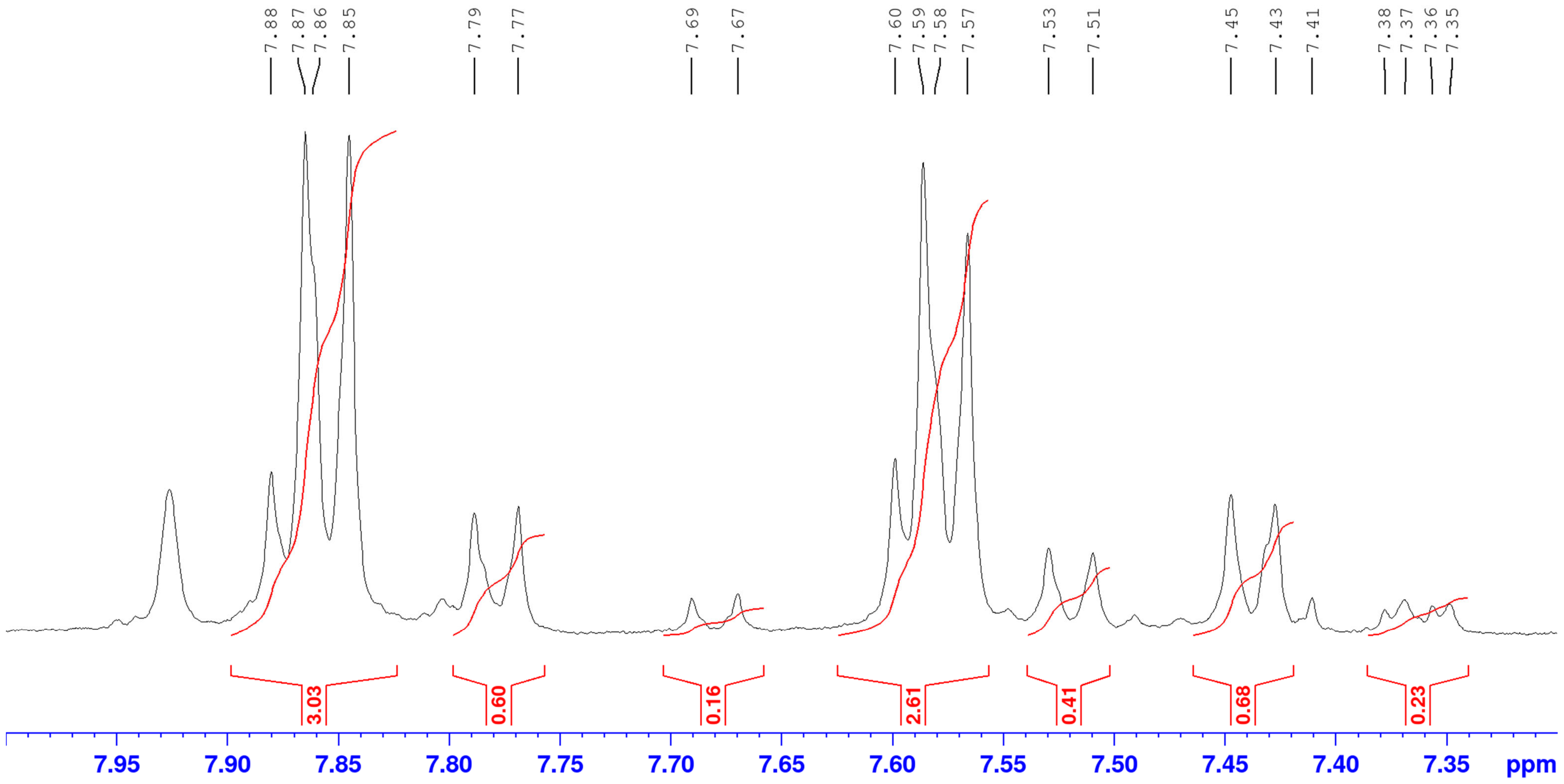
7.88
7.87
7.86
7.85
7.79
7.77
7.69
7.67
7.60
7.59
7.58
7.57
7.53
7.51
7.45
7.43
7.41
7.38
7.37
7.36
7.35
3.03
0.60
0.16
2.61
0.41
0.68
0.23
7.95
7.90
7.85
7.80
7.75
7.70
7.65
7.60
7.55
7.50
7.45
7.40
7.35
ppm

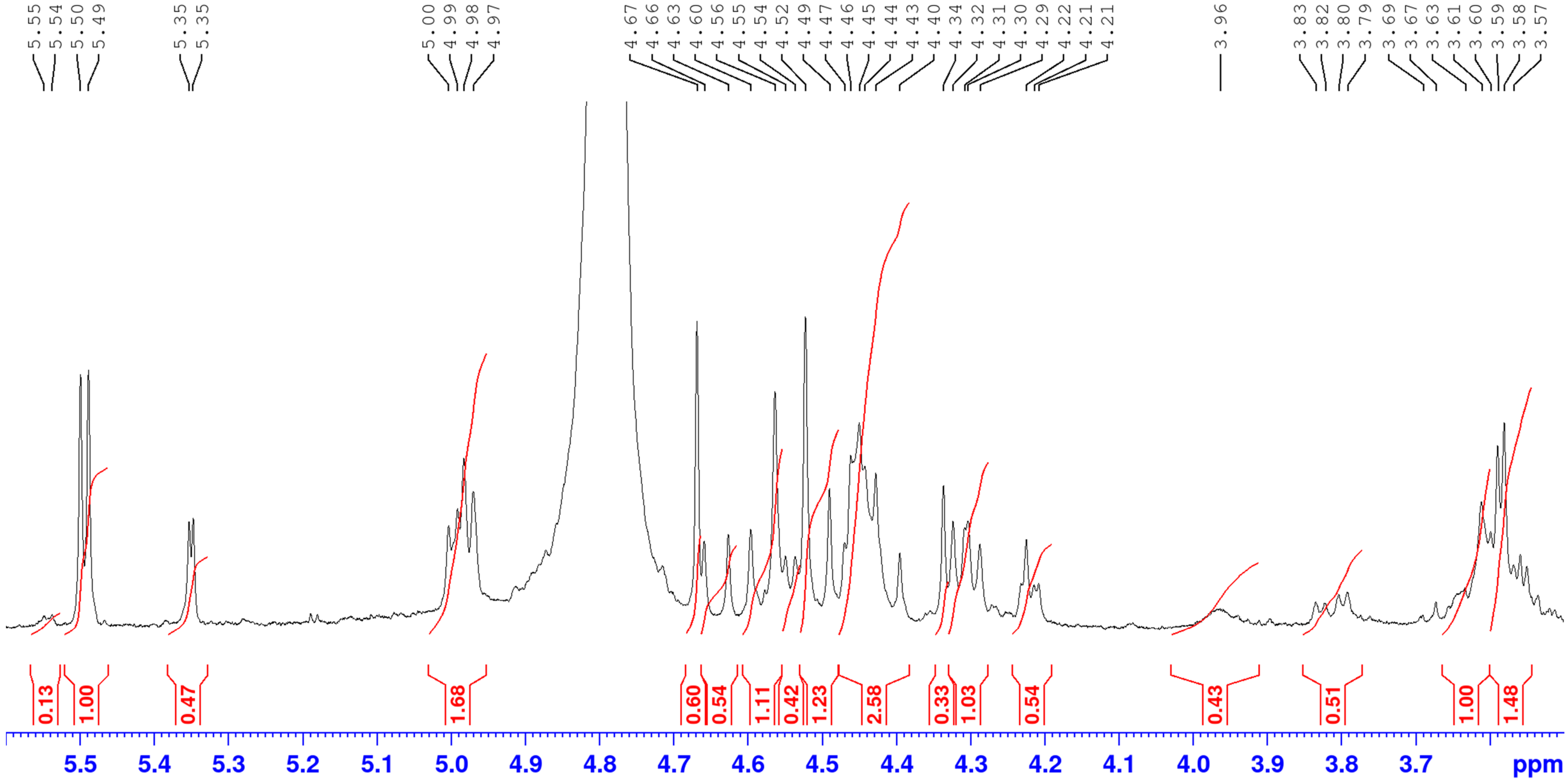
5.55 5.54 5.50 5.49 5.35 5.35 5.00 4.99 4.98 4.97 4.67 4.66 4.63 4.60 4.56 4.55 4.54 4.52 4.49 4.47 4.46 4.45 4.44 4.43 4.40 4.34 4.32 4.31 4.30 4.29 4.22 4.21 4.21 3.96 3.83 3.82 3.80 3.79 3.69 3.67 3.63 3.61 3.60 3.59 3.58 3.57
0.13 1.00 0.47 1.68 0.60 0.54 1.11 0.42 1.23 2.58 0.33 1.03 0.54 0.43 0.51 1.00 1.48
5.5 5.4 5.3 5.2 5.1 5.0 4.9 4.8 4.7 4.6 4.5 4.4 4.3 4.2 4.1 4.0 3.9 3.8 3.7 ppm

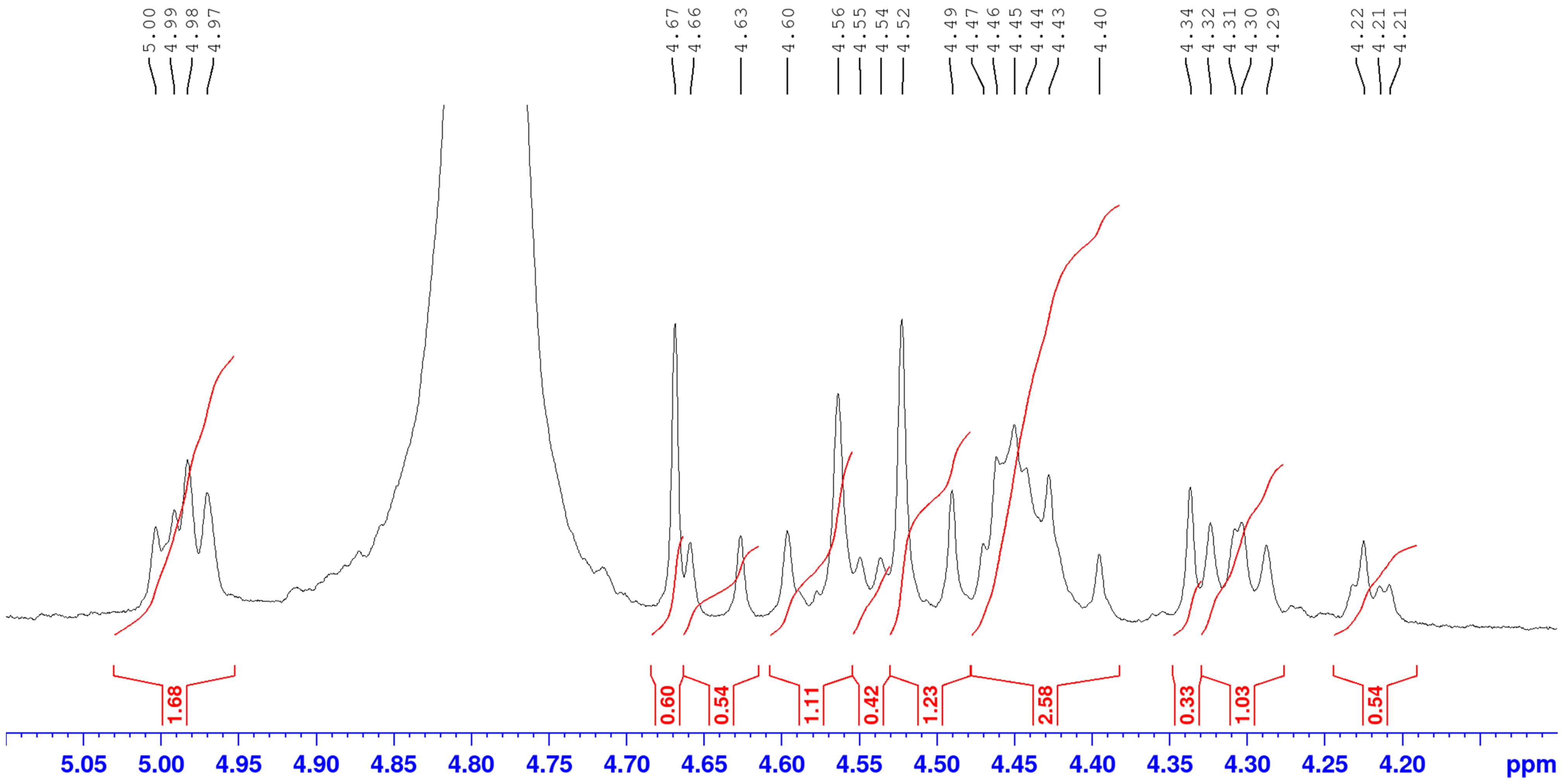
5.00
4.99
4.98
4.97
4.67
4.66
4.63
4.60
4.56
4.55
4.54
4.52
4.49
4.47
4.46
4.45
4.44
4.43
4.40
4.34
4.32
4.31
4.30
4.29
4.22
4.21
4.21
1.68
0.60
0.54
1.11
0.42
1.23
2.58
0.33
1.03
0.54
5.05
5.00
4.95
4.90
4.85
4.80
4.75
4.70
4.65
4.60
4.55
4.50
4.45
4.40
4.35
4.30
4.25
4.20
ppm

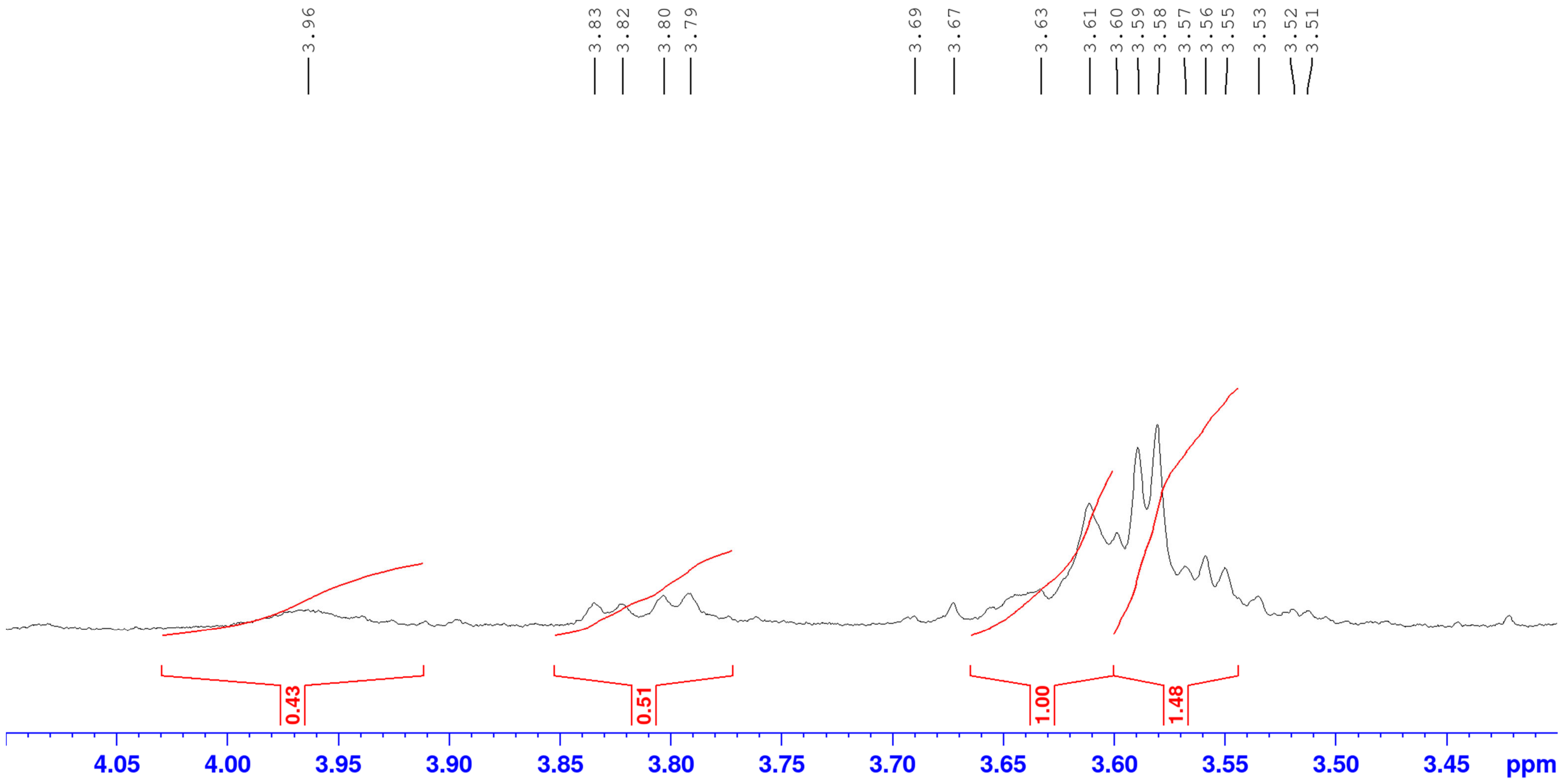
3.96
3.83
3.82
3.80
3.79
3.69
3.67
3.63
3.61
3.60
3.59
3.58
3.57
3.56
3.55
3.53
3.52
3.51
0.43
0.51
1.00
1.48
4.05
4.00
3.95
3.90
3.85
3.80
3.75
3.70
3.65
3.60
3.55
3.50
3.45
ppm

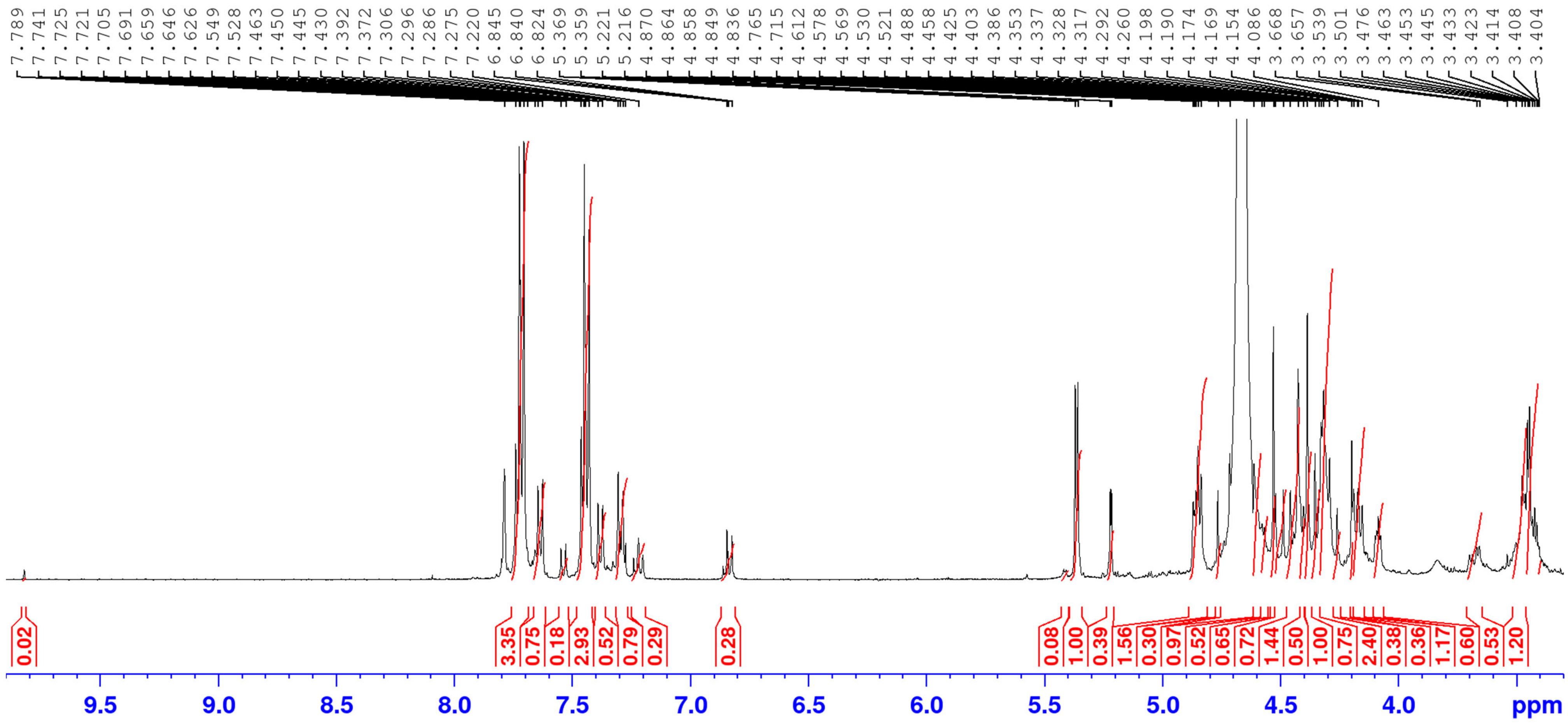
ppm
9.5
9.0
8.5
8.0
7.5
7.0
6.5
6.0
5.5
5.0
4.5
4.0

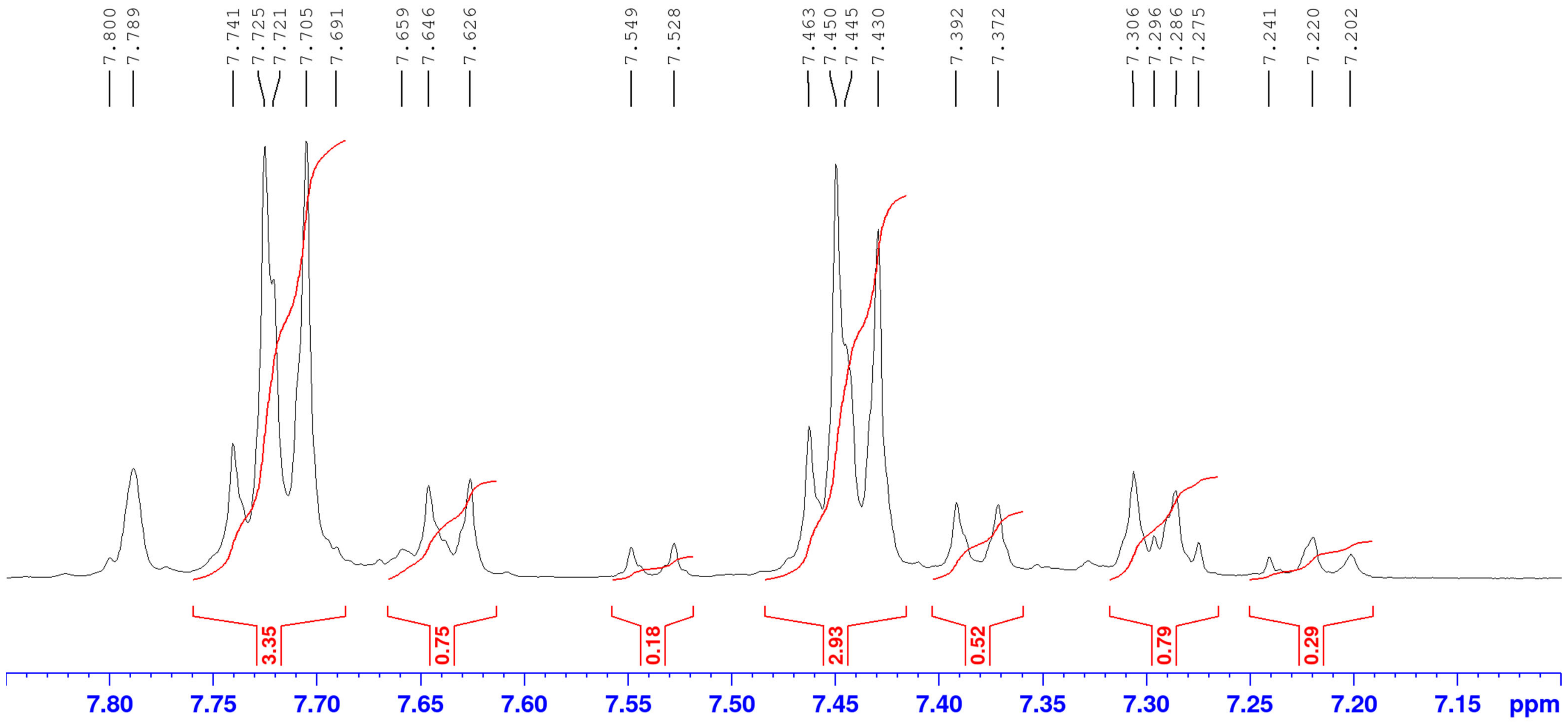

7.800
7.789
7.741
7.725
7.721
7.705
7.691
7.659
7.646
7.626
7.549
7.528
7.463
7.450
7.445
7.430
7.392
7.372
7.306
7.296
7.286
7.275
7.241
7.220
7.202
3.35
0.75
0.18
2.93
0.52
0.79
0.29
7.80
7.75
7.70
7.65
7.60
7.55
7.50
7.45
7.40
7.35
7.30
7.25
7.20
7.15
ppm

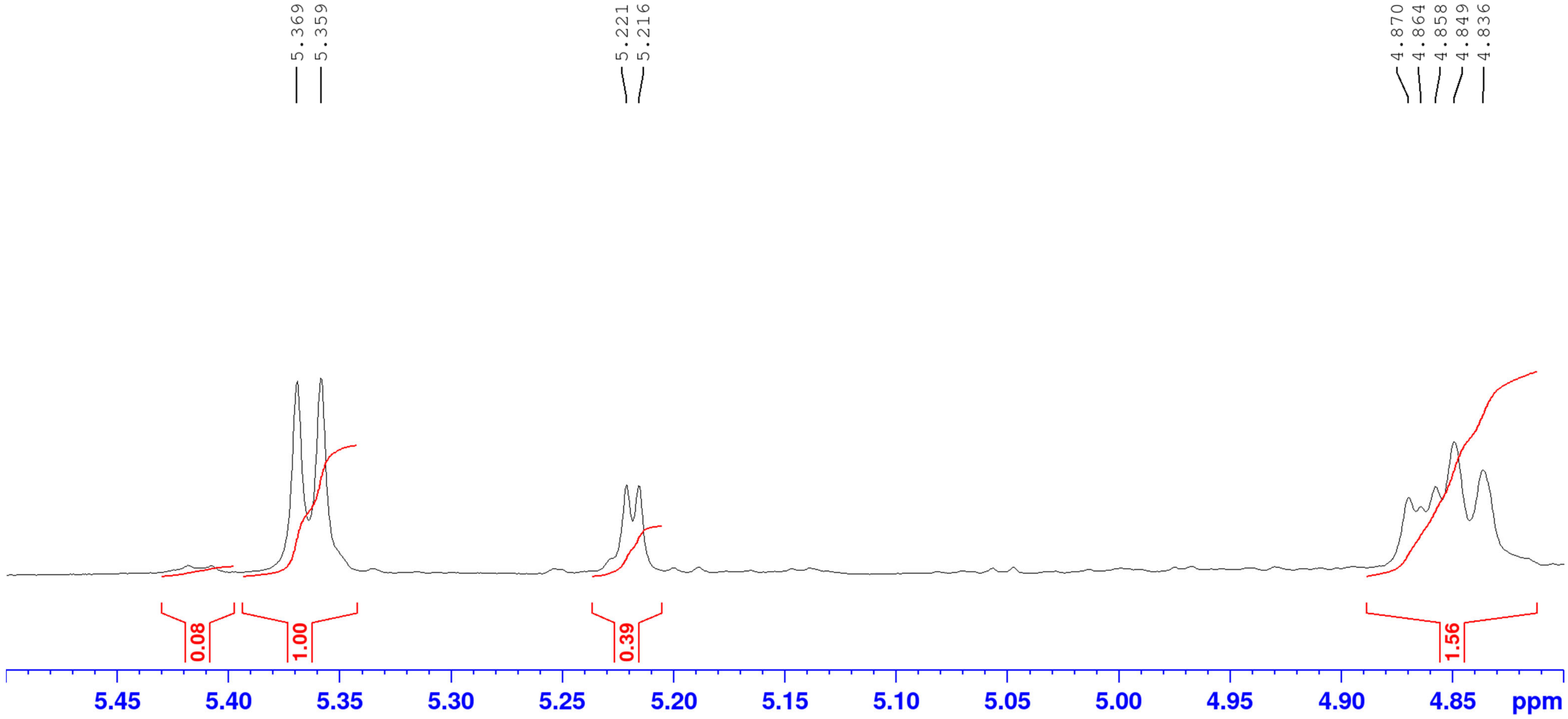
5.369
5.359
5.221
5.216
4.870
4.864
4.858
4.849
4.836
0.08
1.00
0.39
1.56
5.45
5.40
5.35
5.30
5.25
5.20
5.15
5.10
5.05
5.00
4.95
4.90
4.85
ppm

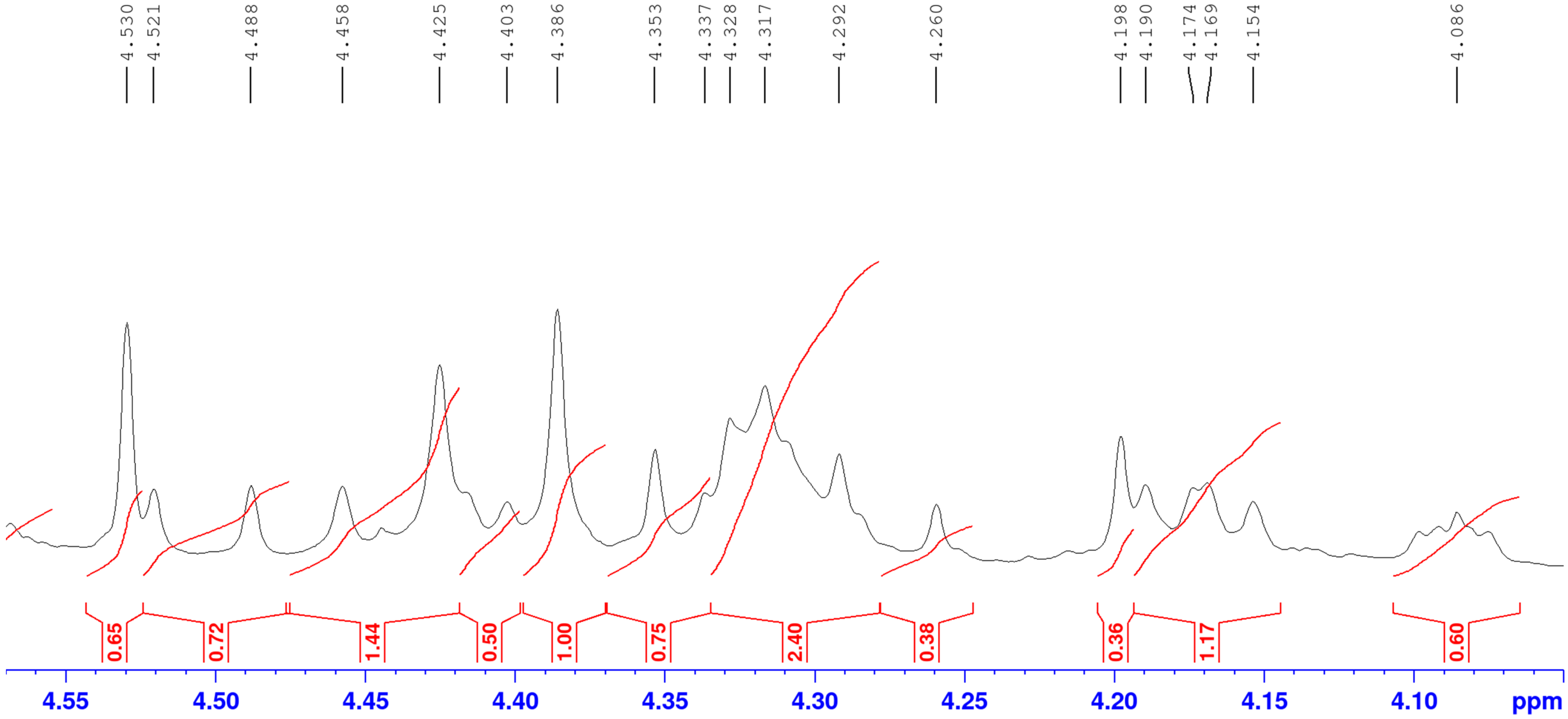
4.530
4.521
4.488
4.458
4.425
4.403
4.386
4.353
4.337
4.328
4.317
4.292
4.260
4.198
4.190
4.174
4.169
4.154
4.086
0.65
0.72
1.44
0.50
1.00
0.75
2.40
0.38
0.36
1.17
0.60
4.55
4.50
4.45
4.40
4.35
4.30
4.25
4.20
4.15
4.10
ppm

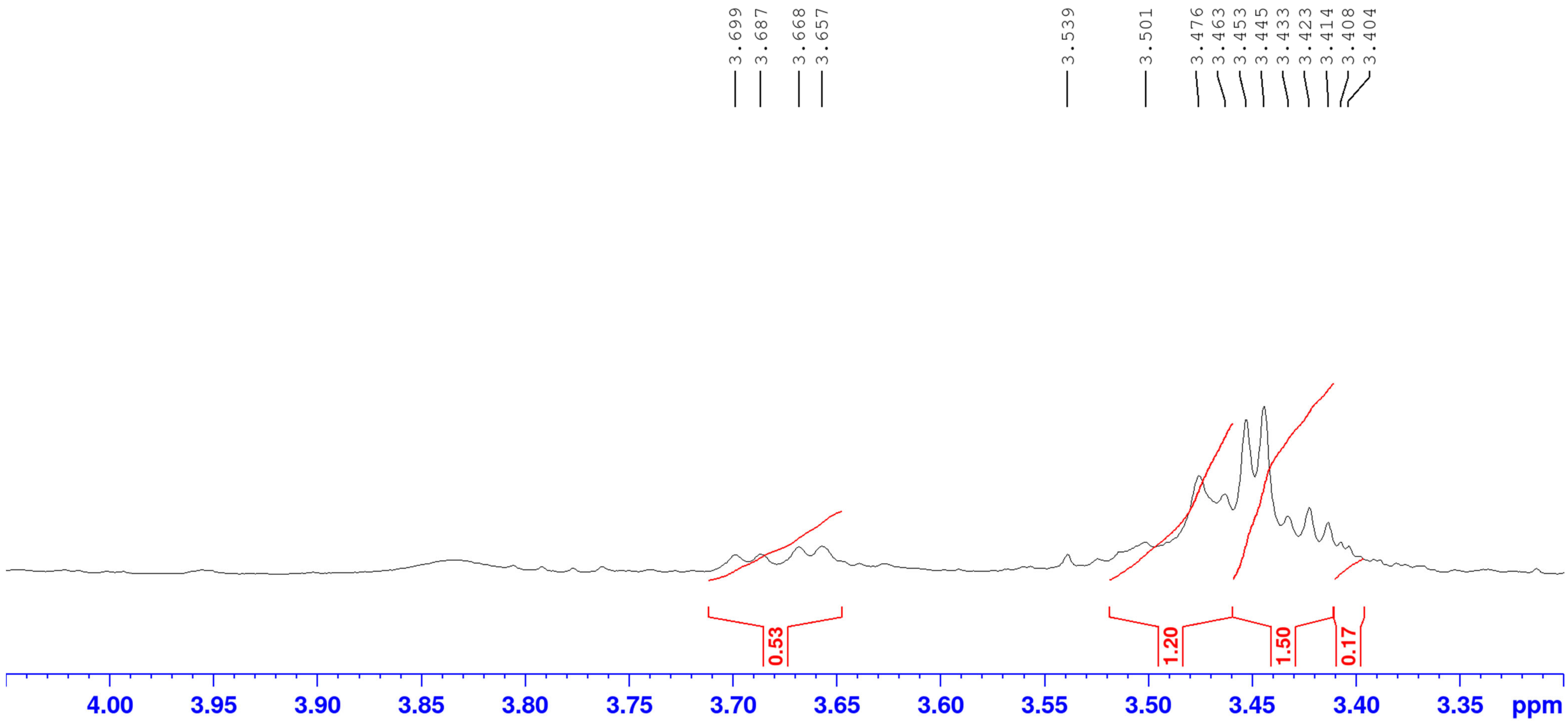

3.699
3.687
3.668
3.657
3.539
3.501
3.476
3.463
3.453
3.445
3.433
3.423
3.414
3.408
3.404
0.53
1.20
1.50
0.17
4.00
3.95
3.90
3.85
3.80
3.75
3.70
3.65
3.60
3.55
3.50
3.45
3.40
3.35
ppm

^{11}B-NMR

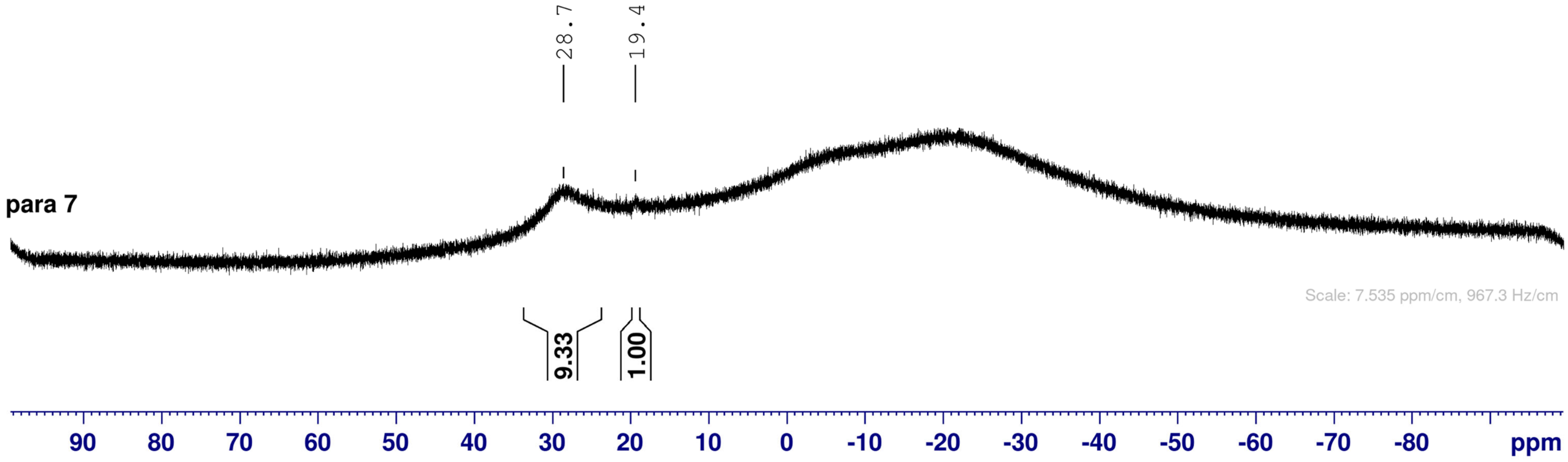

28.7
19.4
para 7
Scale: 7.535 ppm/cm, 967.3 Hz/cm
9.33
1.00
90 80 70 60 50 40 30 20 10 0 -10 -20 -30 -40 -50 -60 -70 -80 ppm

^{13}C-NMR

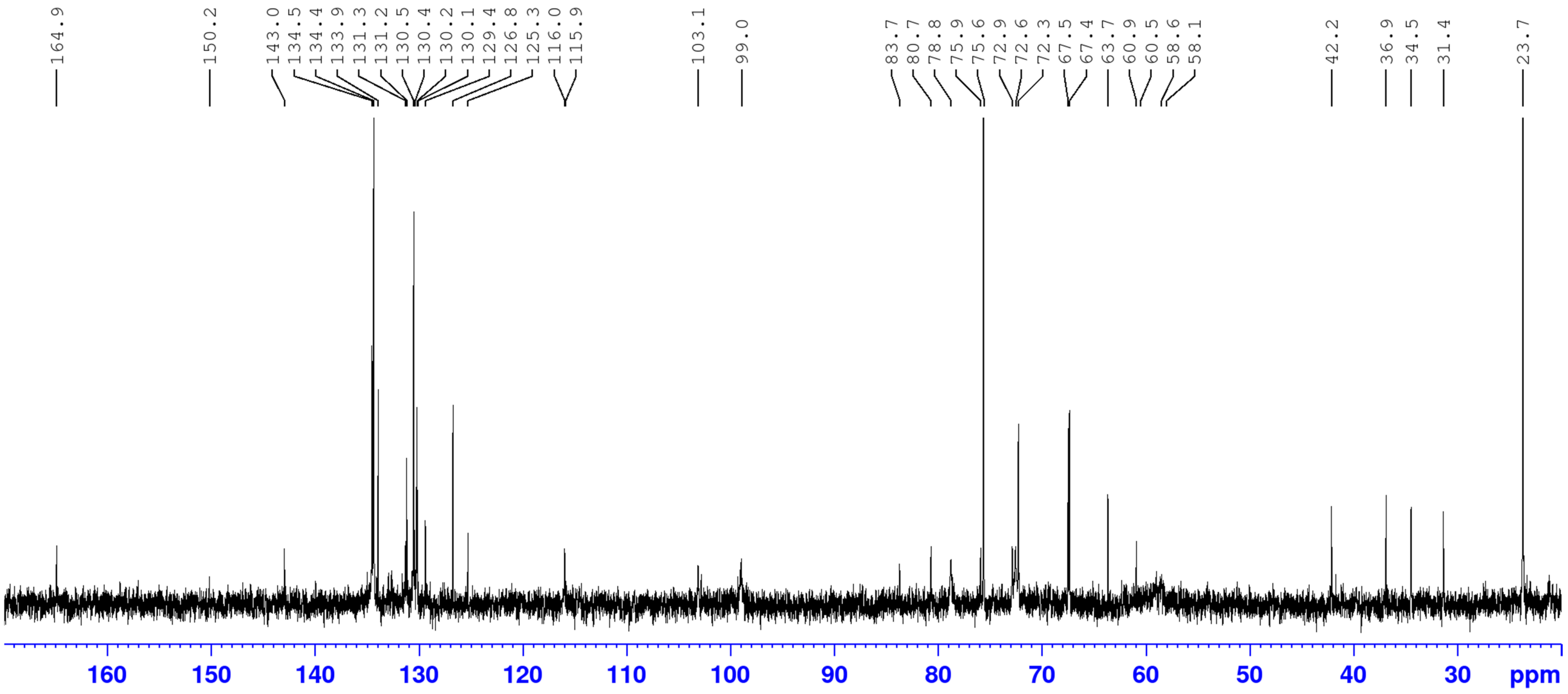

164.9
150.2
143.0
134.5
134.4
133.9
131.3
131.2
130.5
130.4
130.2
130.1
129.4
126.8
125.3
116.0
115.9
103.1
99.0
83.7
80.7
78.8
75.9
75.6
72.9
72.6
72.3
67.5
67.4
63.7
60.9
60.5
58.6
58.1
42.2
36.9
34.5
31.4
23.7
160
150
140
130
120
110
100
90
80
70
60
50
40
30
ppm

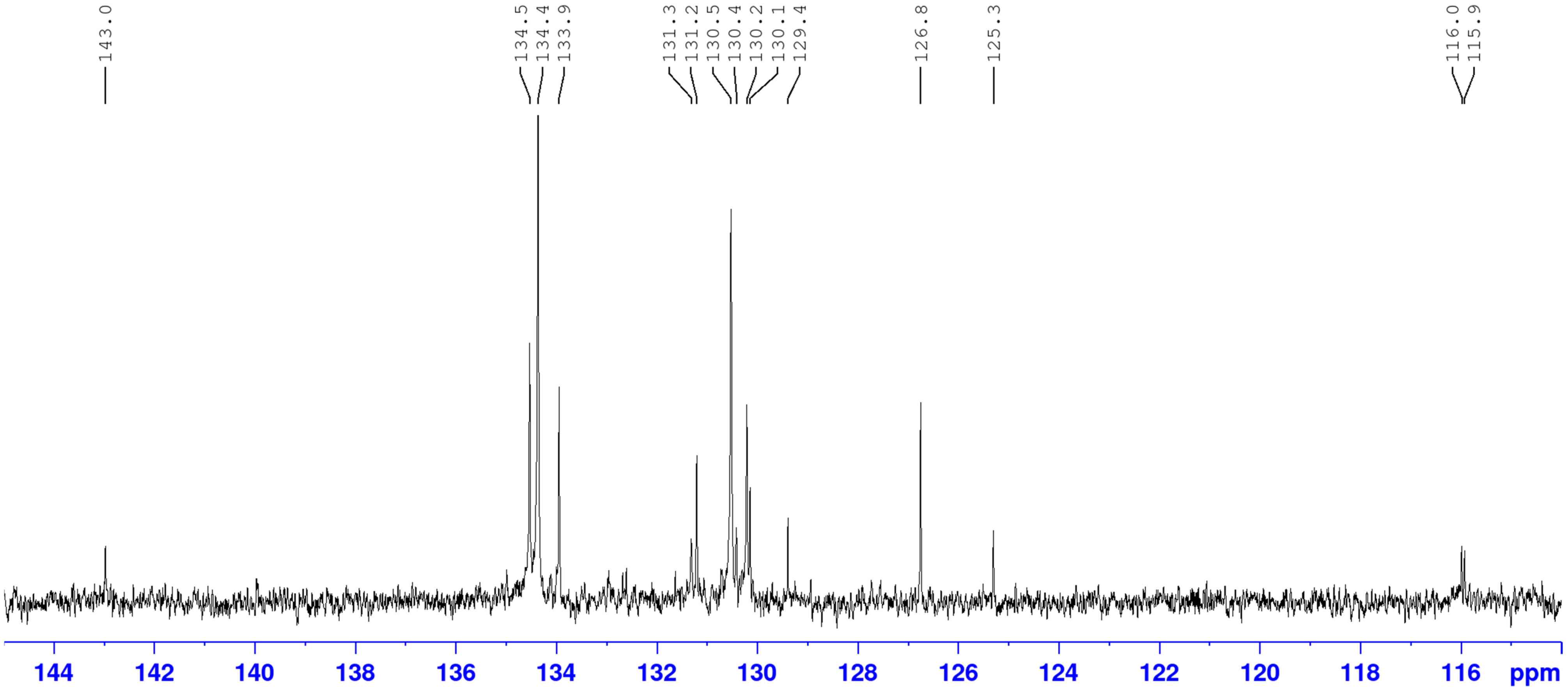
143.0
134.5
134.4
133.9
131.3
131.2
130.5
130.4
130.2
130.1
129.4
126.8
125.3
116.0
115.9
144
142
140
138
136
134
132
130
128
126
124
122
120
118
116
ppm

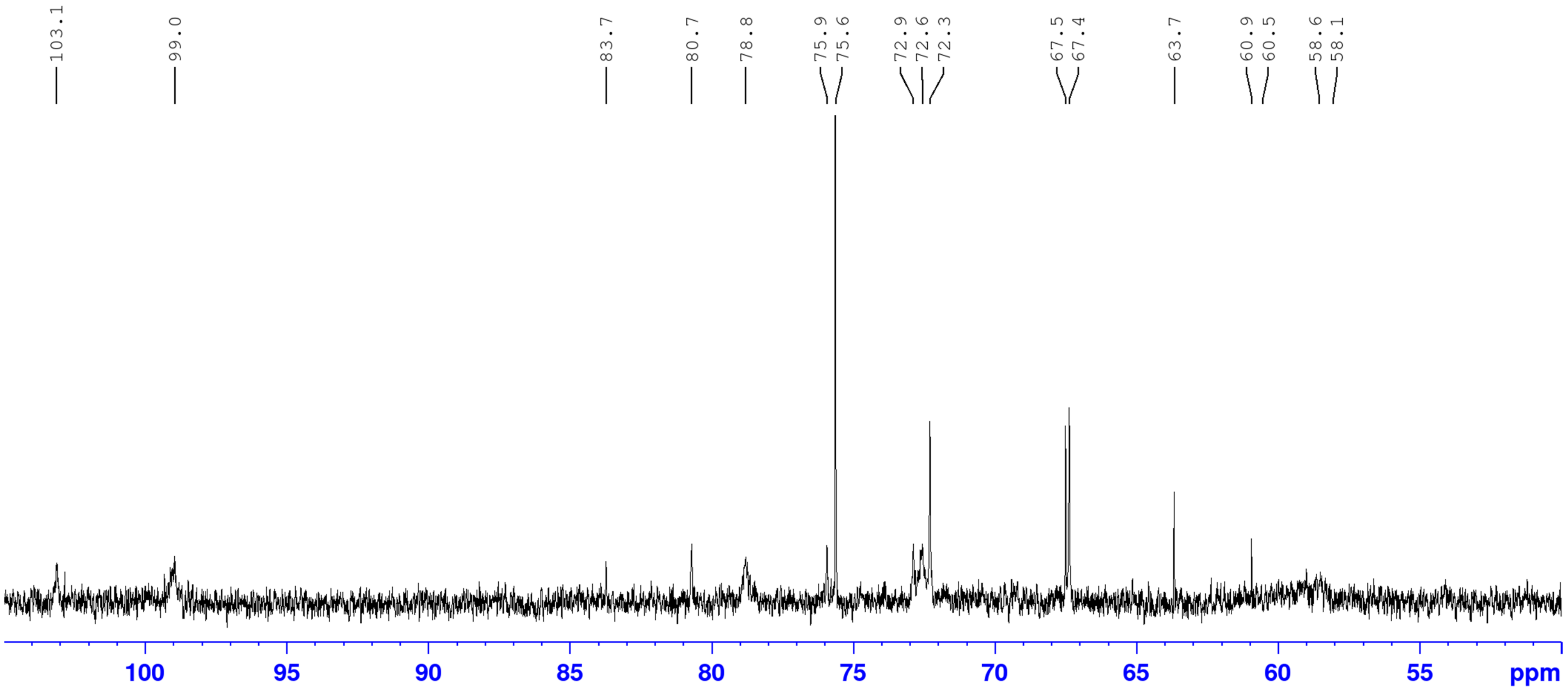
103.1
99.0
83.7
80.7
78.8
75.9
75.6
72.9
72.6
72.3
67.5
67.4
63.7
60.9
60.5
58.6
58.1
100
95
90
85
80
75
70
65
60
55
ppm

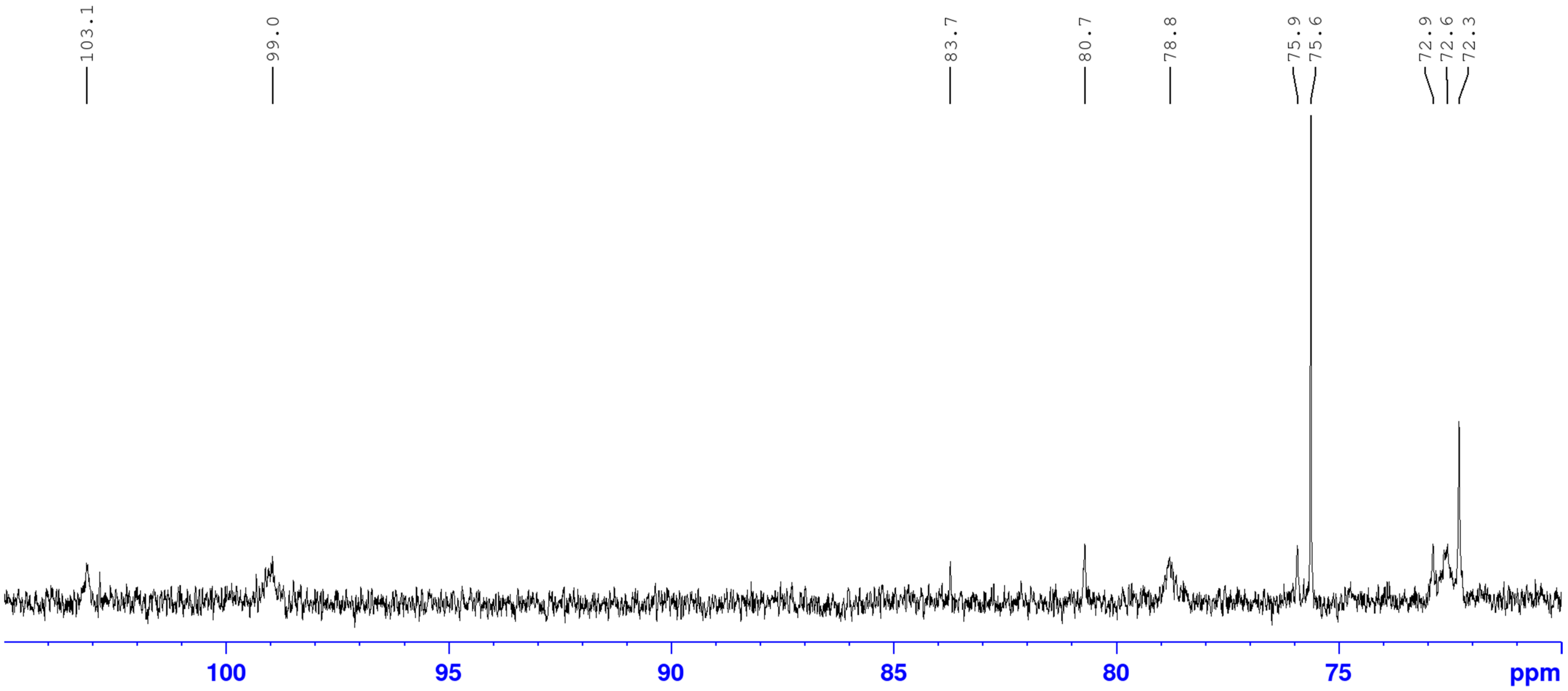
103.1
99.0
83.7
80.7
78.8
75.9
75.6
72.9
72.6
72.3
100
95
90
85
80
75
ppm

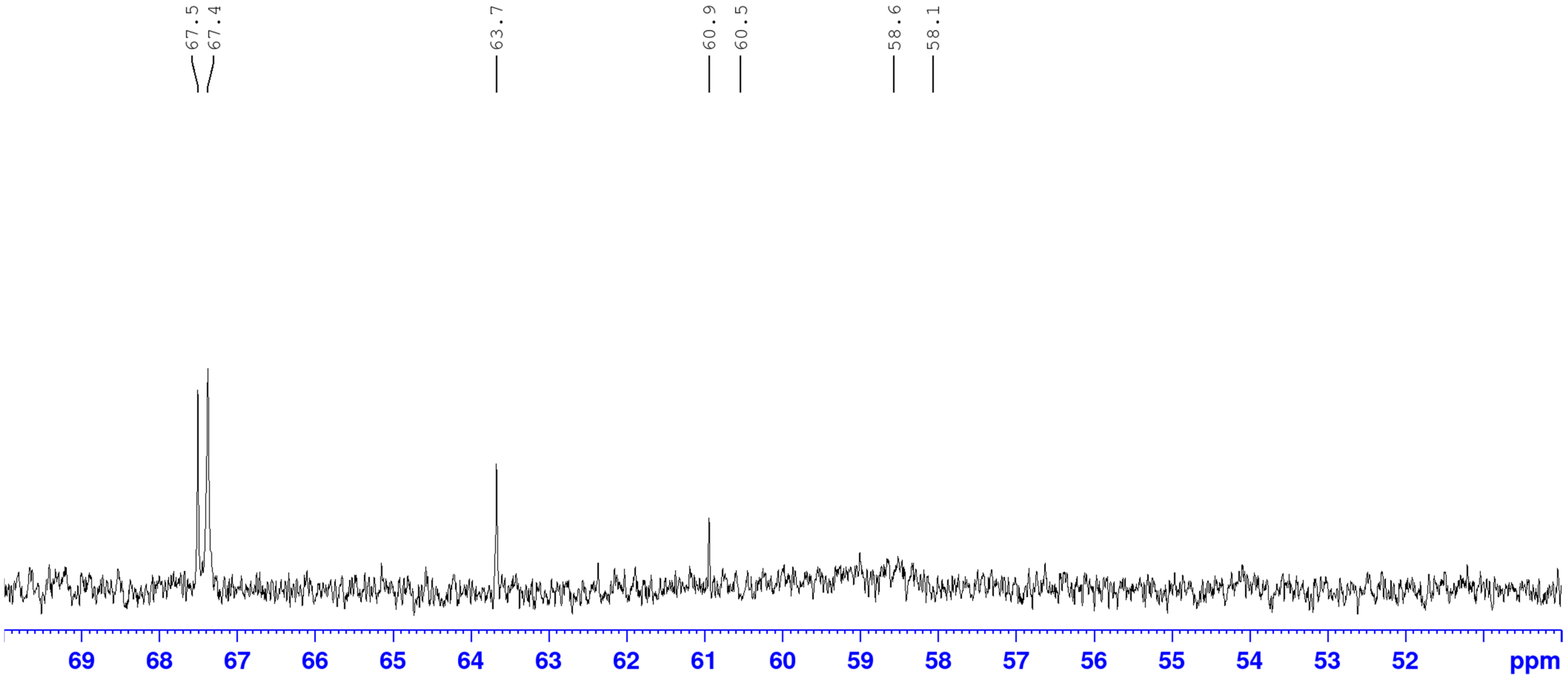

67.5
67.4
63.7
60.9
60.5
58.6
58.1
69 68 67 66 65 64 63 62 61 60 59 58 57 56 55 54 53 52
ppm

COSY

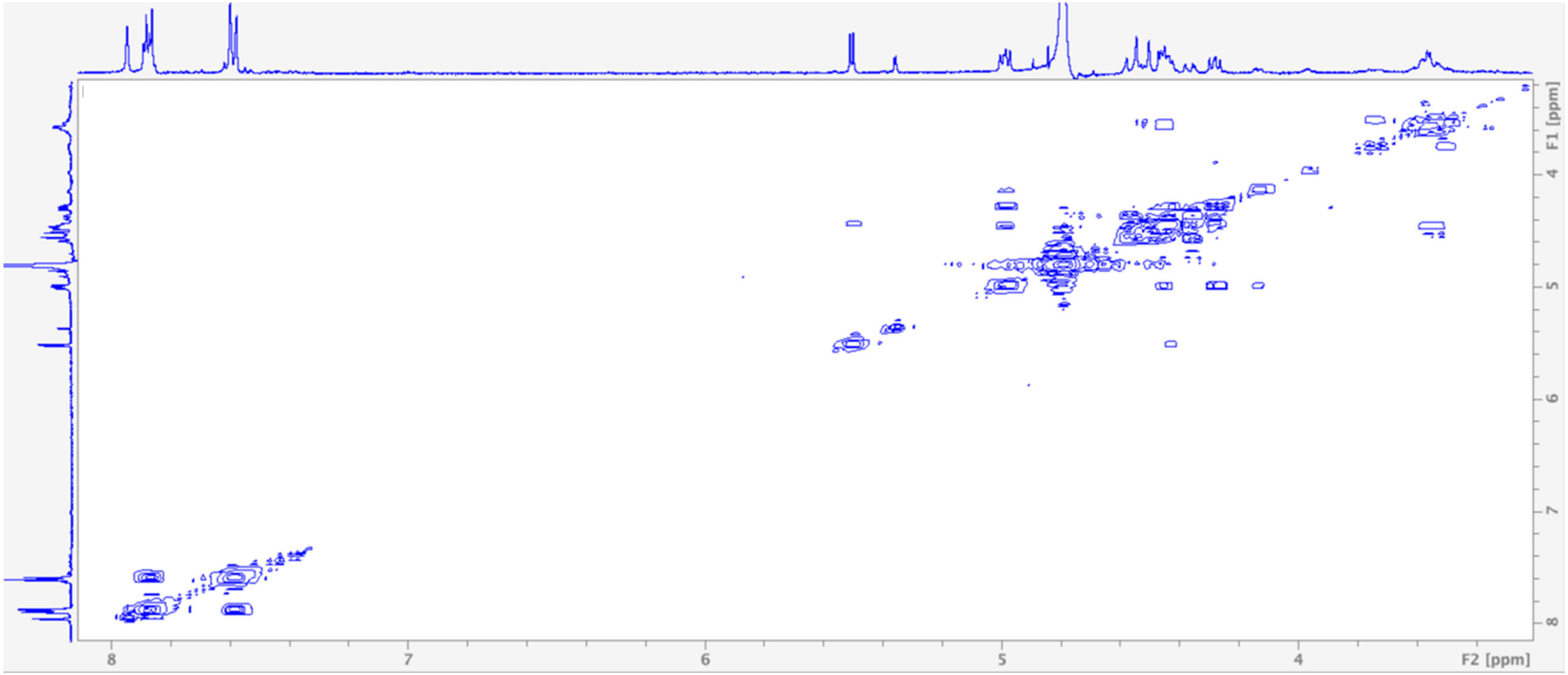

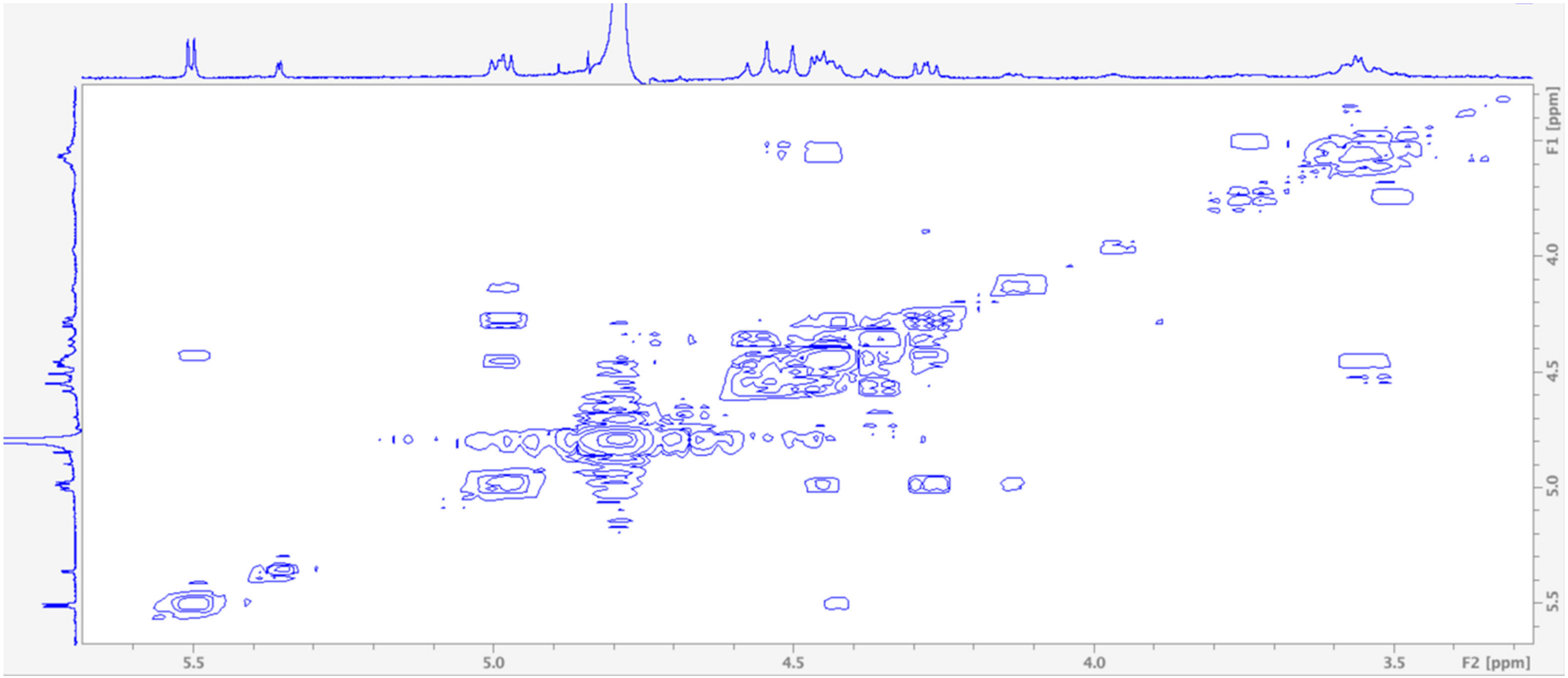

F1 [ppm]
4.0
4.5
5.0
5.5
5.5
5.0
4.5
4.0
3.5
F2 [ppm]

HSQC

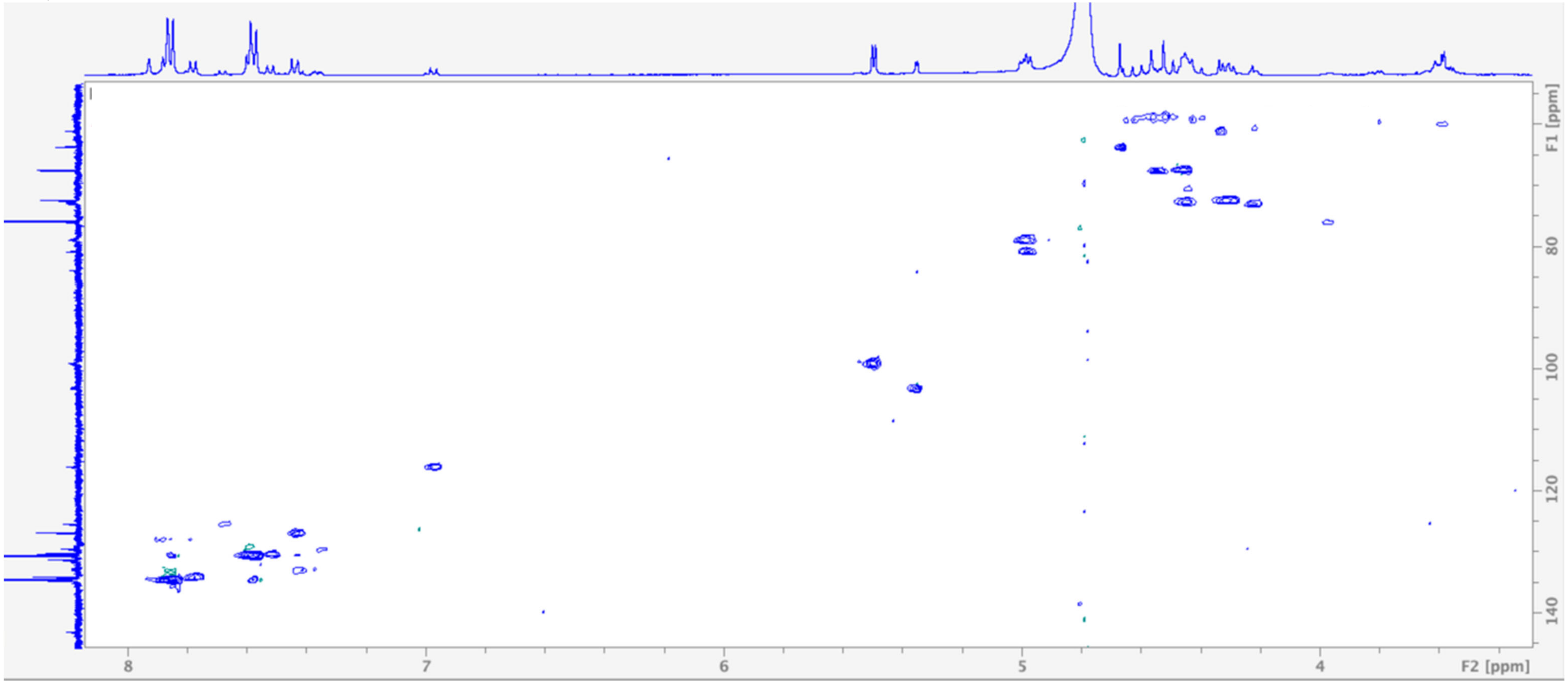

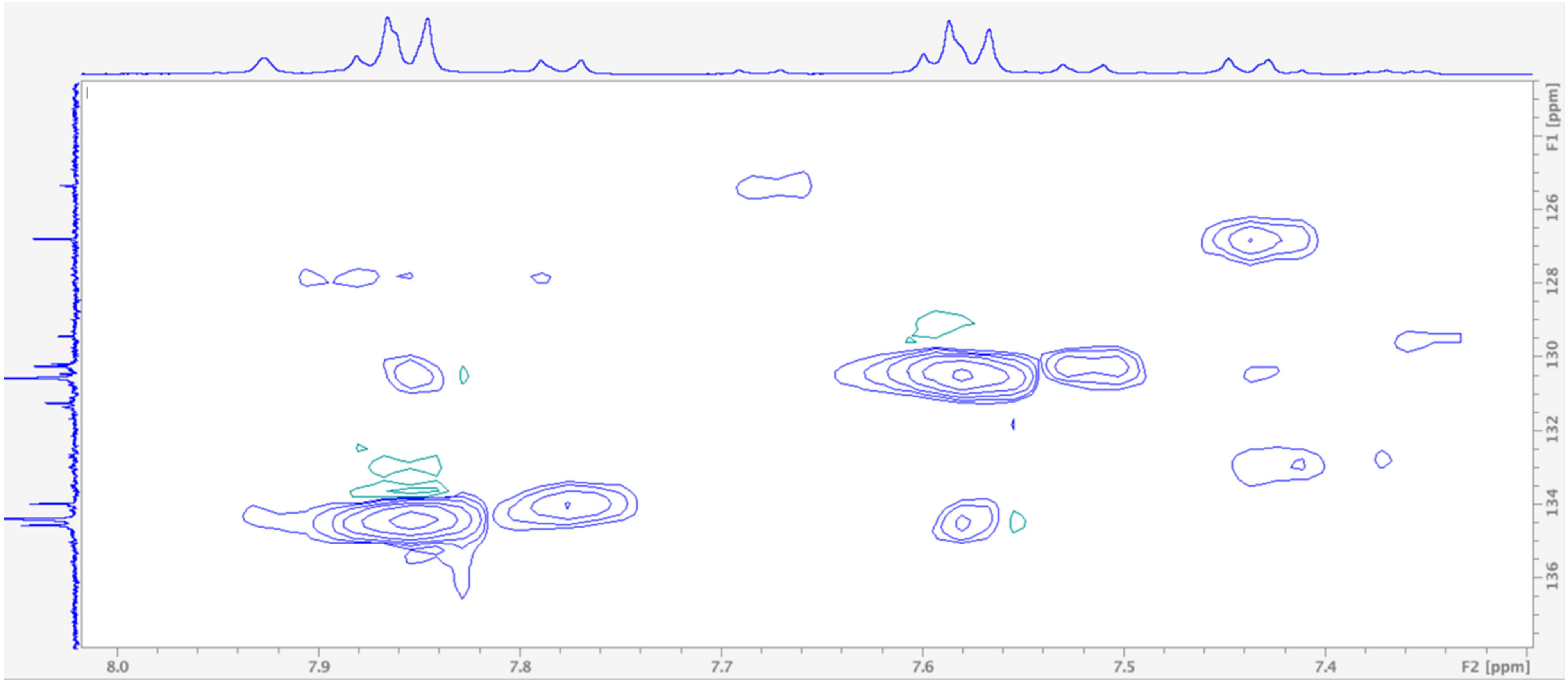
F1 [ppm]
126
128
130
132
134
136
8.0
7.9
7.8
7.7
7.6
7.5
7.4
F2 [ppm]

HMBC

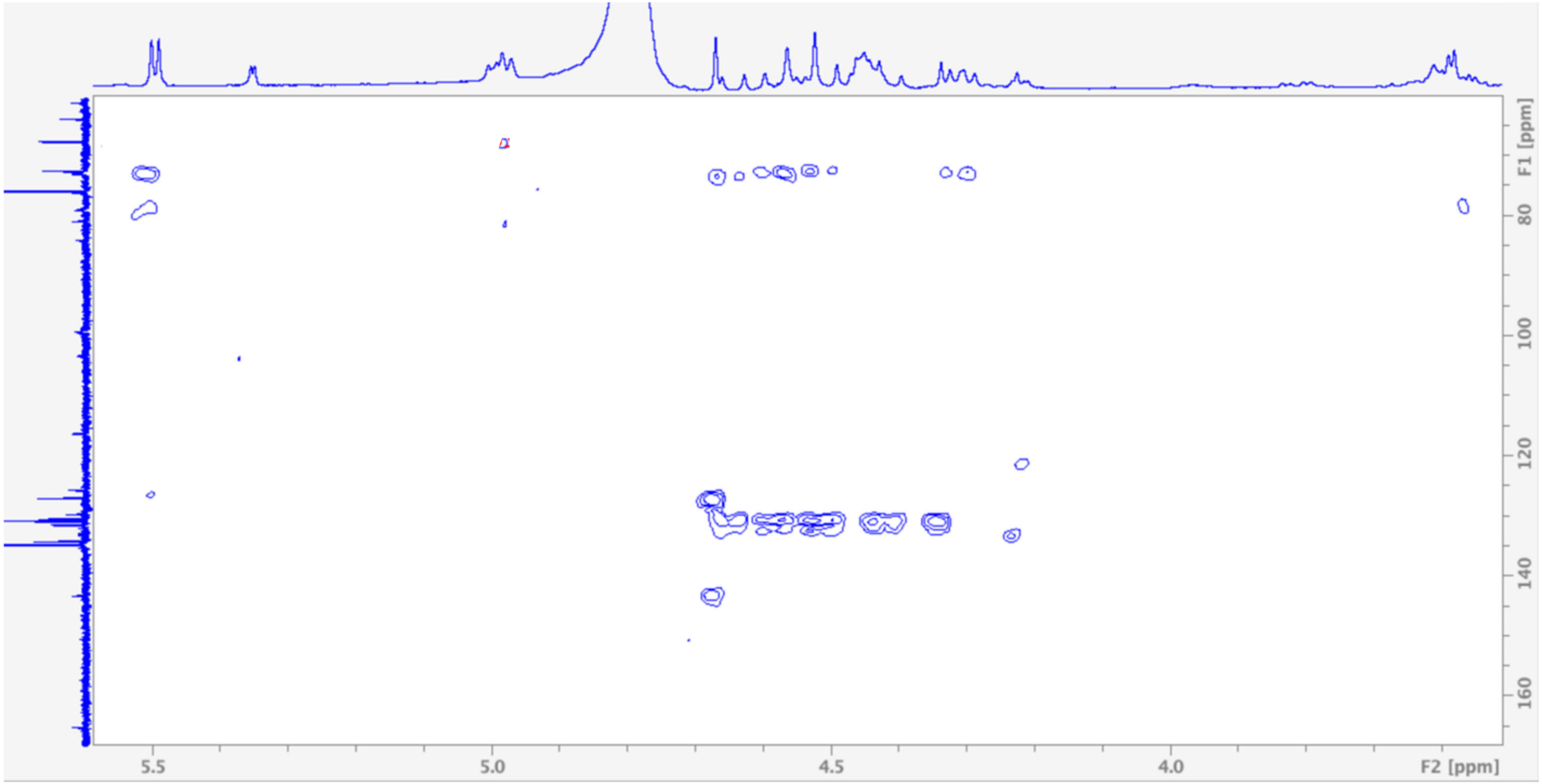

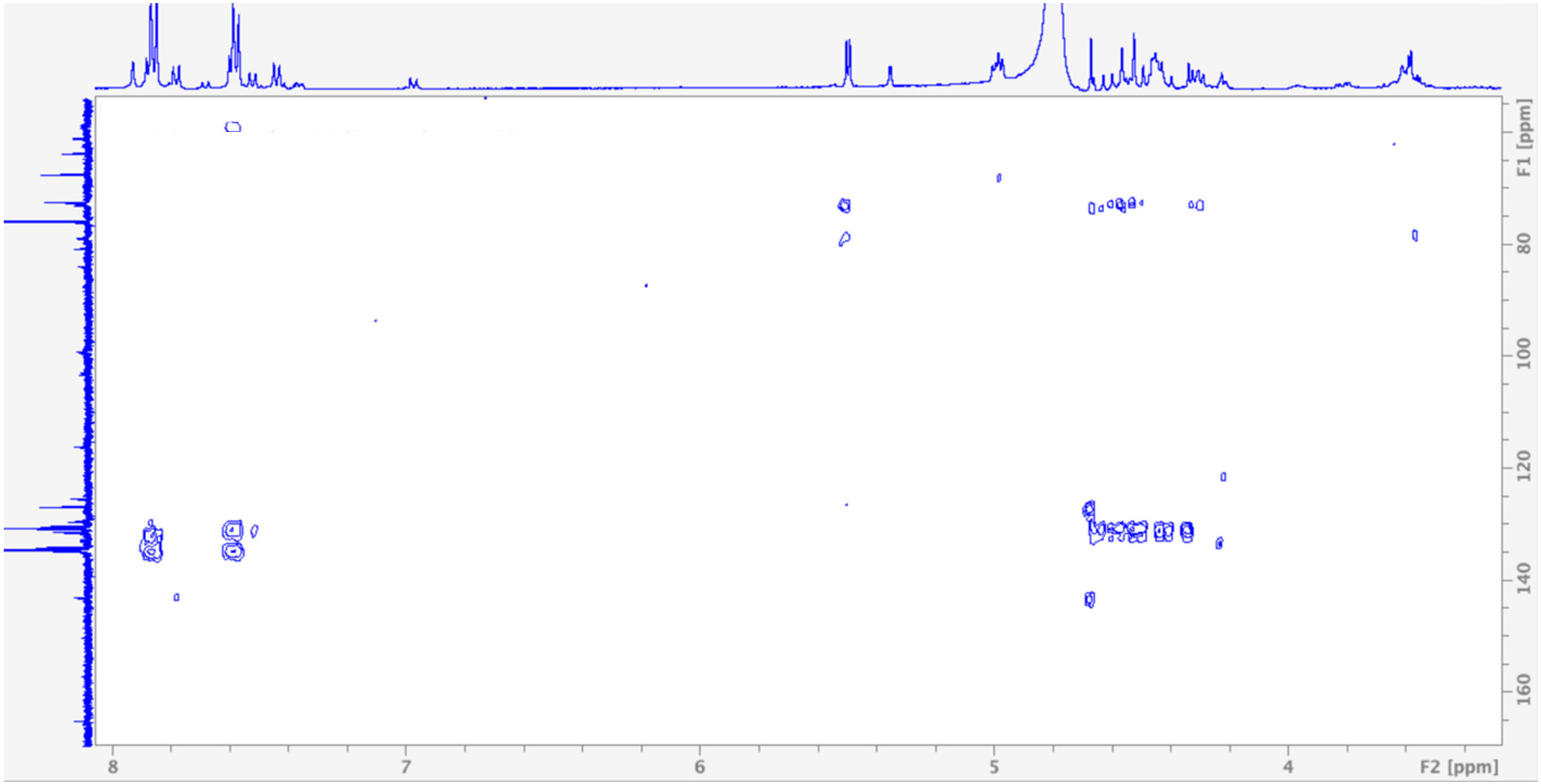

F1 [ppm]
80
100
120
140
160
8
7
6
5
4
F2 [ppm]

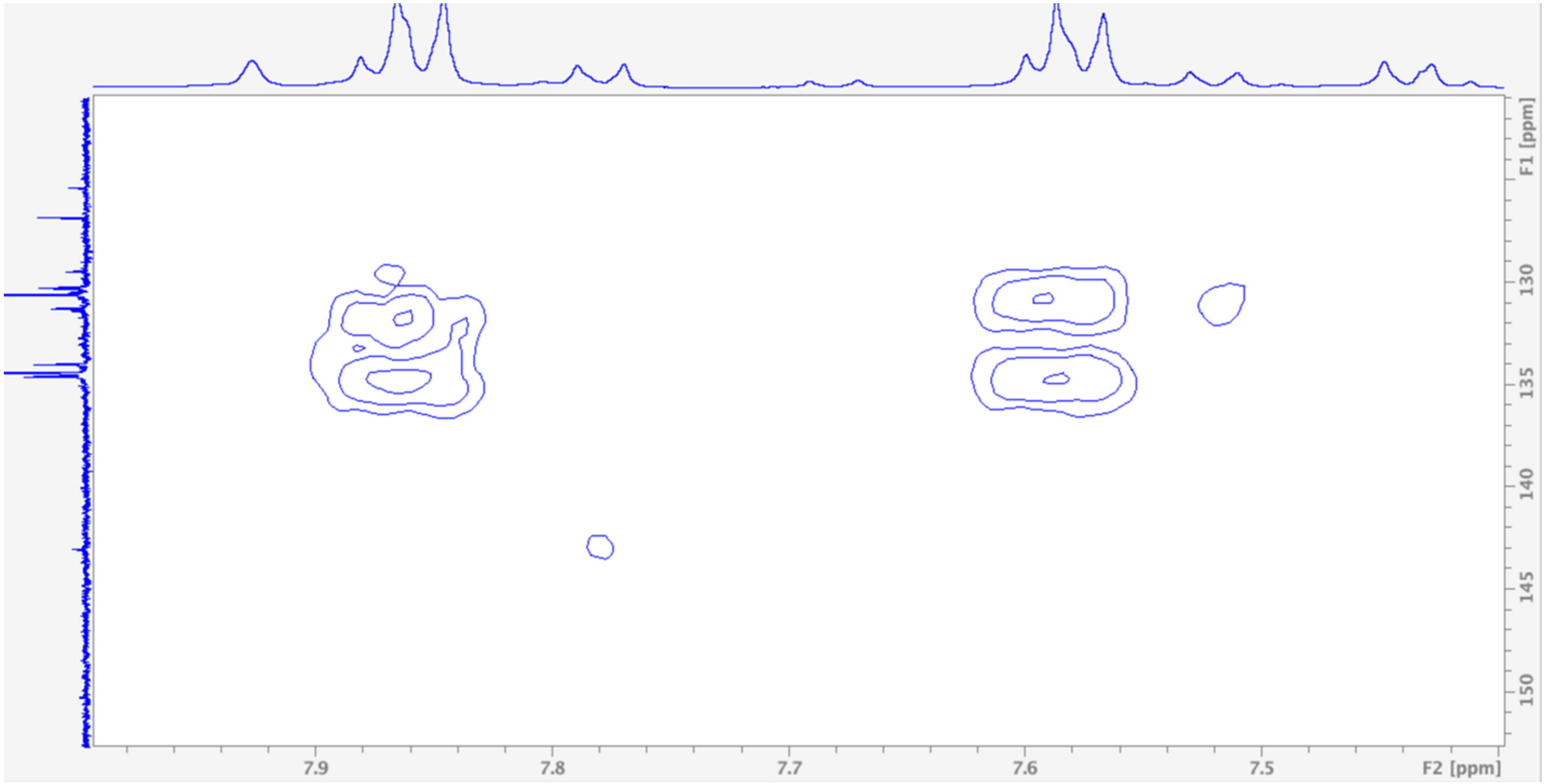

7.9
7.8
7.7
7.6
7.5
F2 [ppm]
130
135
140
145
150
F1 [ppm]

Figure S10. ^{1}H-NMR spectrum (400 MHz, D_2O) of *N*-(4-methylphenyl boronic acid)-3,6-dideoxy-3,6-imino-D-gulofuranose **para 7** with colour-coded signals, highlighting the furanose anomeric forms they belong to, with interpretation of the isolated signals and tentative interpretation of the overlapping ones. Namely, the orange designates the *α-fur* form and indigo designates the *β-fur*. Section 7.92 ppm to 7.55 ppm is visible with highlighted principal COSY correlations to hydrogen atoms within the same spin systems.

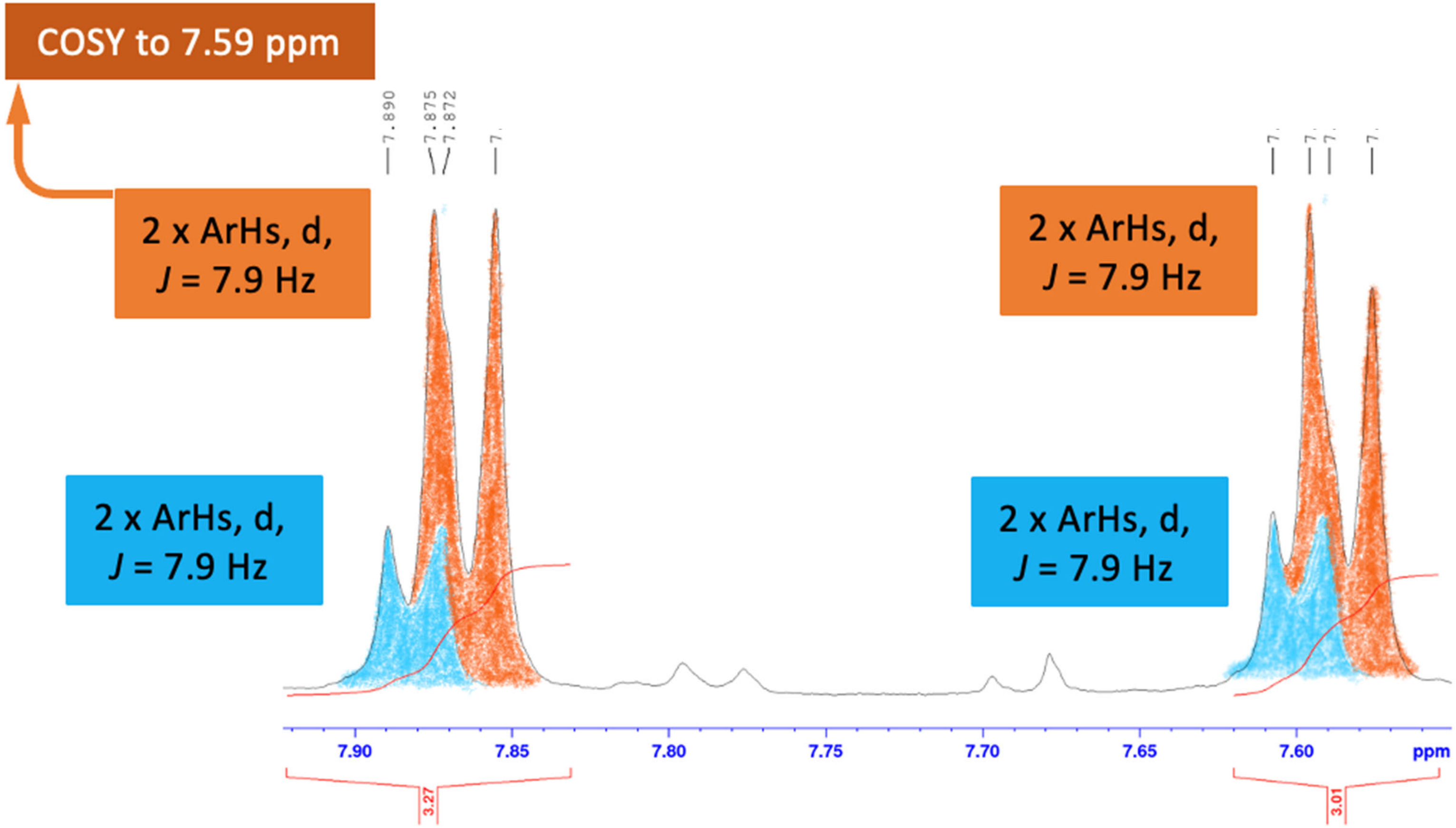

Figure S11. ^{1}H-NMR spectrum (400 MHz, D_2O) of *N*-(4-methylphenyl boronic acid)-3,6-dideoxy-3,6-imino-D-gulofuranose **para 7** with colour-coded signals, highlighting the furanose anomeric forms they belong to, with interpretation of the isolated signals and tentative interpretation of the overlapping ones. Namely, the orange designates the *α-fur* form and indigo designates the *β-fur*. A) section 5.60 ppm to 4.90 ppm; B) section 4.75 ppm to 3.50 ppm. Highlighted are also the principal COSY correlations to hydrogen atoms within the same spin systems.

A

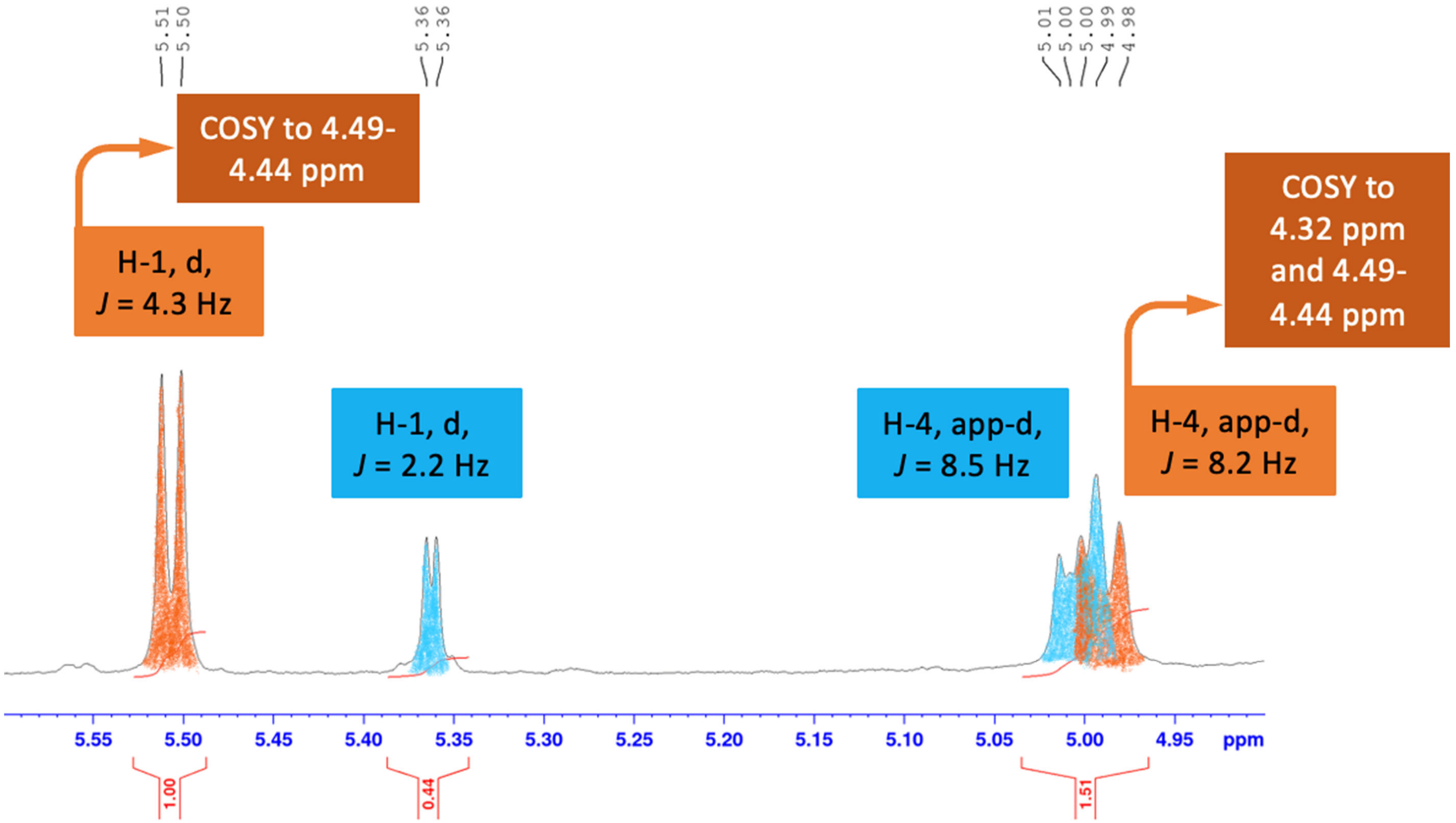

B

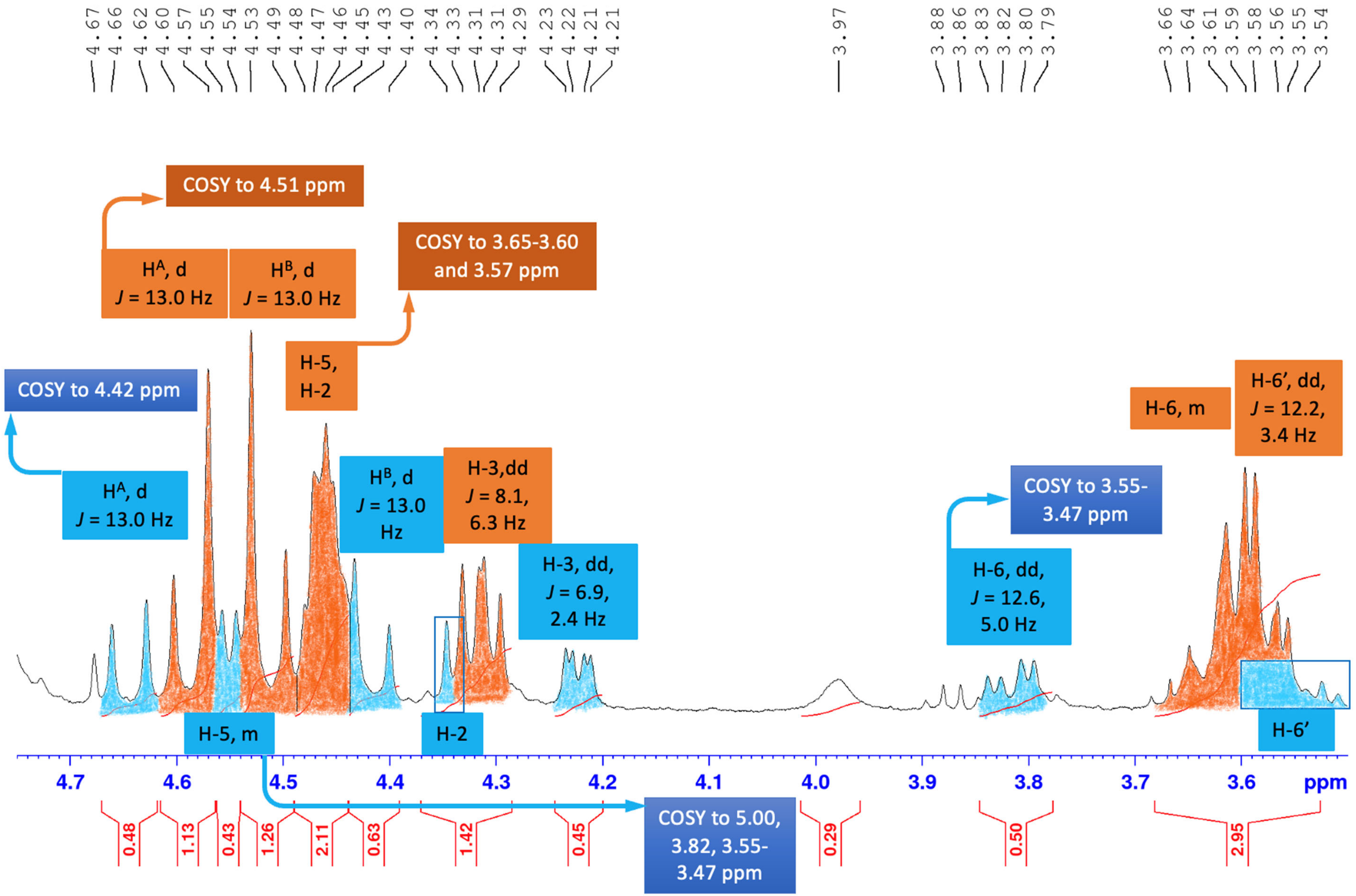
4.67 4.66 4.62 4.60 4.57 4.55 4.54 4.53 4.49 4.48 4.47 4.46 4.45 4.43 4.40 4.34 4.33 4.31 4.31 4.29 4.23 4.22 4.21 4.21
3.97
3.88 3.86 3.83 3.82 3.80 3.79
3.66 3.64 3.61 3.59 3.58 3.56 3.55 3.54
COSY to 4.51 ppm
COSY to 3.65-3.60 and 3.57 ppm
H^A, d J = 13.0 Hz
H^B, d J = 13.0 Hz
H-5, H-2
COSY to 4.42 ppm
H-6, m
H-6′, dd, J = 12.2, 3.4 Hz
H^B, d J = 13.0 Hz
H-3,dd J = 8.1, 6.3 Hz
COSY to 3.55-3.47 ppm
H^A, d J = 13.0 Hz
H-3, dd, J = 6.9, 2.4 Hz
H-6, dd, J = 12.6, 5.0 Hz
H-5, m
H-2
H-6′
4.7 4.6 4.5 4.4 4.3 4.2 4.1 4.0 3.9 3.8 3.7 3.6 ppm
0.48 1.13 0.43 1.26 2.11 0.63 1.42 0.45 0.29 0.50 2.95
COSY to 5.00, 3.82, 3.55-3.47 ppm

Figure S12. ^{13}C-NMR spectrum (100 MHz, D_2O) sections of *N*-(4-methylphenyl boronic acid)-3,6-dideoxy-3,6-imino-D-gulofuranose **para 7** with colour-coded signals, highlighting the furanose anomeric forms they belong to, with interpretation of the isolated signals and tentative interpretation of the overlapping ones. Namely, the orange designates the *α-fur* form and indigo designates the *β-fur*. A) section 103.6 ppm to 97.5 ppm; B) section 81.0 ppm to 72.0 ppm; C) section 68.0 ppm to 56.5 ppm. Highlighted are also the principal HSQC correlations to hydrogen atoms in the same spin systems.

A

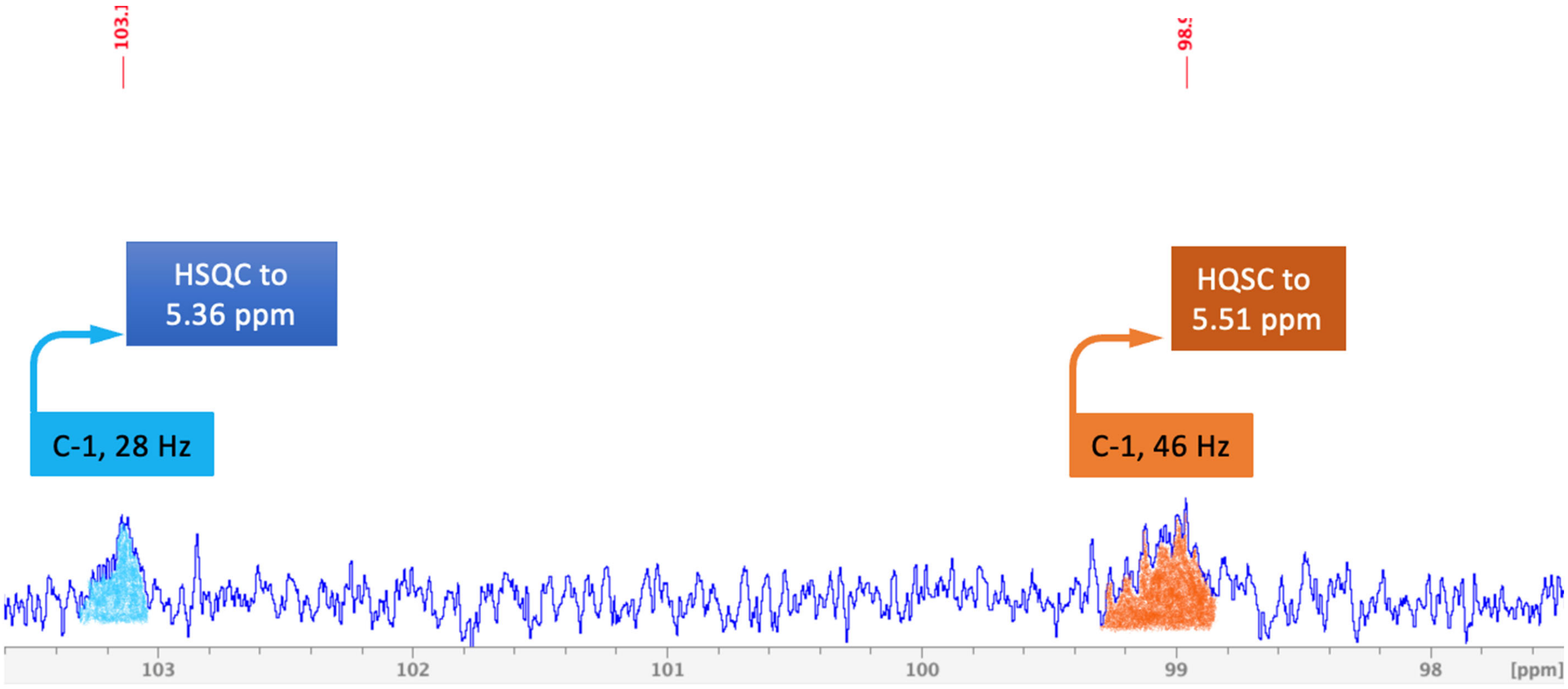

B

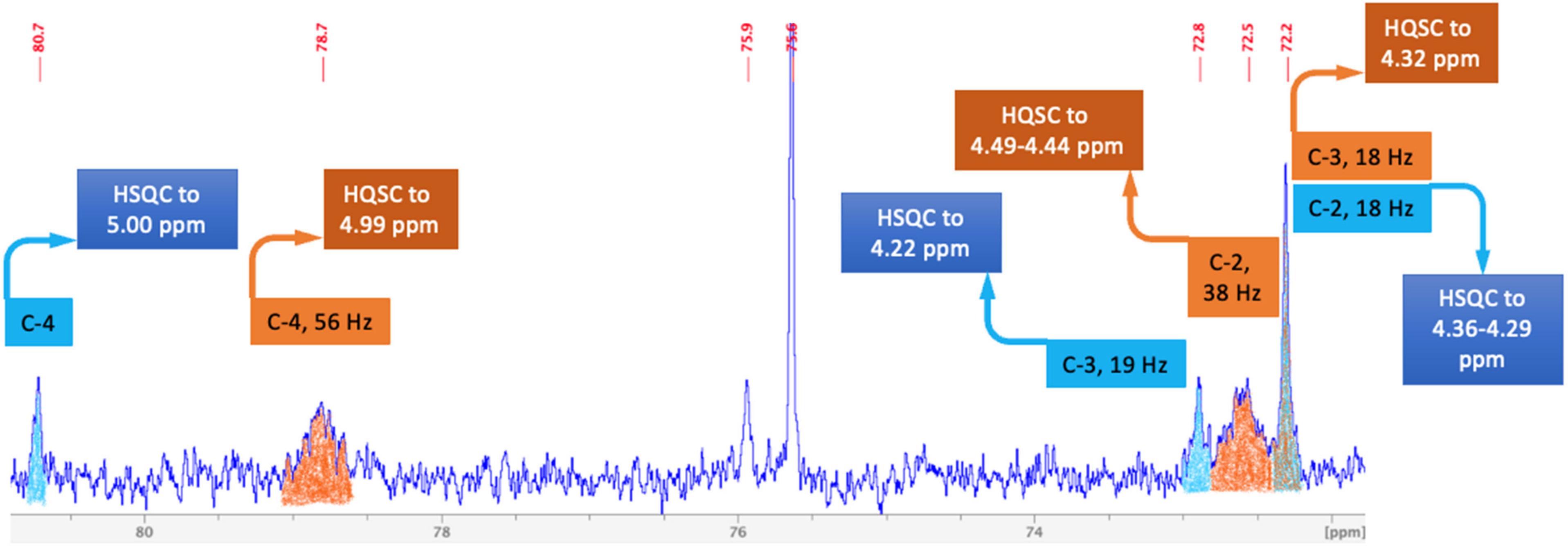
80.7
78.7
75.9
75.6
72.8
72.5
72.2
HSQC to
5.00 ppm
C-4
HQSC to
4.99 ppm
C-4, 56 Hz
HSQC to
4.22 ppm
C-3, 19 Hz
HQSC to
4.49-4.44 ppm
C-2,
38 Hz
HQSC to
4.32 ppm
C-3, 18 Hz
C-2, 18 Hz
HSQC to
4.36-4.29
ppm
80
78
76
74
[ppm]

C

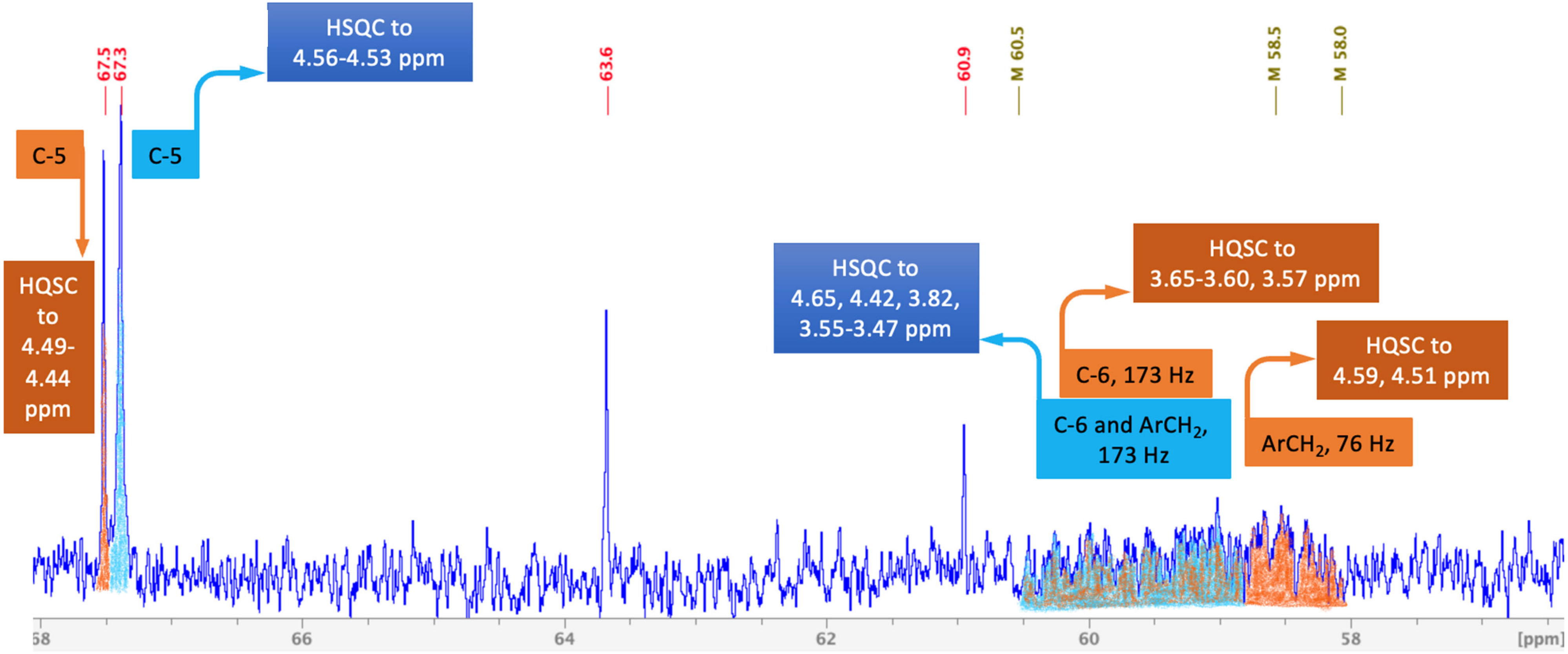

HSQC to
4.56-4.53 ppm
67.5
67.3
63.6
60.9
M 60.5
M 58.5
M 58.0
C-5
C-5
HQSC to 4.49-4.44 ppm
HSQC to
4.65, 4.42, 3.82,
3.55-3.47 ppm
HQSC to
3.65-3.60, 3.57 ppm
C-6, 173 Hz
HQSC to
4.59, 4.51 ppm
C-6 and ArCH2, 173 Hz
ArCH2, 76 Hz
68
66
64
62
60
58
[ppm]

Figure S13. ^{1}H- (400 MHz), ^{13}C-NMR (100 MHz), ^{11}B-NMR (128 MHz), COSY and HSQC spectra of *N*-(4-methylphenyl boronic acid)-1,4-dideoxy-1,4-imino-L-gulitol **para 8** in D_2O.

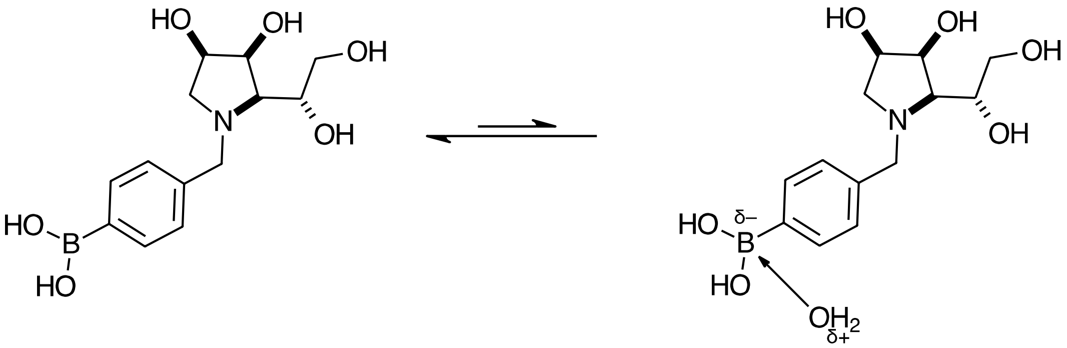

^{1}H-NMR

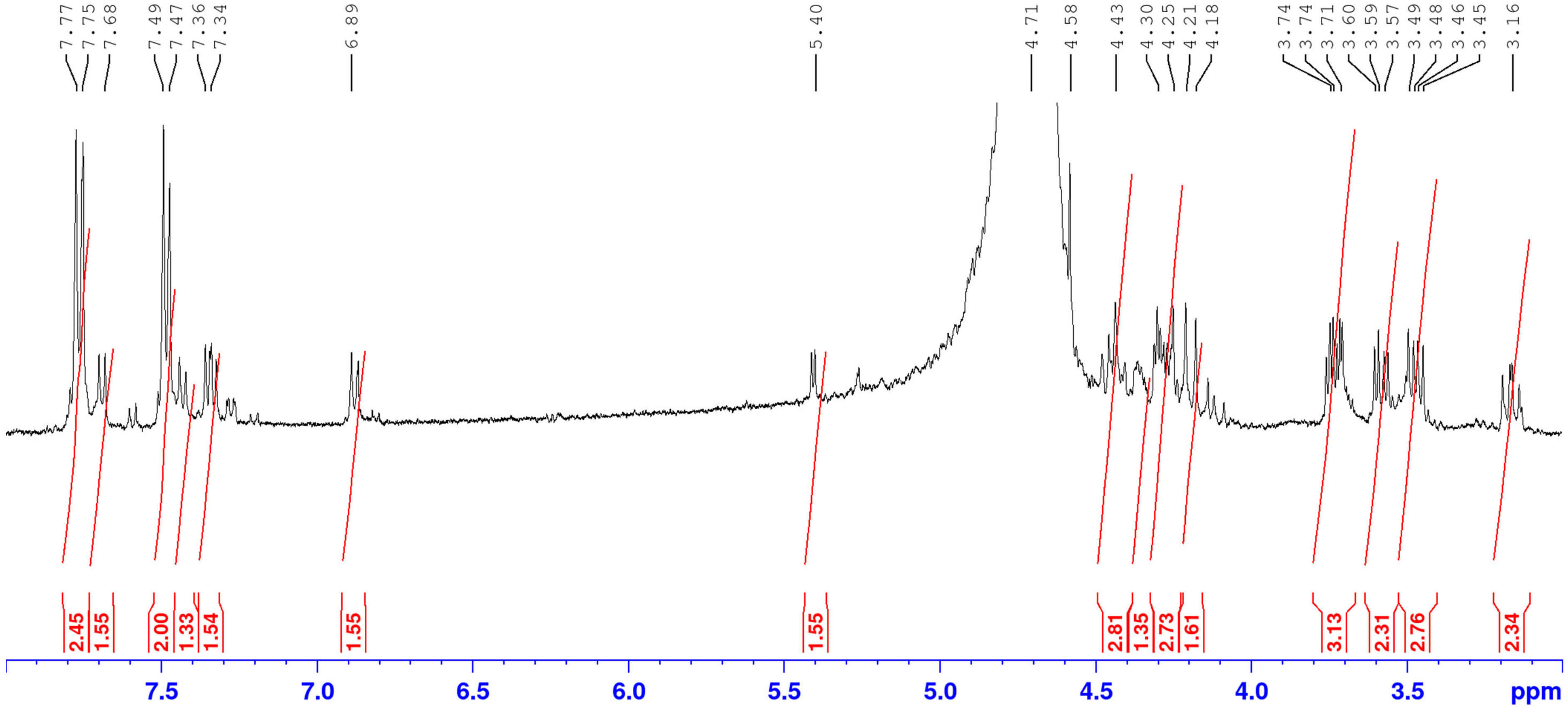

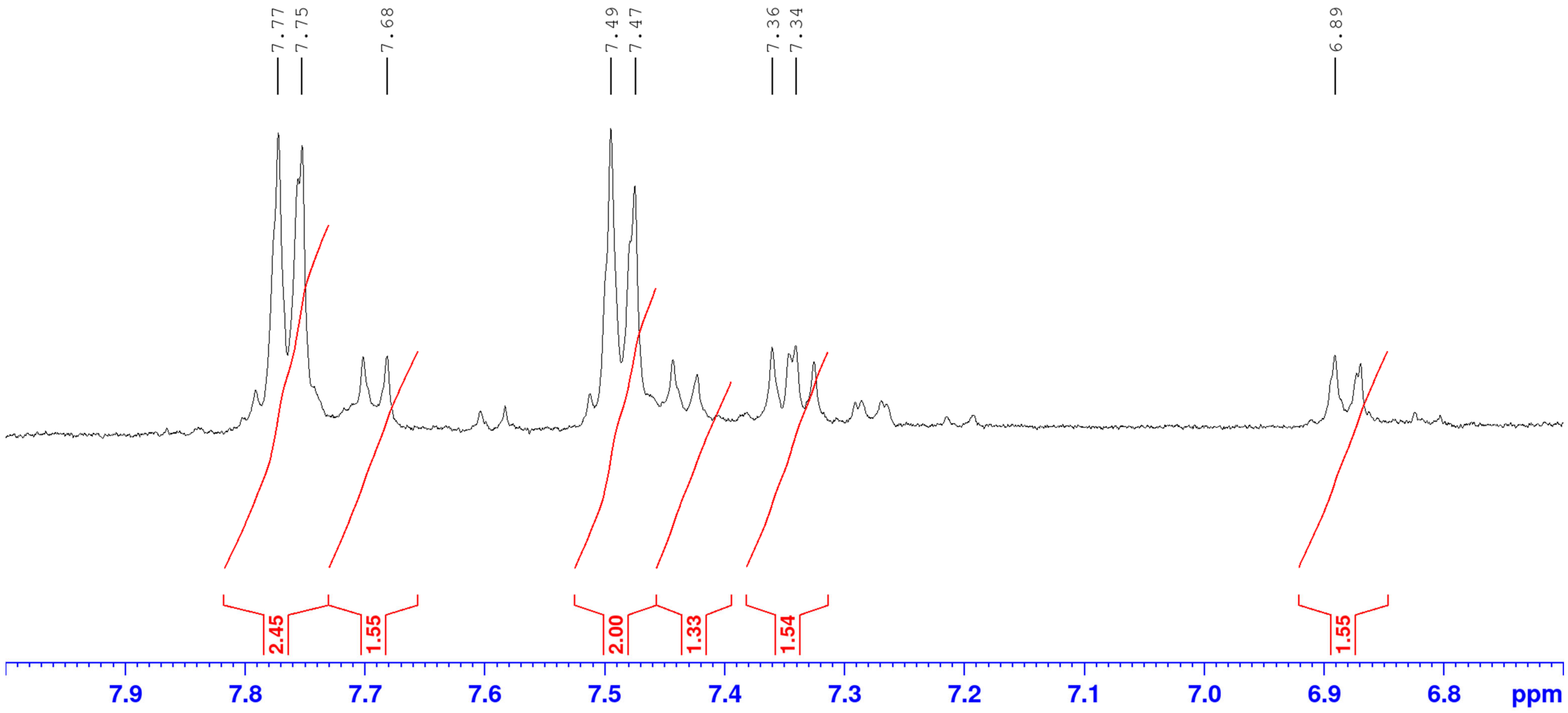
7.77
7.75
7.68
7.49
7.47
7.36
7.34
6.89
2.45
1.55
2.00
1.33
1.54
1.55
7.9
7.8
7.7
7.6
7.5
7.4
7.3
7.2
7.1
7.0
6.9
6.8
ppm

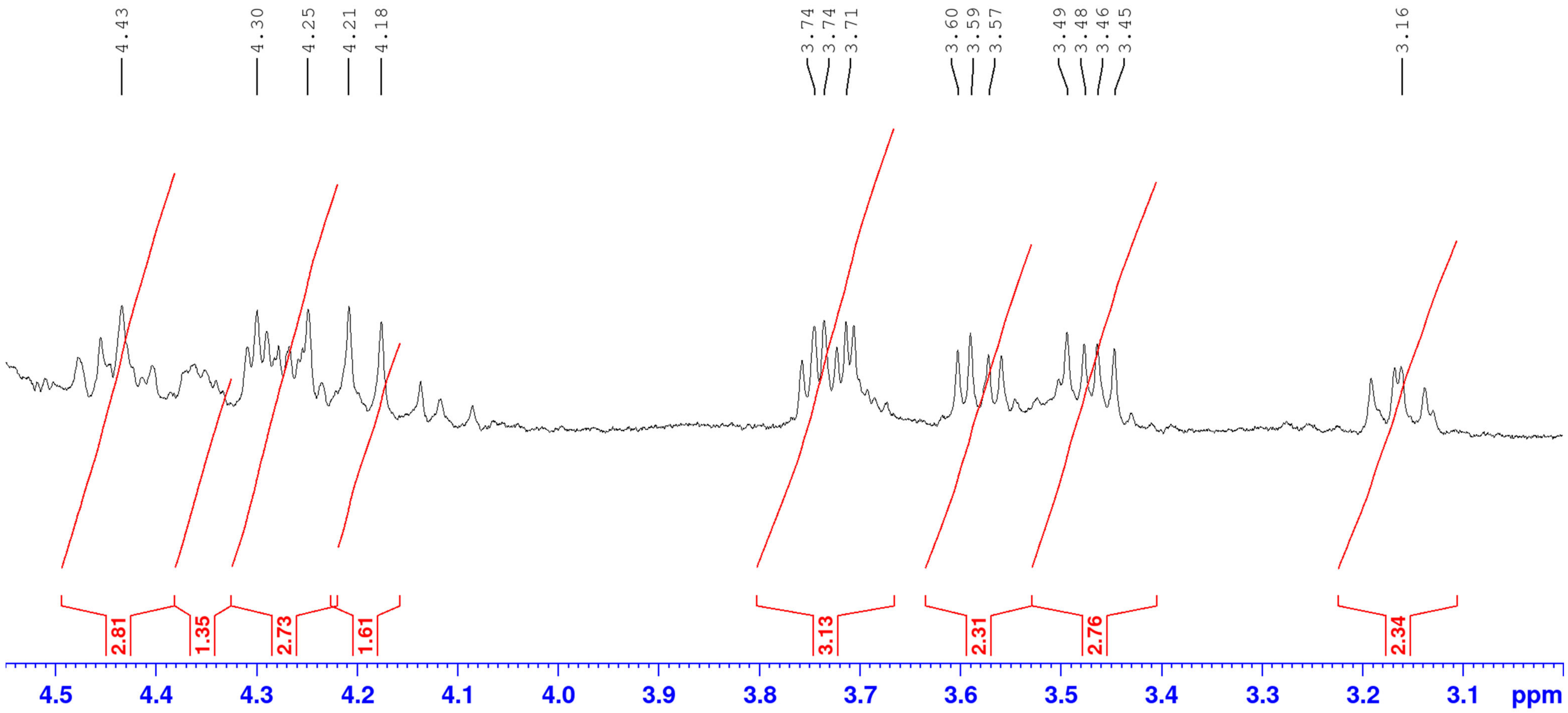
4.43
4.30
4.25
4.21
4.18
3.74
3.74
3.71
3.60
3.59
3.57
3.49
3.48
3.46
3.45
3.16
2.81
1.35
2.73
1.61
3.13
2.31
2.76
2.34
4.5
4.4
4.3
4.2
4.1
4.0
3.9
3.8
3.7
3.6
3.5
3.4
3.3
3.2
3.1
ppm

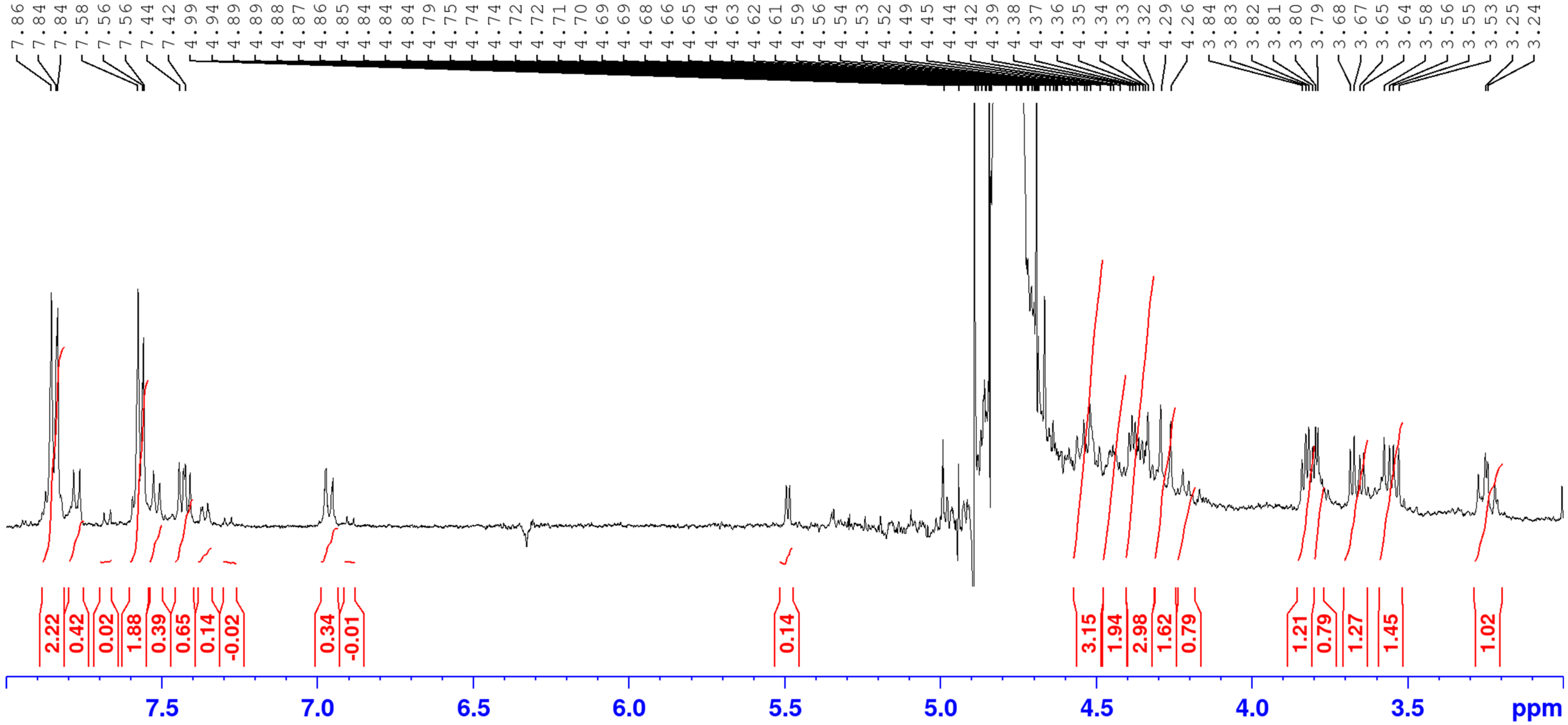
7.5
7.0
6.5
6.0
5.5
5.0
4.5
4.0
3.5
ppm

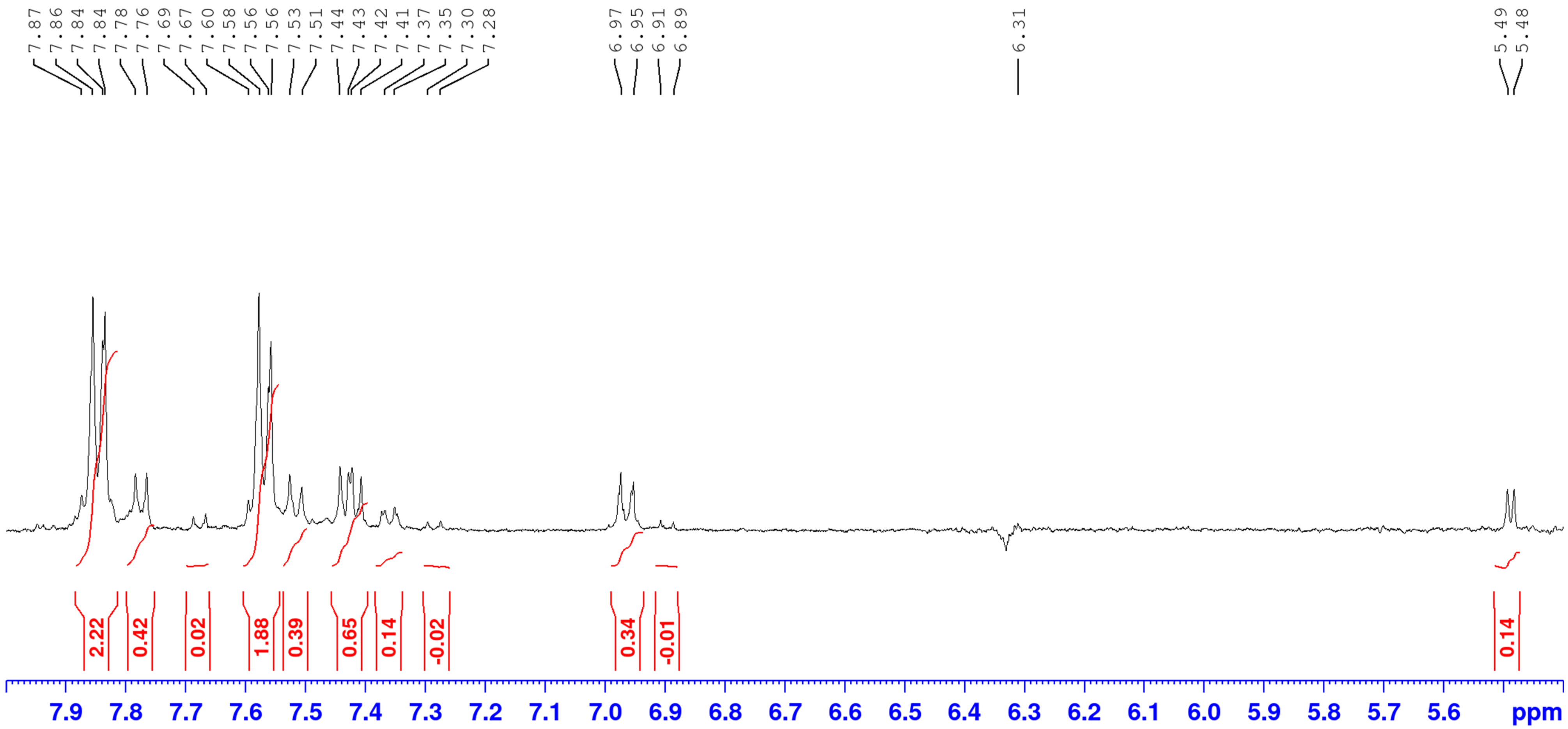
7.87
7.86
7.84
7.84
7.78
7.76
7.69
7.67
7.60
7.58
7.56
7.56
7.53
7.51
7.44
7.43
7.42
7.41
7.37
7.35
7.30
7.28
6.97
6.95
6.91
6.89
6.31
5.49
5.48
2.22
0.42
0.02
1.88
0.39
0.65
0.14
-0.02
0.34
-0.01
0.14
7.9 7.8 7.7 7.6 7.5 7.4 7.3 7.2 7.1 7.0 6.9 6.8 6.7 6.6 6.5 6.4 6.3 6.2 6.1 6.0 5.9 5.8 5.7 5.6 ppm

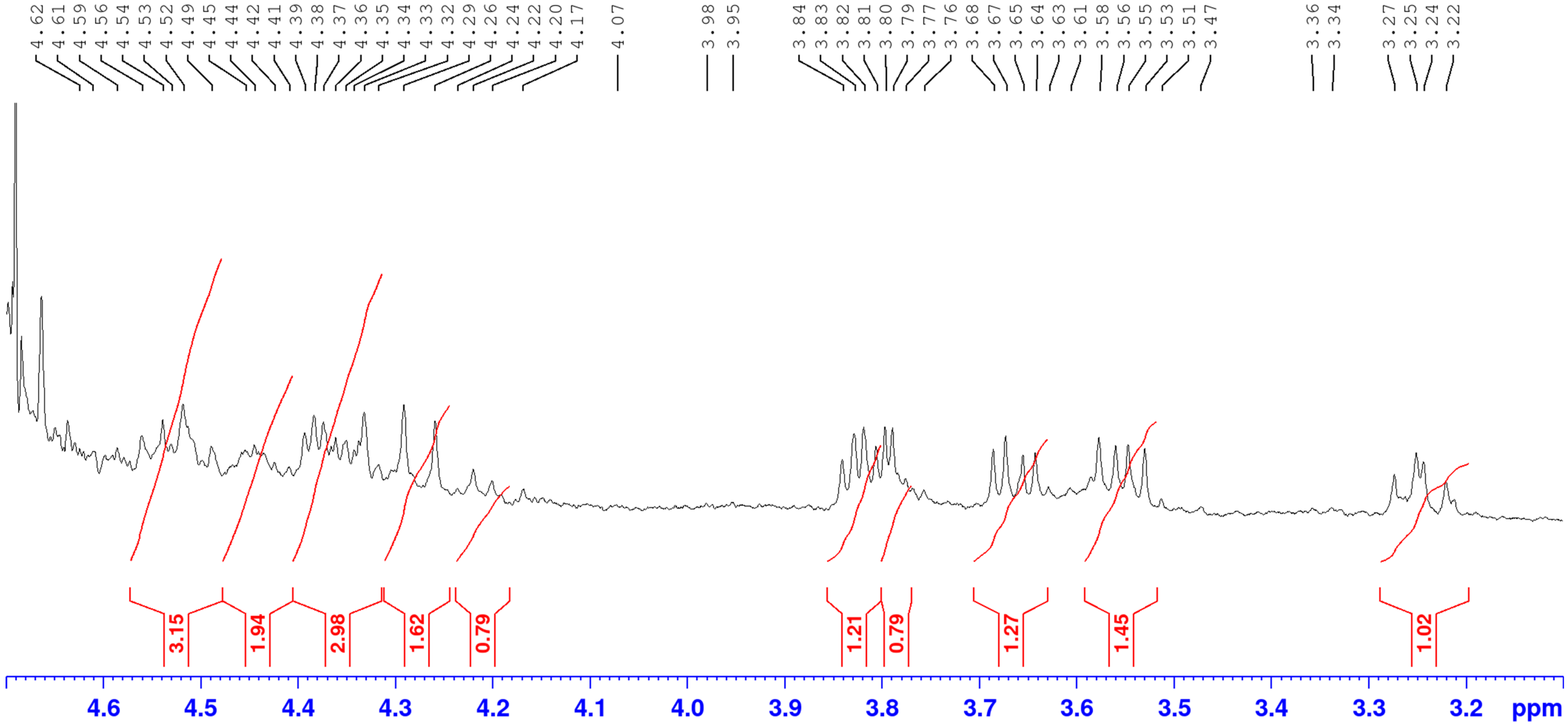

4.62 4.61 4.59 4.56 4.54 4.53 4.52 4.49 4.45 4.44 4.42 4.41 4.39 4.38 4.37 4.36 4.35 4.34 4.33 4.32 4.29 4.26 4.24 4.22 4.20 4.17 4.07 3.98 3.95 3.84 3.83 3.82 3.81 3.80 3.79 3.77 3.76 3.68 3.67 3.65 3.64 3.63 3.61 3.58 3.56 3.55 3.53 3.51 3.47 3.36 3.34 3.27 3.25 3.24 3.22
3.15 1.94 2.98 1.62 0.79 1.21 0.79 1.27 1.45 1.02
4.6 4.5 4.4 4.3 4.2 4.1 4.0 3.9 3.8 3.7 3.6 3.5 3.4 3.3 3.2 ppm

13C-NMR

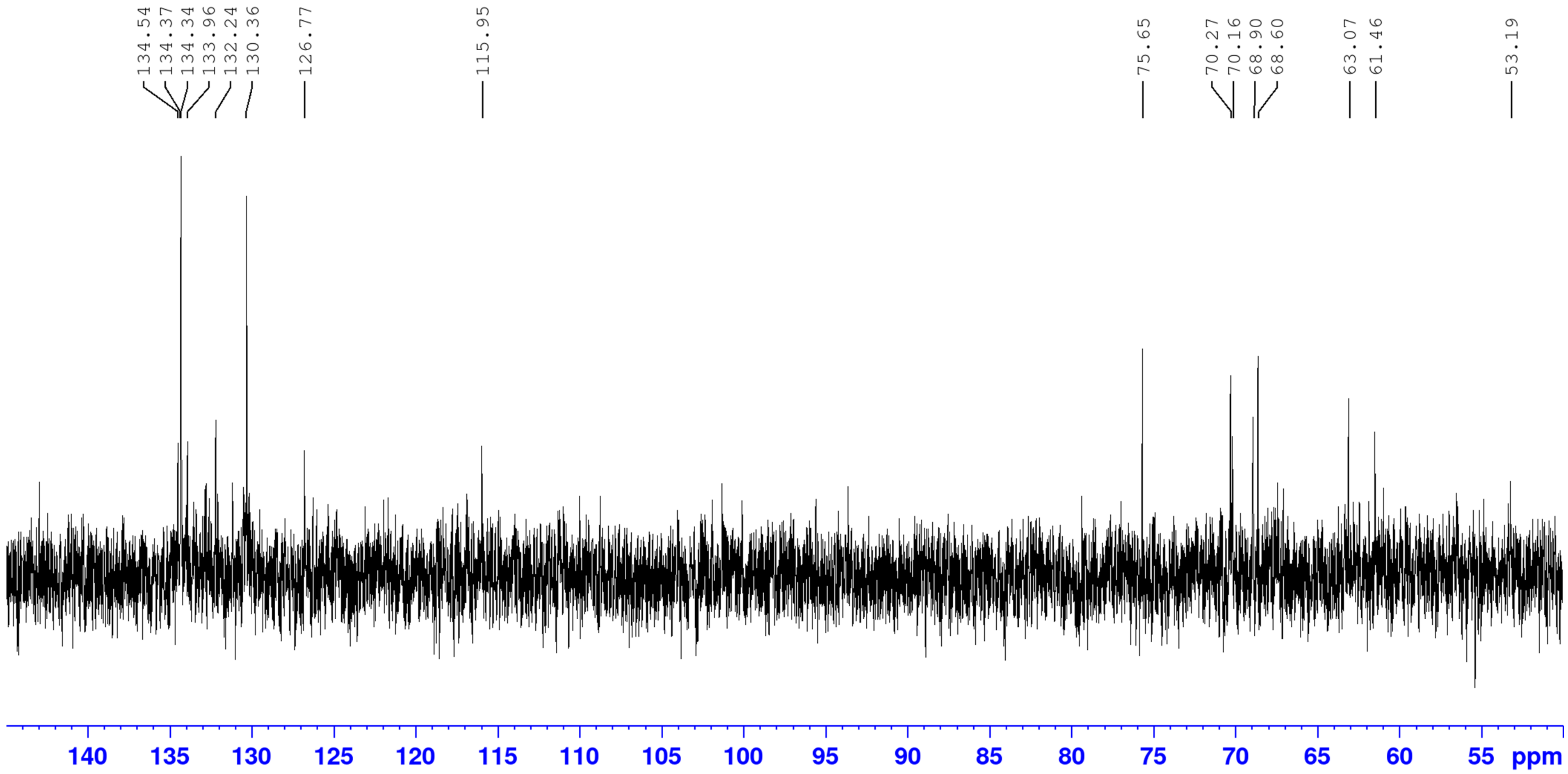
134.54
134.37
134.34
133.96
132.24
130.36
126.77
115.95
75.65
70.27
70.16
68.90
68.60
63.07
61.46
53.19
140
135
130
125
120
115
110
105
100
95
90
85
80
75
70
65
60
55
ppm

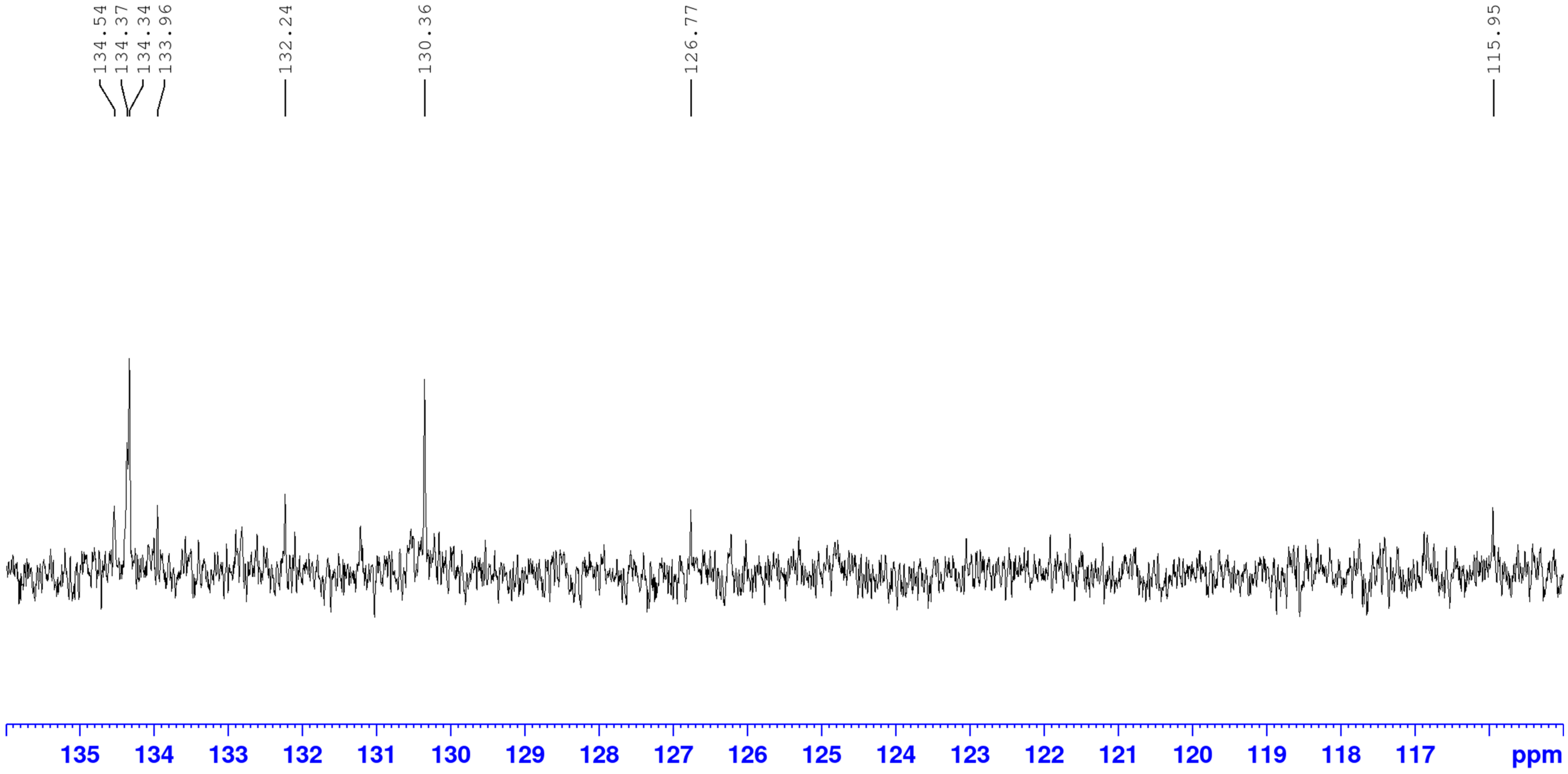
134.54
134.37
134.34
133.96
132.24
130.36
126.77
115.95
135
134
133
132
131
130
129
128
127
126
125
124
123
122
121
120
119
118
117
ppm

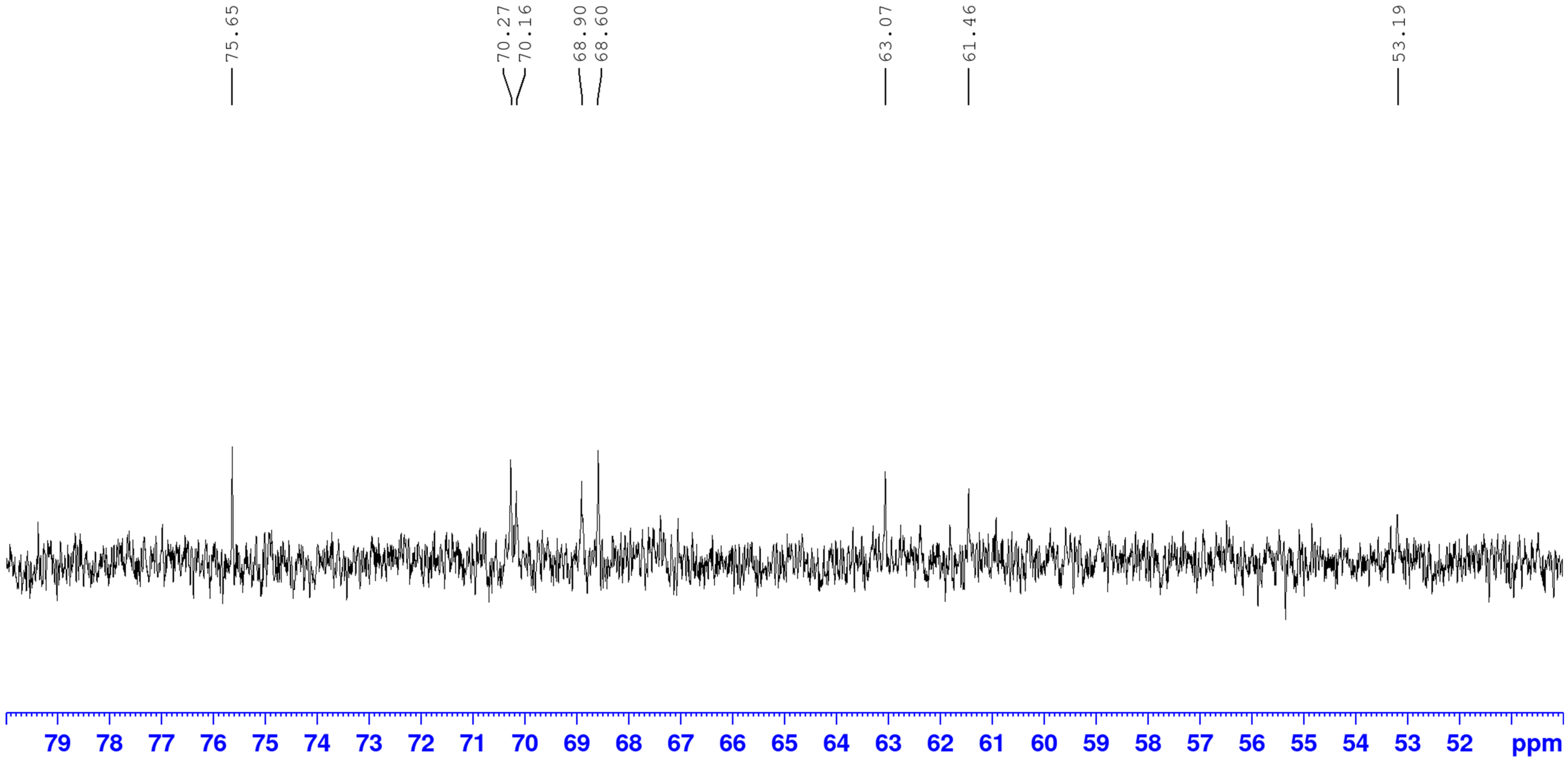
75.65
70.27
70.16
68.90
68.60
63.07
61.46
53.19
79 78 77 76 75 74 73 72 71 70 69 68 67 66 65 64 63 62 61 60 59 58 57 56 55 54 53 52 ppm

^{11}B-NMR

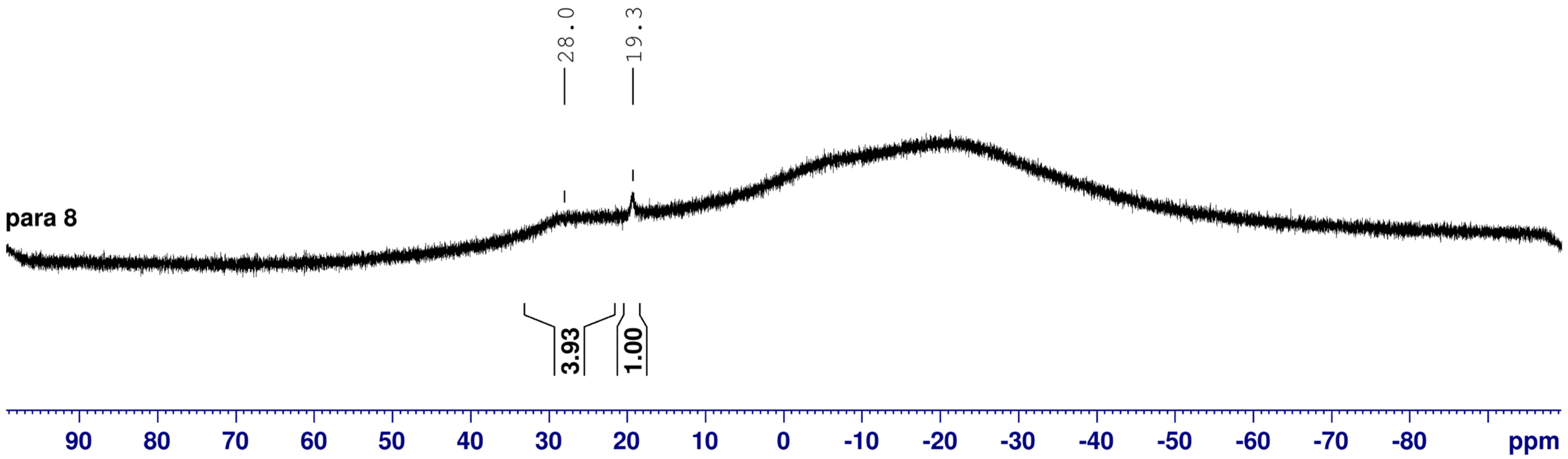
28.0
19.3
para 8
3.93
1.00
90
80
70
60
50
40
30
20
10
0
-10
-20
-30
-40
-50
-60
-70
-80
ppm

COSY

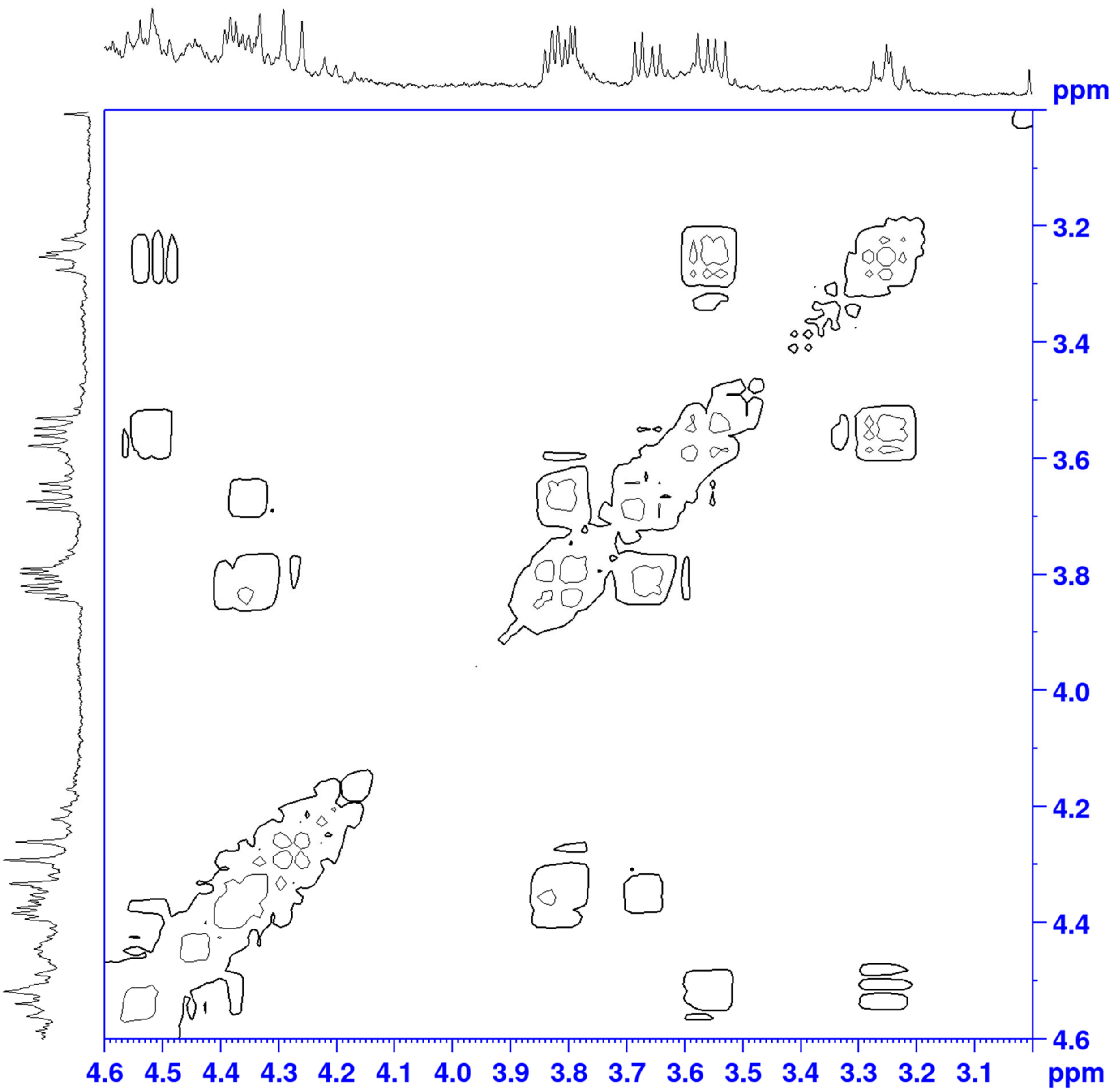
ppm
3.2
3.4
3.6
3.8
4.0
4.2
4.4
4.6
4.6 4.5 4.4 4.3 4.2 4.1 4.0 3.9 3.8 3.7 3.6 3.5 3.4 3.3 3.2 3.1
ppm

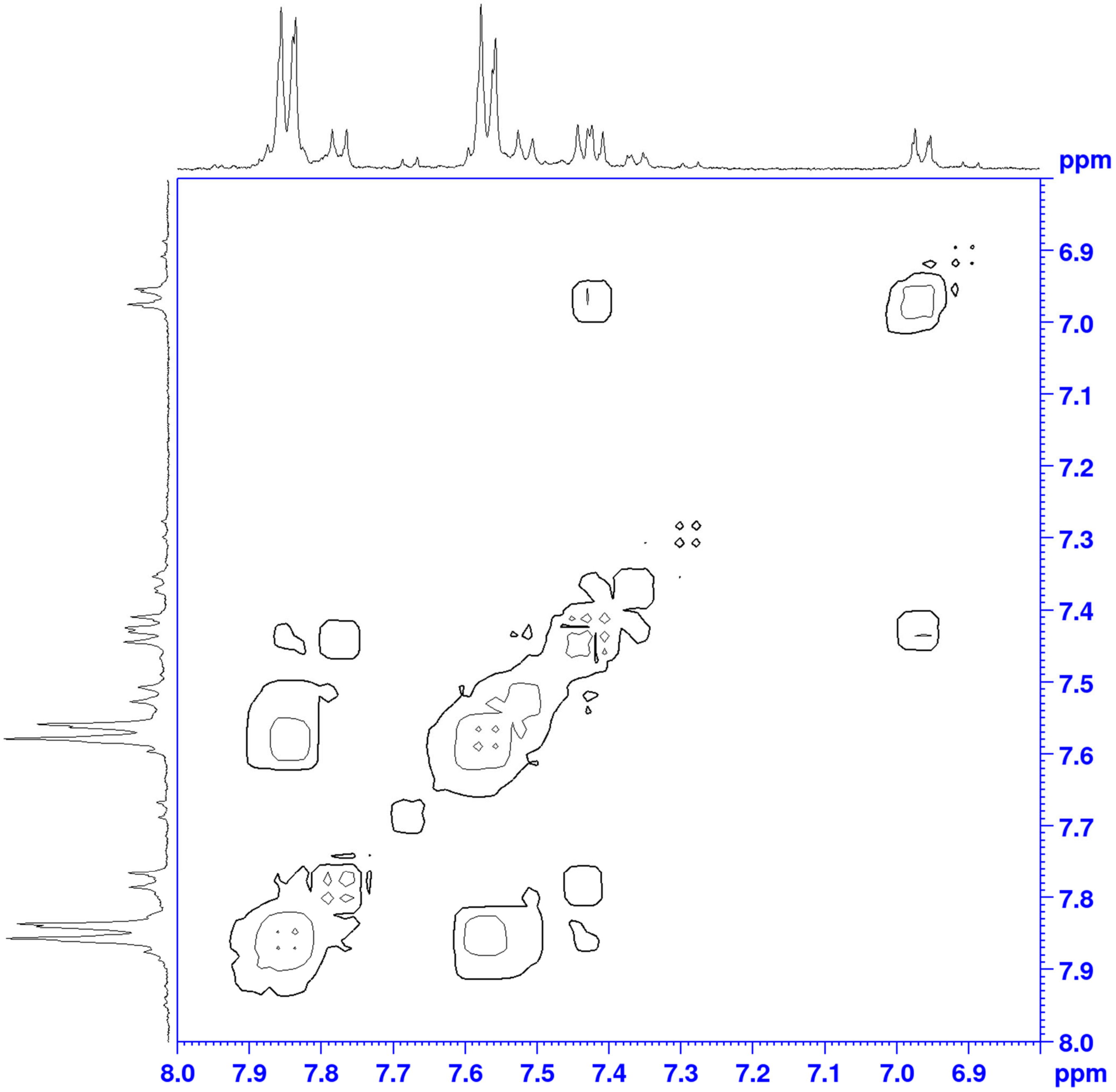
ppm
6.9
7.0
7.1
7.2
7.3
7.4
7.5
7.6
7.7
7.8
7.9
8.0
8.0
7.9
7.8
7.7
7.6
7.5
7.4
7.3
7.2
7.1
7.0
6.9
ppm

HSQC

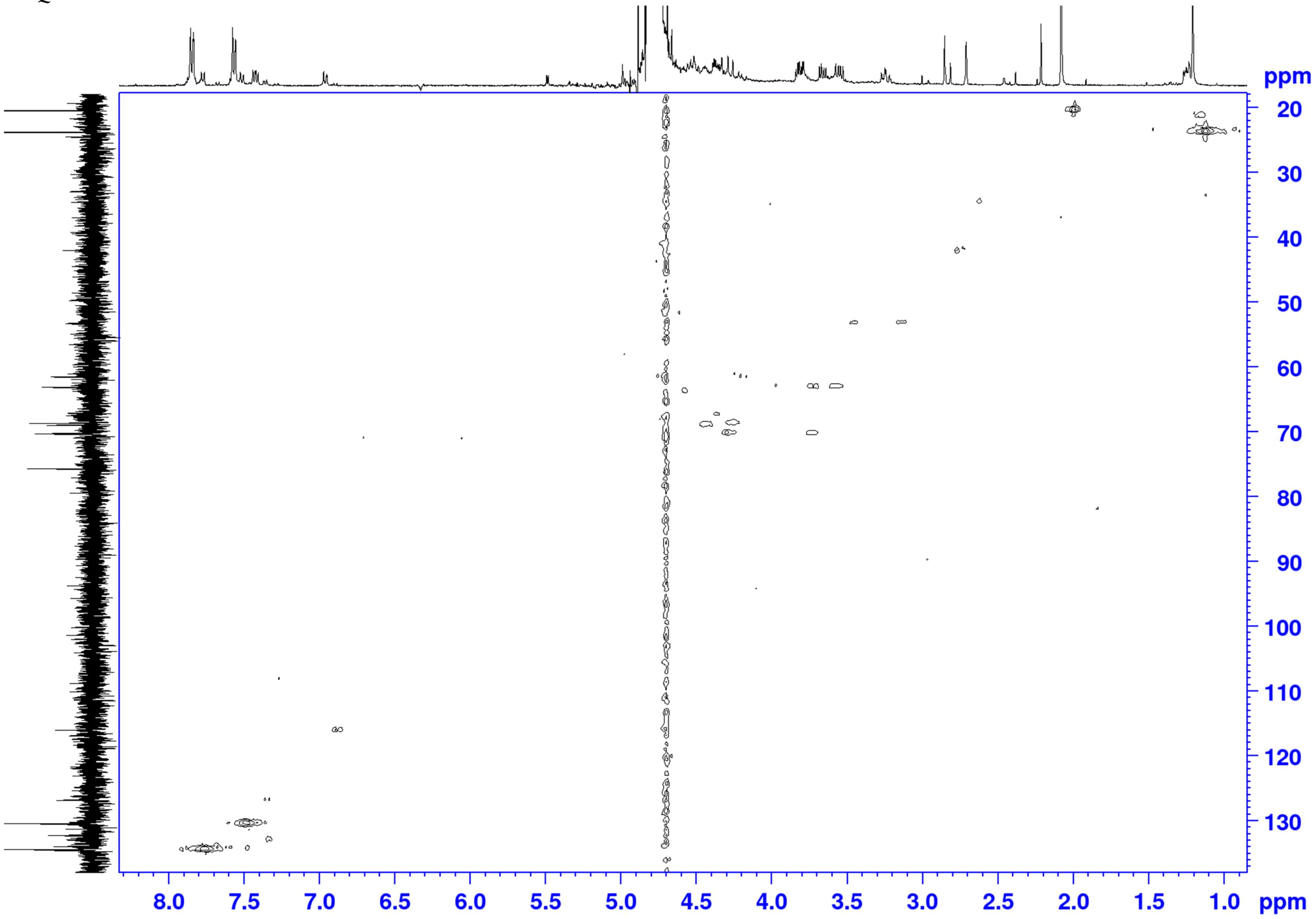
ppm
20
30
40
50
60
70
80
90
100
110
120
130
8.0
7.5
7.0
6.5
6.0
5.5
5.0
4.5
4.0
3.5
3.0
2.5
2.0
1.5
1.0
ppm

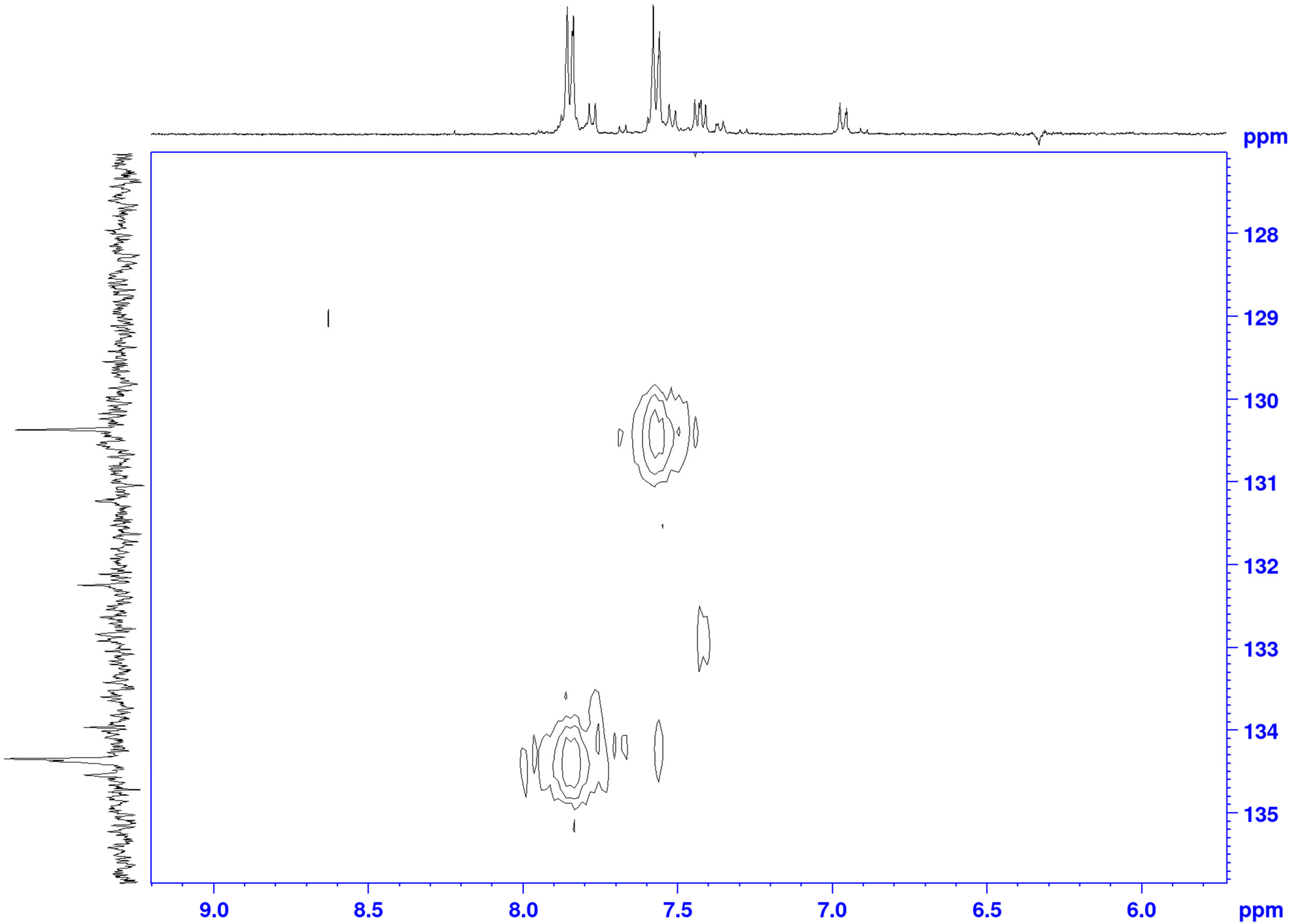

ppm
128
129
130
131
132
133
134
135
9.0
8.5
8.0
7.5
7.0
6.5
6.0
ppm

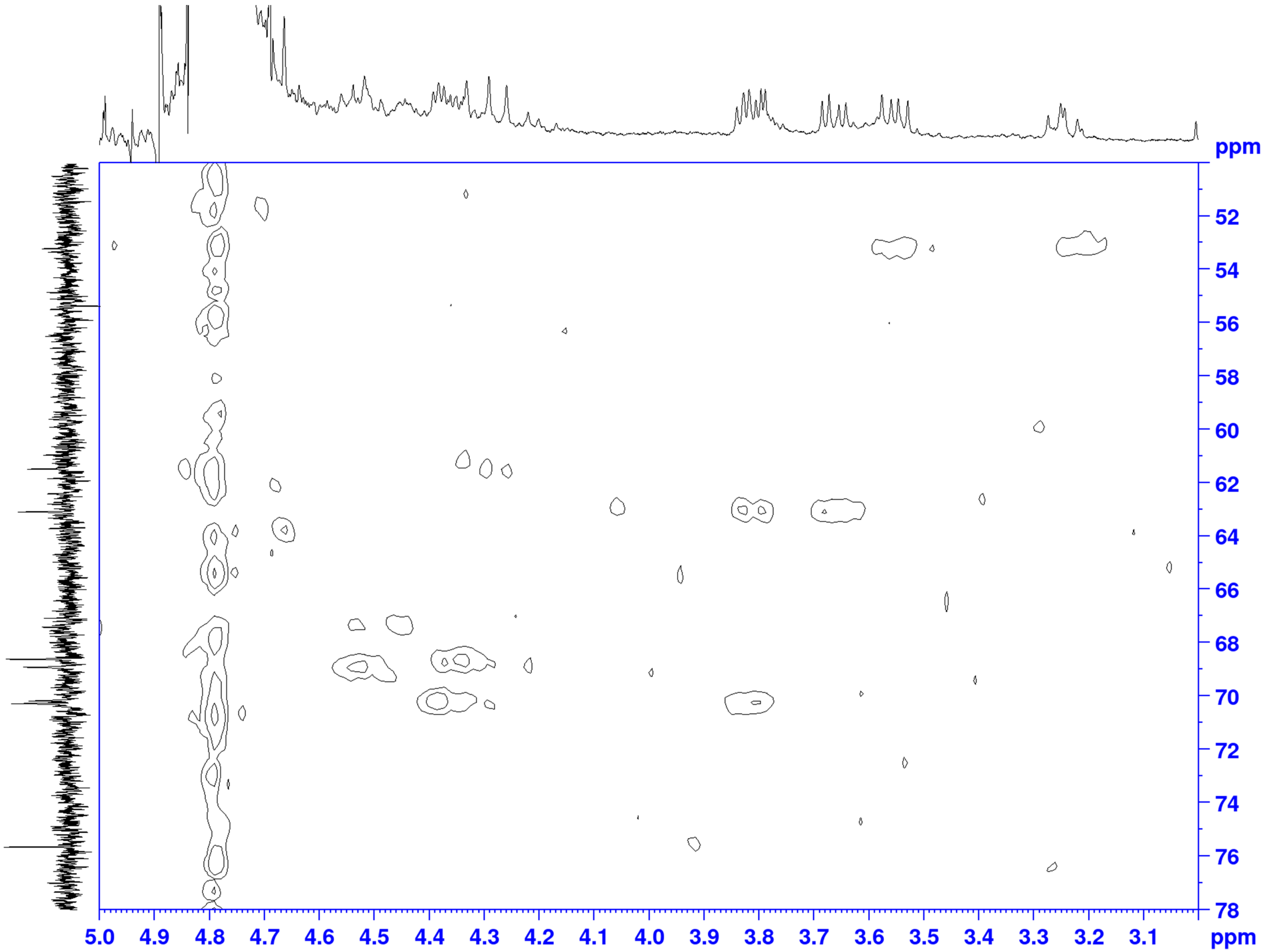
ppm
52
54
56
58
60
62
64
66
68
70
72
74
76
78
5.0 4.9 4.8 4.7 4.6 4.5 4.4 4.3 4.2 4.1 4.0 3.9 3.8 3.7 3.6 3.5 3.4 3.3 3.2 3.1
ppm

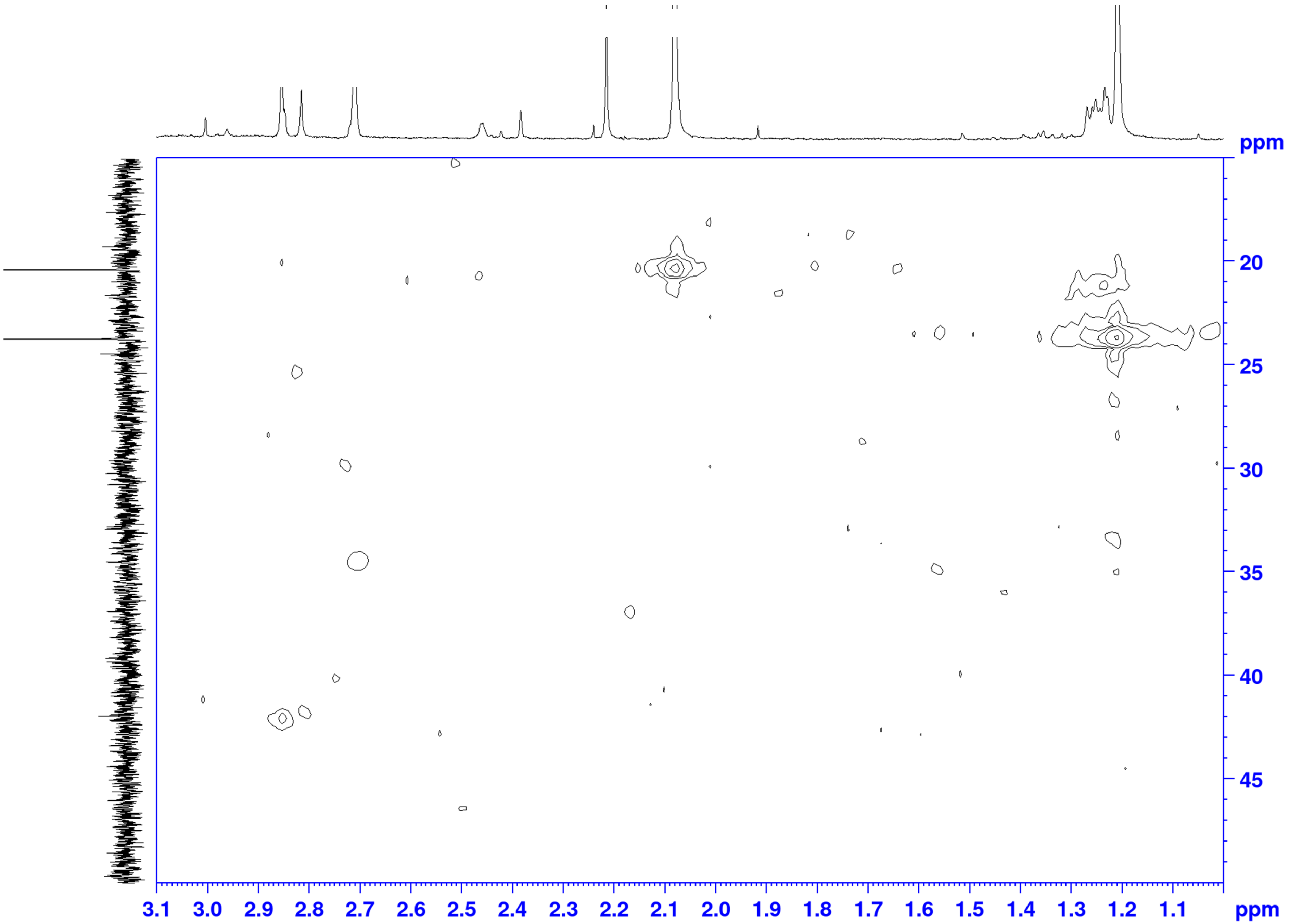
ppm
20
25
30
35
40
45
3.1 3.0 2.9 2.8 2.7 2.6 2.5 2.4 2.3 2.2 2.1 2.0 1.9 1.8 1.7 1.6 1.5 1.4 1.3 1.2 1.1
ppm

Figure S14. ^{1}H-NMR spectrum (400 MHz, D_2O) of *N*-(4-methylphenyl boronic acid)-1,4-dideoxy-1,4-imino-L-gulitol **para 8** with colour-coded signals, highlighting the predominant boronic acid species (orange) and other boronic acid/boronate anion species (shades of green). Section 8.00 ppm to 6.80 ppm is visible with highlighted the principal COSY correlations to hydrogen atoms within the same spin systems.

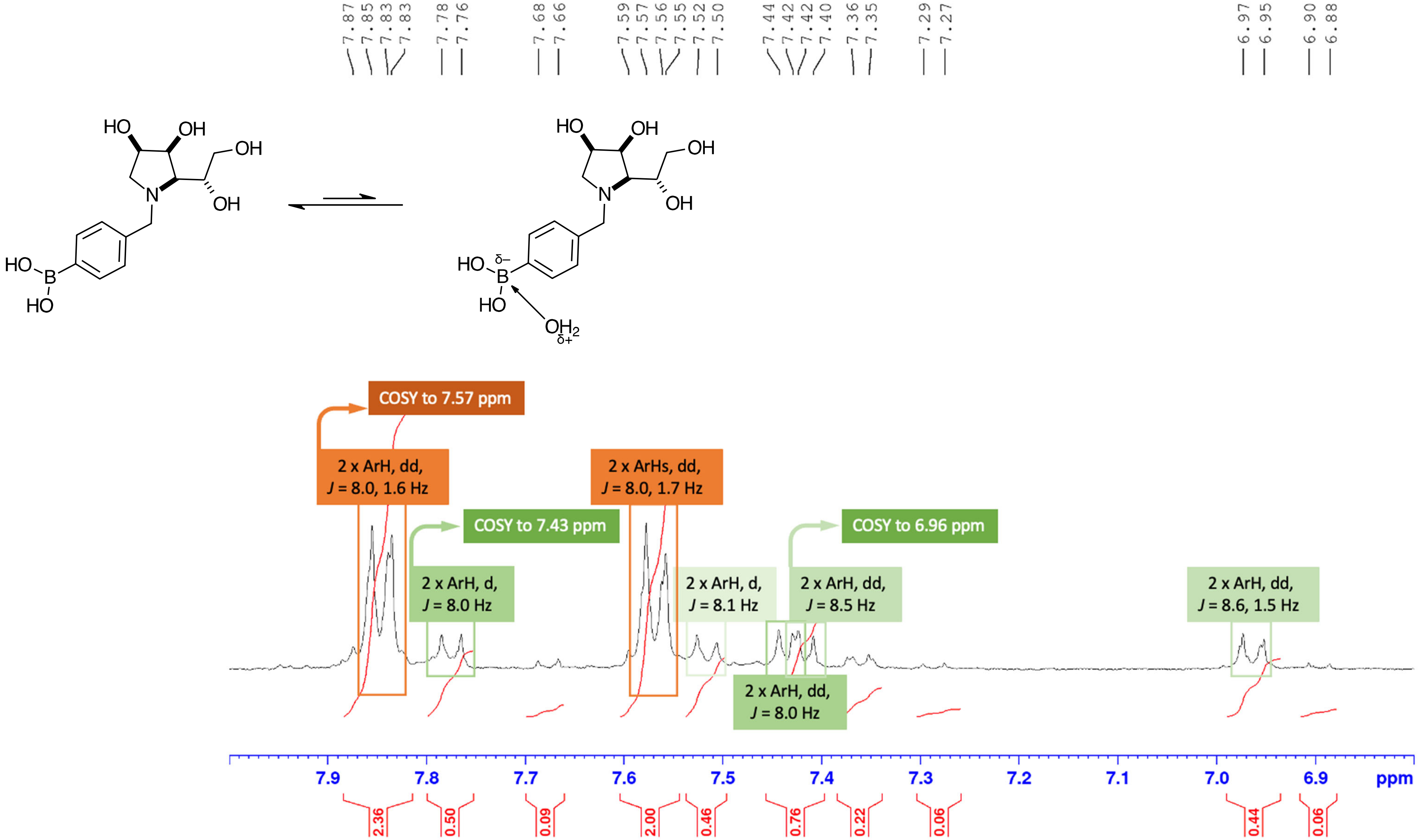

Figure S15. ^{1}H-NMR spectrum (400 MHz, D_2O) of *N*-(4-methylphenyl boronic acid)-1,4-dideoxy-1,4-imino-L-gulitol **para 8** with colour-coded signals, highlighting the predominant boronic acid species (orange) and other boronic acid/boronate anion species (shades of green). A) section 5.10 ppm to 4.00 ppm; B) section 4.10 ppm to 3.10 ppm. Highlighted are also the principal COSY correlations to hydrogen atoms within the same spin systems.

A

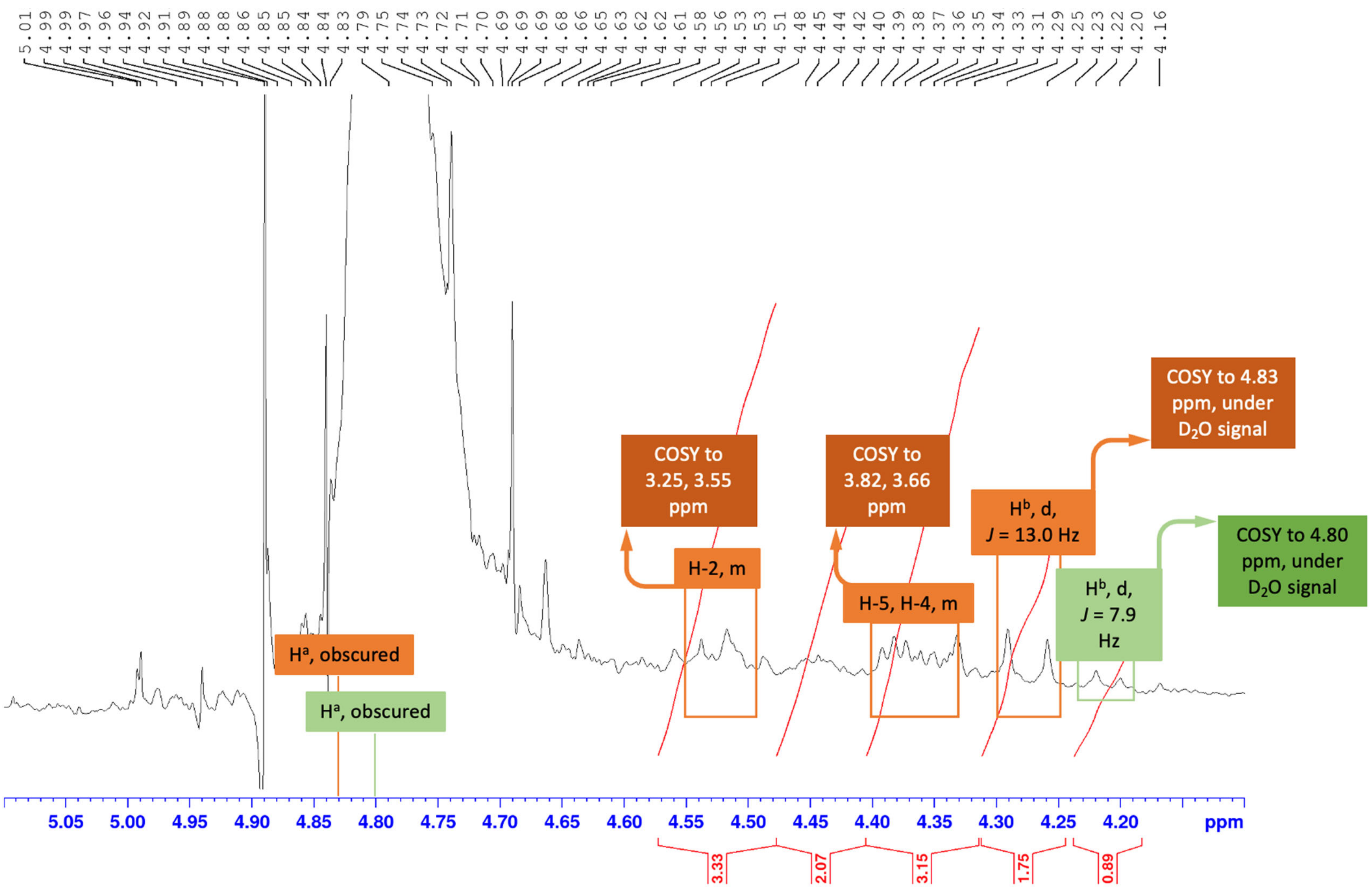

B

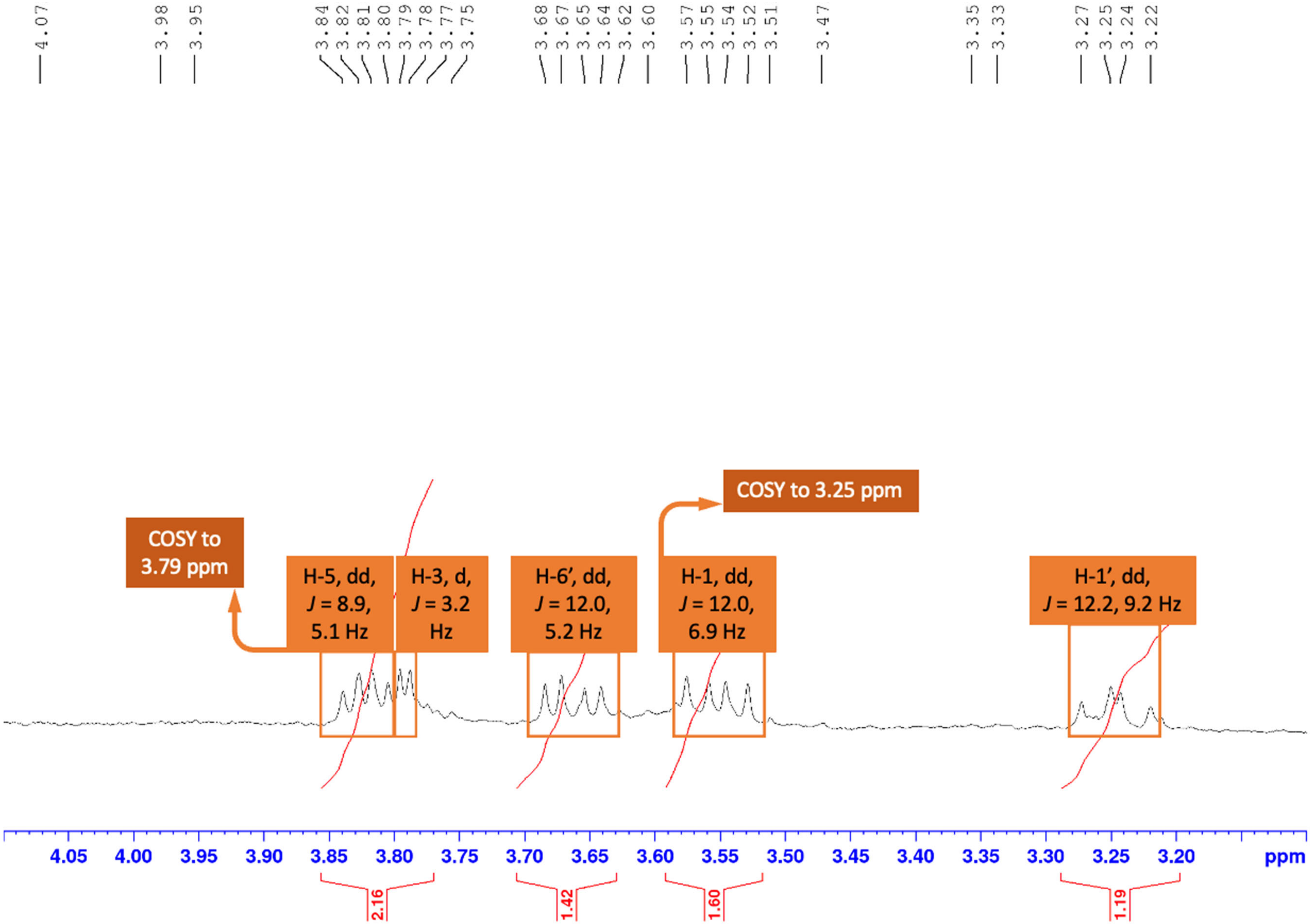
4.07
3.98
3.95
3.84
3.82
3.81
3.80
3.79
3.78
3.77
3.75
3.68
3.67
3.65
3.64
3.62
3.60
3.57
3.55
3.54
3.52
3.51
3.47
3.35
3.33
3.27
3.25
3.24
3.22
COSY to 3.79 ppm
H-5, dd, J = 8.9, 5.1 Hz
H-3, d, J = 3.2 Hz
H-6', dd, J = 12.0, 5.2 Hz
H-1, dd, J = 12.0, 6.9 Hz
COSY to 3.25 ppm
H-1', dd, J = 12.2, 9.2 Hz
4.05
4.00
3.95
3.90
3.85
3.80
3.75
3.70
3.65
3.60
3.55
3.50
3.45
3.40
3.35
3.30
3.25
3.20
ppm
2.16
1.42
1.60
1.19

Figure S16. ^{13}C-NMR spectrum (100 MHz, D_2O) sections of *N*-(4-methylphenyl boronic acid)-1,4-dideoxy-1,4-imino-L-gulitol **para 8**. Only the main set of signals is presented A) section 136.5 ppm to 115.0 ppm; B) section 75.0 ppm to 50.0 ppm.

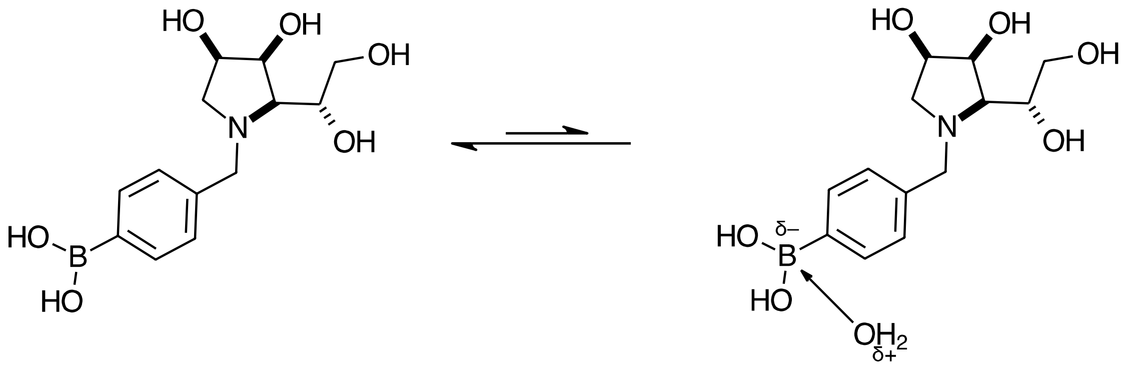

A

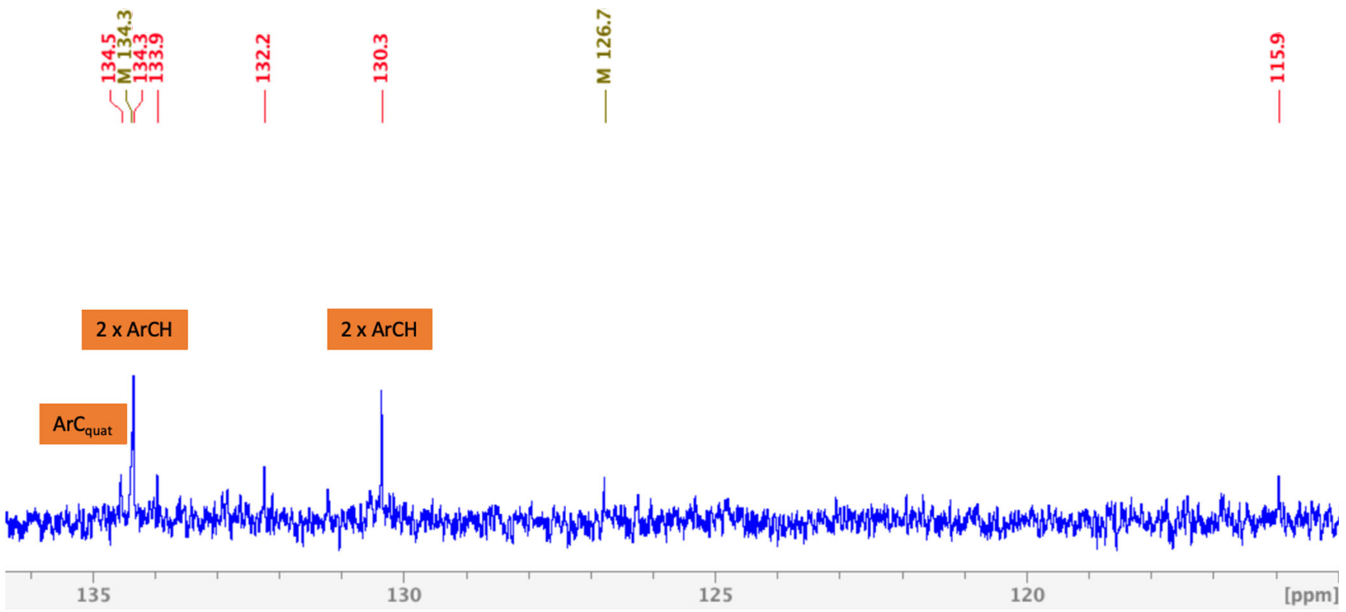

B

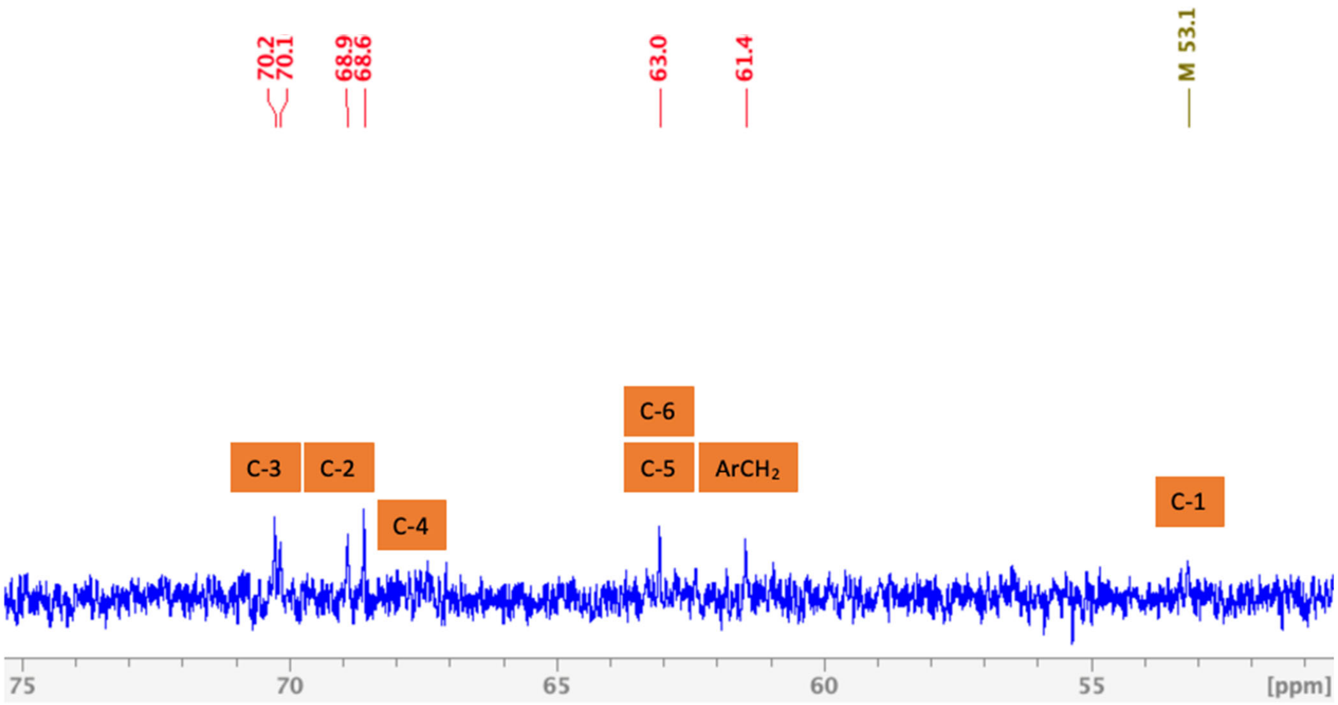
70.2
70.1
68.9
68.6
63.0
61.4
M 53.1
C-6
C-3
C-2
C-5
ArCH2
C-4
C-1
75
70
65
60
55
[ppm]

NMR Experimental details

	3	4	5
^{1}H	F2 - Acquisition Parameters<br>Date_ 20170823<br>Time 10.27<br>INSTRUM spect<br>PROBHD 5 mm PABBO BB-<br>PULPROG zg30<br>TD 65536<br>SOLVENT CDCl3<br>NS 16<br>DS 2<br>SWH 8012.820 Hz<br>FIDRES 0.122266 Hz<br>AQ 4.0894465 sec<br>RG 192.58<br>DW 62.400 usec<br>DE 6.50 usec<br>TE 298.2 K<br>D1 1.00000000 sec<br>TD0 1<br><br>======== CHANNEL f1 ========<br>SFO1 400.1324710 MHz<br>NUC1 1H<br>P1 15.00 usec<br>PLW1 12.50300026 W<br><br>F2 - Processing parameters<br>SI 65536<br>SF 400.1300096 MHz<br>WDW EM<br>SSB 0<br>LB 0.30 Hz<br>GB 0<br>PC 1.00	F2 - Acquisition Parameters<br>Date_ 20171025<br>Time 16.47<br>INSTRUM spect<br>PROBHD 5 mm PABBO BB-<br>PULPROG zg<br>TD 48076<br>SOLVENT D2O<br>NS 8<br>DS 2<br>SWH 6002.401 Hz<br>FIDRES 0.124852 Hz<br>AQ 4.0047307 sec<br>RG 78.18<br>DW 83.300 usec<br>DE 16.70 usec<br>TE 298.1 K<br>D1 5.00000000 sec<br>TD0 1<br><br>======== CHANNEL f1 ========<br>SFO1 400.1320007 MHz<br>NUC1 1H<br>P1 15.00 usec<br>PLW1 12.50300026 W<br><br>F2 - Processing parameters<br>SI 32768<br>SF 400.1299638 MHz<br>WDW EM<br>SSB 0<br>LB 0.30 Hz<br>GB 0<br>PC 1.00	F2 - Acquisition Parameters<br>Date_ 20180207<br>Time 17.02<br>INSTRUM spect<br>PROBHD 5 mm PABBO BB-<br>PULPROG zg30<br>TD 65536<br>SOLVENT D2O<br>NS 16<br>DS 2<br>SWH 8012.820 Hz<br>FIDRES 0.122266 Hz<br>AQ 4.0894465 sec<br>RG 192.58<br>DW 62.400 usec<br>DE 6.50 usec<br>TE 298.2 K<br>D1 1.00000000 sec<br>TD0 1<br><br>======== CHANNEL f1 ========<br>SFO1 400.1324710 MHz<br>NUC1 1H<br>P1 15.00 usec<br>PLW1 12.50300026 W<br><br>F2 - Processing parameters<br>SI 65536<br>SF 400.1299646 MHz<br>WDW EM<br>SSB 0<br>LB 0.30 Hz<br>GB 0<br>PC 1.00

^{13}C	F2 - Acquisition Parameters<br>Date_ 20170222<br>Time 10.28<br>INSTRUM spect<br>PROBHD 5 mm PABBO BB-<br>PULPROG zgpg30<br>TD 65536<br>SOLVENT CDCl3<br>NS 256<br>DS 4<br>SWH 24038.461 Hz<br>FIDRES 0.366798 Hz<br>AQ 1.3631488 sec<br>RG 192.58<br>DW 20.800 usec<br>DE 6.50 usec<br>TE 298.2 K<br>D1 2.00000000 sec<br>D11 0.03000000 sec<br>TD0 1<br><br>======== CHANNEL f1 ========<br>SFO1 100.6238364 MHz<br>NUC1 13C<br>P1 9.80 usec<br>PLW1 60.95399857 W<br><br>======== CHANNEL f2 ========<br>SFO2 400.1316005 MHz<br>NUC2 1H<br>CPDPRG[2 waltz16<br>PCPD2 90.00 usec<br>PLW2 12.50300026 W<br>PLW12 0.34731001 W<br>PLW13 0.28132001 W<br><br>F2 - Processing parameters<br>SI 65536<br>SF 100.6127584 MHz<br>WDW EM<br>SSB 0<br>LB 1.00 Hz<br>GB 0<br>PC 1.40	F2 - Acquisition Parameters<br>Date_ 20171025<br>Time 21.41<br>INSTRUM spect<br>PROBHD 5 mm PABBO BB-<br>PULPROG zgpg30<br>TD 65536<br>SOLVENT D2O<br>NS 4000<br>DS 4<br>SWH 22058.824 Hz<br>FIDRES 0.336591 Hz<br>AQ 1.4854827 sec<br>RG 192.58<br>DW 22.667 usec<br>DE 6.50 usec<br>TE 298.2 K<br>D1 2.00000000 sec<br>D11 0.03000000 sec<br>TD0 1<br><br>======== CHANNEL f1 ========<br>SFO1 100.6223253 MHz<br>NUC1 13C<br>P1 9.80 usec<br>PLW1 60.95399857 W<br><br>======== CHANNEL f2 ========<br>SFO2 400.1316005 MHz<br>NUC2 1H<br>CPDPRG[2 waltz16<br>PCPD2 90.00 usec<br>PLW2 12.50300026 W<br>PLW12 0.34731001 W<br>PLW13 0.28132001 W<br><br>F2 - Processing parameters<br>SI 65536<br>SF 100.6127690 MHz<br>WDW EM<br>SSB 0<br>LB 1.00 Hz<br>GB 0<br>PC 1.40	F2 - Acquisition Parameters<br>Date_ 20180209<br>Time 20.47<br>INSTRUM spect<br>PROBHD 5 mm PABBO BB-<br>PULPROG zgpg30<br>TD 65536<br>SOLVENT D2O<br>NS 3000<br>DS 4<br>SWH 22058.824 Hz<br>FIDRES 0.336591 Hz<br>AQ 1.4854827 sec<br>RG 192.58<br>DW 22.667 usec<br>DE 6.50 usec<br>TE 298.1 K<br>D1 2.00000000 sec<br>D11 0.03000000 sec<br>TD0 1<br><br>======== CHANNEL f1 ========<br>SFO1 100.6223253 MHz<br>NUC1 13C<br>P1 9.80 usec<br>PLW1 60.95399857 W<br><br>======== CHANNEL f2 ========<br>SFO2 400.1316005 MHz<br>NUC2 1H<br>CPDPRG[2 waltz16<br>PCPD2 90.00 usec<br>PLW2 12.50300026 W<br>PLW12 0.34731001 W<br>PLW13 0.28132001 W<br><br>F2 - Processing parameters<br>SI 65536<br>SF 100.6127690 MHz<br>WDW EM<br>SSB 0<br>LB 1.00 Hz<br>GB 0<br>PC 1.40

**DEPT**	F2 - Acquisition Parameters			
	Date_	20170222	20171025	20180209
	Time	10.45	17.04	17.25
	INSTRUM	spect	spect	spect
	PROBHD	5 mm PABBO BB-	5 mm PABBO BB-	5 mm PABBO BB-
	PULPROG	deptqgpsp	deptqgpsp	deptqgpsp
	TD	65536	65536	65536
	SOLVENT	CDCl3	D2O	D2O
	NS	256	256	256
	DS	4	4	4
	SWH	22058.824 Hz	22058.824 Hz	22058.824 Hz
	FIDRES	0.336591 Hz	0.336591 Hz	0.336591 Hz
	AQ	1.4854827 sec	1.4854827 sec	1.4854827 sec
	RG	192.58	192.58	192.58
	DW	22.667 usec	22.667 usec	22.667 usec
	DE	6.50 usec	6.50 usec	6.50 usec
	TE	298.2 K	298.2 K	298.2 K
	CNST2	145.0000000	145.0000000	145.0000000
	CNST12	1.5000000	1.5000000	1.5000000
	D1	2.00000000 sec	2.00000000 sec	2.00000000 sec
	D2	0.00344828 sec	0.00344828 sec	0.00344828 sec
	D12	0.00002000 sec	0.00002000 sec	0.00002000 sec
	D16	0.00020000 sec	0.00020000 sec	0.00020000 sec
	TD0	1	1	1
	======== CHANNEL f1 ========			
	SFO1	100.6223258 MHz	100.6223258 MHz	100.6223258 MHz
	NUC1	13C	13C	13C
	P1	9.80 usec	9.80 usec	9.80 usec
	P13	2000.00 usec	2000.00 usec	2000.00 usec
	PLW0	0 W	0 W	0 W
	PLW1	60.95399857 W	60.95399857 W	60.95399857 W
	SPNAM[5]	Crp60comp.4	Crp60comp.4	Crp60comp.4
	SPOAL5	0.500	0.500	0.500
	SPOFFS5	0 Hz	0 Hz	0 Hz
	SPW5	8.94419956 W	8.94419956 W	8.94419956 W
	======== CHANNEL f2 ========			
	SFO2	400.1316005 MHz	400.1316005 MHz	400.1316005 MHz
	NUC2	1H	1H	1H
	CPDPRG[2	waltz16	waltz16	waltz16
	P0	22.50 usec	22.50 usec	22.50 usec
	P3	15.00 usec	15.00 usec	15.00 usec
	P4	30.00 usec	30.00 usec	30.00 usec
	PCPD2	90.00 usec	90.00 usec	90.00 usec
	PLW2	12.50300026 W	12.50300026 W	12.50300026 W
	PLW12	0.34731001 W	0.34731001 W	0.34731001 W
	====== GRADIENT CHANNEL =====			
	GPNAM[1]	SMSQ10.32	SMSQ10.32	SMSQ10.32
	GPNAM[2]	SMSQ10.32	SMSQ10.32	SMSQ10.32
	GPNAM[3]	SMSQ10.32	SMSQ10.32	SMSQ10.32
	GPZ1	31.00 %	31.00 %	31.00 %
	GPZ2	31.00 %	31.00 %	31.00 %
	GPZ3	31.00 %	31.00 %	31.00 %
	P16	1000.00 usec	1000.00 usec	1000.00 usec
	F2 - Processing parameters			
	SI	65536	65536	65536
	SF	100.6127690 MHz	100.6127690 MHz	100.6127690 MHz
	WDW	EM	EM	EM
	SSB	0	0	0
	LB	1.00 Hz	1.00 Hz	1.00 Hz
	GB	0	0	0
	PC	1.40	1.40	1.40

**COSY**	F2 - Acquisition Parameters<br>Date_ 20170222<br>Time 11.04<br>INSTRUM spect<br>PROBHD 5 mm PABBO BB-<br>PULPROG cosygpqf<br>TD 2048<br>SOLVENT CDCl3<br>NS 1<br>DS 8<br>SWH 4807.692 Hz<br>FIDRES 2.347506 Hz<br>AQ 0.2129920 sec<br>RG 119.51<br>DW 104.000 usec<br>DE 6.50 usec<br>TE 298.0 K<br>D0 0.00000300 sec<br>D1 1.48689198 sec<br>D13 0.00000400 sec<br>D16 0.00020000 sec<br>IN0 0.00020800 sec<br><br>======== CHANNEL f1 ========<br>SFO1 400.1322007 MHz<br>NUC1 1H<br>P0 15.00 usec<br>P1 15.00 usec<br>PLW1 12.50300026 W<br><br>====== GRADIENT CHANNEL =====<br>GPNAM[1] SMSQ10.100<br>GPZ1 10.00 %<br>P16 1000.00 usec<br><br>F1 - Acquisition parameters<br>TD 128<br>SFO1 400.1322 MHz<br>FIDRES 75.120193 Hz<br>SW 12.015 ppm<br>FnMODE QF<br><br>F2 - Processing parameters<br>SI 1024<br>SF 400.1300233 MHz<br>WDW SINE<br>SSB 0<br>LB 0 Hz<br>GB 0<br>PC 1.40<br><br>F1 - Processing parameters<br>SI 1024<br>MC2 QF<br>SF 400.1300374 MHz<br>WDW SINE<br>SSB 0<br>LB 0 Hz<br>GB 0	F2 - Acquisition Parameters<br>Date_ 20171025<br>Time 17.06<br>INSTRUM spect<br>PROBHD 5 mm PABBO BB-<br>PULPROG cosygpqf<br>TD 2048<br>SOLVENT D2O<br>NS 1<br>DS 8<br>SWH 4807.692 Hz<br>FIDRES 2.347506 Hz<br>AQ 0.2129920 sec<br>RG 192.58<br>DW 104.000 usec<br>DE 6.50 usec<br>TE 298.0 K<br>D0 0.00000300 sec<br>D1 1.48689198 sec<br>D13 0.00000400 sec<br>D16 0.00020000 sec<br>IN0 0.00020800 sec<br><br>======== CHANNEL f1 ========<br>SFO1 400.1322007 MHz<br>NUC1 1H<br>P0 15.00 usec<br>P1 15.00 usec<br>PLW1 12.50300026 W<br><br>====== GRADIENT CHANNEL =====<br>GPNAM[1] SMSQ10.100<br>GPZ1 10.00 %<br>P16 1000.00 usec<br><br>F1 - Acquisition parameters<br>TD 128<br>SFO1 400.1322 MHz<br>FIDRES 75.120193 Hz<br>SW 12.015 ppm<br>FnMODE QF<br><br>F2 - Processing parameters<br>SI 1024<br>SF 400.1299232 MHz<br>WDW SINE<br>SSB 0<br>LB 0 Hz<br>GB 0<br>PC 1.40<br><br>F1 - Processing parameters<br>SI 1024<br>MC2 QF<br>SF 400.1299228 MHz<br>WDW SINE<br>SSB 0<br>LB 0 Hz<br>GB 0	F2 - Acquisition Parameters<br>Date_ 20180209<br>Time 17.28<br>INSTRUM spect<br>PROBHD 5 mm PABBO BB-<br>PULPROG cosygpqf<br>TD 2048<br>SOLVENT D2O<br>NS 1<br>DS 8<br>SWH 4807.692 Hz<br>FIDRES 2.347506 Hz<br>AQ 0.2129920 sec<br>RG 192.58<br>DW 104.000 usec<br>DE 6.50 usec<br>TE 298.0 K<br>D0 0.00000300 sec<br>D1 1.48689198 sec<br>D13 0.00000400 sec<br>D16 0.00020000 sec<br>IN0 0.00020800 sec<br><br>======== CHANNEL f1 ========<br>SFO1 400.1322007 MHz<br>NUC1 1H<br>P0 15.00 usec<br>P1 15.00 usec<br>PLW1 12.50300026 W<br><br>====== GRADIENT CHANNEL =====<br>GPNAM[1] SMSQ10.100<br>GPZ1 10.00 %<br>P16 1000.00 usec<br><br>F1 - Acquisition parameters<br>TD 128<br>SFO1 400.1322 MHz<br>FIDRES 75.120193 Hz<br>SW 12.015 ppm<br>FnMODE QF<br><br>F2 - Processing parameters<br>SI 1024<br>SF 400.1299640 MHz<br>WDW SINE<br>SSB 0<br>LB 0 Hz<br>GB 0<br>PC 1.40<br><br>F1 - Processing parameters<br>SI 1024<br>MC2 QF<br>SF 400.1299643 MHz<br>WDW SINE<br>SSB 0<br>LB 0 Hz<br>GB 0

HSQC			
	F2 - Acquisition Parameters	F2 - Acquisition Parameters	F2 - Acquisition Parameters
	Date_ 20170222	Date_ 20171025	Date_ 20180209
	Time 10.48	Time 17.29	Time 20.50
	INSTRUM spect	INSTRUM spect	INSTRUM spect
	PROBHD 5 mm PABBO BB-	PROBHD 5 mm PABBO BB-	PROBHD 5 mm PABBO BB-
	PULPROG hsqcedetgpsisp2.2	PULPROG hsqcetgpsisp2.2	PULPROG hsqcetgpsisp2.2
	TD 2048	TD 2048	TD 2048
	SOLVENT CDCl3	SOLVENT D2O	SOLVENT D2O
	NS 2	NS 2	NS 2
	DS 16	DS 16	DS 16
	SWH 5341.880 Hz	SWH 5341.880 Hz	SWH 5341.880 Hz
	FIDRES 2.608340 Hz	FIDRES 2.608340 Hz	FIDRES 2.608340 Hz
	AQ 0.1916928 sec	AQ 0.1916928 sec	AQ 0.1916928 sec
	RG 192.58	RG 192.58	RG 192.58
	DW 93.600 usec	DW 93.600 usec	DW 93.600 usec
	DE 6.50 usec	DE 6.50 usec	DE 6.50 usec
	TE 298.2 K	TE 298.2 K	TE 298.0 K
	CNST2 145.0000000	CNST2 145.0000000	CNST2 145.0000000
	CNST17 -0.5000000	CNST17 -0.5000000	CNST17 -0.5000000
	D0 0.00000300 sec	D0 0.00000300 sec	D0 0.00000300 sec
	D1 1.50000000 sec	D1 1.50000000 sec	D1 1.50000000 sec
	D4 0.00172414 sec	D4 0.00172414 sec	D4 0.00172414 sec
	D11 0.03000000 sec	D11 0.03000000 sec	D11 0.03000000 sec
	D16 0.00020000 sec	D16 0.00020000 sec	D16 0.00020000 sec
	D21 0.00344828 sec		
	D24 0.00086207 sec	D24 0.00086207 sec	D24 0.00086207 sec
	IN0 0.00003000 sec	IN0 0.00003000 sec	IN0 0.00003000 sec
	-------- CHANNEL f1 --------	======== CHANNEL f1 ========	======== CHANNEL f1 ========
	SFO1 400.1324057 MHz	SFO1 400.1324057 MHz	SFO1 400.1324057 MHz
	NUC1 1H	NUC1 1H	NUC1 1H
	P1 15.00 usec	P1 15.00 usec	P1 15.00 usec
	P2 30.00 usec	P2 30.00 usec	P2 30.00 usec
	P28 1000.00 usec	P28 1000.00 usec	P28 1000.00 usec
	PLW1 12.50300026 W	PLW1 12.50300026 W	PLW1 12.50300026 W
	-------- CHANNEL f2 --------	======== CHANNEL f2 ========	======== CHANNEL f2 ========
	SFO2 100.6202713 MHz	SFO2 100.6202713 MHz	SFO2 100.6202713 MHz
	NUC2 13C	NUC2 13C	NUC2 13C
	CPDPRG[2 bi_p5m4sp_4sp.2	CPDPRG[2 bi_p5m4sp_4sp.2	CPDPRG[2 bi_p5m4sp_4sp.2
	P3 9.80 usec	P3 9.80 usec	P3 9.80 usec
	P14 500.00 usec	P14 500.00 usec	P14 500.00 usec
	P24 2000.00 usec	P24 2000.00 usec	P24 2000.00 usec
	P63 1500.00 usec	P63 1500.00 usec	P63 1500.00 usec
	PLW0 0 W	PLW0 0 W	PLW0 0 W
	PLW2 60.95399857 W	PLW2 60.95399857 W	PLW2 60.95399857 W
	PLW12 0.91469002 W	PLW12 0.91469002 W	PLW12 0.91469002 W
	SPNAM[3] Crp60,0.5,20.1	SPNAM[3] Crp60,0.5,20.1	SPNAM[3] Crp60,0.5,20.1
	SPOAL3 0.500	SPOAL3 0.500	SPOAL3 0.500
	SPOFFS3 0 Hz	SPOFFS3 0 Hz	SPOFFS3 0 Hz
	SPW3 8.94419956 W	SPW3 8.94419956 W	SPW3 8.94419956 W
	SPNAM[7] Crp60comp.4	SPNAM[7] Crp60comp.4	SPNAM[7] Crp60comp.4
	SPOAL7 0.500	SPOAL7 0.500	SPOAL7 0.500
	SPOFFS7 0 Hz	SPOFFS7 0 Hz	SPOFFS7 0 Hz
	SPW7 8.94419956 W	SPW7 8.94419956 W	SPW7 8.94419956 W
	SPNAM[14 Crp32,1.5,20.2	SPNAM[14 Crp32,1.5,20.2	SPNAM[14 Crp32,1.5,20.2
	SPOAL14 0.500	SPOAL14 0.500	SPOAL14 0.500
	SPOFFS14 0 Hz	SPOFFS14 0 Hz	SPOFFS14 0 Hz
	SPW14 3.81620002 W	SPW14 3.81620002 W	SPW14 3.81620002 W
	SPNAM[31 Crp32,1.5,20.2	SPNAM[31 Crp32,1.5,20.2	SPNAM[31 Crp32,1.5,20.2
	SPOAL31 0.500	SPOAL31 0.500	SPOAL31 0.500
	SPOFFS31 0 Hz	SPOFFS31 0 Hz	SPOFFS31 0 Hz
	SPW31 0.95405000 W	SPW31 0.95405000 W	SPW31 0.95405000 W
	------ GRADIENT CHANNEL -----	====== GRADIENT CHANNEL =====	====== GRADIENT CHANNEL =====
	GPNAM[1] SMSQ10.100	GPNAM[1] SMSQ10.100	GPNAM[1] SMSQ10.100
	GPNAM[2] SMSQ10.100	GPNAM[2] SMSQ10.100	GPNAM[2] SMSQ10.100
	GPNAM[3] SMSQ10.100	GPNAM[3] SMSQ10.100	GPNAM[3] SMSQ10.100
	GPNAM[4] SMSQ10.100	GPNAM[4] SMSQ10.100	GPNAM[4] SMSQ10.100
	GPZ1 80.00 %	GPZ1 80.00 %	GPZ1 80.00 %
	GPZ2 20.10 %	GPZ2 20.10 %	GPZ2 20.10 %
	GPZ3 11.00 %	GPZ3 11.00 %	GPZ3 11.00 %
	GPZ4 -5.00 %	GPZ4 -5.00 %	GPZ4 -5.00 %
	P16 1000.00 usec	P16 1000.00 usec	P16 1000.00 usec
	P19 600.00 usec	P19 600.00 usec	P19 600.00 usec
	F1 - Acquisition parameters	F1 - Acquisition parameters	F1 - Acquisition parameters
	TD 256	TD 256	TD 256
	SFO1 100.6203 MHz	SFO1 100.6203 MHz	SFO1 100.6203 MHz
	FIDRES 130.208328 Hz	FIDRES 130.208328 Hz	FIDRES 130.208328 Hz
	SW 165.639 ppm	SW 165.639 ppm	SW 165.639 ppm
	FnMODE Echo-Antiecho	FnMODE Echo-Antiecho	FnMODE Echo-Antiecho
	F2 - Processing parameters	F2 - Processing parameters	F2 - Processing parameters
	SI 1024	SI 1024	SI 1024
	SF 400.1300000 MHz	SF 400.1299264 MHz	SF 400.1299652 MHz
	WDW QSINE	WDW QSINE	WDW QSINE
	SSB 2	SSB 2	SSB 2
	LB 0 Hz	LB 0 Hz	LB 0 Hz
	GB 0	GB 0	GB 0
	PC 1.40	PC 1.40	PC 1.40
	F1 - Processing parameters	F1 - Processing parameters	F1 - Processing parameters
	SI 1024	SI 1024	SI 1024
	MC2 echo-antiecho	MC2 echo-antiecho	MC2 echo-antiecho
	SF 100.6127690 MHz	SF 100.6127711 MHz	SF 100.6127690 MHz
	WDW QSINE	WDW QSINE	WDW QSINE
	SSB 2	SSB 2	SSB 2
	LB 0 Hz	LB 0 Hz	LB 0 Hz
	GB 0	GB 0	GB 0

**HMBC**	NA	F2 - Acquisition Parameters<br>Date_ 20171025<br>Time 17.12<br>INSTRUM spect<br>PROBHD 5 mm PABBO BB-<br>PULPROG hmbcgpl2ndqf<br>TD 2048<br>SOLVENT D2O<br>NS 4<br>DS 16<br>SWH 4807.692 Hz<br>FIDRES 2.347506 Hz<br>AQ 0.2129920 sec<br>RG 192.58<br>DW 104.000 usec<br>DE 6.50 usec<br>TE 298.1 K<br>CNST6 125.0000000<br>CNST7 165.0000000<br>CNST13 7.5000000<br>D0 0.00000300 sec<br>D1 1.50000000 sec<br>D6 0.06666667 sec<br>D16 0.00020000 sec<br>IN0 0.00002240 sec<br><br>======== CHANNEL f1 ========<br>SFO1 400.1322007 MHz<br>NUC1 1H<br>P1 15.00 usec<br>P2 30.00 usec<br>PLW1 12.50300026 W<br><br>======== CHANNEL f2 ========<br>SFO2 100.6228119 MHz<br>NUC2 13C<br>P3 9.80 usec<br>PLW2 60.95399857 W<br><br>====== GRADIENT CHANNEL =====<br>GPNAM[1] SMSQ10.100<br>GPNAM[2] SMSQ10.100<br>GPNAM[3] SMSQ10.100<br>GPNAM[4] SMSQ10.100<br>GPNAM[5] SMSQ10.100<br>GPNAM[6] SMSQ10.100<br>GPZ1 50.00 %<br>GPZ2 30.00 %<br>GPZ3 40.10 %<br>GPZ4 15.00 %<br>GPZ5 -10.00 %<br>GPZ6 -5.00 %<br>P16 1000.00 usec<br><br>F1 - Acquisition parameters<br>TD 128<br>SFO1 100.6228 MHz<br>FIDRES 348.772308 Hz<br>SW 221.833 ppm<br>FnMODE QF<br><br>F2 - Processing parameters<br>SI 2048<br>SF 400.1300000 MHz<br>WDW SINE<br>SSB 0<br>LB 0 Hz<br>GB 0<br>PC 1.40<br><br>F1 - Processing parameters<br>SI 1024<br>MC2 QF<br>SF 100.6127690 MHz<br>WDW SINE<br>SSB 0<br>LB 0 Hz<br>GB 0	F2 - Acquisition Parameters<br>Date_ 20180209<br>Time 17.33<br>INSTRUM spect<br>PROBHD 5 mm PABBO BB-<br>PULPROG hmbcgpl2ndqf<br>TD 2048<br>SOLVENT D2O<br>NS 4<br>DS 16<br>SWH 4807.692 Hz<br>FIDRES 2.347506 Hz<br>AQ 0.2129920 sec<br>RG 192.58<br>DW 104.000 usec<br>DE 6.50 usec<br>TE 298.1 K<br>CNST6 125.0000000<br>CNST7 165.0000000<br>CNST13 7.5000000<br>D0 0.00000300 sec<br>D1 1.50000000 sec<br>D6 0.06666667 sec<br>D16 0.00020000 sec<br>IN0 0.00002240 sec<br><br>======== CHANNEL f1 ========<br>SFO1 400.1322007 MHz<br>NUC1 1H<br>P1 15.00 usec<br>P2 30.00 usec<br>PLW1 12.50300026 W<br><br>======== CHANNEL f2 ========<br>SFO2 100.6228119 MHz<br>NUC2 13C<br>P3 9.80 usec<br>PLW2 60.95399857 W<br><br>====== GRADIENT CHANNEL =====<br>GPNAM[1] SMSQ10.100<br>GPNAM[2] SMSQ10.100<br>GPNAM[3] SMSQ10.100<br>GPNAM[4] SMSQ10.100<br>GPNAM[5] SMSQ10.100<br>GPNAM[6] SMSQ10.100<br>GPZ1 50.00 %<br>GPZ2 30.00 %<br>GPZ3 40.10 %<br>GPZ4 15.00 %<br>GPZ5 -10.00 %<br>GPZ6 -5.00 %<br>P16 1000.00 usec<br><br>F1 - Acquisition parameters<br>TD 128<br>SFO1 100.6228 MHz<br>FIDRES 348.772308 Hz<br>SW 221.833 ppm<br>FnMODE QF<br><br>F2 - Processing parameters<br>SI 2048<br>SF 400.1300000 MHz<br>WDW SINE<br>SSB 0<br>LB 0 Hz<br>GB 0<br>PC 1.40<br><br>F1 - Processing parameters<br>SI 1024<br>MC2 QF<br>SF 100.6127690 MHz<br>WDW SINE<br>SSB 0<br>LB 0 Hz<br>GB 0

<table>
<tr><th></th><th>para 6</th><th>para 7</th><th>para 8</th></tr>
<tr><td>^{1}H</td><td>F2 - Acquisition Parameters
Date_ 20171025
Time 15.39
INSTRUM spect
PROBHD 5 mm PABBO BB-
PULPROG zg
TD 48076
SOLVENT CDCl3
NS 8
DS 2
SWH 6002.401 Hz
FIDRES 0.124852 Hz
AQ 4.0047307 sec
RG 23.44
DW 83.300 usec
DE 16.70 usec
TE 298.2 K
D1 5.00000000 sec
TD0 1

======== CHANNEL f1 ========
SFO1 400.1320007 MHz
NUC1 1H
P1 15.00 usec
PLW1 12.50300026 W

F2 - Processing parameters
SI 32768
SF 400.1300096 MHz
WDW EM
SSB 0
LB 0.30 Hz
GB 0
PC 1.00</td><td>F2 - Acquisition Parameters
Date_ 20180209
Time 10.59
INSTRUM spect
PROBHD 5 mm PABBO BB-
PULPROG zg30
TD 65536
SOLVENT D2O
NS 16
DS 2
SWH 8012.820 Hz
FIDRES 0.122266 Hz
AQ 4.0894465 sec
RG 86.04
DW 62.400 usec
DE 6.50 usec
TE 298.2 K
D1 1.00000000 sec
TD0 1

======== CHANNEL f1 ========
SFO1 400.1324710 MHz
NUC1 1H
P1 15.00 usec
PLW1 12.50300026 W

F2 - Processing parameters
SI 65536
SF 400.1299656 MHz
WDW EM
SSB 0
LB 0.30 Hz
GB 0
PC 1.00</td><td>F2 - Acquisition Parameters
Date_ 20180221
Time 11.07
INSTRUM spect
PROBHD 5 mm PABBO BB-
PULPROG zg30
TD 65536
SOLVENT D2O
NS 16
DS 2
SWH 8012.820 Hz
FIDRES 0.122266 Hz
AQ 4.0894465 sec
RG 33.39
DW 62.400 usec
DE 6.50 usec
TE 298.2 K
D1 1.00000000 sec
TD0 1

======== CHANNEL f1 ========
SFO1 400.1324710 MHz
NUC1 1H
P1 15.00 usec
PLW1 12.50300026 W

F2 - Processing parameters
SI 65536
SF 400.1300000 MHz
WDW EM
SSB 0
LB 0.30 Hz
GB 0
PC 1.00</td></tr>
<tr><td>^{13}C</td><td>F2 - Acquisition Parameters
Date_ 20171027
Time 21.00
INSTRUM spect
PROBHD 5 mm PABBO BB-
PULPROG zgpg30
TD 65536
SOLVENT CDCl3
NS 4000
DS 4
SWH 22058.824 Hz
FIDRES 0.336591 Hz
AQ 1.4854827 sec
RG 192.58
DW 22.667 usec
DE 6.50 usec
TE 298.2 K
D1 2.00000000 sec
D11 0.03000000 sec
TD0 1

======== CHANNEL f1 ========
SFO1 100.6223253 MHz
NUC1 13C
P1 9.80 usec
PLW1 60.95399857 W

======== CHANNEL f2 ========
SFO2 400.1316005 MHz
NUC2 1H
CPDPRG[2 waltz16
PCPD2 90.00 usec
PLW2 12.50300026 W
PLW12 0.34731001 W
PLW13 0.28132001 W

F2 - Processing parameters
SI 65536
SF 100.6127589 MHz
WDW EM
SSB 0
LB 1.00 Hz
GB 0
PC 1.40</td><td>F2 - Acquisition Parameters
Date_ 20180213
Time 23.03
INSTRUM spect
PROBHD 5 mm PABBO BB-
PULPROG zgpg30
TD 65536
SOLVENT D2O
NS 3000
DS 4
SWH 22058.824 Hz
FIDRES 0.336591 Hz
AQ 1.4854827 sec
RG 192.58
DW 22.667 usec
DE 6.50 usec
TE 298.2 K
D1 2.00000000 sec
D11 0.03000000 sec
TD0 1

======== CHANNEL f1 ========
SFO1 100.6223253 MHz
NUC1 13C
P1 9.80 usec
PLW1 60.95399857 W

======== CHANNEL f2 ========
SFO2 400.1316005 MHz
NUC2 1H
CPDPRG[2 waltz16
PCPD2 90.00 usec
PLW2 12.50300026 W
PLW12 0.34731001 W
PLW13 0.28132001 W

F2 - Processing parameters
SI 65536
SF 100.6127690 MHz
WDW EM
SSB 0
LB 1.00 Hz
GB 0
PC 1.40</td><td>F2 - Acquisition Parameters
Date_ 20180221
Time 20.00
INSTRUM spect
PROBHD 5 mm PABBO BB-
PULPROG zgpg30
TD 65536
SOLVENT D2O
NS 3000
DS 4
SWH 22058.824 Hz
FIDRES 0.336591 Hz
AQ 1.4854827 sec
RG 192.58
DW 22.667 usec
DE 6.50 usec
TE 298.1 K
D1 2.00000000 sec
D11 0.03000000 sec
TD0 1

======== CHANNEL f1 ========
SFO1 100.6223253 MHz
NUC1 13C
P1 9.80 usec
PLW1 60.95399857 W

======== CHANNEL f2 ========
SFO2 400.1316005 MHz
NUC2 1H
CPDPRG[2 waltz16
PCPD2 90.00 usec
PLW2 12.50300026 W
PLW12 0.34731001 W
PLW13 0.28132001 W

F2 - Processing parameters
SI 65536
SF 100.6127690 MHz
WDW EM
SSB 0
LB 1.00 Hz
GB 0
PC 1.40</td></tr>
</table>

**DEPT**	F2 - Acquisition Parameters		F2 - Acquisition Parameters	F2 - Acquisition Parameters
	Date_	20171025	20180209	20180221
	Time	15.56	15.41	16.18
	INSTRUM	spect	spect	spect
	PROBHD	5 mm PABBO BB-	5 mm PABBO BB-	5 mm PABBO BB-
	PULPROG	deptqgpsp	deptqgpsp	deptqgpsp
	TD	65536	65536	65536
	SOLVENT	CDCl3	D2O	D2O
	NS	256	256	256
	DS	4	4	4
	SWH	22058.824 Hz	22058.824 Hz	22058.824 Hz
	FIDRES	0.336591 Hz	0.336591 Hz	0.336591 Hz
	AQ	1.4854827 sec	1.4854827 sec	1.4854827 sec
	RG	192.58	192.58	192.58
	DW	22.667 usec	22.667 usec	22.667 usec
	DE	6.50 usec	6.50 usec	6.50 usec
	TE	298.2 K	298.2 K	298.1 K
	CNST2	145.0000000	145.0000000	145.0000000
	CNST12	1.5000000	1.5000000	1.5000000
	D1	2.00000000 sec	2.00000000 sec	2.00000000 sec
	D2	0.00344828 sec	0.00344828 sec	0.00344828 sec
	D12	0.00002000 sec	0.00002000 sec	0.00002000 sec
	D16	0.00020000 sec	0.00020000 sec	0.00020000 sec
	TD0	1	1	1
	======== CHANNEL f1 ========		======== CHANNEL f1 ========	======== CHANNEL f1 ========
	SFO1	100.6223258 MHz	100.6223258 MHz	100.6223258 MHz
	NUC1	13C	13C	13C
	P1	9.80 usec	9.80 usec	9.80 usec
	P13	2000.00 usec	2000.00 usec	2000.00 usec
	PLW0	0 W	0 W	0 W
	PLW1	60.95399857 W	60.95399857 W	60.95399857 W
	SPNAM[5]	Crp60comp.4	Crp60comp.4	Crp60comp.4
	SPOAL5	0.500	0.500	0.500
	SPOFFS5	0 Hz	0 Hz	0 Hz
	SPW5	8.94419956 W	8.94419956 W	8.94419956 W
	======== CHANNEL f2 ========		======== CHANNEL f2 ========	======== CHANNEL f2 ========
	SFO2	400.1316005 MHz	400.1316005 MHz	400.1316005 MHz
	NUC2	1H	1H	1H
	CPDPRG[2	waltz16	waltz16	waltz16
	P0	22.50 usec	22.50 usec	22.50 usec
	P3	15.00 usec	15.00 usec	15.00 usec
	P4	30.00 usec	30.00 usec	30.00 usec
	PCPD2	90.00 usec	90.00 usec	90.00 usec
	PLW2	12.50300026 W	12.50300026 W	12.50300026 W
	PLW12	0.34731001 W	0.34731001 W	0.34731001 W
	====== GRADIENT CHANNEL =====		====== GRADIENT CHANNEL =====	====== GRADIENT CHANNEL =====
	GPNAM[1]	SMSQ10.32	SMSQ10.32	SMSQ10.32
	GPNAM[2]	SMSQ10.32	SMSQ10.32	SMSQ10.32
	GPNAM[3]	SMSQ10.32	SMSQ10.32	SMSQ10.32
	GPZ1	31.00 %	31.00 %	31.00 %
	GPZ2	31.00 %	31.00 %	31.00 %
	GPZ3	31.00 %	31.00 %	31.00 %
	P16	1000.00 usec	1000.00 usec	1000.00 usec
	F2 - Processing parameters		F2 - Processing parameters	F2 - Processing parameters
	SI	65536	65536	65536
	SF	100.6127584 MHz	100.6127690 MHz	100.6127690 MHz
	WDW	EM	EM	EM
	SSB	0	0	0
	LB	1.00 Hz	1.00 Hz	1.00 Hz
	GB	0	0	0
	PC	1.40	1.40	1.40

^{11}**B**	F2 - Acquisition Parameters<br>Date_ 20171026<br>Time 5.49<br>INSTRUM spect<br>PROBHD 5 mm PABBO BB-<br>PULPROG zg<br>TD 65536<br>SOLVENT CDCl3<br>NS 128<br>DS 4<br>SWH 25510.203 Hz<br>FIDRES 0.389255 Hz<br>AQ 1.2845056 sec<br>RG 192.58<br>DW 19.600 usec<br>DE 6.50 usec<br>TE 298.1 K<br>D1 1.00000000 sec<br>TD0 1<br><br>======== CHANNEL f1 ========<br>SFO1 128.3776052 MHz<br>NUC1 11B<br>P1 17.05 usec<br>PLW1 11.69499969 W<br><br>F2 - Processing parameters<br>SI 32768<br>SF 128.3776052 MHz<br>WDW no<br>SSB 0<br>LB 0 Hz<br>GB 0<br>PC 1.40	F2 - Acquisition Parameters<br>Date_ 20180212<br>Time 5.50<br>INSTRUM spect<br>PROBHD 5 mm PABBO BB-<br>PULPROG zg<br>TD 65536<br>SOLVENT D2O<br>NS 128<br>DS 4<br>SWH 25510.203 Hz<br>FIDRES 0.389255 Hz<br>AQ 1.2845056 sec<br>RG 192.58<br>DW 19.600 usec<br>DE 6.50 usec<br>TE 298.1 K<br>D1 1.00000000 sec<br>TD0 1<br><br>======== CHANNEL f1 ========<br>SFO1 128.3776052 MHz<br>NUC1 11B<br>P1 17.05 usec<br>PLW1 11.69499969 W<br><br>F2 - Processing parameters<br>SI 32768<br>SF 128.3776052 MHz<br>WDW no<br>SSB 0<br>LB 0 Hz<br>GB 0<br>PC 1.40	F2 - Acquisition Parameters<br>Date_ 20180221<br>Time 20.24<br>INSTRUM spect<br>PROBHD 5 mm PABBO BB-<br>PULPROG zg<br>TD 65536<br>SOLVENT D2O<br>NS 128<br>DS 4<br>SWH 25510.203 Hz<br>FIDRES 0.389255 Hz<br>AQ 1.2845056 sec<br>RG 192.58<br>DW 19.600 usec<br>DE 6.50 usec<br>TE 298.1 K<br>D1 1.00000000 sec<br>TD0 1<br><br>======== CHANNEL f1 ========<br>SFO1 128.3776052 MHz<br>NUC1 11B<br>P1 17.05 usec<br>PLW1 11.69499969 W<br><br>F2 - Processing parameters<br>SI 32768<br>SF 128.3776052 MHz<br>WDW no<br>SSB 0<br>LB 0 Hz<br>GB 0<br>PC 1.40

**COSY**	F2 - Acquisition Parameters	F2 - Acquisition Parameters	F2 - Acquisition Parameters
	Date_ 20171025	Date_ 20180209	Date_ 20180221
	Time 15.58	Time 15.48	Time 16.25
	INSTRUM spect	INSTRUM spect	INSTRUM spect
	PROBHD 5 mm PABBO BB-	PROBHD 5 mm PABBO BB-	PROBHD 5 mm PABBO BB-
	PULPROG cosygpqf	PULPROG cosygpqf	PULPROG cosygpqf
	TD 2048	TD 2048	TD 2048
	SOLVENT CDCl3	SOLVENT D2O	SOLVENT D2O
	NS 1	NS 1	NS 1
	DS 8	DS 8	DS 8
	SWH 4807.692 Hz	SWH 4807.692 Hz	SWH 4807.692 Hz
	FIDRES 2.347506 Hz	FIDRES 2.347506 Hz	FIDRES 2.347506 Hz
	AQ 0.2129920 sec	AQ 0.2129920 sec	AQ 0.2129920 sec
	RG 86.04	RG 192.58	RG 192.58
	DW 104.000 usec	DW 104.000 usec	DW 104.000 usec
	DE 6.50 usec	DE 6.50 usec	DE 6.50 usec
	TE 298.0 K	TE 298.1 K	TE 298.1 K
	D0 0.00000300 sec	D0 0.00000300 sec	D0 0.00000300 sec
	D1 1.48689198 sec	D1 1.48689198 sec	D1 1.48689198 sec
	D13 0.00000400 sec	D13 0.00000400 sec	D13 0.00000400 sec
	D16 0.00020000 sec	D16 0.00020000 sec	D16 0.00020000 sec
	IN0 0.00020800 sec	IN0 0.00020800 sec	IN0 0.00020800 sec
	======== CHANNEL f1 ========	======== CHANNEL f1 ========	======== CHANNEL f1 ========
	SFO1 400.1322007 MHz	SFO1 400.1322007 MHz	SFO1 400.1322007 MHz
	NUC1 1H	NUC1 1H	NUC1 1H
	P0 15.00 usec	P0 15.00 usec	P0 15.00 usec
	P1 15.00 usec	P1 15.00 usec	P1 15.00 usec
	PLW1 12.50300026 W	PLW1 12.50300026 W	PLW1 12.50300026 W
	====== GRADIENT CHANNEL =====	====== GRADIENT CHANNEL =====	====== GRADIENT CHANNEL =====
	GPNAM[1] SMSQ10.100	GPNAM[1] SMSQ10.100	GPNAM[1] SMSQ10.100
	GPZ1 10.00 %	GPZ1 10.00 %	GPZ1 10.00 %
	P16 1000.00 usec	P16 1000.00 usec	P16 1000.00 usec
	F1 - Acquisition parameters	F1 - Acquisition parameters	F1 - Acquisition parameters
	TD 128	TD 128	TD 128
	SFO1 400.1322 MHz	SFO1 400.1322 MHz	SFO1 400.1322 MHz
	FIDRES 75.120193 Hz	FIDRES 75.120193 Hz	FIDRES 75.120193 Hz
	SW 12.015 ppm	SW 12.015 ppm	SW 12.015 ppm
	FnMODE QF	FnMODE QF	FnMODE QF
	F2 - Processing parameters	F2 - Processing parameters	F2 - Processing parameters
	SI 1024	SI 1024	SI 1024
	SF 400.1300631 MHz	SF 400.1299556 MHz	SF 400.1300000 MHz
	WDW SINE	WDW SINE	WDW SINE
	SSB 0	SSB 0	SSB 0
	LB 0 Hz	LB 0 Hz	LB 0 Hz
	GB 0	GB 0	GB 0
	PC 1.40	PC 1.40	PC 1.40
	F1 - Processing parameters	F1 - Processing parameters	F1 - Processing parameters
	SI 1024	SI 1024	SI 1024
	MC2 QF	MC2 QF	MC2 QF
	SF 400.1300630 MHz	SF 400.1299552 MHz	SF 400.1300000 MHz
	WDW SINE	WDW SINE	WDW SINE
	SSB 0	SSB 0	SSB 0
	LB 0 Hz	LB 0 Hz	LB 0 Hz
	GB 0	GB 0	GB 0

HSQC			
F2 - Acquisition Parameters			
Date_	20171025	20180213	20180221
Time	16.28	23.06	20.03
INSTRUM	spect	spect	spect
PROBHD	5 mm PABBO BB-	5 mm PABBO BB-	5 mm PABBO BB-
PULPROG	hsqcetgpsisp2.2	hsqcetgpsisp2.2	hsqcetgpsisp2.2
TD	2048	2048	2048
SOLVENT	CDCl3	D2O	D2O
NS	2	2	2
DS	16	16	16
SWH	5341.880 Hz	5341.880 Hz	5341.880 Hz
FIDRES	2.608340 Hz	2.608340 Hz	2.608340 Hz
AQ	0.1916928 sec	0.1916928 sec	0.1916928 sec
RG	192.58	192.58	192.58
DW	93.600 usec	93.600 usec	93.600 usec
DE	6.50 usec	6.50 usec	6.50 usec
TE	298.3 K	298.0 K	298.0 K
CNST2	145.0000000	145.0000000	145.0000000
CNST17	-0.5000000	-0.5000000	-0.5000000
D0	0.00000300 sec	0.00000300 sec	0.00000300 sec
D1	1.50000000 sec	1.50000000 sec	1.50000000 sec
D4	0.00172414 sec	0.00172414 sec	0.00172414 sec
D11	0.03000000 sec	0.03000000 sec	0.03000000 sec
D16	0.00020000 sec	0.00020000 sec	0.00020000 sec
D24	0.00086207 sec	0.00086207 sec	0.00086207 sec
IN0	0.00003000 sec	0.00003000 sec	0.00003000 sec
======== CHANNEL f1 ========			
SFO1	400.1324057 MHz	400.1324057 MHz	400.1324057 MHz
NUC1	1H	1H	1H
P1	15.00 usec	15.00 usec	15.00 usec
P2	30.00 usec	30.00 usec	30.00 usec
P28	1000.00 usec	1000.00 usec	1000.00 usec
PLW1	12.50300026 W	12.50300026 W	12.50300026 W
======== CHANNEL f2 ========			
SFO2	100.6202713 MHz	100.6202713 MHz	100.6202713 MHz
NUC2	13C	13C	13C
CPDPRG[2	bi_p5m4sp_4sp.2	bi_p5m4sp_4sp.2	bi_p5m4sp_4sp.2
P3	9.80 usec	9.80 usec	9.80 usec
P14	500.00 usec	500.00 usec	500.00 usec
P24	2000.00 usec	2000.00 usec	2000.00 usec
P63	1500.00 usec	1500.00 usec	1500.00 usec
PLW0	0 W	0 W	0 W
PLW2	60.95399857 W	60.95399857 W	60.95399857 W
PLW12	0.91469002 W	0.91469002 W	0.91469002 W
SPNAM[3]	Crp60,0.5,20.1	Crp60,0.5,20.1	Crp60,0.5,20.1
SPOAL3	0.500	0.500	0.500
SPOFFS3	0 Hz	0 Hz	0 Hz
SPW3	8.94419956 W	8.94419956 W	8.94419956 W
SPNAM[7]	Crp60comp.4	Crp60comp.4	Crp60comp.4
SPOAL7	0.500	0.500	0.500
SPOFFS7	0 Hz	0 Hz	0 Hz
SPW7	8.94419956 W	8.94419956 W	8.94419956 W
SPNAM[14	Crp32,1.5,20.2	Crp32,1.5,20.2	Crp32,1.5,20.2
SPOAL14	0.500	0.500	0.500
SPOFFS14	0 Hz	0 Hz	0 Hz
SPW14	3.81620002 W	3.81620002 W	3.81620002 W
SPNAM[31	Crp32,1.5,20.2	Crp32,1.5,20.2	Crp32,1.5,20.2
SPOAL31	0.500	0.500	0.500
SPOFFS31	0 Hz	0 Hz	0 Hz
SPW31	0.95405000 W	0.95405000 W	0.95405000 W
====== GRADIENT CHANNEL =====			
GPNAM[1]	SMSQ10.100	SMSQ10.100	SMSQ10.100
GPNAM[2]	SMSQ10.100	SMSQ10.100	SMSQ10.100
GPNAM[3]	SMSQ10.100	SMSQ10.100	SMSQ10.100
GPNAM[4]	SMSQ10.100	SMSQ10.100	SMSQ10.100
GPZ1	80.00 %	80.00 %	80.00 %
GPZ2	20.10 %	20.10 %	20.10 %
GPZ3	11.00 %	11.00 %	11.00 %
GPZ4	-5.00 %	-5.00 %	-5.00 %
P16	1000.00 usec	1000.00 usec	1000.00 usec
P19	600.00 usec	600.00 usec	600.00 usec
F1 - Acquisition parameters			
TD	256	256	256
SFO1	100.6203 MHz	100.6203 MHz	100.6203 MHz
FIDRES	130.208328 Hz	130.208328 Hz	130.208328 Hz
SW	165.639 ppm	165.639 ppm	165.639 ppm
FnMODE	Echo-Antiecho	Echo-Antiecho	Echo-Antiecho
F2 - Processing parameters			
SI	1024	1024	1024
SF	400.1300624 MHz	400.1299619 MHz	400.1300000 MHz
WDW	QSINE	QSINE	QSINE
SSB	2	2	2
LB	0 Hz	0 Hz	0 Hz
GB	0	0	0
PC	1.40	1.40	1.40
F1 - Processing parameters			
SI	1024	1024	1024
MC2	echo-antiecho	echo-antiecho	echo-antiecho
SF	100.6127604 MHz	100.6127690 MHz	100.6127690 MHz
WDW	QSINE	QSINE	QSINE
SSB	2	2	2
LB	0 Hz	0 Hz	0 Hz
GB	0	0	0

HMBC			
	F2 - Acquisition Parameters	F2 - Acquisition Parameters	F2 - Acquisition Parameters
	Date_ 20171025	Date_ 20180209	Date_ 20180221
	Time 16.11	Time 15.54	Time 16.31
	INSTRUM spect	INSTRUM spect	INSTRUM spect
	PROBHD 5 mm PABBO BB-	PROBHD 5 mm PABBO BB-	PROBHD 5 mm PABBO BB-
	PULPROG hmbcgpl2ndqf	PULPROG hmbcgpl2ndqf	PULPROG hmbcgpl2ndqf
	TD 2048	TD 2048	TD 2048
	SOLVENT CDCl3	SOLVENT D2O	SOLVENT D2O
	NS 4	NS 4	NS 4
	DS 16	DS 16	DS 16
	SWH 4807.692 Hz	SWH 4807.692 Hz	SWH 4807.692 Hz
	FIDRES 2.347506 Hz	FIDRES 2.347506 Hz	FIDRES 2.347506 Hz
	AQ 0.2129920 sec	AQ 0.2129920 sec	AQ 0.2129920 sec
	RG 192.58	RG 192.58	RG 192.58
	DW 104.000 usec	DW 104.000 usec	DW 104.000 usec
	DE 6.50 usec	DE 6.50 usec	DE 6.50 usec
	TE 298.2 K	TE 298.1 K	TE 298.1 K
	CNST6 125.0000000	CNST6 125.0000000	CNST6 125.0000000
	CNST7 165.0000000	CNST7 165.0000000	CNST7 165.0000000
	CNST13 7.5000000	CNST13 7.5000000	CNST13 7.5000000
	D0 0.00000300 sec	D0 0.00000300 sec	D0 0.00000300 sec
	D1 1.50000000 sec	D1 1.50000000 sec	D1 1.50000000 sec
	D6 0.06666667 sec	D6 0.06666667 sec	D6 0.06666667 sec
	D16 0.00020000 sec	D16 0.00020000 sec	D16 0.00020000 sec
	IN0 0.00002240 sec	IN0 0.00002240 sec	IN0 0.00002240 sec
	======== CHANNEL f1 ========	======== CHANNEL f1 ========	======== CHANNEL f1 ========
	SFO1 400.1322007 MHz	SFO1 400.1322007 MHz	SFO1 400.1322007 MHz
	NUC1 1H	NUC1 1H	NUC1 1H
	P1 15.00 usec	P1 15.00 usec	P1 15.00 usec
	P2 30.00 usec	P2 30.00 usec	P2 30.00 usec
	PLW1 12.50300026 W	PLW1 12.50300026 W	PLW1 12.50300026 W
	======== CHANNEL f2 ========	======== CHANNEL f2 ========	======== CHANNEL f2 ========
	SFO2 100.6228119 MHz	SFO2 100.6228119 MHz	SFO2 100.6228119 MHz
	NUC2 13C	NUC2 13C	NUC2 13C
	P3 9.80 usec	P3 9.80 usec	P3 9.80 usec
	PLW2 60.95399857 W	PLW2 60.95399857 W	PLW2 60.95399857 W
	====== GRADIENT CHANNEL =====	====== GRADIENT CHANNEL =====	====== GRADIENT CHANNEL =====
	GPNAM[1] SMSQ10.100	GPNAM[1] SMSQ10.100	GPNAM[1] SMSQ10.100
	GPNAM[2] SMSQ10.100	GPNAM[2] SMSQ10.100	GPNAM[2] SMSQ10.100
	GPNAM[3] SMSQ10.100	GPNAM[3] SMSQ10.100	GPNAM[3] SMSQ10.100
	GPNAM[4] SMSQ10.100	GPNAM[4] SMSQ10.100	GPNAM[4] SMSQ10.100
	GPNAM[5] SMSQ10.100	GPNAM[5] SMSQ10.100	GPNAM[5] SMSQ10.100
	GPNAM[6] SMSQ10.100	GPNAM[6] SMSQ10.100	GPNAM[6] SMSQ10.100
	GPZ1 50.00 %	GPZ1 50.00 %	GPZ1 50.00 %
	GPZ2 30.00 %	GPZ2 30.00 %	GPZ2 30.00 %
	GPZ3 40.10 %	GPZ3 40.10 %	GPZ3 40.10 %
	GPZ4 15.00 %	GPZ4 15.00 %	GPZ4 15.00 %
	GPZ5 -10.00 %	GPZ5 -10.00 %	GPZ5 -10.00 %
	GPZ6 -5.00 %	GPZ6 -5.00 %	GPZ6 -5.00 %
	P16 1000.00 usec	P16 1000.00 usec	P16 1000.00 usec
	F1 - Acquisition parameters	F1 - Acquisition parameters	F1 - Acquisition parameters
	TD 128	TD 128	TD 128
	SFO1 100.6228 MHz	SFO1 100.6228 MHz	SFO1 100.6228 MHz
	FIDRES 348.772308 Hz	FIDRES 348.772308 Hz	FIDRES 348.772308 Hz
	SW 221.833 ppm	SW 221.833 ppm	SW 221.833 ppm
	FnMODE QF	FnMODE QF	FnMODE QF
	F2 - Processing parameters	F2 - Processing parameters	F2 - Processing parameters
	SI 2048	SI 2048	SI 2048
	SF 400.1300628 MHz	SF 400.1299573 MHz	SF 400.1299618 MHz
	WDW SINE	WDW SINE	WDW SINE
	SSB 0	SSB 0	SSB 0
	LB 0 Hz	LB 0 Hz	LB 0 Hz
	GB 0	GB 0	GB 0
	PC 1.40	PC 1.40	PC 1.40
	F1 - Processing parameters	F1 - Processing parameters	F1 - Processing parameters
	SI 1024	SI 1024	SI 1024
	MC2 QF	MC2 QF	MC2 QF
	SF 100.6127463 MHz	SF 100.6127310 MHz	SF 100.6127690 MHz
	WDW SINE	WDW SINE	WDW SINE
	SSB 0	SSB 0	SSB 0
	LB 0 Hz	LB 0 Hz	LB 0 Hz
	GB 0	GB 0	GB 0

References

1. Boron Molecular, https://www.boronmolecular.com. A fine chemicals manufacturer.
2. Campkin, D.M.; Shimadate, Y.; Bartholomew, B.; Bernhardt, P.V.; Nash, R.J.; Sakoff, J.A.; Kato, A.; Simone, M. Borylated 2,3,4,5-Tetrachlorophthalimide and Their 2,3,4,5-Tetrachlorobenzamide Analogues: Synthesis, Their Glycosidase Inhibition and Anticancer Properties in View to Boron Neutron Capture Therapy. *Molecules* **2022**, *27*, 3447-3475, doi:https://doi.org/10.3390/molecules27113447.
3. Legge, W.J.; Shimadate, Y.; Sakoff, J.; Houston, T.A.; Kato, A.; Bernhardt, P.V.; Simone, M. Borylated methyl cinnamates: Green synthesis, characterization, crystallographic analysis and biological activities – in glycosidase inhibition and in cancer cells lines. *Beilstein Arch.* **2021**, 20214, doi:https://doi.org/10.3762/bxiv.2021.4.v1.
4. Simone, M. Diastereoselective Synthesis of the Borylated D-Galactose Monosaccharide 3-Boronic-3-Deoxy-D-Galactose and Biological Evaluation in Glycosidase Inhibition and in Cancer for Boron Neutron Capture Therapy (BNCT). *Molecules* **2023**, *28*, 4321-4337, doi:https://doi.org/10.3390/molecules28114321.
5. Simone, M. Borylated Monosaccharide 3-Boronic-3-deoxy-D-galactose: Detailed NMR Spectroscopic Characterisation, and Method for Spectroscopic Analysis of Anomeric and Boron Equilibria. *Internat. J. Mol. Sci.* **2024**, *25*, 12396, doi:https://doi.org/10.3390/ijms252212396.
